# Integrated analysis of phase 1a and 1b randomized controlled trials; Treg-targeted cancer immunotherapy with the humanized anti-CCR4 antibody, KW-0761, for advanced solid tumors

**DOI:** 10.1371/journal.pone.0291772

**Published:** 2023-09-20

**Authors:** Kaoru Fujikawa, Takuro Saito, Koji Kurose, Takashi Kojima, Takeru Funakoshi, Eiichi Sato, Kazuhiro Kakimi, Shinsuke Iida, Yuichiro Doki, Mikio Oka, Ryuzo Ueda, Hisashi Wada

**Affiliations:** 1 Department of Gastroenterological Surgery, Osaka University Graduate School of Medicine, Suita, Osaka, Japan; 2 Department of Respiratory Medicine, Kawasaki Medical School, Kurashiki, Okayama, Japan; 3 Department of Gastrointestinal Oncology, National Cancer Center Hospital East, Chiba, Japan; 4 Department of Dermatology, Keio University School of Medicine, Shinjuku-ku, Tokyo, Japan; 5 Department of Pathology, Institute of Medical Science (Medical Research Center), Tokyo Medical University, Shinjuku-ku, Tokyo, Japan; 6 Department of Immunotherapeutics, The University of Tokyo Hospital, Bunkyo-Ku, Tokyo, Japan; 7 Department of Hematology and Oncology, Nagoya City University Graduate School of Medical Sciences, Mizuho-cho, Mizuho-ku, Nagoya, Aichi, Japan; 8 Department of Immuno-Oncology, Kawasaki Medical School, Kurashiki, Okayama, Japan; 9 Department of Immunology, Nagoya University Graduate School of Medicine, Nagoya, Japan; 10 Department of Clinical Research in Tumor Immunology, Osaka University Graduate School of Medicine, Suita, Osaka, Japan; National Cancer Center, JAPAN

## Abstract

**Introduction:**

Regulatory T cells (Tregs) have attracted attention as a novel therapeutic target to augment the clinical efficacy of immunotherapy. We conducted phase Ia and Ib trials to examine the safety and efficacy of the anti-CCR4 antibody, KW-0761 (mogamulizumab), which may eliminate effector Tregs (eTregs). We herein overviewed the results of these trials, presented cases with a durable clinical response, and investigated factors associated with the clinical effects of KW-0761.

**Methods:**

Forty-nine patients with CCR4-negative solid cancers were enrolled in the phase Ia and Ib trials on KW-0761. An integral analysis of safety, clinical responses, prognosis, blood laboratory data, and cancer testis antigen-specific immune responses was performed.

**Results:**

Grade 3–4 treatment-related adverse events were reported in 21 (42.9%) out of 49 patients, all of which were manageable. A partial response and stable disease were observed in 1 and 9 patients, respectively. A durable clinical response was noted in 2 esophageal and 2 lung cancer patients. eTreg depletion in peripheral blood was confirmed in most patients, and eTreg depletion was sustained during the KW-0761 treatment. High lymphocyte levels at baseline and 2 weeks after the initiation of KW-0761 were associated with a favorable clinical outcome.

**Conclusions:**

A durable clinical response was noted in some patients, and high lymphocyte levels before treatment initiation may be a biomarker for the efficacy of KW-0761. The synergistic effect of KW-0761 for depleting Tregs and other immunotherapies is expected in the future.

## Introduction

Cancer immunotherapy is now more frequently used in clinical practice and immune checkpoint inhibitors (ICIs) have improved the prognosis of patients with various cancer types; however, their efficacy is still insufficient [1–3]. Further development of ICIs is needed to improve treatment outcomes. FoxP3^+^CD4^+^ regulatory T cells (Tregs) suppress immune responses and their removal enhanced anti-tumor immunity, resulting in tumor regression in mice; therefore, Treg control has become a new target in cancer immunotherapy [4–6]. Miyara et al. functionally divided FoxP3^+^CD4^+^ T cells into three distinct populations, and FoxP3^hi^CD45RA^-^CD4^+^ T cells were defined as effector Tregs (eTregs) with high suppressive activity [7]. Extensive efforts have been made to identify antigens that are uniquely expressed on eTregs for the control of immunosuppression in cancer patients.

KW-0761 (mogamulizumab) is a humanized anti-C-C chemokine receptor type 4 (CCR4) IgG1 monoclonal antibody (mAb) with enhanced antibody-dependent cellular cytotoxicity. Since CCR4 is overexpressed in relapsed Adult T-cell leukemia-lymphoma, Mycosis Fungoides, and Sezary Syndrome [8], KW-0761 is now clinically used as a therapeutic agent for these diseases [9, 10]. Moreover, CCR4 is highly expressed in eTregs [6], and, thus, KW-0761 may selectively suppress eTregs via CCR4 and regulate cancer immunosuppression in patients treated with KW-0761. The selective depletion of Tregs was examined in phase Ia and Ib clinical trials on the effects of KW-0761 on CCR4-negative solid cancer, and we reported the outcomes of these trials separately [11–13]. In the present study, we overviewed phase Ia [11] and Ib trials [12, 13], presented cases with a durable clinical response, and investigated factors associated with the clinical effects of KW-0761.

## Materials and methods

### Patients

Patients were eligible if they had CCR4-negative advanced or recurrent solid cancer with target lesions. CCR4 expression was examined by immunohistochemistry (IHC) using an anti-CCR4 mAb (KM2160; Kyowa Kirin) and confirmed by a review committee with a central evaluation. The inclusion criteria were the same as those in previous studies [11–13].

### Study design

**Fig 1:**
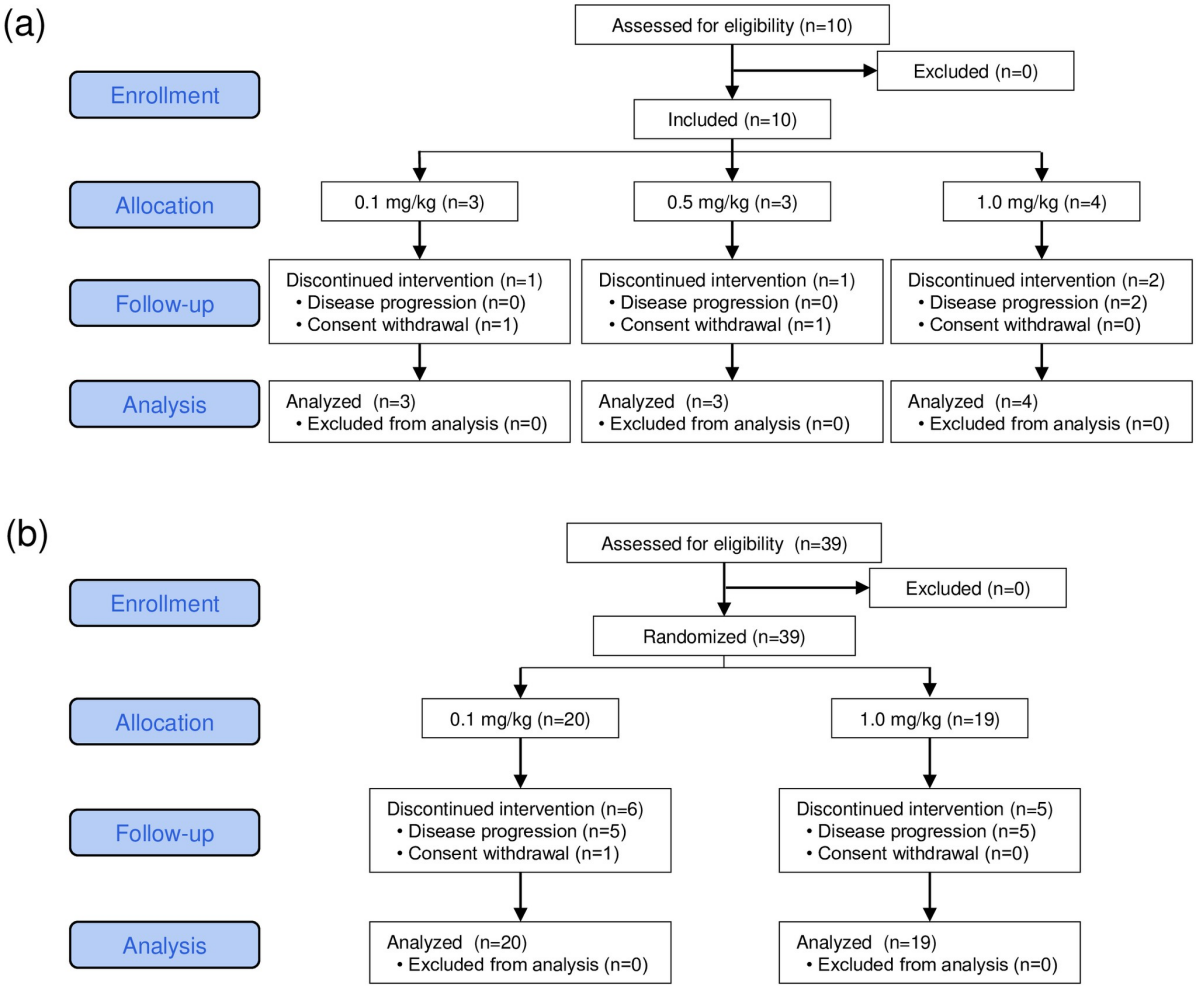
CONSORT diagram for trial. Phase Ia (a) and Phase Ib (b) studies.

The present study was designed as multi-institutional, open-label, investigator-initiated phase Ia and Ib clinical trials on KW-0761 (mogamulizumab). The investigational drug KW-0761 was provided by Kyowa Kirin. Patients received 8 intravenous infusions of KW-0761 weekly followed by monthly infusions until disease progression or patient refusal. The phase Ia study consisted of a non-randomized 3+3 dose-escalation design with 0.1, 0.5, and 1.0 mg/kg of KW-0761, and the sample size was set using Fibonacci’s method (Fig 1a). Based on our previous findings [9], 0.1 mg/kg was set as the minimal dose for Treg suppression. When dose-limiting toxicity (DLT) did not occur in any of the three patients in one dose group, the dose level was increased. Three, three, and four patients were assigned doses of 0.1, 0.5, and 1.0 mg/kg, respectively, and no DLT was observed [12]. According to these results, 0.1 and 1.0 mg/kg were set as the minimally and maximally tolerated doses, respectively, and were compared in the phase Ib study. The sample size of the phase Ib study was 20 patients per group, which was sufficient to evaluate Treg depletion. Randomization was performed with a dynamic random assignment by a registry center using a minimization method with cancer type as the assignment factor (Fig 1b). This was an open-label study with no blinding. Twenty and twenty-two patients were assigned doses of 0.1 and 1.0 mg/kg, respectively. The primary endpoints were the safety of the KW-0761 treatment and the effects of eTreg depletion in peripheral blood. The secondary endpoints were the overall response rate (ORR), progression-free survival (PFS), and overall survival (OS). The present study was registered with ClinicalTrials.gov as NCT01929486 and was approved by local Institutional Review Boards at Osaka University (124904), Kawasaki Medical School (250122), National Cancer Center Hospital East (K0288), Keio University Hospital (D13-01), Tokyo University (2013040-11DX), and Nagoya City University (31-13-0001). The independent data monitoring committee was chaired by Ryuzo Ueda, Department of Tumor Immunology, Aichi Medical University School of Medicine. The efficacy assessment committee was chaired by Koji Iwata, Deputy Director, Aichi Cancer Center Hospital. Patients were enrolled after obtaining written informed consent in accordance with the Declaration of Helsinki between February 12, 2013, and February 5, 2016.

### Toxicity evaluation

Toxicity was graded according to the National Cancer Institute Common Terminology Criteria for Adverse Events version 4.0. The independent data monitoring committee chaired by Professor Ryuzo Ueda, Department of Tumor Immunology, Aichi Medical University School of Medicine evaluated the safety data for each dose level.

### Clinical response evaluation

Responses were evaluated 12 weeks after the first KW-0761 treatment or at the point of study discontinuation based on computed tomography (CT) scans according to RECIST (ver. 1.1) and immune-related RECIST [14, 15]. OS was defined as the time from the day of the first KW-0761 treatment until the day of death from any cause. PFS was defined as the time from the day of the first KW-0761 treatment until either the day of progressive disease (PD) detection or death from any cause. The tumor response, OS, and PFS were confirmed by the efficacy assessment committee chaired by Dr. Koji Iwata, Deputy Director, Aichi Cancer Center Hospital with a central evaluation.

### Blood laboratory data

Peripheral blood sampling was performed at baseline and at every timepoint of KW-0761 infusions. Blood laboratory tests consisted of a blood count, alanine aminotransferase (ALT), aspartate aminotransferase (AST), alkaline phosphatase (ALP), lactate dehydrogenase (LDH), phosphate, albumin, γ-glutamyltransferase (GGT), amylase and thyroid stimulating hormone (TSH). The neutrophil-to-lymphocyte ratio (NLR), platelet-to-lymphocyte ratio (PLR), and monocyte-to-lymphocyte ratio (MLR) were defined as the values of the neutrophil, platelet, and monocyte counts divided by the lymphocyte count, respectively.

### Treg depletion effects on PBMCs

Treg depletion was assessed as previously described [11, 13, 16]. In brief, blood samples were obtained at baseline, 4 and 8 weeks after the first KW-0761 treatment, and every 4 weeks during the continuous treatment until the point of study discontinuation. PBMCs were isolated from heparinized blood by density gradient centrifugation using Ficoll-Paque Plus (GE Healthcare, Fairfield, CT). Cells were stored in liquid N2 until used. Treg depletion was evaluated by flow cytometry. After thawing, PBMCs were incubated with mAb at 4°C for 20 minutes. Cells were stained with anti-CD4-PerCP (clone SK3; BD Biosciences, San Jose, CA), anti-CD25-APC (clone 2A3; BD Biosciences), and anti-CD45RA-FITC (clone ALB11; Beckman Coulter, Brea, CA) mAbs. The intracellular staining of FoxP3 was performed with anti-FoxP3-PE (clone PCH101; eBioscience) mAb and a FoxP3/Transcription Factor Staining Buffer Set (eBioscience, San Diego, CA) according to the manufacturer’s instructions. After the incubation, cells were washed and analyzed by FACS Calibur (BD Biosciences). CD45RA^+^ FoxP3^lo^ resting/naïve Tregs, CD45RA^-^FoxP3^hi^ activated/effector Tregs (eTregs), and CD45RA^-^FoxP3^lo^ non-Tregs were analyzed as previously described [7].

### IHC

Archived or newly obtained tumor samples from patients were screened for the expression of CCR4, NY-ESO-1, and XAGE1 (GAGED2a) by IHC as previously described [16–18].

### Enzyme-linked Immunosorbent Assay (ELISA) for NY-ESO-1 or XAGE1 antibodies

Serum samples were obtained at baseline, 4 and 8 weeks after the first KW-0761 treatment, and every 4 weeks during the continuous treatment until the point of study discontinuation. Recombinant NY-ESO-1 and the synthetic XAGE1 protein (1 μM) in ELISA Buffer kit (PeproTech, Rocky Hill, NJ) were adsorbed onto a 96-well ELISA plate (Nunc, Roskilde, Denmark) and incubated at 4°C overnight. Plates were washed with 0.05% Tween-20 in PBS and blocked with 1% BSA/PBS (200 μl/well) at room temperature for 1 hour. After washing, 100 μl of serially diluted serum was added to each well and incubated at room temperature for 2 hours. After washing, horseradish peroxidase-conjugated goat anti-human IgG (MBL, Nagoya, Japan) was added to the wells, and the plates were incubated at room temperature for 1 hour. After washing and development, absorbance was read at 490 nm [12, 19].

### Statistical analysis

Quantitative data without a normal distribution were analyzed using the 2-tailed non-parametric Mann-Whitney U test. The 2-tailed Fisher’s exact probability test was used for bivariate analyses. Cumulative survival was plotted using the Kaplan–Meier method, and differences were compared using the Log-rank test. Statistical analyses were performed using JMP Pro, version 16.0.0 (JMP, Tokyo). P values <0.05 were considered to be significant.

## Results

### Patient characteristics

Between October 2013 and April 2016, 49 patients with CCR4-negative advanced solid cancer were assigned to receive the KW-0761 treatment at doses of 0.1 mg/kg (n = 23), 0.5 mg/kg (n = 3), and 1.0 mg/kg (n = 23) in the phase I study (Fig 1, S1 Table). The cohort included esophageal cancer (n = 14), lung cancer (n = 16), malignant melanoma (n = 6), gastric cancer (n = 5), ovarian cancer (n = 5), and mesothelioma (n = 3). The initial 8 infusions were completed by 35 patients and additional monthly infusions were administered to 9 patients, including esophageal cancer (n = 4), lung cancer (n = 3), and mesothelioma (n = 2). The median number of KW-0761 infusions was 8 (range, 2–23) and the median follow-up duration was 114 days (range, 21–1024 days). All allocated patients were analyzed for primary and secondary endpoints.

### Adverse events (AEs)

All grade and grade 3–4 treatment-related AEs occurred in 46 (93.9%) and 21 (42.9%), respectively, out of 49 patients (S1 Table). In total, 163 treatment-related AEs were observed, with the most frequent categories being skin disorders (n = 34) and lymphopenia (n = 34) (Table 1). There were no grade 3–4 skin disorders, while 15 patients developed grade 3–4 lymphopenia. The frequency of treatment-related AEs did not significantly differ between the 0.1, 0.5, and 1.0 mg/kg cohorts. All treatment-related AEs were manageable or recovered without any treatment, and there were no drug-related deaths.

**Table 1:** Reagent-related adverse events.

	Grade	0.1 mg/kg (N = 23)			0.5 mg/kg (N = 3)			1.0 mg/kg (N = 23)		
		1–2	3	4	1–2	3	4	1–2	3	4
Total events		67	8	1	14	2		60	9	2
Non-hematological										
Skin and subcutaneous tissue										
	Rash	15	1		2			12		
	Pruritus	1								
	Drug eruption							1		
	Erythema	2								
	Papules							1		
	Cutaneous papilloma				1					
Hematological										
	Lymphopenia	12	4	1		2		7	5	2
	Hypophosphatemia	1							1	
	GGT increased		1						2	
	ALT increased	3	1							
	AST increased	2	1		1					

ALT, alanine aminotransferase; AST, aspartate aminotransferase; GGT, Gamma-glutamyltransferase

### Clinical responses

**Fig 2:**
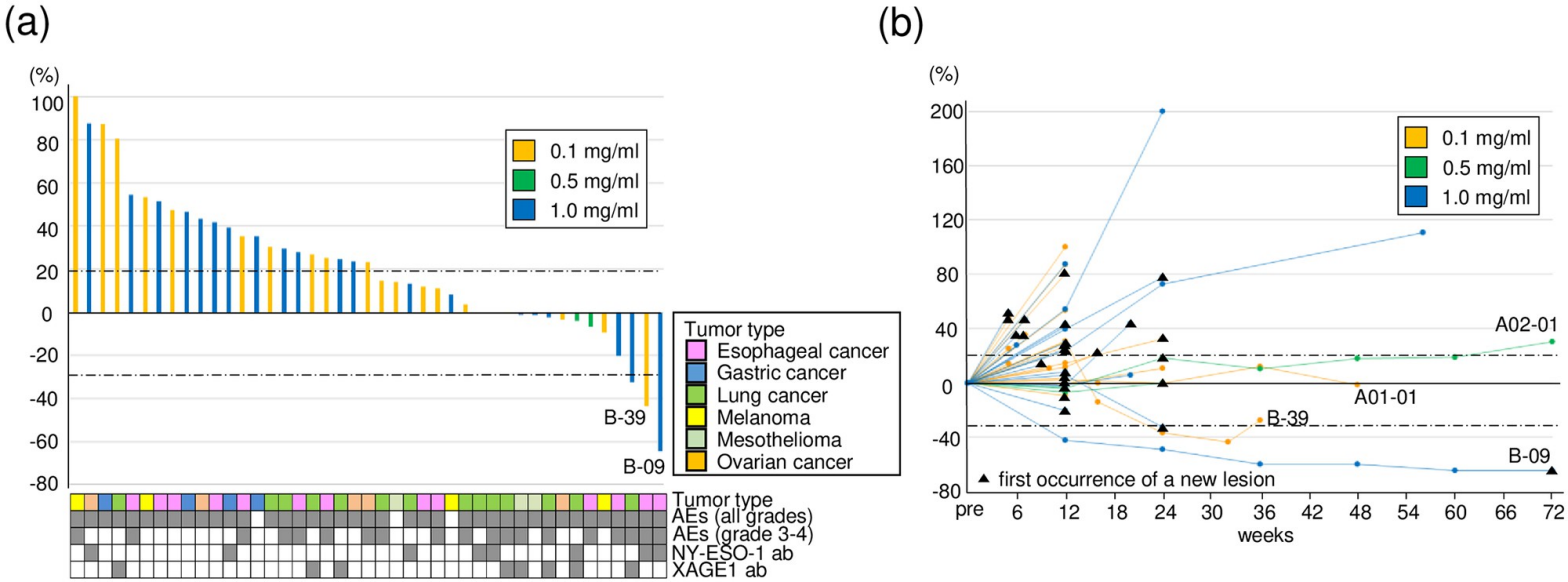
Characteristics of clinical responses. (a) A Waterfall plot for the best percentage change from baseline. A positive change in the tumor burden indicates tumor growth, while a negative change reflects a tumor reduction. The tumor type, presence or absence of adverse events, and presence or absence of NY-ESO-1 and XAGE1 antibody responses are annotated for each patient. (b) A Spaghetti plot for the percentage change in the target lesion tumor burden from baseline over time. Tumor burden was measured as the sum of the longest diameters of the target lesions by patients over time. The black triangle indicates the first occurrence of a new lesion. Horizontal dotted lines denote a 30% decrease and a 20% increase. 0.1 mg/ml (n = 23), blue bar; 0.5 mg/ml (n = 3), green bar; 1.0 mg/ml (n = 23), orange bar.

Clinical responses were 1 partial response (PR), 9 stable disease (SD), and 39 PD (Fig 2a and 2b, S1 Table). Median PFS and OS were 65 days (range, 21–491 days) and 114 days (range, 21–1024 days), respectively. No significant differences were observed in survival between the 0.1, 0.5, and 1.0 mg/kg cohorts (S1a and S1b Fig), whereas PFS and OS were significantly longer in patients with PR or SD than in those with PD (S1c and S1d Fig). A durable clinical response was observed in 2 patients with esophageal cancer (B-09 and B-39) and 2 with lung cancer (A1-01 and A2-01) (Fig 2b) [12].

**Fig 3:**
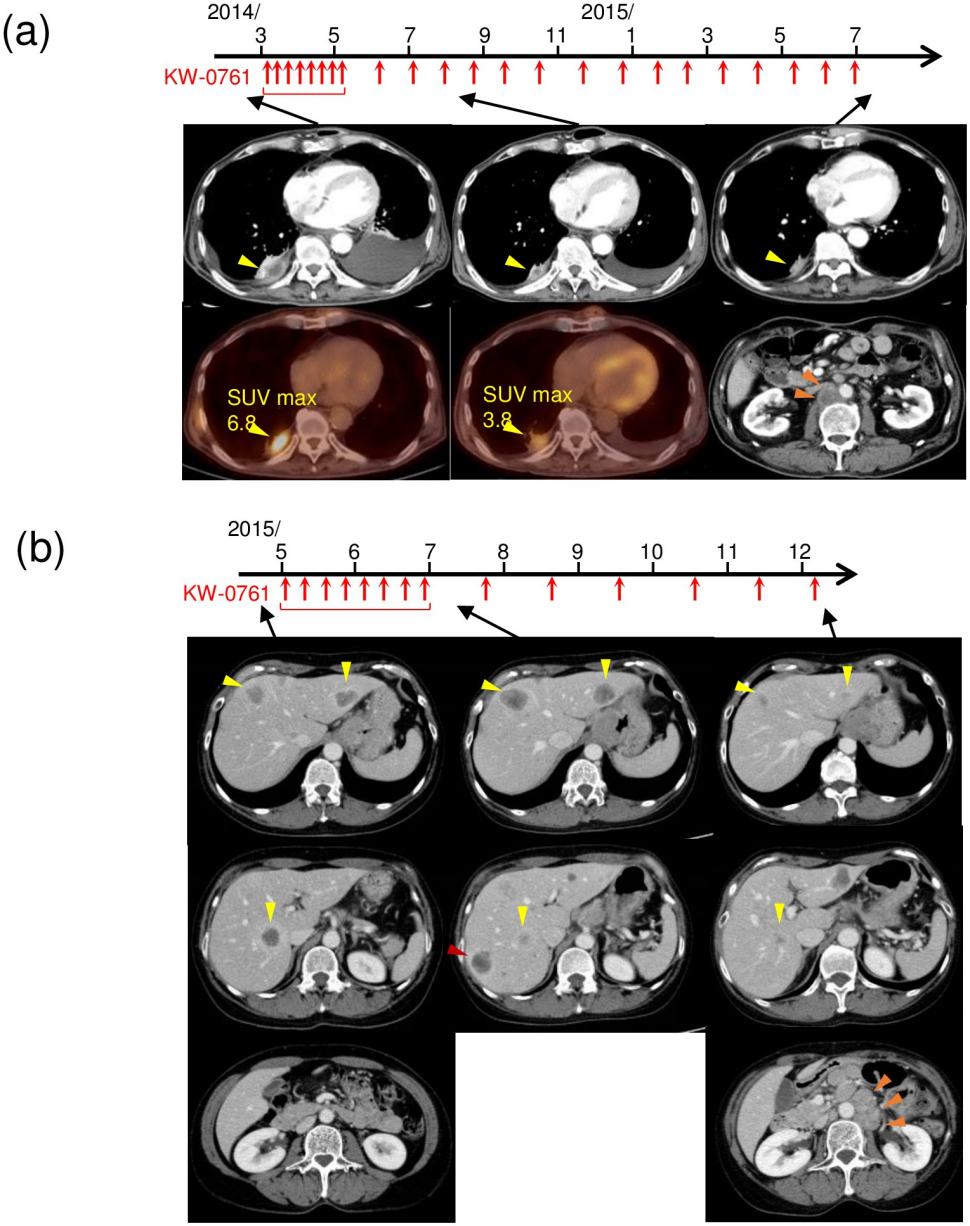
Representative CT imaging in patients with a durable clinical response. (a) Esophageal cancer patient B-09. Pleural metastasis (yellow arrow) was observed on CT and FDG-PET at baseline. The pleural tumor was decreased in size by the KW-0761 treatment, which was also confirmed by a reduction in standardized uptake value-max (SUV-max) on FDG-PET. Although pleural metastasis shrank, abdominal lymph node metastasis (orange arrow) developed after 23 infusions, which was evaluated as a new lesion, leading to the discontinuation of treatment. (b) Esophageal cancer patient B-39. Multiple liver metastases (yellow arrow) were observed on CT at baseline. Disease progression and a new lesion in the liver (red arrow) were confirmed after the first 8 infusions; however, all lesions subsequently decreased in size. The objective feasible response was sustained until 36 weeks after the first treatment when abdominal lymph node metastasis developed (orange arrow).

Patient B-09 was a 64-year-old male with chemotherapy-resistant relapsed esophageal cancer metastasis in the pleura at the baseline (Fig 3a). CT showed reductions in the pleural tumor and pleural fluid after the initiation of KW-0761 and a decrease in the standardized uptake value-max (SUV-max) in the pleural tumor was confirmed using FDG-positron emission tomography (PET), which lead to the diagnosis of PR. However, abdominal lymph node metastasis developed after 23 infusions, which was evaluated as a new lesion and led to the discontinuation of treatment. PR lasted for 72 weeks until the new lesion was confirmed (Fig 2b).

Patient B-39 was a 59-year-old female with chemotherapy-resistant relapsed metastasis in the liver from esophageal cancer (Fig 3b). CT showed disease progression and a new lesion in the liver after the first 8 infusions, based on which PD was diagnosed. However, since some target lesions shrank, KW-0761 was continued beyond PD and all lesions subsequently decreased in size. Abdominal lymph node metastasis developed after 14 infusions, which led to the discontinuation of treatment; however, a durable clinical response was maintained until 36 weeks after the initiation of KW-0761.

Although 2 patients with esophageal cancer showed a durable clinical response, there was no significant prognostic difference between patients with esophageal cancer and those with other cancer types (S2 Fig).

### FoxP3^+^CD4^+^ Treg depletion

eTreg depletion in peripheral blood by KW-0761 was examined at baseline and during the KW-0761 treatment by flow cytometry. The percentage of eTregs in CD4^+^ T cells markedly decreased 4 weeks after the initiation of KW-0761 in all patients, except for 2 patients whose post-treatment samples were not available (S3 Fig). The median percentage of eTregs in CD4^+^ T cells was 2.1% (range, 0.45–6.1%) at baseline and 0.20% (range, 0.04–0.92%) 4 weeks after the initiation of KW-0761. Prolonged Treg depletion was observed in patients with a durable clinical response (B-09, B-39, A1-01). In other patients, the percentage of eTregs in peripheral blood remained low during the KW-0761 treatment and increased a few weeks after KW-0761 infusions were stopped.

### Blood laboratory data

**Fig 4:**
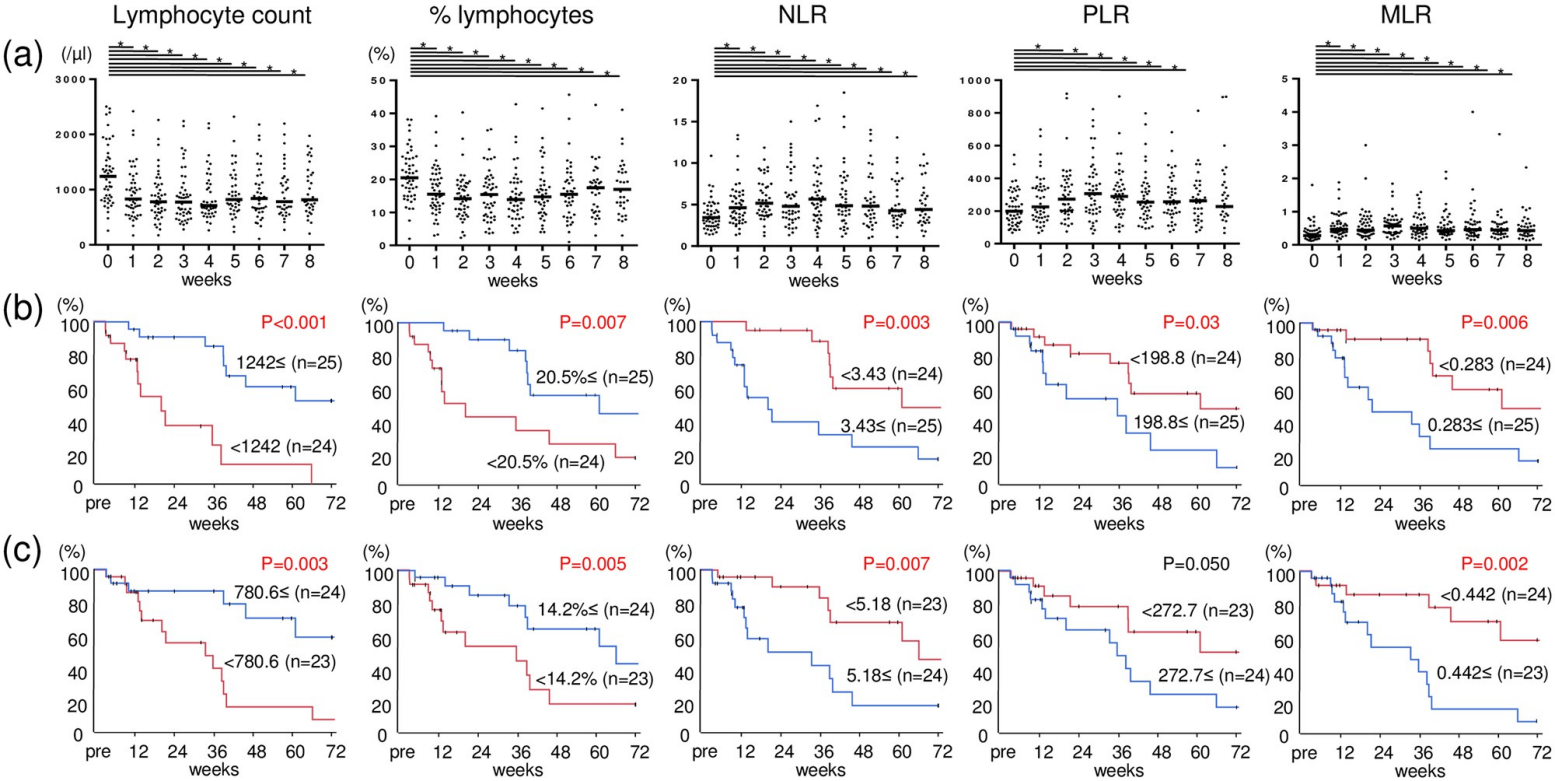
Changes in blood laboratory data and correlations for overall survival. (a) The lymphocyte count (/μl), percent lymphocytes in leukocytes (% lymphocytes), NLR, MLR, and PLR were assessed by blood laboratory tests at baseline and weekly after the first KW-0761 treatment until 8 infusions. Kaplan-Meier curves of OS were analyzed between high and low groups divided by the median values of calculated parameters at baseline (b) and 2 weeks after the initiation of KW-0761 (c). Bar, median; *, p<0.05.

The lymphocyte count, percent lymphocytes in leukocytes (% lymphocytes), NLR, MLR, and PLR were analyzed by blood laboratory tests at baseline and weekly after the initiation of KW-0761 until 8 infusions. The lymphocyte count and % lymphocytes markedly decreased immediately after the initiation of KW-0761, along with rapid increases in NLR, PLR, and MLR (Fig 4a). The median values of each parameter were 1242 (/μl), 20.5%, 3.43, 198.8, and 0.283, respectively, at baseline and 780.6 (/μl), 14.2%, 5.18, 272.7, and 0.442, respectively, 2 weeks after the initiation of KW-0761. Patients with the clinical response of SD or PR had a lower NLR at baseline and 2 weeks after the initiation of KW-0761, and tended to have a higher lymphocyte count and % lymphocytes at baseline, and higher % lymphocytes 2 weeks after the initiation of KW-0761 (Table 2). Moreover, a higher lymphocyte count or lower ratio of lymphocytes to other blood cells at baseline or 2 weeks after the initiation of KW-0761 correlated with better OS (Fig 4b and 4c).

**Table 2:** Blood laboratory data and clinical responses.

			PD	SD, PR	p-value ^a^
Pre	Lymphocyte count (/μl)	<1242.3 / 1242.3≤	22 / 17	2 / 8	0.07
	% lymphocyte	<20.5 / 20.5≤	22 /17	2 / 8	0.07
	NLR	<3.43 / 3.43≤	16 / 23	8 / 2	0.04
	PLR	<198.8 / 198.8≤	17 / 22	7 / 3	0.17
	MLR	<0.283 / 0.283≤	17 / 22	7 / 3	0.17
Week 2	Lymphocyte count (/μl)	<780.6 / 780.6≤	19 / 18	4 / 6	0.72
	% lymphocyte	<14.2 / 14.2≤	21 / 16	2 / 8	0.07
	NLR	<5.18 / 5.18≤	15 / 22	8 / 2	0.04
	PLR	<272.7 / 272.7≤	17 / 20	6 / 4	0.49
	MLR	<0.442 / 0.442≤	16 / 21	7 / 3	0.17

Abbreviations: NLR, Neutrophil/Lymphocyte Ratio; PLR, Platelet/Lymphocyte Ratio; MLR, Monocyte/Lymphocyte Ratio.

^a^ The 2-tailed Fisher’s exact probability test

### Cancer-testis (CT) antigen-specific antibody responses

Serum NY-ESO-1 and XAGE1 antibodies at baseline and after the first 8 infusions or at the time of the discontinuation of treatment were analyzed by ELISA in patients with less than 8 infusions (S1 Table). Among 49 patients, 8 showed the production of the NY-ESO-1 antibody at baseline (sero-positive), 6 of whom expressed the tumor NY-ESO-1 antigen. Six sero-positive patients and 1 sero-negative patient (B-09) showed increased O.D. values during treatment (S1 Table, S4 Fig). Regarding XAEG1, 8 patients showed the production of the XAGE1 antibody at baseline (sero-positive), 7 of whom expressed the tumor XAGE1 antigen. Two sero-positive patients showed an increased XAGE1 antibody response during treatment (S1 Table, S4 Fig). An increased NY-ESO-1 or XAGE1 antibody response after treatment did not correlate with clinical responses or OS, whereas the baseline XAGE1 antibody response was associated with better OS (S1 Table, S5 Fig).

## Discussion

We conducted phase Ia and Ib clinical trials on KW-0761 to examine the selective depletion of Tregs in 49 patients with CCR4-negative solid cancer [11–13]. An integrated analysis was performed with an emphasis on the presentation of patients with a durable clinical response and the examination of biomarkers, such as lymphocyte and antibody responses against CT antigens. Several treatment-related AEs were observed, including grade 3–4 lymphopenia and grade 1–2 skin disorders; however, all were manageable and there were no drug-related deaths. eTregs in peripheral blood were depleted in most patients and a durable clinical response by KW-0761 was observed in some patients along with prolonged eTreg depletion. Four patients achieved a durable clinical response. Higher lymphocyte counts or a higher ratio of lymphocytes in peripheral blood predicted a better clinical response and prognosis for patients treated with KW-0761.

Strategies targeting Tregs to increase anti-tumor immunity, including antibodies against CTLA-4, OX40, 4-1BB, and ICOS, have attracted attention [3, 20, 21]. Although drugs targeting Tregs have not yet been approved, preclinical studies indicated that one of the mechanisms underlying the anti-tumor effects of anti-CTLA-4 mAb is Treg depletion [22, 23]. In this clinical trial, KW-0761 clearly depleted eTregs in peripheral blood; however, most patients did not show tumor regression. A possible explanation for the low clinical efficacy of KW-0761 is the unexpected decrease in central memory CD8^+^ T cells, which also express CCR4 and have anti-tumor immunity [11]. KW-0761 may concurrently deplete eTregs and central memory CD8^+^ T cells, and this dual depletion may cancel anti-tumor immune responses. Another plausible explanation for the impaired clinical responses of KW-0761 was that eTregs were not sufficiently depleted in the tumor microenvironment. The number of Tregs in peripheral blood does not necessarily reflect the abundance of Tregs in tumors. However, we observed a reduction in Tregs in the tumor of a patient whose tumor biopsy specimens were available at baseline and post-treatment (B-24) [11]. eTregs remained low during the KW-0761 treatment, particularly in patients with a durable clinical response. Therefore, eTregs in tumors may have been depleted by the KW-0761 treatment, leading to a durable clinical response in some patients.

Treg reductions by KW-0761 have been analyzed in trials on adult T-cell leukemia/lymphoma, primary peripheral T-cell lymphoma, and HTLV-1-associated myelopathy-tropical spastic paraparesis, in which a wide range of dosages (0.003–1.0 mg/kg) of KW-0761 were examined [9, 24]. A significant reduction in Tregs in peripheral blood was observed even at lower dosages, along with a lower incidence of AEs. In our trial, we adopted 3 dosages: 0.1, 0.5, and 1.0 mg/kg. Efficient Treg depletion in peripheral blood and a durable clinical response was observed even in the 0.1 mg/kg cohort. *In vitro* and *in vivo* experiments showed that eTregs and central memory CD8^+^ T cells were equally impaired at high concentrations of KW-0761, whereas central memory CD8^+^ T cells were less impaired at low concentrations [11]. Therefore, excess doses of KW-0761 may reduce eTregs and unexpectedly deplete central-memory CD8^+^ T cells expressing CCR4. It may be important to optimize the amount of KW-0761 in order to achieve the selective reduction of eTregs and avoid the unexpected depletion of beneficial CD8^+^ T cells. Further trials are warranted to select appropriate doses that are effective against solid cancers. In the future, trials with lower doses of KW-0761 are needed to demonstrate its overall benefit to patients with solid cancers.

CT antigens, such as NY-ESO-1 and XAGE1, are known to show unique expression patterns and induce spontaneous humoral and cellular immune responses in various cancer patients [25]. Since an evaluation of CT antigen-specific immune responses is useful for monitoring the induction of anti-tumor immunity by immunotherapy, we previously used CT antigen-specific immune responses for immune monitoring in cancer vaccine clinical trials [26–28]. In this trial, we hypothesized that the induction of anti-tumor immunity by the KW-0761 treatment may be monitored by analyzing CT antigen-specific immune responses in patients with CT antigen-expressing tumors. Increases in the humoral immune responses of NY-ESO-1 and XAGE1 were observed in some patients. Additionally, strong NY-ESO-1 or XAGE-1 antibody responses were observed in 4 patients with a durable clinical response during the KW-0761 treatment (A1-01, A2-01, B-09, and B-39) (S4a Fig) [10], suggesting the potential to monitor an enhanced anti-tumor immune response. However, neither NY-ESO-1 nor XAGE1 antibody production correlated with clinical effects. This may be because only a limited number of patients achieved clinical benefits from the KW-0761 treatment. Therefore, accurate monitoring methods need to be employed for the KW-0761 treatment.

Regarding predictive markers of treatment efficacy, our group previously reported that serum NY-ESO-1 and XAGE1 antibody responses predicted good clinical responses with an anti-PD-1 treatment for non-small cell lung cancer [29]. However, neither NY-ESO-1 nor XAGE1 antibody expression at baseline correlated with clinical responses to the KW-0761 treatment. In contrast, higher lymphocyte counts and a lower ratio of lymphocytes to other populations of blood cells assessed by NLR, PLR, and MLR correlated with better clinical responses and prognoses. Lymphopenia in blood laboratory tests was observed along with Treg depletion soon after treatment initiation, which may support the above considerations that the efficacy of KW-0761 depends on the balance between the depletion of Tregs and other types of lymphocytes. However, 4 patients with a durable response showed long-term Treg depletion and had significantly higher pretreatment lymphocyte counts than patients without a durable response. In addition, a high lymphocyte count or higher % lymphocytes before treatment correlated with a better treatment response. Since immunity is considered to be preserved in patients with high lymphocyte counts, it may have contributed to greater treatment efficacy, resulting in a good prognosis. Thus, pretreatment lymphocyte counts may be used as a biomarker for the clinical effects of KW-0761.

In conclusion, 49 patients were enrolled in the KW-0761 phase I trial, in which KW-0761 was safely administered and eTregs in peripheral blood were depleted in most patients. A durable clinical response was observed in some patients, and high lymphocyte levels before treatment may be a biomarker for the efficacy of the KW-0761 treatment. A phase I clinical trial on preoperative combination therapy of KW-0761 plus an anti-PD-1 treatment in patients with advanced or recurrent solid tumors is currently underway. The synergistic effect of KW-0761 for depleting Tregs and other immunotherapies is expected in the future.

## Supporting information

S1 Fig Clinical courses of patients. Kaplan–Meier curves of OS and PFS for 49 CCR4-negative solid cancer patients were analyzed with doses of KW-0761 (a, b) and clinical responses (c, d). PD, progressive disease; SD, stable disease; PR, partial response.
(TIF)

S2 Fig Characteristics of clinical responses and survival in esophageal cancer patients. (a) A Spaghetti plot for the percentage change in the target lesion tumor burden from baseline over time in esophageal cancer patients (n = 14, left) and non-esophageal cancer patients (n = 35, right). (b-c) Kaplan-Meier curves of OS and PFS for 49 CCR4-negative solid cancer patients were analyzed with or without esophageal cancer. Horizontal dotted lines denote a 30% decrease and a 20% increase. Esophageal cancer (n = 14), red line; non-esophageal cancer (n = 35), blue line.
(TIF)

S3 Fig Effects of Treg depletion in peripheral blood. Longitudinal changes in the percentage of eTregs in CD4^+^ T cells at baseline and post-KW-0761 treatment in patients with blood assessments (n = 37). 0.1 mg/ml (n = 23), blue bar; 0.5 mg/ml (n = 3), green bar; 1.0 mg/ml (n = 23), orange bar. Solid lines indicate data obtained during the KW-0761 treatment and dotted lines indicate data collected after the completion of the KW-0761 treatment.
(TIF)

S4 Fig Longitudinal changes in serological immune responses for NY-ESO-1 and XAGE-1. Antibody responses in the phase Ib trial were analyzed for NY-ESO-1 (a) and XAGE-1 (b) in patients with positive antibody responses at baseline or during the KW-0761 treatment.
(TIF)

S5 Fig Clinical courses of patients based on NY-ESO-1 and XAGE1 antibody responses. Kaplan-Meier curves of OS and PFS were analyzed based on the presence or absence of tumor NY-ESO-1 or XAGE1 antigen expression (a, b), baseline serum NY-ESO-1 or XAGE1 antibody responses (c, d), and increased NY-ESO-1 or XAGE1 antibody responses after the KW-0761 treatment (e, f).
(TIF)

S1 Table Patient characteristics and serological immune response. (DOCX)

S1 Checklist CONSORT 2010 checklist of information to include when reporting a randomised trial*. (DOC)

S1 Text Phase Ia/Ib multicenter physician-initiated clinical trial of Mogamulizumab for patients with advanced or recurrent solid tumors. (DOCX)

S1 File (PDF)
