# Supplementary figures and images for: Integrated analysis of phase 1a and 1b randomized controlled trials; Treg-targeted cancer immunotherapy with the humanized anti-CCR4 antibody, KW-0761, for advanced solid tumors
Source: PLoS One. 2023 Sep 20;18(9):e0291772. doi: 10.1371/journal.pone.0291772 (PMC10511099; doi:10.1371/journal.pone.0291772)

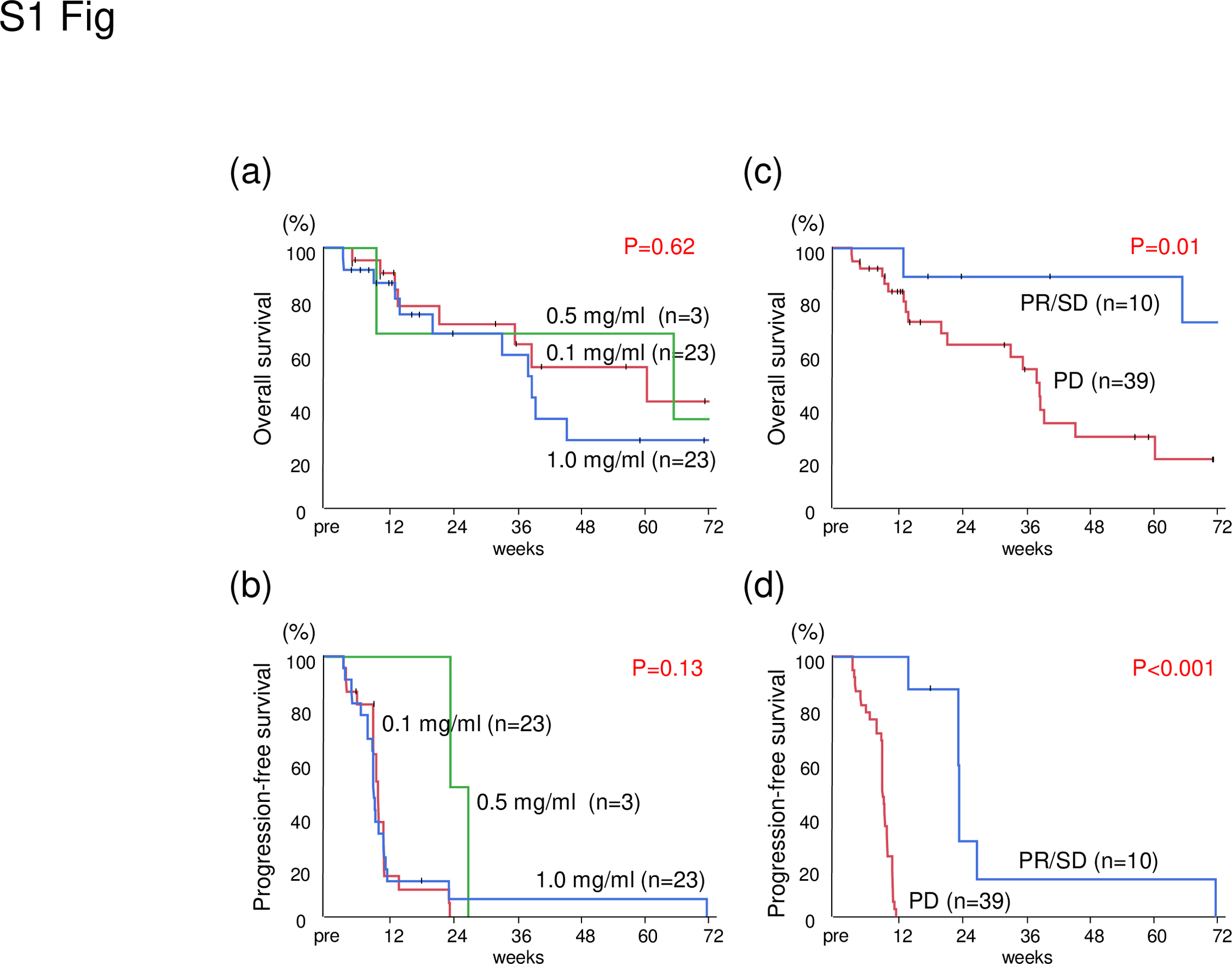

Supplement: S1 Fig — Kaplan–Meier curves of OS and PFS for 49 CCR4-negative solid cancer patients were analyzed with doses of KW-0761 (a, b) and clinical responses (c, d). PD, progressive disease; SD, stable disease; PR, partial response. (TIF) [file pone.0291772.s001.tif]

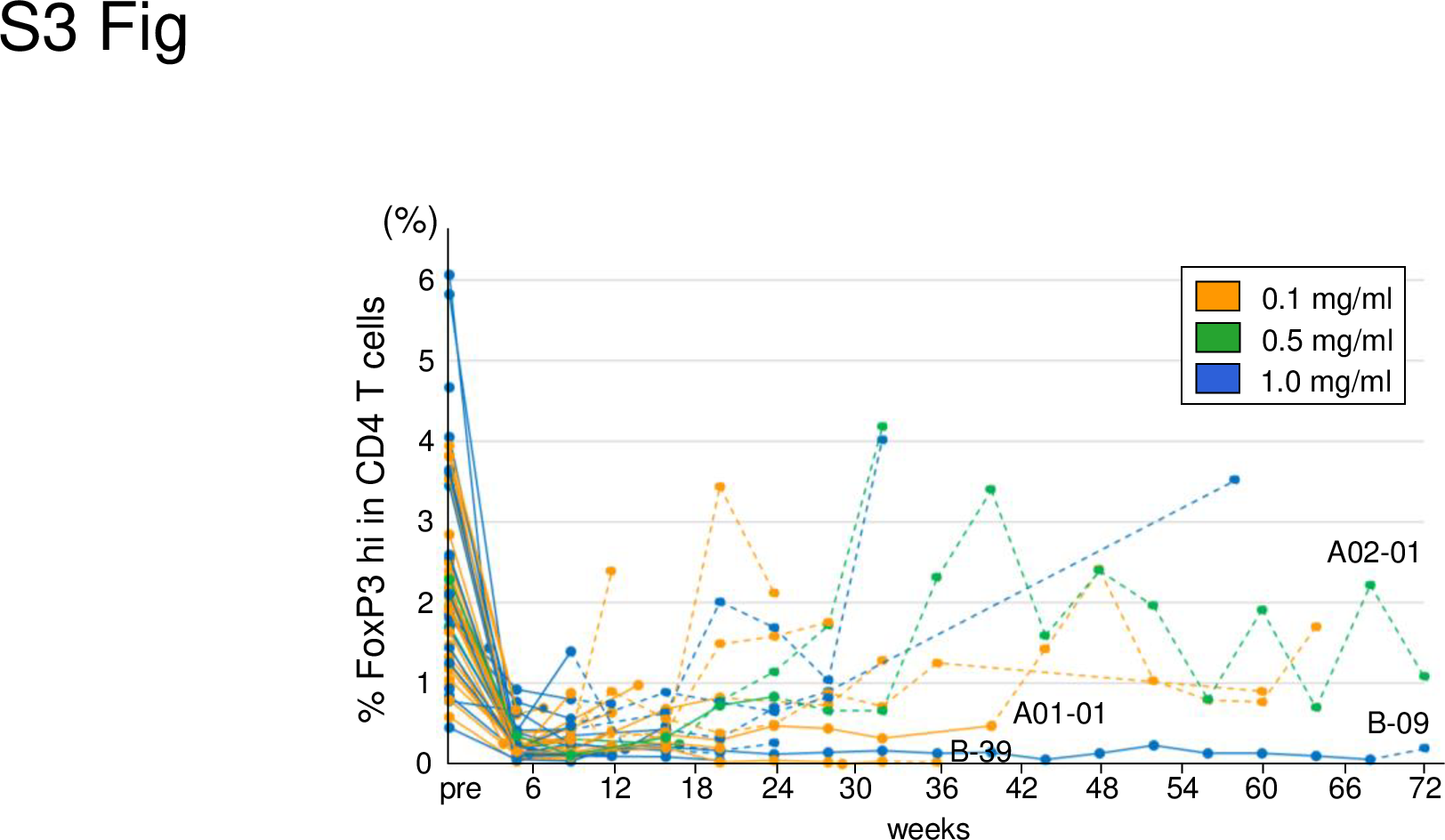

Supplement: S2 Fig — (a) A Spaghetti plot for the percentage change in the target lesion tumor burden from baseline over time in esophageal cancer patients (n = 14, left) and non-esophageal cancer patients (n = 35, right). (b-c) Kaplan-Meier curves of OS and PFS for 49 CCR4-negative solid cancer patients were analyzed with or without esophageal cancer. Horizontal dotted lines denote a 30% decrease and a 20% increase. Esophageal cancer (n = 14), red line; non-esophageal cancer (n = 35), blue line. (TIF) [file pone.0291772.s002.tif]

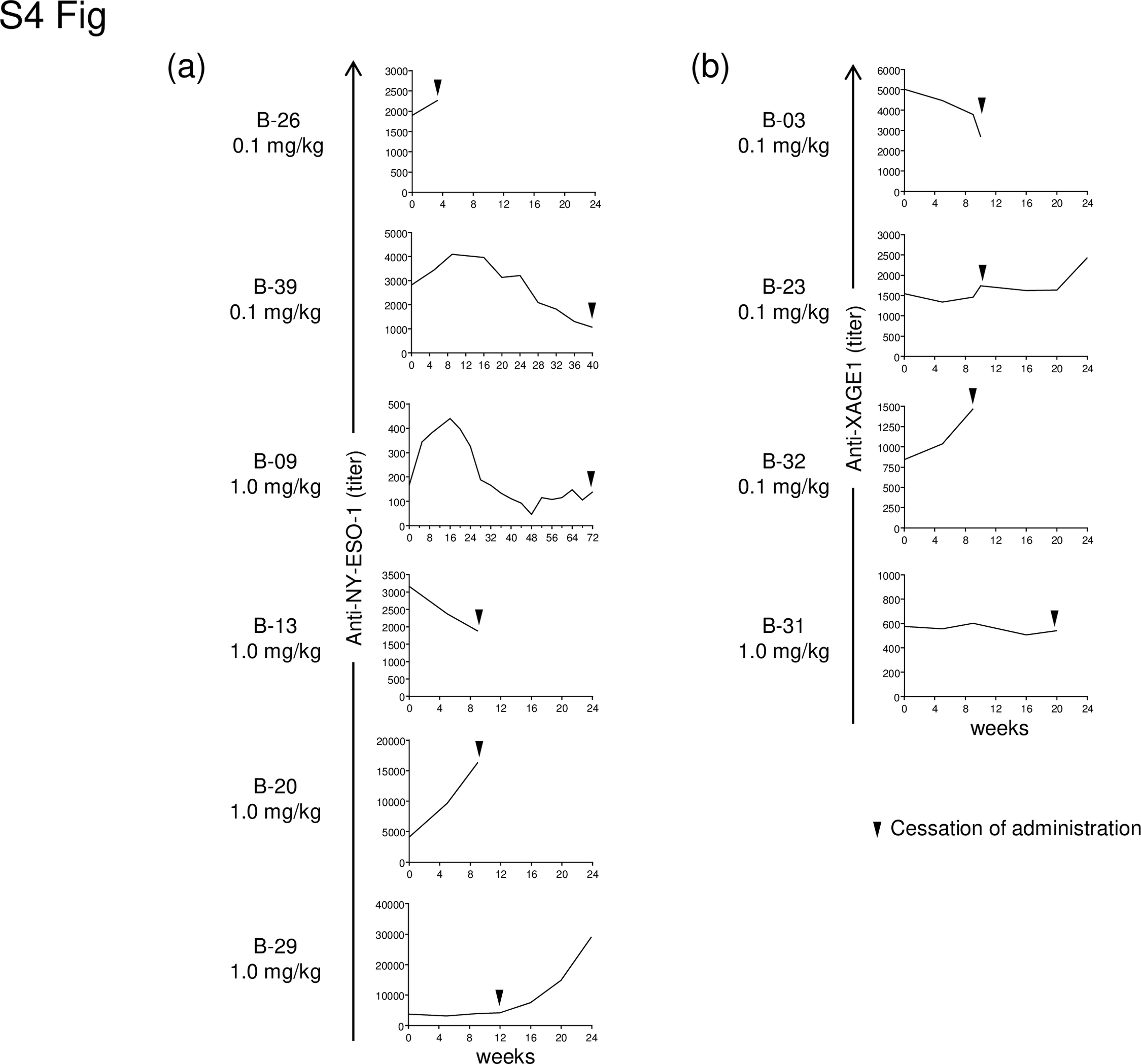

Supplement: S3 Fig — Longitudinal changes in the percentage of eTregs in CD4+ T cells at baseline and post-KW-0761 treatment in patients with blood assessments (n = 37). 0.1 mg/ml (n = 23), blue bar; 0.5 mg/ml (n = 3), green bar; 1.0 mg/ml (n = 23), orange bar. Solid lines indicate data obtained during the KW-0761 treatment and dotted lines indicate data collected after the completion of the KW-0761 treatment. (TIF) [file pone.0291772.s003.tif]

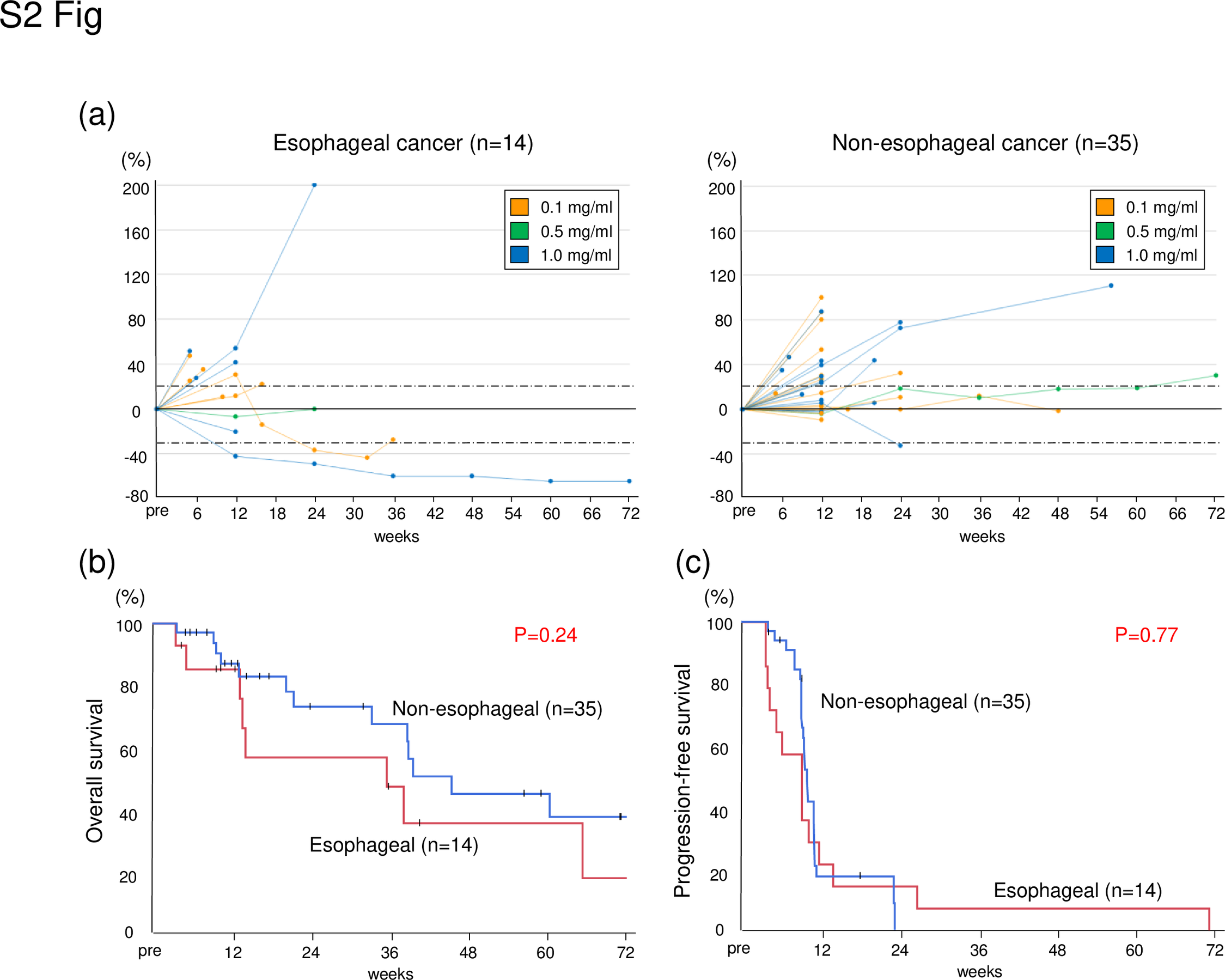

Supplement: S4 Fig — Antibody responses in the phase Ib trial were analyzed for NY-ESO-1 (a) and XAGE-1 (b) in patients with positive antibody responses at baseline or during the KW-0761 treatment. (TIF) [file pone.0291772.s004.tif]

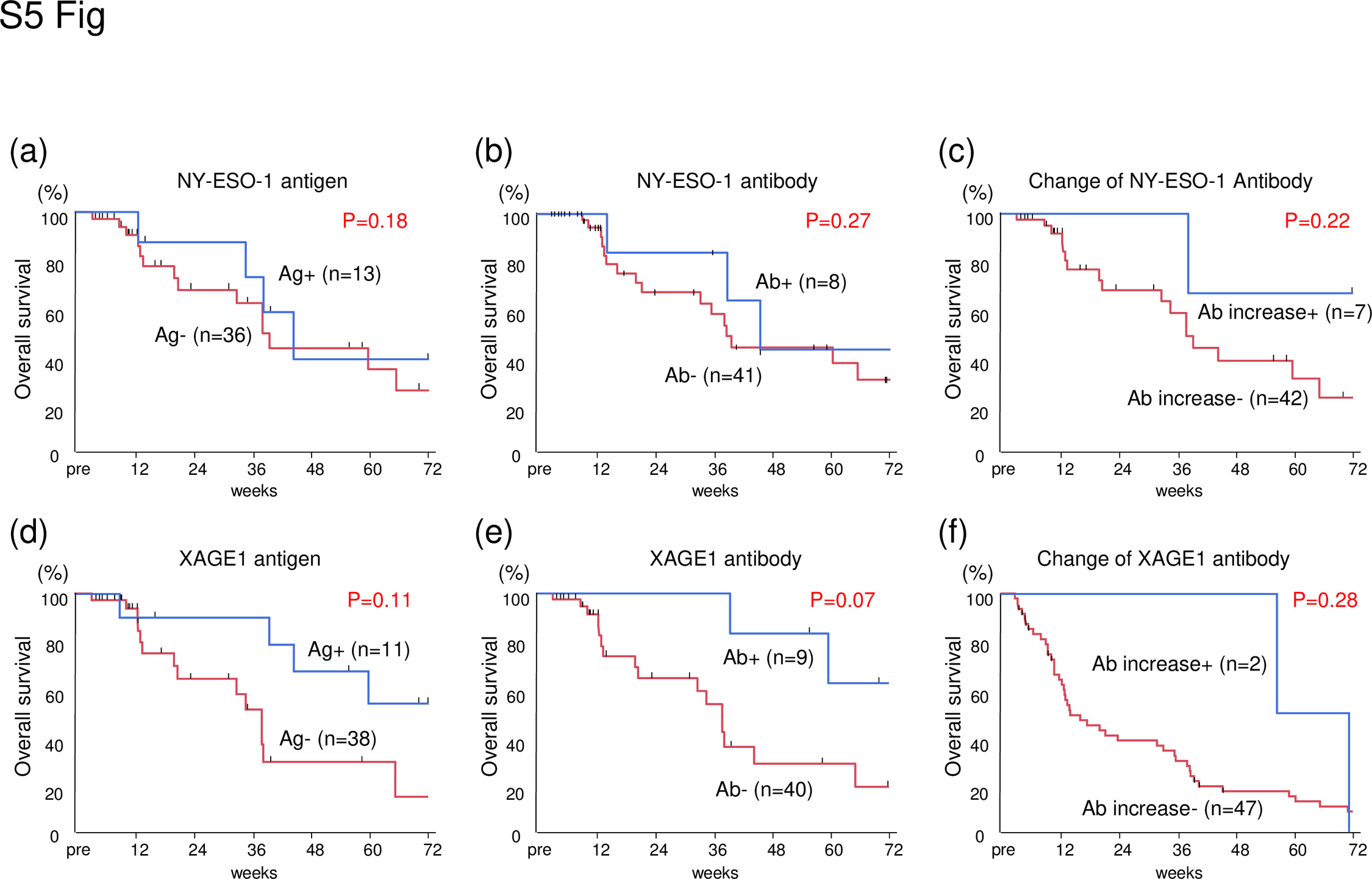

Supplement: S5 Fig — Kaplan-Meier curves of OS and PFS were analyzed based on the presence or absence of tumor NY-ESO-1 or XAGE1 antigen expression (a, b), baseline serum NY-ESO-1 or XAGE1 antibody responses (c, d), and increased NY-ESO-1 or XAGE1 antibody responses after the KW-0761 treatment (e, f). (TIF) [file pone.0291772.s005.tif]
