# Supplementary material for: Integrated analysis of phase 1a and 1b randomized controlled trials; Treg-targeted cancer immunotherapy with the humanized anti-CCR4 antibody, KW-0761, for advanced solid tumors
Source: PLoS One. 2023 Sep 20;18(9):e0291772. doi: 10.1371/journal.pone.0291772 (PMC10511099; doi:10.1371/journal.pone.0291772)
Supplement: S1 Table — (DOCX) [file pone.0291772.s006.docx]

**S1 Table. Patient characteristics and serological immune response**

| Dose (mg/kg) | ID | Tumor Type | No. of  infusions | Best  overall response | PFS (days) | OS (days) | Treatment-related AEs | | NY-ESO-1 | | | XAGE-1 | | |
| --- | --- | --- | --- | --- | --- | --- | --- | --- | --- | --- | --- | --- | --- | --- |
|  |  |  |  |  |  |  | All | Grade  3-4 | Ag | Ab | | Ag | Ab | |
|  |  |  |  |  |  |  |  |  |  | Baseline | Change^*^ |  | Baseline | change^*^ |
| 0.1 | A1-01 | NSCLC | 14 | SD | 160 | 1024 | + | - | + | + | down | - | - | - |
| 0.1 | A1-02 | NSCLC | 11 | SD | 161 | 538 | + | + | - | - | - | - | - | - |
| 0.1 | A1-03 | NSCLC | 6 | PD | 77 | 100 | + | + | + | + | up | - | - | - |
| 0.1 | B-01 | Esophageal cancer | 6 | PD | 42 | 96 | + | + | - | - | - | - | - | - |
| 0.1 | B-03 | NSCLC | 8 | PD | 63 | 422 | + | - | - | - | - | + | + | down |
| 0.1 | B-05 | Melanoma | 5 | PD | 28 | 73 | + | - | - | - | - | - | - | - |
| 0.1 | B-07 | Melanoma | 8 | PD | 77 | 77 | + | - | + | - | - | - | - | - |
| 0.1 | B-10 | Esophageal cancer | 8 | PD | 63 | 249 | + | + | + | - | - | - | - | - |
| 0.1 | B-11 | Esophageal cancer | 4 | PD | 25 | 35 | + | + | - | - | - | - | - | - |
| 0.1 | B-12 | Melanoma | 8 | PD | 76 | 150 | + | - | - | - | - | - | - | - |
| 0.1 | B-15 | NSCLC | 8 | PD | 67 | 77 | + | - | - | - | - | - | - | - |
| 0.1 | B-19 | NSCLC | 8 | SD | 63 | 92 | + | + | + | - | - | - | - | - |
| 0.1 | B-21 | Melanoma | 8 | PD | 77 | 91 | + | + | - | - | - | - | - | - |
| 0.1 | B-22 | Ovarian cancer | 8 | PD | 67 | 224 | + | + | - | - | - | - | - | - |
| 0.1 | B-23 | NSCLC | 8 | PD | 63 | 498 | + | + | - | - | - | + | + | up |
| 0.1 | B-24 | Gastric cancer | 8 | PD | 70 | 271 | + | - | - | - | - | - | - | - |
| 0.1 | B-26 | Gastric cancer | 2 | PD | 21 | 21 | + | - | + | + | up | - | - | - |
| 0.1 | B-28 | Mesothelioma | 4 | SD | 40 | 40 | - | - | - | - | - | - | - | - |
| 0.1 | B-32 | NSCLC | 8 | PD | 63 | 395 | + | - | - | - | - | + | + | up |
| 0.1 | B-33 | Ovarian cancer | 8 | PD | 69 | 77 | + | - | - | - | - | - | - | - |
| 0.1 | B-35 | Esophageal cancer | 4 | PD | 29 | 31 | + | - | - | - | - | - | - | - |
| 0.1 | B-37 | Esophageal cancer | 9 | SD | 96 | 284 | + | - | + | - | - | - | - | - |
| 0.1 | B-39 | Esophageal cancer | 14 | PD | 70 | 251 | + | + | - | + | up | - | - | - |
| 0.5 | A2-01 | NSCLC | 11 | SD | 161 | 559 | + | - | + | + | up | + | + | stable |
| 0.5 | A2-02 | NSCLC | 7 | PD | 66 | 66 | + | + | - | - | - | - | - | - |
| 0.5 | A2-03 | Esophageal cancer | 9 | SD | 184 | 457 | + | + | - | - | - | - | - | - |
| 1.0 | A3-01 | Esophageal cancer | 8 | PD | 63 | 267 | + | + | - | - | - | - | - | - |
| 1.0 | A3-02 | NSCLC | 7 | PD | 64 | 277 | + | + | - | - | - | + | + | down |
| 1.0 | A3-03 | Esophageal cancer | 3 | PD | 25 | 25 | + | - | - | - | - | - | - | - |
| 1.0 | A3-04 | NSCLC | 8 | PD | 77 | 114 | + | + | - | - | - | + | + | down |
| 1.0 | B-02 | Ovarian cancer | 8 | PD | 66 | 413 | + | - | - | - | - | - | - | - |
| 1.0 | B-04 | Melanoma | 8 | PD | 79 | 142 | - | - | - | - | - | - | - | - |
| 1.0 | B-06 | Melanoma | 8 | PD | 56 | 233 | + | - | - | - | - | - | - | - |
| 1.0 | B-08 | Esophageal cancer | 2 | PD | 27 | 93 | + | - | - | - | - | - | - | - |
| 1.0 | B-09 | Esophageal cancer | 23 | PR | 491 | 511 | + | + | + | - | up | + | - | - |
| 1.0 | B-13 | Ovarian cancer | 8 | PD | 65 | 318 | + | - | + | + | down | + | - | - |
| 1.0 | B-14 | Gastric cancer | 4 | PD | 35 | 35 | - | - | - | - | - | - | - | - |
| 1.0 | B-16 | NSCLC | 3 | PD | 25 | 25 | + | + | + | - | - | + | + | NA |
| 1.0 | B-17 | Gastric cancer | 5 | PD | 47 | 47 | + | - | - | - | - | - | - | - |
| 1.0 | B-18 | NSCLC | 8 | PD | 62 | 65 | + | + | - | - | - | + | - | - |
| 1.0 | B-20 | NSCLC | 8 | PD | 56 | 58 | + | - | - | + | up | - | - | - |
| 1.0 | B-25 | NSCLC | 8 | PD | 63 | 497 | + | - | - | - | - | + | + | - |
| 1.0 | B-27 | Esophageal cancer | 5 | PD | 36 | 99 | + | + | - | - | - | - | - | - |
| 1.0 | B-29 | Gastric cancer | 8 | PD | 70 | 272 | + | - | + | + | up | - | - | - |
| 1.0 | B-30 | Esophageal cancer | 8 | PD | 63 | 68 | + | - | - | - | - | - | - | - |
| 1.0 | B-31 | Mesothelioma | 10 | SD | 124 | 124 | + | + | - | - | - | - | + | down |
| 1.0 | B-34 | Esophageal cancer | 8 | PD | 81 | 88 | + | + | + | - | - | - | - | - |
| 1.0 | B-36 | Mesothelioma | 10 | SD | 160 | 168 | + | - | - | - | - | - | - | - |
| 1.0 | B-38 | Ovarian cancer | 8 | PD | 76 | 84 | + | - | - |  | - | - |  | - |

Abbreviations: NSCLC, non-small cell lung cancer; PR, partial response; SD, stable disease; PD, progression disease; PFS, progression free survival; OS, overall survival; AE, adverse event; Ag, antigen; Ab, antibody; NA, not assessed

*Antibody change after the first 8 injections or at study discontinuation.
