# Supplementary material for: Integrated analysis of phase 1a and 1b randomized controlled trials; Treg-targeted cancer immunotherapy with the humanized anti-CCR4 antibody, KW-0761, for advanced solid tumors
Source: PLoS One. 2023 Sep 20;18(9):e0291772. doi: 10.1371/journal.pone.0291772 (PMC10511099; doi:10.1371/journal.pone.0291772)
Supplement: S1 Text — (DOCX) [file pone.0291772.s008.docx]

**Phase Ia/Ib multicenter physician-initiated clinical trial of Mogamulizumab for patients with advanced or recurrent solid tumors**

**Clinical trial protocol**

Clinical protocol number: KW0761-IIT-01

Version number: edition 6.0

Date of preparation: prepared on 23 February 2015

**Summary of the clinical trial protocol**

**1. Objectives of the clinical trial**

To investigate the safety and pharmacokinetics of Mogamulizumab (anti-CCR4 antibody) when administered repeatedly once weekly to patients with advanced recurrent cancer (Phase Ia), and to investigate the safety and efficacy in removing regulatory T cells when administered repeatedly once weekly (Phase Ib).

**2. The evaluation questions**

**2-1. Primary endpoints**

**2-1-1. Phase Ia part**

1) Safety: maximum tolerated dose (MTD), dose-limiting toxicity (DLT), type, frequency and extent of adverse events

2) Pharmacokinetic studies

**2-1-2. Phase Ib part**

1) Safety: type, frequency and extent of adverse events

2) Efficacy in eliminating regulatory T cells

**2-2. Secondary endpoints**

**2-2-1. Phase Ia part**

1) Efficacy: efficacy in eliminating regulatory T cells

2) Efficacy: tumor shrinkage, progression-free survival (PFS), overall survival (OS)

**2-2-2. Phase Ib part**

1) Efficacy: tumor reduction, progression-free survival (PFS), overall survival (OS)

2) Determination of recommended doses for Phase II trials and beyond

**3. Patients**

Patients with advanced or recurrent solid tumors

**3-1. Patients’ selection criteria**

Patients with advanced or recurrent solid tumors who meet all of the following criteria at the time of enrolment will be eligible.

1) Patients with negative CCR4 expression in tumor cells (diagnosis should be made using a method commercialized in Japan) and a confirmed diagnosis of malignancy such as lung cancer, gastric cancer, esophageal cancer, malignant melanoma or ovarian cancer by histopathological diagnosis.

2) Patients who are refractory or intolerant to standard treatment methods, have no suitable treatment options, or have refused standard treatment.

3) Patients with a performance status (ECOG criteria) of 0, 1 or 2

4) Age at least 20 years on the date of obtaining consent to participate in the clinical trial

5) No severe impairment of major organs (bone marrow, heart, lungs, liver, kidneys, etc.) and clinical laboratory values (within 2 weeks prior to enrolment) meet the following criteria

Neutrophil count: ≥ 1,500/µL

Hemoglobin level: ≥ 8.0 g/dL

Platelet count: 75,000/µL or more

Serum total bilirubin level: ≤ 2.0 mg/dL

AST (GOT), ALT (GPT): not more than the upper limit of the facility standard x 2.5 (not more than the upper limit of the facility standard x 5.0 when judged to be due to hepatic infiltration from the primary disease)

Serum creatinine: 1.5 mg/dL or less

Arterial blood oxygen saturation: 93% or more (room temperature)

ECG: No abnormal findings requiring treatment

Left ejection fraction (by echocardiography): ≥ 50%.

6) The patient agrees to use a condom or other contraceptive method from the time consent is obtained until 24 weeks after the end of study drug administration (excluding post-menopausal women (more than one year after the last menstrual period) or women who have undergone surgical sterilization and men who have undergone surgical sterilization).

7) The patient has given written consent.

8) The patient is able to be hospitalized for the administration of Mogamulizumab from before the first dose until the day after the first dose

9) RECIST ver. 1.1 (New response evaluation criteria in solid tumors: Revised RECIST guideline (version 1.1). 12) The patient must have measurable disease at

10) Expected survival of at least 3 months.

**3-2. Exclusion criteria for patients**

Patients who meet any of the following criteria at the time of enrolment are excluded.

1) HIV antibody-positive cases

2) HCV antibody-positive cases

3) Cases of autoimmune disease

4) Patients reported as HBs antigen positive or HBV-DNA 'detected'* by real-time PCR.

* HBV-DNA testing should be performed if the result of the HBc or HBs antibody test is positive.

5) Patients with a history of serious hypersensitivity to the administration of antibody products

6) Patients with overlapping cancers. However, overlapping cancers are defined as simultaneous overlapping cancers and iatrogenic overlapping cancers with a disease-free period of 5 years or less. Carcinoma in situ (intraepithelial carcinoma) or lesions equivalent to intramucosal carcinoma that are considered curable by local treatment shall not be included in overlapping cancers.

7) More than 4 weeks have not elapsed from the scheduled start date of the study drug administration since the administration of anticancer drugs, continuous systemic administration of corticosteroids, immunosuppressive and immunoenhancing drugs, cytokine therapy, radiotherapy or surgery for the underlying disease.

8) Pregnant, lactating or potentially pregnant patients

9) Patients with active infectious complications

10) Patients with psychiatric disorders or dementia

11) Patients requiring continued treatment with systemic steroids

12) Patients who have undergone transplantation therapy such as hematopoietic stem cell transplantation

13) Patients with central nervous system tumor invasion or with clinical findings that are suspicious for such invasion

14) Patients who received another investigational drug within 4 weeks of case enrolment

15) Patients who have received immunotherapy (e.g., tumor vaccine) against tumors within 12 weeks of case enrolment

16) Other patients considered unsuitable for the conduct of the clinical trial.


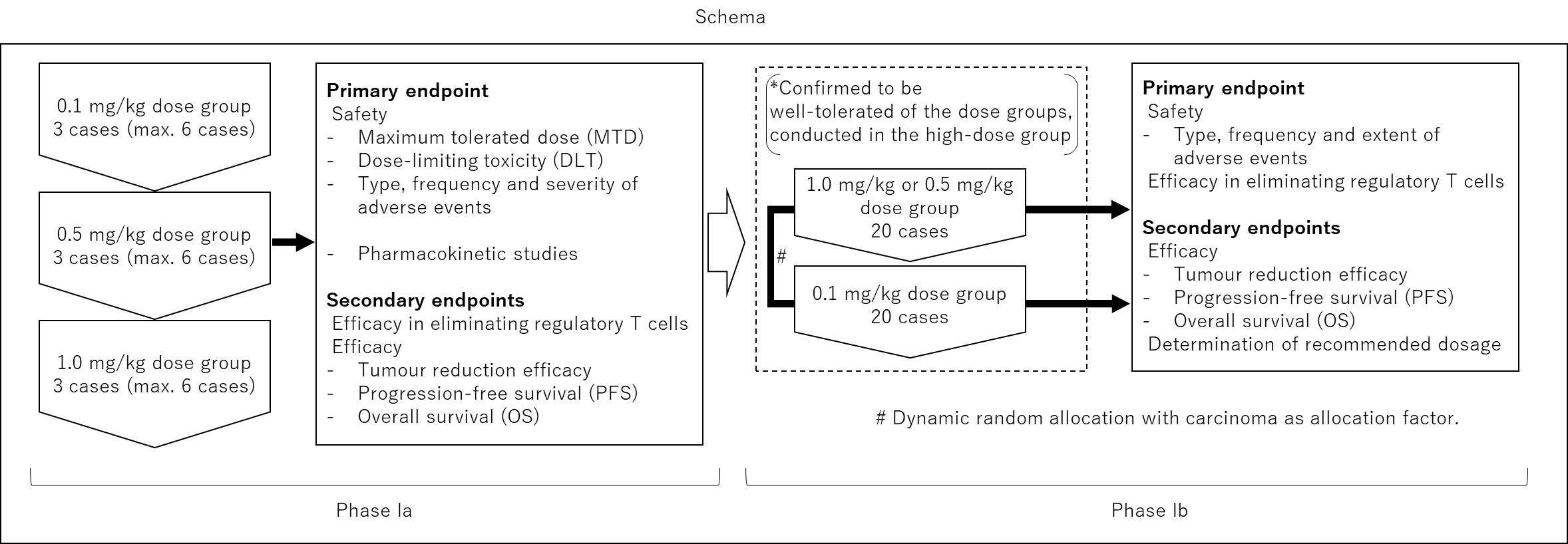
**4. Clinical trial design**

【Phase Ia】 Start at 0.1 mg/kg and dose escalation to 0.5 mg/kg and 1.0 mg/kg if tolerability is confirmed.

【Phase Ib】 Two dose escalation groups (0.1 mg/kg in case 0.5 mg/kg is MTD in phase Ia, 0.1 mg/kg in case 0.5 mg/kg is MTD in phase Ib) and a high-dose group in the tolerated dose group. Enrolment of 20 patients in each group.

【Criteria for tolerability】The dose one step below the MTD is the 'dose that has been found to be well tolerated'. The MTD is the dose at which the number of subjects who develop DLT during the period up to 28 days after the start of treatment with the study drug is 3 or more.

**5. Dosing regimen**

When administering the drug, make sure that the "7-7-1. Dosing criteria" are met and that the "7-7-2. Dosing deferment criteria" and "7-7-3. Dosing discontinuation criteria" are not violated.

【Phase Ia】 Either 0.1 mg/kg, 0.5 mg/kg or 1.0 mg/kg is administered intravenously over 2 hours once a week for a total of eight doses.

【Phase Ib】 Either 0.1 mg/kg or 1.0 mg if tolerated up to 1.0 mg/kg in Phase Ia, either 0.1 mg/kg or 0.5 mg/kg if tolerated up to 0.5 mg/kg, or 0.1 mg/kg intravenously over 2 hours once a week for a total of 8 doses.

【Common to Phase Ia and Phase Ib】

1) Maintenance dose

The ninth and subsequent doses may be administered if the study drug meets the criteria for maintenance dosing described in 7-8, and maintenance dosing should be conducted every 4 weeks. Criteria for Drug Discontinuation".

2) Dosing interval

The dosing interval should not be shortened until eight doses have been administered. After nine doses, dosing may be postponed for up to two weeks and the next dose may be shortened by up to two weeks.

3) Pre-treatment at the time of administration

As reactions to injection are expected in almost all patients, diphenhydramine 30-50 mg and acetaminophen 300-500 mg should be taken internally 30 minutes before administration of the study drug. Hydrocortisone 100 mg is also administered intravenously pre-dose; after the second dose, diphenhydramine 30-50 mg and acetaminophen 300-500 mg are administered pre-dose. Drugs or doses may be changed or increased or decreased as appropriate according to age, symptoms and other factors.

**6. Pre-treatment and concomitant therapy**

Confirm that no prior treatment (e.g., chemotherapy, radiotherapy) has been given for the target disease within 4 weeks prior to the start of the trial. For immunotherapy against tumors (e.g., tumor vaccines), confirm that no immunotherapy has been given within 12 weeks prior to case enrolment. Ensure that exclusion criteria are not violated.

Anticancer drugs, immunosuppressive agents (excluding steroids), immunoenhancing agents, radiotherapy and surgery for the primary disease (including metastasized tumors) are prohibited during the period of study drug administration. Corticosteroids may be administered locally or systemically as pre-treatment for the administration of investigational drugs and for symptomatic improvement of complications and adverse events.

Intravenous infusion of zoledronic acid or pamidronate and subcutaneous injection of denosumab for cases of bone metastases may be administered concomitantly once every four weeks if they were continued prior to participation in the study. However, administration on the same day as the study drug should be avoided.

The following vaccines should be used with caution.

1. Inactivated vaccines [may reduce the efficacy of vaccines (response to vaccination is unknown)].

2. Live or attenuated live vaccines [appropriate measures should be taken if symptoms based on the original disease of the live vaccine inoculated develop (response to vaccination is unknown and secondary infection by live vaccine cannot be ruled out)]. Treatment for other complications and adverse events should be available. Unapproved medicines and alternative therapies (e.g., folk remedies) are prohibited, but the use of food-like supplements is not prohibited.

**7. Trial schedule**

The prescribed observations and tests will be carried out according to the following schedule1. The patient will be hospitalized from before the first administration until the day after the administration.

For subjects in Phase Ia, the investigators will again obtain consent for the continuation of the trial prior to the fifth administration.

For subjects who have demonstrated a regulatory T-cell clearance effect and antigen-specific antibody immunoreactivity or SD or better on RECIST after 8 doses, maintenance administration of the investigational drug is allowed if the subject wishes to receive it and the investigators consider it appropriate to do so.

The date of the first dose of investigational drug is defined as 1 week.

Table 1. Clinical trial schedule


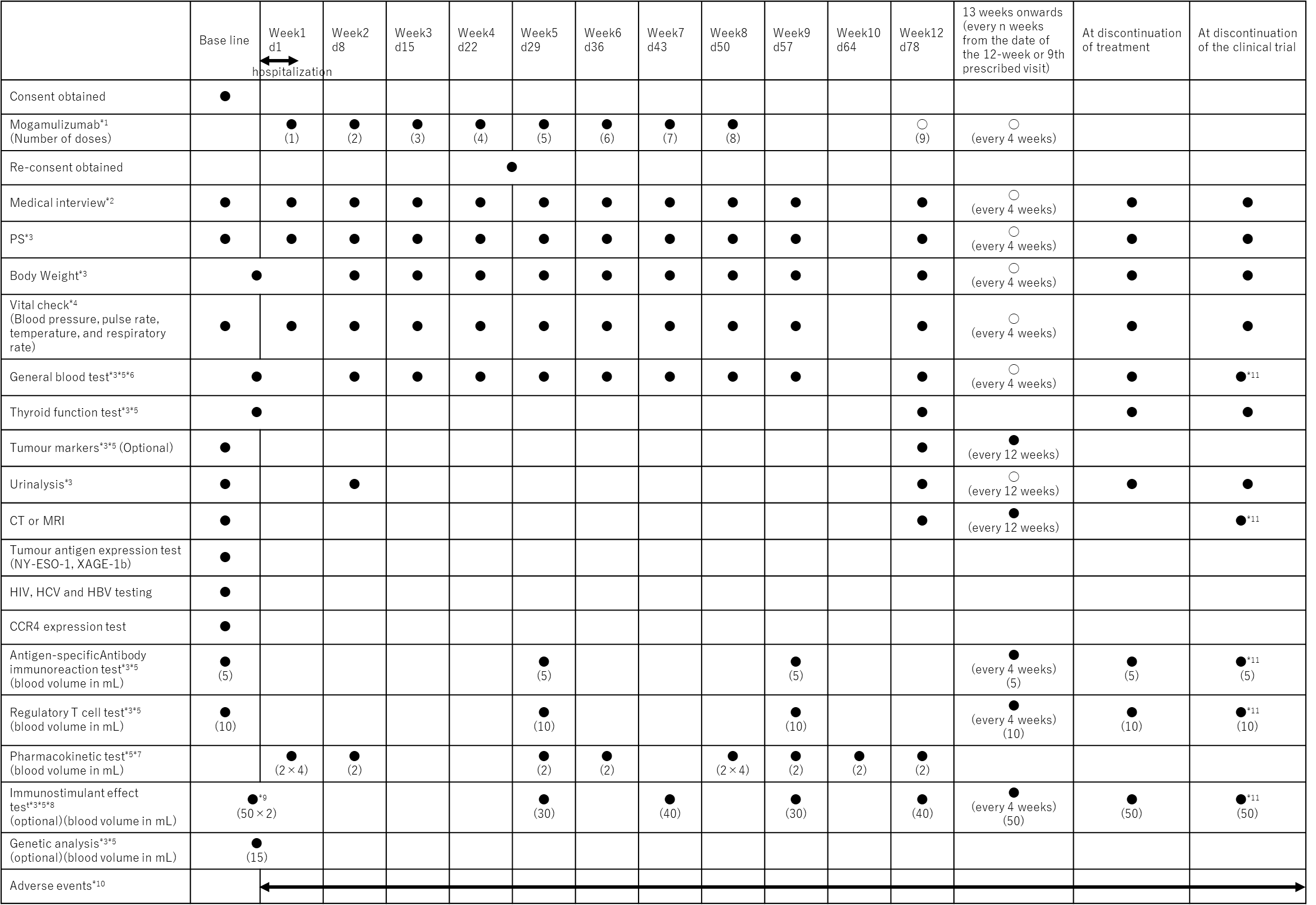


○：Additional items to be performed when maintenance dosing is performed after 8 doses.

*1: The ninth and subsequent doses of the investigational drug should be administered only when SD or better is confirmed in the regulatory T-cell elimination effect and antigen-specific antibody immunoreactivity or RECIST, the subject wishes to receive a maintenance dose of the investigational drug, and the investigator and others consider that the maintenance dose of the investigational drug is appropriate.

*2: Continuous observation from before the start to the end of study drug administration.

*3: To be conducted before administration of the investigational medicinal product during the period of administration of the investigational medicinal product.

*4: Before administration of the investigational drug, at 30 minutes, 1 hour, 1.5 hours after the start of administration, at the end of administration, 30 minutes and 1 hour after the end of administration.

*5: If it is difficult to collect the prescribed volume of blood due to signs of anemia, etc., the following tests should be performed in consideration of subject safety: 1. General blood test (facility prescribed volume), 2. Thyroid function test (facility prescribed volume), 3. Regulatory T cell test (10 mL), 4. Pharmacokinetic test (Phase Ia part only, 2 mL), 5. Antigen-specific antibody immunological reaction test (5mL), 6. Genetic analysis (15mL), 7. Immunopotentiation effect test (30~50mL), 8. Tumor markers (facility's specified volume), as much blood as possible in the order of priority.

*6: Testing is also performed at week 12 after the last dose of the study drug.

*7: The volume of blood drawn per dose should be 2 mL. See Table 2 for the timing of the test.

*8: The test items should be those indicated in (2), (4) to (6) of 8-3-3 Definition of other items.

*9: Blood sampling on multiple days is acceptable.

*10: Adverse events should be observed until 12 weeks after the last dose of the study drug. However, for non-hematological toxicities of Grade 3 and above, the observation period for adverse events is up to 24 weeks.

*11: If no maintenance dose is given after 8 doses and the study is discontinued after 12 weeks of testing, only general blood tests, CT or MRI, antigen-specific antibody immunoreactivity tests, regulatory T-cell tests and immunoenhancing effect tests should be performed when the study is discontinued.


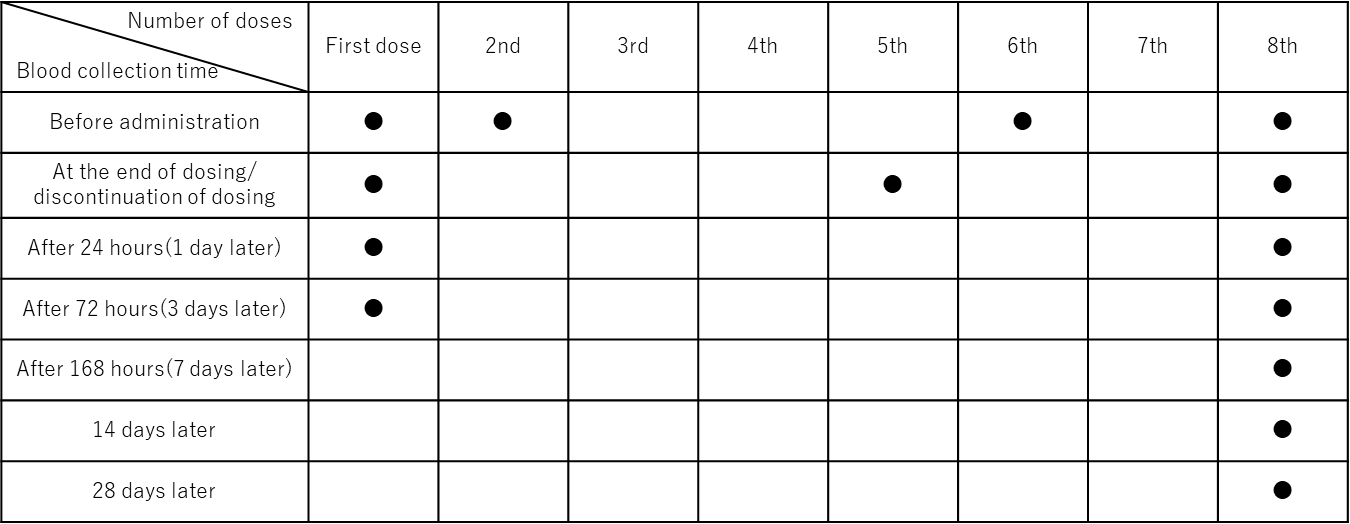
Table 2. Schedule of blood sampling for pharmacokinetic testing (only in phase Ia part)

**8. Discontinuation criteria**

Dosing of the subject shall be discontinued if any of the following criteria are met.

1) When dosing is postponed for more than 2 weeks after the specified scheduled dosing date according to the dosing postponement criteria.

2) When "PD in overall efficacy in RECIST variant evaluation" is achieved after 24 weeks.

3) If an adverse event occurs that makes it difficult for the investigator to continue the study

4) If the subject requests discontinuation of the investigational drug

5) If the subject is found to be ineligible

6) Other cases in which the investigator decides that the trial should be terminated.

**9. Expected number of patients enrolled**

**9-1. Phase Ia part**

0.1 mg/kg group 3 cases (maximum 6 cases)

0.5 mg/kg group 3 cases (maximum 6 cases)

1.0 mg/kg group 3 cases (maximum 6 cases)

Total 9 cases (maximum 18 cases)

**9-2. Phase Ib part**

20 cases if conducted in 1 group

If conducted in 2 groups, 20 cases in each group, 40 cases in total.

**10. Expected duration of the clinical trial**

February 2013 - March 2016

Abbreviation table

| Abbreviation | | | Name |
| --- | --- | --- | --- |
| 5-FU | | | 5-Fluorouracil |
| ADCC | | | Antibody Dependent Cellular Cytotoxicity |
| ADP | | | Adenosine Diphosphate |
| ALP | | | Alkaline Phosphatase |
| ALT | | | Alanine Aminotransferase |
| AST | | | Aspartate Aminotransferase |
| ATL | | | Adult T-cell Leukemia |
| AUC0-7d | | | Area Under the Curve 0-7day |
| CBDCA | | | cis-Diamine(1,1-cyclobutanedicarboxylato)-Platinum(II), Carboplatin |
| CCR4 | | | Chemokine (C-C motif) Receptor 4 |
| CD | | | Cluster of Differentiation |
| CDDP | | | cis-Diamminedichloro-platinum(II), Cisplatin |
| CL | | | Clearance |
| C_max_ | | | Maximum Concentration |
| CPT-11 | | | Camptothecin11, Irinotecan Hydrochloride |
| CR | | | Complete Response |
| C_trough_ | | | Trough Concentration |
| CT | | | Cancer-testis |
| CT | | | Computer Tomography |
| CTCAE | | | Common Terminology Criteria for Adverse Events |
| CTL | | | Cytotoxic T Lymphocyte |
| CTLA4 | | | Cytotoxic T Lymphocyte Antigen 4 |
| DCF | | | Data Clarification Form |
| DLT | | | Dose Limiting Toxicity |
| DTIC | | | Dimethyl TriazenoImidazole Carboxamide |
| ECOG | | | Eastern Cooperative Oncology Group |
| ELISA | | | Enzyme-linked Immune Sorbent Assay |
| FCM | | | Flow Cytometry |
| FT3 | | | Free Triiodothyronine |
| FT4 | | | Free Thyroxine |
| GCP | | | Good Clinical Practice |
| G-CSF | | | Granulocyte Colony Stimulating Factor |
| γ-GTP | | | Gamma-Glutamyl Transpeptidase |
| GMP | | Good Manufacturing Practice | |
| GOT | | Glutamic Oxaloacetic Transaminase | |
| GPT | | Glutamic Pyruvic Transaminase | |
| GVHD | | Graft-Versus-Host Disease | |
| HBc | | Hepatitis B Core | |
| HBs | | Hepatitis B Surface | |
| HBV-DNA | | Hepatitis B Virus Deoxyribonucleotide | |
| hCG | | Human Chorionic Gonadotrophin | |
| HCV | | Hepatitis C Virus | |
| HIV | | Human Immunodeficiency Virus | |
| HLA | | Human Leukocyte Antigen | |
| IFN-γ | | Interferon-gamma | |
| IgG | | Immunoglobulin G | |
| IHC | | Immunohistochemistry | |
| LDH | | Lactase Dehydrogenase | |
| MAGE-A4 | | Melanoma-Associated Antigen 4 | |
| MDSC | | Myeloid-derived Suppressor Cell | |
| MedDRA | | Medical Dictionary for Regulatory Activities | |
| MF | | Mycosis Fungoides | |
| miRNA | | micro-RNA | |
| MRI | | Magnetic Resonance Imaging | |
| MRT | | Mean Residence Time | |
| MTD | | Maximum-tolerated Dose | |
| OS | | Overall Survival | |
| PCR | | Polymerase Chain Reaction | |
| PD | | Progressive Disease | |
| PFS | | Progression Free Survival | |
| PR | | Partial Response | |
| PS | | Performance Status | |
| PTCL | | Peripheral T-cell Lymphoma | |
| PTX | | Paclitaxel | |
| RECIST | | Response Evaluation Criteria in Solid Tumors | |
| SD | | Standard Deviation | |
| SD | | Stable Disease | |
| t_1/2_ | Half Life | | |
| TARC | Thymus and Activation-regulated Chemokine | | |
| TLS | Tumor Lysis Syndrome | | |
| TNF-α | Tumor Necrosis Factor-alpha | | |
| TSH | Thyroid Stimulating Hormone | | |
| Vss | Volume of Distribution at Steady State | | |

**Table of Contents.**
1. Objectives of the clinical trial

2. The evaluation questions

2-1. Primary endpoints

2-1-1. Phase Ia part

2-1-2. Phase Ib part

2-2. Secondary endpoints

2-2-1. Phase Ia part

2-2-2. Phase Ib part

3. Patients

3-1. Patients’ selection criteria

3-2. Exclusion criteria for patients

4. Clinical trial design

5. Dosing regimen

6. Pre-treatment and concomitant therapy

7. Trial schedule

8. Discontinuation criteria

9. Expected number of patients enrolled

9-1. Phase Ia part

9-2. Phase Ib part

10. Expected duration of the clinical trial

1. Development background

1-1. Advanced or recurrent, esophageal, gastric, lung, malignant melanoma and ovarian cancer and their treatment

1-2. Tumor immunity

1-2-1. Anti-tumor effects of immunity

1-2-2. Clinical applications of tumor immunity

1-3. About Mogamulizumab

1-3-1. Overview of non-clinical data

1-3-2. Overview of clinical trial data

1-4. Side effects

1-5. Summary

2. The structure and role of clinical trials

3. Objectives of the clinical trial

4. Subjects

4-1. Inclusion criteria for subjects

4-2. Exclusion criteria for subjects

5. Obtaining consent of subjects

5-1. Consent and other explanatory documents

5-2. Timing, method and content of obtaining consent

5-2-1. Obtaining consent at the start of a clinical trial

5-2-2. Obtaining consent for the fifth and subsequent doses of study drug in Phase Ia part

5-3. Notes on consent

5-4. Revising consent and other explanatory documents

6. Investigational medicines

6-1. Names

6-2. Dosage form and ingredients

6-3. Molecular formula and molecular weight

6-4. Storage methods

6-5. Validity period

6-6. Preparation of investigational medicinal products

6-7. Packaging and labelling of investigational medicinal products

6-8. Provision of investigational medicines

6-9. Management of investigational medicines and return of unused investigational medicines

7. Clinical trial methods

7-1. Clinical trial design

7-2. Group-to-group transition and procedures in phase Ia part

7-3. Transition to Phase Ib part

7-4. Planned duration of the clinical trial

7-5. Method of enrolment and allocation

7-5-1. Procedure for enrolment of subjects

7-5-2. Allocation

7-6. Expected number of cases to be enrolled

7-6-1. Phase Ia part

7-6-2. Phase Ib part

7-7. Dosing methods

7-7-1. Dosing criteria

7-7-2. Dosing deferral criteria

7-7-3. Dose discontinuation criteria

7-8. Maintenance dose implementation criteria

7-9. Pre-treatment and concomitant therapy

7-10. Post-treatment

8. Evaluation items

8-1. Primary endpoints

8-1-1. Phase Ia part

8-1-2. Phase Ib part

8-2. Secondary endpoints

8-2-1. Phase Ia part

8-2-2. Phase Ib part

8-3. Definition of evaluation items

8-3-1. Definition of primary evaluation items

8-3-2. Definition of secondary evaluation items

8-3-3. Other items defined

9. Observation items and methods

9-1. Clinical trial schedule

9-2. Patient background

9-3. Status of investigational drug administration and concomitant treatment

9-4. Efficacy parameters

9-4-1. Antigen-specific antibody immunoreaction tests (NY-ESO-1 and XAGE-1b)

9-4-2. Regulatory T cell tests (FOUR-COLOR FOXP3 test, CD4/CD25/CCR4 test and MLA CD45 gating)

9-4-3. CT and MRI

9-4-4. Survival Surveys and Post-treatment

9-4-5. Tumor markers (optional)

9-4-6. Evaluation of regulatory T cells (optional)

9-4-7. Antigen-specific Antibody Immunoreactivity Testing (for antigens other than NY-ESO-1 and XAGE-1b) (optional)

9-4-8 Antigen-specific cellular immuno-correspondence testing (arbitrary)

9-4-9. Cytokine Productivity Evaluation (optional)

9-4-10. Analysis of immune response cells and control cells (optional)

9-4-11. Immunohistochemistry (optional)

9-4-12. Gene analysis (optional)

9-5. Safety-related Items

9-5-1. PS (Performance Status)

9-5-2. Vital signs (blood pressure, pulse rate, temperature, respiratory rate)

9-5-3. Body weight

9-5-4. General blood tests

9-5-5. Thyroid function test

9-5-6. Urinalysis

9-6. Pharmacokinetics (Phase Ia part)

10. Safety handling

10-1. Definitions

10-1-1. Definition of Clinical Examination Value Abnormalities and Abnormal Changes

10-1-2. Definition of Harmful Events and Side Effects (Harmful Events with a Negative Causal Relationship)

10-1-3. Definition of Harmful Signs of Rehabilitation

10-1-4. Harmful Signs Related to Symptoms and Observations

10-2. Survey Items

10-2-1. Names of Harmful Events

10-2-2. Date of onset (manifestation) and date of worst deterioration

10-2-3. Severity of illness

10-2-4. Severity

10-2-5. Treatment

10-2-6. Outcome

10-2-7. Outcome date

10-2-8. Causal relationship with investigational drug

10-3. Adverse event reporting and response

10-3-1. Serious adverse event reporting procedures

10-3-2. Evaluation and recommendations by the Effectiveness and Safety Assessment Committee

10-3-3. Determination of measures

10-4. Expected side effects

10-5. Action to be taken when adverse effects occur

10-5-1. Reactions associated with injection

10-5-2. Skin disorders

10-5-3. Hepatitis B virus hepatitis

10-5-4. Tumor lysis syndrome

10-5-5. Hematotoxicity and liver dysfunction

10-6. Measures to be taken if pregnancy is confirmed

10-7. Measures in case of overdose

11. Statistical analysis

11-1. Analysis population

11-2. Criteria for handling subjects

11-3. Data handling standards

11-4. Analysis items and methods

12. Conducting genomic and genetic analyses

12-1. Objectives

12-2. Sample handling

12-3. Analysis methods

12-4. Consent for sample provision

12-5. Disclosure of information to subjects

13. Compliance with and deviations, changes and amendments to the protocol

14. Suspension and termination of treatment

14-1. Suspension requirements for individual test subjects and basis for suspension

14-1-1. Test drug delivery and discontinuation criteria for individual test subjects

14-1-2. Basis for discontinuance of experimental drugs for individual subjects

14-1-3. Discontinuation or interruption of treatment in an actual medical institution

14-2. Interruptions and suspensions of part and all tests

14-3. Completion of tests

15. Completion and submission of case report forms

15-1. Submission materials

15-2. Forms and submission deadlines

15-3. How to fill in the form

15-4. Preparation and sending of case report forms

15-5. Changing or amending case report forms

15-6. Issuing and submitting Data Clarification Forms (DCFs)

15-7. Discrepancies between the case report form and original documents such as medical records

15-8. Handling of case report forms and data

16. Matters relating to access to source documents

17. Quality control and quality assurance of clinical trials

18. Ethical considerations

18-1. Rules and regulations to be observed

18-2. Clinical trial review committees

18-3. Informed consent

18-4. Provision of new information

18-5. Ensuring confidentiality and privacy of subjects

19. Preservation of essential documents

19-1. Self-initiated clinical trial provider

19-2. The conducting medical institution

19-3. Principal investigator

20. Monetary payments and insurance

20-1. Payment of money

20-2. Compensation

21. Sources of funding and possible conflicts of interest

22. Attribution of test results and publication of results

22-1. Publication of results

22-2. Preparation of a comprehensive report

22-3. Provision of data

22-4. Secondary use of data

23. References

**1. Development background**

**1-1. Advanced or recurrent, esophageal, gastric, lung, malignant melanoma and ovarian cancer and their treatment**

Esophageal cancer is a cancer with a poor prognosis because it is often advanced at the time of diagnosis. It accounts for 3.5% of all malignant tumor deaths in Japan, with an incidence rate of approximately 10 per 100,000 persons^1)2)^ . The esophagus is surrounded by major organs, making it susceptible to direct invasion, and hematogenous and lymphogenous metastases are also common. Surgery is the only curative treatment, but even in operable cases, the 5-year survival rate is as low as 36%, and 10% in stage IV cases. In recent years, chemotherapy (CDDP, 5-FU, Docetaxel, etc.) and radiation therapy have improved the treatment outcome, but the 3-year survival rate is still around 30%.

Gastric cancer causes the highest number of deaths among gastrointestinal cancers, and the number of patients is very high in international comparisons. Although both the number of deaths and incidence rates have been declining in recent years, it still ranks second or third among both men and women, with more than 100,000 people developing gastric cancer each year and nearly 50,000 dying from the disease^1)^. Surgery is the only curative treatment, but even in operable cases, the 5-year survival rate is about 60%, and in stage IV, it is about 20%. In recent years, the response rate has improved with the use of multidrug therapy including CPT-11, CDDP, and TS-1, but the prognosis remains poor.

Lung cancer is the leading cause of death from malignant tumors in both men and women^1)^ . Both morbidity and mortality rates for lung cancer are higher in men than in women, with men three to four times more likely to die from the disease than women. There is no significant difference in the number of cases and deaths, which is associated with a lower survival rate among lung cancer patients. Lung cancer is classified into two main types: small cell and non-small cell. Small cell carcinoma, which accounts for about 15-20% of lung cancer cases, is a high-grade cancer that grows rapidly and tends to metastasize to the brain, lymph nodes, liver, adrenal glands, bones, and other organs. As treatment options other than surgical resection, radiation therapy and multiple drug administration in combination with platinum drugs have been used. In recent years, the efficacy of taxanes, gemcitabine, and irinotecan has been evaluated, but the prognosis is poor, with a 5-year survival rate of less than 20% even for stage III non-small cell lung cancer.

The mortality rate of malignant melanoma has continued to decline since the end of World War II, and is now about 1 per 100,000, which is rare compared to the United States and Europe^1)^. However, it is a highly malignant tumor, and the 5-year survival rate for unresectable advanced cases is as low as approximately 10%. Various multi-agent chemotherapy regimens have been tried in the past. In recent years, DTIC has been the most frequently used agent for patients with advanced malignant melanoma, but the response rate is unsatisfactory, with a response rate of approximately 20%, complete response rate of 5-10%, and long-term complete response rate of less than 2%.

Ovarian cancer has an incidence of 11.4 per 100,000 population, accounting for about 32% of all gynecologic cancers^1)^. It shows an increase after the age of 50, and the incidence is higher in the elderly. Most cases are treated with aggressive surgical debulking, but surgery alone is not curative, and chemotherapy is administered postoperatively. TC therapy, a combination of CBDCA and PTX, is the standard first-line drug therapy, but the 5-year survival rate is only 49.7%; the 5-year survival rate for stage IV is 12%.

For these and other types of highly advanced or recurrent carcinomas, there is a need to develop new treatments with superior specificity, efficacy, and safety as alternatives to surgery, chemotherapy, and radiation therapy.

**1-2. Tumor immunity**

**1-2-1. Anti-tumor effects of immunity**

Immunity is involved from the developmental stages of tumor development to the elimination of established tumors. Immunological surveillance mechanisms are involved in tumor development. This is true for RAG gene deletion immunodeficient mice, in which most mice, as evidenced by tumor formation in individual mice. In addition, when allogeneic tumors are transplanted between inbred mice, the same tumor that is re-transplanted is rejected in mice in which the viable tumor has been resected. On the other hand, another tumor is viable and grows, demonstrating the specific antitumor effect of immunity even for established tumors. At the same time, the presence of tumor antigens and cytotoxic T lymphocytes (CTL: cytotoxic T lymphocyte), the effector cells of tumor rejection, has been demonstrated^3)^.

**1-2-2. Clinical applications of tumor immunity**

Efficient induction or proliferation of tumor-specific responsive CTLs in the patient body is thought to provide anti-tumor effects clinically. Currently, three major methods are used for efficient induction and proliferation of anti-tumor CTLs.

The first is adoptive immunotherapy, in which CTLs that specifically act on tumor antigens are induced in the body, as in the case of cancer vaccines, or CTLs themselves are infused. Since 2004, we have conducted cancer vaccine clinical trials using NY-ESO-1 protein, NY-ESO-1 long-chain peptide, NY-ESO-1 long-chain complex peptide, and MAGE-A4 protein. Induction and enhancement of antigen-specific humoral and cellular immunity in patients after vaccine administration has been demonstrated^4)^. However, the clinical effect was limited.

The second method is to control molecules that inhibit the function of CTLs. Ipilimumab is an antibody against CTLA4, a molecule that suppresses T-cell function, and its antagonist effect is believed to enhance anti-tumor immunity, especially tumor-specific reactive CTL. In a clinical trial of Ipilimumab treatment, 34% of 140 participating patients with malignant melanoma were reported to have had some clinical response^5)^. NY-ESO-1, a cancer testis antigen with strong immunogenicity, was simultaneously reported as a very important factor involved in anti-tumor immunity during the clinical response to Ipilimumab, with significantly higher clinical response reported in patients with NY-ESO-1 antibody^6)^.

**
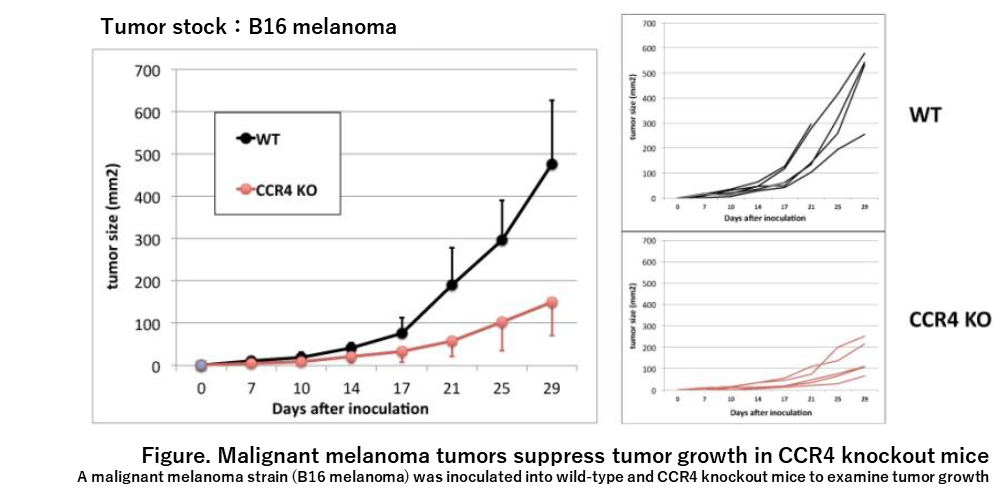
**A third method that has shown promise in recent years is the control of immune cells that suppress the function of CTLs. This type of cell is called regulatory T cells (Treg), a group of CD4-positive and CD25-positive cells that specifically express the transcription factor Foxp3. Regulatory T cells originally regulate immune responses against the self and play an important role in maintaining immune tolerance, but they also suppress anti-tumor immunity, and it has been shown that removal of CD4-positive CD25-positive cells leads to regression of transplanted tumors in mice^7)^. Furthermore, it has recently been shown that tumor growth is suppressed in CCR4 knockout mice due to suppression of regulatory T cells (see figure below); among Foxp3-expressing human regulatory T cells, the fraction that acts to suppress CTLs are activated regulatory T cells, which are found to strongly express CCR4 ^7, 8)^. In the bodies of patients who received the cancer vaccine therapy described above, although antigen-specific immune responses were induced and enhanced, the presence of regulatory T cells may have suppressed the clinical response^9)^.

**1-3. About Mogamulizumab**

KW-0761, a humanized anti-CCR4 monoclonal antibody developed by Kyowa Hakko Kirin, is an antibody with dramatically enhanced ADCC activity through de-fucosylation technology^9)^. In March 2012, it was approved by the Ministry of Health, Labour and Welfare for production and use as a therapeutic agent for adult T-cell leukemia-lymphoma (ATL) that expresses CCR4^10)11)^.

CCR4 is reported to be expressed in approximately 90% of patients with ATL^9)^. This is the world's first antibody drug targeting CCR4. It is the world's first POTELLIGENT® antibody that uses POTELLIGENT® technology to enhance ADCC activity and shows anti-tumor effect through ADCC activity. The response rate when the agent is administered as a single agent to patients with CCR4-positive relapsed/relapsed ATL (acute type, lymphoma type or chronic type with poor prognostic factors*), excluding treatment-resistant patients who did not achieve remission by chemotherapy as the previous treatment, is 50.0% [13/26 patients, complete remission (CR) 30.8%, partial remission (PR) The response rate was 50.0% [13/26 patients, 30.8% complete remission (CR), 19.2% partial remission (PR)]^11)^.［Phase II study in Japan　(ATL/monotherapy)］

＊Patients with either high plasma urea nitrogen (BUN), high lactate dehydrogenase (LDH), or low albumin.

**1-3-1. Overview of non-clinical data**

**1-3-1-1. Pharmacological studies**

Results of in vitro studies conducted to support the efficacy of mogamulizumab showed that the mechanism of action of mogamulizumab is ADCC activity, and that mogamulizumab showed ADCC activity against tumor cells derived from ATL patients as well as tumor cell lines derived from T cells. Mogamulizumab showed ADCC activity not only against T-cell derived tumor cell lines, but also against tumor cells derived from ATL patients, suggesting its efficacy in ATL patients. In an in vivo study in which Mogamulizumab was administered intravenously to crab-eating macaques, a clear decrease in the number of CD4 positive/CCR4 positive cells in peripheral blood lymphocytes was demonstrated in the group receiving ≥ 0.01 mg/kg. Furthermore, in a mouse model of subcutaneously transplanted CCR4-positive human T-cell lymphoma cells, inhibition of tumor growth was observed at a dose of 20 mg/kg, indicating in vivo antitumor activity. These results suggest that mogamulizumab may be effective in the treatment of CCR4-positive ATL.

Secondary pharmacological evaluation evaluated the effect of Mogamulizumab on platelets, which have been reported to express CCR4; Mogamulizumab did not bind to platelets and did not affect platelet aggregation induced by TARC, ADP and collagen. Furthermore, there was no decrease in platelet counts in human whole blood with Mogamulizumab. In addition, there was no direct effect of Mogamulizumab on human platelets; Mogamulizumab did not induce IFN-γ secretion from lymphocytes in 5 of 6 patients; and in the one case where IFN-γ secretion was observed, the secretory effect was weaker than that of the positive control antibody. The one case in which IFN-γ secretion was observed was also weaker than that of the positive control antibody. TNF-α secretion was not observed in all 6 patients. Therefore, the TNF-α and IFN-γ secretory effects of mogamulizumab were lower than those of the positive control antibody, and administration of mogamulizumab to humans was not expected to cause any particular problems.

Safety pharmacology was evaluated during single and repeated dose toxicity studies. In addition to general condition observation, electrophysiological, neurobehavioral, electrocardiogram, heart rate, blood pressure, body temperature, blood biochemical and histopathological tests were performed. No abnormalities were found in any of the measurements, and it was considered that mogamulizumab had no effect on the cardiovascular, respiratory, renal, or central nervous systems.

**1-3-1-2. Toxicity test**

Toxicity was evaluated using crab-eating macaques, in which antigen expression was observed in tissues similar to those of humans. As for acute toxicity, no deaths or changes in general condition were observed at doses up to 100 mg/kg, and the approximate lethal dose was considered to be 100 mg/kg or higher. No effects of the test substance were observed after repeated weekly dosing for up to 13 weeks, and the non-toxic dose was 40 mg/kg/week, the highest dose studied in this study. The AUC0-7d at the non-toxic dose was more than 33 times higher than the AUC0-7d value at 8 doses of 1 mg/kg in the clinical study. In a study on embryo-fetal development, administration of 40 mg/kg/week to pregnant crab-eating macaques showed no general toxicity or reproductive effects in the mother. Furthermore, no effects on embryo-fetal development were observed, although there was some transfer of mogamulizumab to the fetus. In addition, no findings of local irritation were observed in the evaluation of the site of administration within each toxicity study.

Single and repeated administration studies in crab-eating macaques showed the production of anti-Mogamulizumab antibodies. Even in individuals that did produce anti-Mogamulizumab antibodies, there were no significant findings in toxicity studies, and anti-Mogamulizumab antibodies were not considered to pose a safety issue.

The decrease in CCR4-positive T cells observed in the toxicity study was based on the pharmacologic effects of mogamulizumab. In the single-dose study with a 12-month observation period, a recovery trend was observed, but the levels remained low throughout the observation period; in the 4-week repeat-dose study, a recovery trend was observed after approximately a 3-month withdrawal period, but the levels remained low at the last examination. However, no signs of toxicity were observed in either study, and the persistent decrease in CCR4-positive T cells was considered not to be a concern for developing toxicity.

**1-3-1-3. Pharmacokinetics and drug metabolism**

Pharmacokinetics were evaluated based on plasma concentration trends after intravenous administration. The results of single and repeated dose studies showed linearity at doses of 0.5 mg/kg or higher. There were no differences in plasma concentration trends between males and females, suggesting that there are no differences in the pharmacokinetics of mogamulizumab between males and females. Repeated dosing did not alter pharmacokinetic parameters, and repeated dosing was shown to result in a steady state.

The results of distribution studies in crab-eating macaques using [^125^I]KW-0761 showed that the maximum tissue distribution of mogamulizumab, excluding plasma and blood, was 4.86% of the administered dose, suggesting that tissue distribution of mogamulizumab is low. Repeated dosing of pregnant crab-eating macaques resulted in plasma concentrations similar to those in non-pregnant macaques, suggesting no pharmacokinetic changes due to pregnancy. In addition, exposure of the fetus to Mogamulizumab was confirmed, indicating that Mogamulizumab passes through the placenta to the fetus.

Based on the results of the non-clinical studies, no safety concerns were identified.

**1-3-2. Overview of clinical trial data**

**1-3-2-1. Domestic phase I clinical study in patients with CCR4-positive relapsed ATL and Peripheral T-Cell Lymphoma (PTCL)**

In Japan, the Phase I study was conducted as a multicenter, open-label, dose-ranging study.

The subjects were ATL patients who relapsed or relapsed after CCR4-positive response to chemotherapy or PTCL patients including MF, etc. The safety and pharmacokinetics of intravenous administration of 0.01, 0.1, 0.5 or 1.0 mg/kg of mogamulizumab four times at weekly intervals were investigated. Efficacy was also examined as a secondary outcome. The dose at which dose-limiting toxicity (DLT) was observed in 3 subjects was defined as the maximum tolerated dose (MTD), the dose one level below the MTD was defined as the recommended dose (1.0 mg/kg if the MTD was not reached), and the recommended dose was determined by adding 3 subjects to the recommended dose. Three new patients were added to the recommended dose. In addition, subjects who relapsed or relapsed after achieving a response in the Phase I study were allowed to receive a second dose of mogamulizumab.

As no DLT was observed and MTD was not reached during the sequential dose increase from 0.01 mg/kg to 1.0 mg/kg (13 subjects in total), the recommended dose was set at 1.0 mg/kg and 3 more subjects were added (16 subjects in total). One of the three patients added to the recommended dose (1.0 mg/kg) had adverse events corresponding to DLT (neutropenia Grade 4, febrile neutropenia Grade 3, and skin rash Grade 3), but since DLT occurred in only one of six patients at the 1.0 mg/kg dose, the dose of 1.0 mg/kg Mogamulizumab was judged to be tolerable at doses up to 1.0 mg/kg.

As with other antibody drugs, mogamulizumab was associated with cytokine release syndrome/acute infusion reactions such as fever and chills, and decreased blood counts of lymphocytes, neutrophils, and leukocytes in a high frequency, all of which resolved spontaneously or with appropriate treatment such as drugs. Although thrombocytopenia was observed in about half of the subjects, all of them showed Grade 2 or lower decrease (Grade 1: 7 subjects, Grade 2: 1 subject), and all of them showed recovery. Serious adverse events that occurred in this study were herpes zoster, hypoxemia, and skin rash.

The maximum plasma drug concentration (Cmax) and plasma drug concentration from days 0-7 and area under the time curve (AUC0-7 days) at the fourth dose of mogamulizumab showed a proportional increase with increasing dose in the dose range of 0.01 to 1.0 mg/kg. Plasma anti-Mogamulizumab antibody levels were below the lower limit of quantification (<5.0 mg/mL) in all subjects.

The response rate was 31.3% (2 CR + 3 PR/16 patients), and for ATL the response rate was 30.8% (2 CR + 2 PR/13 patients).

One patient who received a repeat dose of mogamulizumab did not meet dosing criteria after two doses (AST elevation Grade 3) and was discontinued. The serious adverse event that occurred after re-dosing was hepatitis B. Efficacy could not be evaluated due to discontinuation.

**1-3-2-2. Phase II clinical study in Japan in patients with CCR4-positive relapsed ATL**

This clinical trial was conducted as a multicenter, open-label study. In this study,

Mogamulizumab 1.0 mg/kg was administered intravenously 8 times at 1-week intervals as Twenty-eight patients were enrolled in the study, of whom 27 received the study drug; 26 were included in the efficacy analysis and 27 in the safety and pharmacokinetic analysis.

The overall best response rate, the primary endpoint, was 50.0% (13/26 patients), with a 95% confidence interval of 29.9-70.1%; the lower limit of the 95% confidence interval (29.9%) was above the threshold response rate of 5%, confirming the efficacy of mogamulizumab. The 50% point of PFS estimated by the Kaplan-Meier method was 158 days, with a minimum value of 12 days and a maximum value of 401 days (censored). The 50% point for overall survival (OS) could not be calculated because the survival rate was greater than 50% at the date of death, but the minimum value ranged from 101 days to a maximum value of 401 days (censored).

No deaths were observed in this study, and although serious or severe adverse events were observed, all were controllable with appropriate treatment. The serious adverse events in which a causal relationship could not be ruled out were Stevens-Johnson syndrome and rash. As in the Phase I study, the major adverse events that occurred in this study were "injection reactions" such as "fever" and "chills," and "blood cell count decrease" such as "lymphocyte count decrease," "white blood cell count decrease," "neutrophil count decrease," and "platelet count decrease," as well as "rash.

The Cmax and Ctrough estimates after 8 doses of Mogamulizumab analyzed in the compartmentalized open model were similar to the mean of the measured values, suggesting that repeated dosing hardly changed the pharmacokinetics of Mogamulizumab.

Plasma anti-Mogamulizumab antibody levels were below the lower limit of quantification (<5.0 mg/mL) in all subjects.

The best overall response was judged to be PR or better after 8 doses of mogamulizumab, but subjects who relapsed or relapsed could be readministered mogamulizumab if they wished and if the physician judged it to be useful. The best overall response at re-dose was PR in this subject. No adverse events occurred for the first time at the time of re-dose, and the most common events were the same as those observed in previous studies: injection reactions and decreased blood cell counts ("decreased lymphocyte counts" and "decreased white blood cell counts"). Plasma anti-Mogamulizumab antibody levels were below the limit of quantification (<5.0 mg/mL) at all time points.

**1-4. Side effects**

The incidence of adverse reactions (including laboratory test abnormalities) in 43 safety evaluation subjects in the domestic clinical study was 100% (43/43 subjects). The most common adverse reactions (≥10.0%) were lymphopenia in 41 patients (95.3%), infusion reaction in 37 patients (86.0%), fever in 34 patients (79.1%), leukopenia in 29 patients (67.4%), neutropenia in 24 patients (55.8%), chills in 24 patients (55.8%), thrombocytopenia in 23 patients (53.5%), rash in 18 patients (41.9%), ALT (GPT) increased in 17 patients (39.5%), AST (GOT) increased in 16 patients (37.2%), LDH increased in 13 patients (30.2%), tachycardia in 12 patients (27.9%), ALP increased in 11 patients (25.6%), hypoxemia in 9 patients (20.9%), hemoglobin decreased in 9 patients (20.9%), and, nausea in 8 patients (18.6%), increased blood pressure in 8 patients (18.6%), hypoalbuminemia in 7 patients (16.3%), pruritus in 6 patients (14.0%), weight gain in 6 patients (14.0%), decreased albumin in 5 patients (11.6%), increased γ-GTP in 5 patients (11.6%), headache in 5 patients (11.6%), and hypertension in 5 patients (11.6%)., etc. Serious adverse reactions reported at the time of application included infusion reaction (86.0%), severe skin disorder, infection (4.7%), fulminant hepatitis caused by hepatitis B virus (frequency unknown), hepatitis (2.3%), tumor disruption syndrome (2.3%), severe hematologic toxicity, and hepatic dysfunction.

**1-5. Summary**

In cancer patients, regulatory T cells are known to suppress anti-tumor immune responses. Since regulatory T cells, especially activated regulatory T cells, express CCR4 on their membrane surface, this investigator-initiated clinical trial was designed to eliminate the activated regulatory T cell fraction by administration of mogamulizumab with the expectation that it would produce a clinical response.

**2. The structure and role of clinical trials**

See Appendix 1, "Clinical Trial Implementation Structure". 3.

**3. Objectives of the clinical trial**

The objective of the study is to investigate the safety and pharmacokinetics of Mogamulizumab (anti-CCR4 antibody) when administered repeatedly once weekly to patients with advanced recurrent cancer (Phase Ia), and to investigate the safety and efficacy in removing regulatory T cells when administered repeatedly once weekly (Phase Ib).

**4. Patients**

Patients with advanced or recurrent solid tumors

**4-1. Selection criteria for patients**

Patients with advanced or recurrent solid tumors who meet all of the following criteria at the time of enrollment will be eligible.

1) Patients whose tumor cells are negative for CCR4 expression (diagnosis should be made using a method commercialized in Japan) and whose histopathological diagnosis of malignancy such as lung cancer, gastric cancer, esophageal cancer, malignant melanoma, or ovarian cancer is confirmed.

2) Patients who are refractory or intolerant to standard treatment, have no appropriate treatment, or have refused standard treatment.

3) Patients with a performance status (ECOG criteria) of 0, 1, or 2

4) Patients must be at least 20 years of age on the date of obtaining consent to participate in the clinical trial.

5) No severe damage to major organs (bone marrow, heart, lungs, liver, kidney, etc.) and laboratory values (within 2 weeks prior to enrollment) that meet the following criteria

Neutrophil count: 1,500/μL or more

Hemoglobin level: 8.0 g/dL or higher

Platelet count: 75,000/μL or higher

Serum total bilirubin level: 2.0 mg/dL or less

AST (GOT), ALT (GPT): not more than 2.5 times the upper limit of the facility standard (not more than 5.0 times the upper limit of the facility standard if the cause is judged to be hepatic infiltration due to the primary disease)

Serum creatinine: 1.5 mg/dL or less

Arterial blood oxygen saturation: 93% or higher (room temperature)

Electrocardiogram: No abnormal findings requiring treatment

Left heart ejection fraction (by echocardiography): 50% or more

6) The patient must agree to use a condom or other contraceptive method from the time of consent until 24 weeks after completion of study drug administration (excluding postmenopausal women (more than 1 year after the last menstrual period) or women who have undergone surgical sterilization and men who have undergone surgical sterilization)

7) The patient has given written consent.

8) Patients who are able to be hospitalized for the administration of Mogamulizumab from before the first dose until the day after the first dose.

9) Patients with measurable lesions in RECIST ver. 1.1 (New response evaluation criteria in solid tumours:Revised RECIST guideline (version 1.1)^12)^. Patients must have measurable disease at

10) Expected survival of at least 3 months.

<Rationale for setting>

1) To confirm the clinical efficacy of the drug in eliminating regulatory T cells, and to confirm that the patients have pathologically eligible tumors.

2) To select patients for whom there are no medical or ethical problems in administering the investigational drug.

3) The selection criteria were set to select patients with general conditions that would not interfere with participation in the clinical trial.

4) The age was set as the age at which consent can be obtained from the patient himself/herself.

5) The age was set to select patients for whom safety can be ensured.

6) The safety of the investigational drug for the fetus has not been established.

7) The selection was made in order to select patients whose consent was appropriately obtained.

8) Established to ensure the safety of subjects in the event of an injection-related reaction.

9) To select patients for whom the efficacy of the investigational drug can be evaluated.

10) The selection was made to evaluate the safety and efficacy of repeated administration of the investigational drug, and to select patients who are expected to survive for at least 3 months or longer.

**4-2. Exclusion criteria for patients**

Patients who meet any of the following criteria at the time of enrollment will be excluded.

1) HIV antibody-positive cases

2) HCV antibody positive

3) Patients with autoimmune diseases

4) Patients who are reported to be HBs antigen positive or HBV-DNA "detected" by real-time PCR

5) Patients with a history of serious hypersensitivity to the administration of antibody products

6) Patients with multiple cancers. However, overlapping cancers are defined as simultaneous overlapping cancers and iatrogenic overlapping cancers with a disease-free period of 5 years or less. Carcinoma in situ (intraepithelial carcinoma) or intramucosal carcinoma that is considered curable by local treatment is not included in overlapping cancers.

7) More than 4 weeks have not elapsed from the scheduled start date of the study drug administration since the administration of anticancer agents, continuous systemic administration of corticosteroids, immunosuppressive or immunoenhancing agents, cytokine therapy, radiation therapy, or surgery for the primary disease.

8) Pregnant, lactating, or possibly pregnant patients

9) Patients with active infectious diseases

10) Patients with psychiatric disorders or dementia

11) Patients requiring continued treatment with systemic steroids

12) Patients undergoing transplantation therapy such as hematopoietic stem cell transplantation

13) Patients with central nervous system tumor invasion or with clinical findings that suggest such invasion

14) Patients who received other investigational drugs within 4 weeks of case enrollment

15) Patients who have received immunotherapy (e.g., tumor vaccine) within 12 weeks of case enrollment

16) Other patients who are considered to be inappropriate for the clinical trial

<Rationale for setting>

1), 2), 4) The above values were determined in consideration of safety and the safety of those who handle blood and other specimens.

3) The investigational drug may cause exacerbation of autoimmune diseases.

5), 8), and 9) The safety of the study subjects could not be ensured.

6) The dose was set because of the possibility of affecting safety and life prognosis.

7) The efficacy evaluation may be affected.

10) The reason for exclusion of patients who have difficulty in obtaining appropriate consent.

11) The inclusion criteria were established because of the possibility of influencing the evaluation of the investigational drug.

12) The inclusion criteria were established due to the possibility that graft-versus-host disease (GVHD) may occur as a result of the administration of the investigational drug.

13) The reason for setting this criterion is that there is a possibility that it may affect the evaluation of safety.

14) Established due to the possibility of affecting safety assurance and the evaluation of the investigational drug.

15) Set because of the possibility of affecting safety assurance and the evaluation of the investigational drug.

**5. Obtaining consent of subjects**

**5-1. Consent and other explanatory documents**

The investigator who conducts the clinical trial shall prepare consent documents and other explanatory documents to obtain consent for participation in the clinical trial from subjects, and shall revise them when necessary. Such documents prepared or revised must be approved in advance by the head of the site based on the approval of the Clinical Trial Review Committee.

**5-2. Timing, method and content of obtaining consent**

**5-2-1. Obtaining consent at the start of a clinical trial**

Prior to case enrollment, the investigator or sub investigator (hereinafter referred to as "investigator, etc.") must hand the subject an explanatory document (approved in advance by the investigational review committee of each site) that describes the following information, explain it sufficiently, and confirm that the subject fully understands its contents. After confirming that the subject fully understands the contents, the subject's free and voluntary consent to participate in the study will be obtained in writing. The investigator who provided the explanation and the subject himself/herself shall write their names, affix their seals, or sign and date the consent form.

If a collaborator provides supplementary explanations, he/she should also write his/her name, seal, or signature on the consent form and date it. Unless the site has special arrangements, one copy of the triple-copy consent form should be handed to the subject, one copy should be submitted to the site's clinical trial office or other department designated by the site, and the remaining copy should be kept with the medical record.

1) The clinical trial must involve research

2) Purpose of the clinical trial

3) Name, title and contact information of the investigator(s)

4) Methods of the clinical trial (investigational aspects of the clinical trial, criteria for selection of subjects)

5) Anticipated clinical benefits and risks or inconveniences to the subject's physical and mental health from the investigational drug

6) Availability of other treatment options and the anticipated significant benefits and risks associated with such treatment options

7) The expected duration of the subject's participation in the clinical trial

8) That participation in the clinical trial is of the subject's own free will and that the subject may refuse or withdraw from participation in the clinical trial at any time. Further, the subject shall not lose any benefits to which he/she is entitled due to such refusal or withdrawal.

9) Monitors, auditors, the Clinical Trial Review Committee, and regulatory authorities shall have access to the source documents. In such cases, the confidentiality of the subject shall be maintained. In addition, the subject's name, seal, or signature on the consent document shall be required to authorize access.

10) Subjects' confidentiality shall be maintained even if the results of the clinical trial are made public.

11) A consultation service at the site where subjects should inquire about or contact if they want further information about the clinical trial and their rights or if they have any health problems related to the clinical trial.

12) Treatment to which subjects may be entitled in the event of a clinical trial-related adverse health event

13) Information that may influence the subject's decision to continue participation in the clinical trial, if available, and that will be promptly communicated to the subject.

14) Conditions or reasons for discontinuation of participation in the clinical trial

15) If subjects are required to bear any costs, the details of such costs

16) The subjects' compliance with the requirements of the study

17) Types of investigational review committees that will investigate and deliberate on the appropriateness of the clinical trial, matters to be investigated and deliberated by each investigational review committee, and other matters related to the investigational review committee for the clinical trial concerned.

18) The fact that the protocols, etc. of the investigational review committee can be confirmed and that the investigational review committee should be asked to confirm the protocols, etc. if they wish to do so. In addition, if the written procedures, etc. of the investigational review committee are published on a website, the address of said website shall be available for public inspection, if not published.

19) Matters pertaining to genetic testing

20) Necessary matters pertaining to the said clinical trial

**5-2-2. Obtaining consent for the fifth and subsequent doses of study drug in phase Ia part**

Prior to the fifth administration of the investigational drug, the investigator shall hand the subject an explanatory document containing the following information, explain it thoroughly, and obtain the subject's free and voluntary additional consent in writing for continued participation in this clinical trial. Other methods of obtaining consent shall be in accordance with "5-2-1. Obtaining Consent at the Start of the Clinical Trial".

1) The administration period to determine the maximum tolerated dose has been completed or is scheduled to be completed.

2) Purpose of the clinical trial

3) Name, title and contact information of the investigator(s)

4) Expected duration of subject's participation in the clinical trial

5) Participation in the clinical trial is of the subject's own free will, and the subject may refuse or withdraw from participation at any time. Further, refusal or withdrawal shall not cause the subject to be treated unfavorably or to lose any benefits to which he/she would be entitled if he/she did not participate in the clinical trial.

6) A consultation service at the site where subjects should inquire about or contact if they want further information about the clinical trial and their rights or if they experience any health problems related to the clinical trial.

7) Information that may influence the subject's decision to continue participation in the clinical trial should be promptly communicated to the subject.

8) Conditions or reasons for discontinuation of participation in the clinical trial.

**5-3. Notes on consent**

1) Before obtaining consent, the investigator should give the prospective subject an opportunity to ask questions and sufficient time to decide whether or not to participate in the clinical trial. At that time, the investigator or a collaborator acting as a supplementary informant shall answer all questions to the satisfaction of the prospective subject.

2) Neither the investigator nor the collaborator shall coerce or exert undue influence on the prospective subject with respect to participation or continued participation in the clinical trial.

3) In principle, the subject himself/herself should write, seal or sign the consent document. However, if the subject is unable to read the consent document and other explanatory documents, but is able to understand their contents orally or by other means of communication, an impartial witness is required at the time of explanation. The subject is given the consent document and other explanatory documents, the contents of which are explained orally or by other means of communication, the subject verbally agrees to participate in the trial, and the witness also signs and dates the consent form after the subject signs, seals, or stamps the consent form and dates it him/herself. The subject must also sign and date the consent form.

4) Explanations must not contain any words or phrases that would waive or imply waiver of the rights of prospective subjects, or that would waive or imply waiver of the legal responsibility of the investigator, sub investigator, collaborator, site, or person conducting the clinical trial. The information must not contain any words or phrases that would exempt or imply exemption from the legal responsibility of the investigators, sub investigators, collaborators, medical institutions, or those conducting the clinical trial themselves. 4) The language should be as non-technical as possible and understandable by the person who is to be the subject (including an impartial witness in cases where the person who is to be the subject is unable to read the explanatory document but is able to understand its content by oral or other means of communication).

5) The investigators plan to conduct genomic/genetic analysis for the evaluation of the investigational drug in this study, and will obtain written consent for the use of the provided samples for the purpose of genomic/genetic analysis after providing sufficient explanation to the prospective subjects in advance. The use of samples for genomic/genetic analysis may be conducted only with subjects who have given their written consent to the use of the samples. The investigator or sub investigator must explain to the prospective subject that the provision of the sample is based on the free will of the prospective subject, and that participation in this clinical trial is not dependent on the provision of the sample. If a subject wishes to withdraw consent after obtaining consent for the use of samples for the purpose of genomic/genetic analysis, a separate written consent withdrawal form must be submitted. If the investigational review committee of the investigational site does not approve the use of the sample, the sample will not be used for the purpose of genomic/genetic analysis.

**5-4. Revising consent and other explanatory documents**

1) When information is obtained that may influence the subject's intention to continue participation in the clinical trial, the investigator shall promptly inform the subject of such information and confirm the subject's intention to continue participation in the clinical trial. The date the information was given to the subject, the content of the information given, and the results of the confirmation shall be recorded in the original medical record such as the medical record.

2) When the investigator finds it necessary to revise the consent document and other explanatory documents (when new important information that may be relevant to the subject's consent is obtained), the investigator shall promptly revise the consent document and other explanatory documents based on such information and obtain approval from the investigational review committee in advance. In addition, the investigators shall promptly explain the revised consent document and other explanatory documents to subjects who are already participating in the clinical trial, and obtain their free will consent in writing for their continued participation in the clinical trial. However, consent is not required for subjects for whom administration of the investigational drug or observation has already been completed at the time of the revision. This does not apply to information regarding the progress of the clinical trial, such as fluctuations in general laboratory test values for individual subjects participating in the clinical trial.

**6. Investigational medicines**

All investigational drugs used in this clinical trial are manufactured, handled, stored, and controlled in compliance with the investigational drug GMP ("Standards for Manufacturing Control and Quality Control of Investigational Drugs (Investigational Drug GMP)" No. 0709002 dated July 9, 2008).

**6-1. Names**

Identification number: KW-0761

Generic name: Mogamulizumab (genetical recombination)

**6-2. Dosage form and ingredients**

The pH is adjusted to 5.0-6.0 by hydrochloric acid or sodium hydroxide. The concentration of mogamulizumab is 4.0 mg/mL, and 5 mL is dispensed in one vial.

**6-3. Molecular formula and molecular weight**

Molecular formula: C6520H10072N1736O2020S42

Molecular weight: approximately 146,443

Chemical structural formula: Glycoprotein consisting of two light chain molecules with 219 amino acids and two heavy chain molecules with 449 amino acids

**6-4. Storage methods**

Stored at 2-8℃ in a light-shielded container

**6-5. Validity period**

3 years from the date of manufacture

**6-6. Preparation of investigational medicinal products**

1) Do not shake the vial. 1) Do not shake the vial and do not agitate it vigorously.

2) When administering the drug, the required amount should be drawn off with a syringe and added to 200mL or 250mL of Japanese Pharmacopoeia saline solution.

3) Only Japanese Pharmacopoeia saline solution should be used for preparation.

4) After addition, mix gently and avoid rapid shaking.

5) Prepare the solution as needed and use it immediately after preparation. Discard any remaining liquid.

6) Do not mix with other drugs. 6-7.

**6-7. Packaging and labelling of investigational medicinal products**

1) Drug packaging

One box should contain 4 vials to make one unit. The box should be sealed by the investigational drug supplier.

2) Labeling

Labeling and packaging shall be described in the "Procedures for Administration of Investigational New Drugs," which will be separately stipulated.

**6-8. Provision of investigational medicines**

Investigational drugs are provided to each site in units of one box.

The investigator who conducts the clinical trial on his/her own shall not receive the investigational drug until the notification of the clinical trial plan to the Minister of Health, Labor and Welfare is accepted.

**6-9. Management of investigational medicines and return of unused investigational medicines**

(1) Storage and management of investigational new drug

The investigational new drug is provided by the investigational new drug provider to the investigator at each site at a predetermined time after the submission of the clinical trial plan. The investigator who conducts the clinical trial himself/herself explains the details of the clinical trial to the investigational drug manager at his/her site and submits the "Protocol for Management of Investigational Drugs" separately stipulated, and then requests storage and management of the investigational drug. The investigational drug manager shall appropriately store and manage the investigational drug, used vials, and outer packaging regardless of whether the investigational drug is used or not during the clinical trial period, and shall prepare an investigational drug management chart to monitor the usage status of the investigational drug. (2) The investigator who conducts the clinical trial shall check the consistency of the contents of the investigational drug control record, remaining drugs and case report forms, and if any inconsistency is found, immediately investigate the cause and make the necessary corrections.

(2) Return of unused investigational new drugs

After the completion of a clinical trial, the investigational drug manager shall return unused investigational drugs, empty boxes, and used vials to the person who conducts the clinical trial. When returning them, the subject's name (initials), medical record ID, and other information pertaining to the subject's privacy should not be legible. If unused investigational new drug, empty boxes or used vials are lost or disposed of, a record should be made of the details and reasons. (3) The sponsor shall submit all unused investigational new drugs, empty boxes and used vials returned by the investigational new drug manager to the secretariat of the TPC. The secretariat of the TIC shall properly dispose of all unused investigational new drugs, empty boxes, and used vials submitted by the investigator.

**7. Clinical trial methods**

**7-1. Clinical trial design**

[Phase Ia] Three patients (maximum 6 patients) in the Mogamulizumab 0.1 mg/kg group, followed by three patients (maximum 6 patients) in the 0.5 mg/kg group if the 0.1 mg/kg group is found to be well tolerated, and three patients (maximum 6 patients) in the 1.0 mg/kg group if the 0.5 mg/kg group is found to be well tolerated, at the prescribed dose. The investigational drug will be administered intravenously once a week for 8 consecutive weeks.

<Rationale for Dosing>

ATL, the current indication for which mogamulizumab is indicated, has various degrees of immunocompromised immunity, whereas patients with solid tumors usually have preserved immunocompetence. Therefore, the immune response induced in patients with solid tumors when regulatory T cells are removed by administration of mogamulizumab is stronger than that in ATL, and an autoimmune response is expected to occur simultaneously with an immune response to the tumor.

Furthermore, while Mogamulizumab administered to patients with ATL would be consumed primarily in response to CCR4-expressing tumors in the blood, Mogamulizumab in patients with solid tumors would be used primarily to remove a small number of regulatory T cells, resulting in a lower concentration of Mogamulizumab in the blood compared to patients with ATL. However, it is likely that in patients with solid tumors, mogamulizumab is primarily used to eliminate a small number of regulatory T cells, and blood levels may be higher than in patients with ATL.

In addition, a Phase I study conducted in patients with ATL showed that the 0.01 mg/kg dose of mogamulizumab was effective in eliminating regulatory T cells, and a 10-fold higher dose (0.1 mg/kg) of mogamulizumab would be effective in eliminating regulatory T cells in this subject population.

For these reasons, the risk of developing autoimmune diseases due to the regulatory T-cell elimination effect may be higher in patients with solid tumors, and therefore, the initial dose for patients with solid tumors should be set at 0.1 mg/kg, which is 1/10 of 1.0 mg/kg, the dose used for patients with ATL, to confirm the tolerability of the dose escalation. The initial dose was set at 0.1 mg/kg, 1/10 of the 1.0 mg/kg dose used in the study.

[Phase Ib] The patient population will be divided into 20 patients in each group, each of whom will receive one dose and 0.1 mg/kg, the highest dose among the doses that were found to be well tolerated in Phase Ia (one step below the MTD or 1.0 mg/kg if the MTD has not been reached). The dose should be administered intravenously once a week for 8 consecutive weeks. However, if the dose of 0.1 mg/kg was the only dose that was found to be tolerated in Phase Ia, a dose of 0.1 mg/kg will be used in Phase Ib for 20 patients in each group.

<Rationale for Dose Setting>

If the safety and regulatory T-cell effects of Mogamulizumab in solid tumors are confirmed by this study, it is assumed that a drug that releases immunosuppression by a mechanism different from that of Mogamulizumab may be used in combination with Mogamulizumab in the future. In such a case, the possibility of autoimmune reactions would be higher than with single-agent administration of Mogamulizumab. Therefore, the low dose group was set at 0.1 mg/kg, the lowest dose established in Phase Ia, in order to obtain data over a wide dose range for confirming regulatory T-cell removal efficacy. The low-dose group was set at 0.1 mg/kg.

In the Phase Ib study, the high-dose group and the 0.1 mg/kg group were used in the Phase Ib study.

For subjects who showed SD or higher in the regulatory T-cell test, antigen-specific antibody immunoreactivity test, or diagnostic imaging after 8 doses, if the subject wished to receive maintenance dosing of the study drug and if the investigators determined that maintenance dosing of the study drug was appropriate, the investigator or other appropriate personnel would administer the study drug to the subject. If the subject wishes to receive maintenance dosing of the investigational drug and the investigator determines that maintenance dosing is appropriate, maintenance dosing of the investigational drug is allowed every 4 weeks after 12 weeks until the discontinuation criteria are met.


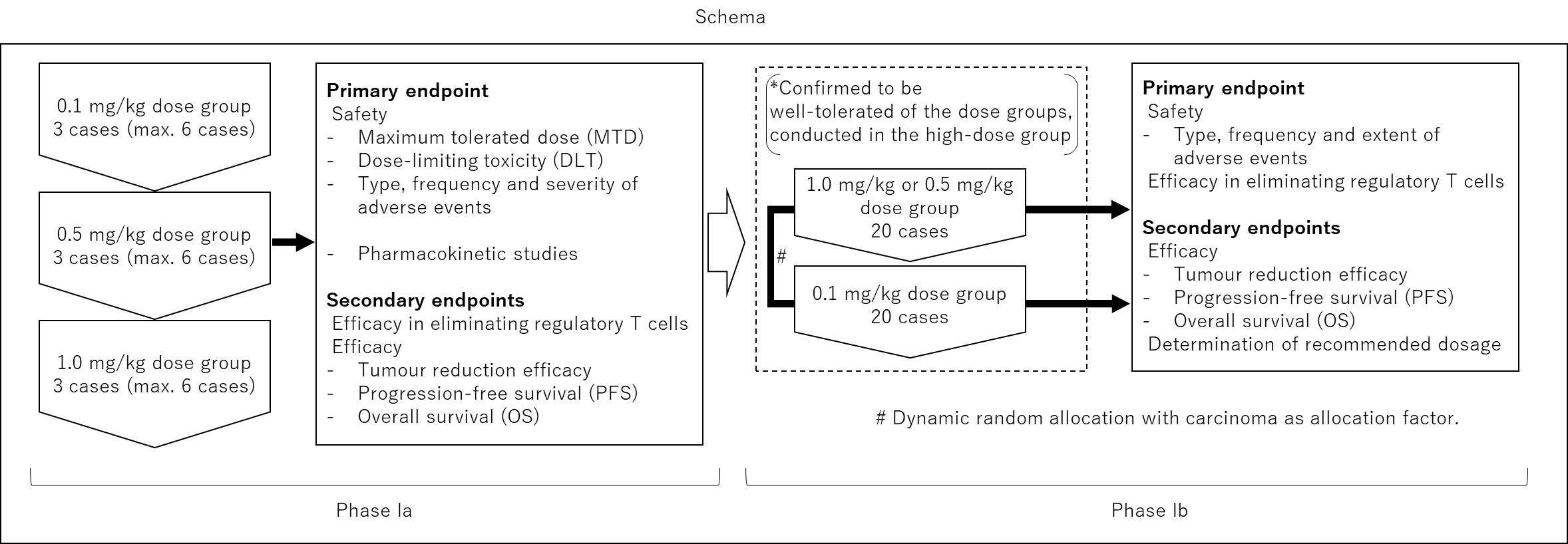
Phase Ia: Starting at 0.1 mg/kg, dose escalation to 0.5 mg/kg and 1.0 mg/kg if tolerability is confirmed.

Phase Ib: Among the treatment groups that were found to be well tolerated, a high-dose group and two 0.1 mg/kg groups (if 0.5 mg/kg was the MTD in Phase Ia, one 0.1 mg/kg group) were administered. Enrollment to be 20 patients in each group.

**7-2. Group-to-group transition and procedures in phase Ia part**

The following procedure will be used to transition from the 0.1 mg/kg dose group to the 0.5 mg/kg dose group and then to the 1.0 mg/kg dose group. The Clinical Trial Coordinating Committee will decide on the transition of dosing groups. However, the efficacy and safety evaluation committee will also evaluate if necessary.

(1) If no DLT occurs in any of the 3 patients in the 0.1 mg/kg dose group, the patient will be shifted to the 0.5 mg/kg dose group.

If one of the three patients in the 0.1 mg/kg group develops DLT, three more patients will be enrolled in the 0.1 mg/kg group; if three or more patients in the 0.1 mg/kg group develop DLT, the MTD will be 0.1 mg/kg and the study will be terminated. If DLT occurs in 2 or less cases, the patient will be shifted to the 0.5 mg/kg group.

(2) If no DLT occurs in any of the 3 patients in the 0.5 mg/kg group, the patient will be moved to the 1.0 mg/kg group.

If 1 of 3 patients in the 0.5 mg/kg group develops DLT, enroll 3 more patients in the 0.5 mg/kg group. If more than 3 patients in the 0.5 mg/kg group develop DLT, the MTD will be 0.5 mg/kg and Phase Ia will be terminated. /Kg group if there are 2 or less cases of DLT.

(3) If none of the 3 patients in the 1.0 mg/kg group develop DLT, the MTD will be greater than 1.0 mg/kg and the Phase Ia portion will be terminated.

If 1 of 3 patients in the 1.0 mg/kg group develops DLT, enroll an additional 3 patients in the 1.0 mg/kg group; if 3 or more patients in the 1.0 mg/kg group develop DLT, the MTD will be 1.0 mg/kg and Phase Ia will be terminated. If DLT develops in 2 or fewer patients, the MTD will be 1.0 mg/kg. If fewer than 2 cases of DLT occur, the MTD will be greater than 1.0 mg/kg and Phase Ia will be terminated.

If the MTD is determined, investigational drug administration in the dose group corresponding to the MTD will be discontinued.

The safety evaluation for transfer to the next group (DLT observation period) can be determined based on information and test results up to 28 days after the start of the investigational drug administration, but the final safety evaluation period will be up to 24 weeks after the last dose in each case.

If more than 2 doses (including 1-week post-dose evaluation) cannot be administered during the DLT observation period (up to 28 days after the start of the investigational drug), except for cases in which dosing is postponed due to DLT, the case will be added to the same dosing group.

**7-3. Transition to phase Ib part**

After completion of the DLT observation period of Phase Ia, Phase Ib should be conducted in the higher tolerated dose group and the 0.1 mg/kg dose group. The Clinical Trial Coordinating Committee will decide whether to proceed to Phase Ib. However, the efficacy and safety evaluation committee will also evaluate if necessary.

**7-4. Planned duration of the clinical trial**

February 2013 - March 2016

**7-5. Method of enrolment and allocation**

**7-5-1. Procedure for enrolment of subjects**

After receiving approval from the Clinical Trial Review Committee and submitting the clinical trial plan, the investigators at each site obtain consent in writing after providing sufficient explanation to the candidate cases, conduct necessary tests to determine eligibility, and request case registration to the Case Registration Center. The case registration center will determine the eligibility of the cases, assign a designated registration number only to subjects who are determined to be eligible, and report the results to the principal investigator, etc. via fax.

**7-5-2. Allocation**

The registration center allocates the prescribed doses to subjects in Phase Ia according to "7-2. In addition, when Phase Ib is conducted with two dose groups, the registration center will dynamically perform random allocation using the minimization method with carcinoma as the allocation factor so that there is no large difference in the number of subjects in the two groups within a carcinoma.

**7-6. Expected number of cases to be enrolled**

**7-6-1. Phase Ia part**

0.1 mg/kg group 3 cases (maximum 6 cases)

0.5 mg/kg group 3 cases (maximum 6 cases)

1.0 mg/kg group 3 cases (max. 6 cases)

Total 9 cases (maximum 18 cases)

＜Rationale for setting the dose>

The dose was set up with reference to the usual Fibonacci's modification method in order to confirm safety up to 1.0 mg/kg, the dose for patients with ATL, while confirming that the dose limiting toxicity (DLT) does not exceed 2/6 (33.3%).

**7-6-2. Phase Ib part**

20 cases if performed in 1 group

In case of 2 groups, 20 cases in each group, 40 cases in total

＜Rationale for setting the dose>

In order to determine the recommended dose for Phase II and beyond, 20 cases per group was set based on the number of cases in which the effect of regulatory T-cell removal can be studied and the feasibility of case enrollment.

**7-7. Dosing methods**

When administering the drug, ensure that the drug meets the "7-7-1. Administration Criteria" and does not conflict with the "7-7-2. Postponement of Administration Criteria" and "7-7-3. Criteria for discontinuation of administration”.

The investigational drug must be started within 2 weeks of case enrollment.

[Phase Ia] Either 0.1 mg/kg, 0.5 mg/kg or 1.0 mg/kg dose is administered intravenously over 2 hours once a week for a total of 8 doses.

[Phase Ib] If tolerability up to 1.0 mg/kg was confirmed in Phase Ia, 0.1 mg/kg or 1.0 mg/kg18

or 0.1 mg/kg or 0.5 mg/kg if tolerability up to 0.5 mg/kg is confirmed, or 0.1 mg/kg if tolerability up to 0.1 mg/kg is confirmed, administered intravenously over 2 hours once a week for a total of 8 doses.

[Common to both Phase Ia and Phase Ib]

1) Maintenance dose

The ninth and subsequent doses of the investigational drug will be administered every 4 weeks as maintenance doses, provided that "7-8. Criteria for Drug Discontinuation" are not violated.

2) Dosing Interval

The dosing interval should not be shortened until 8 doses have been administered. After the 9th dose, dosing may be postponed for up to 2 weeks, and the next dose may be shortened by up to 2 weeks.

3) Pre-treatment at the time of administration

Since reactions associated with injection are expected to occur in almost all patients, 30 to 50 mg of diphenhydramine and 300 to 500 mg of acetaminophen should be taken 30 minutes prior to administration of the study drug. In addition, 100 mg of hydrocortisone should be administered intravenously before the second dose of diphenhydramine 30-50 mg and 300-500 mg of acetaminophen after the first dose. Dosage may be adjusted according to age and symptoms.

**7-7-1. Dosing criteria**

Patients must have had no more than Grade 3 adverse hematologic events^*1^ and no more than Grade 2 adverse non-hematologic events^*2^ prior to administration on the day of administration^*3^.

*1 "Febrile neutropenia" should not have occurred. Lymphocyte count decreased" and "CD4 lymphocyte decreased" are also excluded.

*2 Laboratory tests should be related to AST (GOT), ALT (GPT), ALP, total bilirubin, creatinine, and potassium only.

*3 If a clinical examination cannot be performed prior to the day of administration, a clinical examination performed within 3 days prior to administration may be substituted (or the most recent clinical examination if performed more than once during this period). Symptoms and findings must be confirmed prior to administration on the day of administration.

**7-7-2. Dosing deferral criteria**

Dosing of the investigational drug will be postponed if any of the following conditions occur on the scheduled dosing date^*1^. The period of postponement shall be a maximum of 2 weeks from the scheduled administration date.

1) Grade 4 or higher hematological adverse events. However, "febrile neutropenia" must be Grade 3 or higher.

2) Nonhematologic adverse events of Grade 3 or higher (excluding skin disorders). However, regarding laboratory tests, only those related to AST (GOT), ALT (GPT), ALP, total bilirubin, creatinine, and potassium should be considered.

3) In the case of ongoing skin disorders

4) If the subject requests a postponement of administration

5) Other cases in which the investigator determines that administration should be postponed

*1 Including within 3 days of the scheduled administration date as specified in the regulations.

**7-7-3. Dose discontinuation criteria**

Dosing of the subject will be discontinued if any of the following criteria are met

1) When dosing is postponed for more than 2 weeks from the scheduled dosing date according to the criteria for dose postponement.

2) When "PD" is observed in the overall efficacy in the evaluation of RECIST variant after Week 24.

3) When a skin disorder of Grade 3 or higher is observed

4) Adverse events that make it difficult for the investigator to continue the study

5) The subject requests discontinuation of the investigational drug

6) The subject is found to be ineligible

7) Other cases in which the investigator determines that the study should be terminated.

**7-8. Maintenance dose implementation criteria**

If all of the following criteria are met, maintenance dosing of the investigational drug to the subject concerned will be permitted until "7-7-3.

1) Maintenance dosing can be started within 6 weeks after the 8 doses.

2) If the subject shows an SD or greater in regulatory T-cell elimination and antigen-specific antibody immunoreactivity or RECIST after treatment with the investigational drug.

3) The subject wishes to receive maintenance dosing of the investigational drug

4) The investigator determines that maintenance administration of the investigational drug is appropriate.

**7-9. Pre-treatment and concomitant therapy**

Confirm that no prior therapy (chemotherapy, radiotherapy, etc.) has been given for the subject's disease within 4 weeks prior to the start of the study. Immunotherapy (tumor vaccine, etc.) must not have been administered within 12 weeks prior to case enrollment. Confirm that there are no exclusion criteria.

Anticancer agents, immunosuppressive agents (excluding steroids), immunoenhancing agents, radiation therapy, and surgery for the primary disease (including metastasized tumors) are prohibited during the period of study drug administration. Corticosteroids may be administered locally or systemically as pretreatment for administration of the investigational drug or for symptomatic improvement of complications and adverse events.

Intravenous infusion of zoledronic acid or pamidronate and subcutaneous injection of denosumab for bone metastases are allowed if they have been administered once every 4 weeks if they were continued prior to participation in this study. However, they should not be administered on the same day as the investigational drug.

The following vaccines should be administered concomitantly.

1. Inactivated vaccines [may attenuate the effect of the vaccine (response to vaccination is unknown)].

2. Live or attenuated live vaccines [appropriate measures should be taken if symptoms based on the original disease of the live vaccine inoculated develop (response to vaccination is unknown, and secondary infection by live vaccine cannot be ruled out)]. Treatment for other complications and adverse events should be available. Unapproved drugs and alternative therapies (e.g., folk remedies) are prohibited, but the use of food-like supplements is not prohibited.

**7-10. Post-treatment**

There are no restrictions on post-treatment after discontinuation of treatment.

**8. Evaluation items**

**8-1. Primary endpoints**

**8-1-1. Phase Ia part**

1) Safety: maximum tolerated dose (MTD), dose-limiting toxicities (DLT), and type, frequency, and severity of adverse events

2) Pharmacokinetic study

**8-1-2. Phase Ib part**

1) Safety: Types, frequency, and severity of adverse events

2) Effects on elimination of regulatory T cells

**8-2. Secondary endpoints**

**8-2-1. Phase Ia part**

1) Regulatory T-cell elimination efficacy

2) Efficacy: tumor shrinkage, progression-free survival (PFS), overall survival (OS)

**8-2-2. Phase Ib part**

1） Efficacy: tumor shrinkage, progression-free survival (PFS), overall survival (OS)

2) Determination of recommended doses for Phase II trials and beyond

**8-3. Definition of evaluation items**

**8-3-1. Definition of primary evaluation items**

1) Phase Ia

(1) Safety

The maximum tolerated dose (MTD) and dose-limiting toxicity (DLT) will be determined, and the type, frequency, and severity of adverse events will be observed. The Common Terminology Criteria for Adverse Events (CTCAE) v4.0 Japanese translation of the JCOG version^13)^ will be used for grading adverse events. Adverse events will be graded according to CTCAE v4.0.

The MTD is defined as the dose at which the number of subjects who developed DLT during the period up to 28 days after the start of treatment with the investigational drug was 3 or more. However, if the MTD is not reached at any dose, 1.0 mg/kg shall be the dose that was found to be well tolerated.

The observation period for DLT will be up to 28 days after the start of study drug administration. Among adverse events for which a causal relationship to the investigational drug cannot be ruled out, the following events will be considered DLTs.

Grade is determined according to CTCAE v4.0.

① Hematologic toxicity of Grade 4 or higher lasting for more than 7 days^*1^.

*1 "Febrile neutropenia" is defined as Grade 3 or higher regardless of duration, and "Neutropenia" is defined as treatment with G-CSF even if it is Grade 3 or lower. Lymphocytopenia" and "CD4 lymphocytopenia" are excluded.

② Non-hematologic toxicity of Grade 3 or higher

Symptoms judged to be "injection-related reactions" ("fever," "headache," "pain," "pruritus," "allergic reaction/hypersensitivity," etc.) are defined as Grade 4 or higher DLT. In addition, only AST (GOT), ALT (GPT), ALP, total bilirubin, creatinine, and potassium should be included in the clinical examination.

③ Others

i) Toxicity that caused the scheduled administration of the investigational drug to exceed 2 weeks beyond the specified scheduled administration date due to a Grade 3 in any of the symptoms judged to be "infusion-related reactions" in 2).

ii) Toxicities other than the above that prevented administration of the study drug more than twice during the DLT observation period due to the same event.

(2) Pharmacokinetics

Pharmacokinetic parameters will be calculated from the plasma concentration of Mogamulizumab during repeated dosing of the study drug. To evaluate the plasma pharmacokinetics of Mogamulizumab during repeated dosing. Plasma Mogamulizumab pharmacokinetic parameters will be calculated from Cmax after the first, fifth and eighth doses, Ctrough after the first, fifth and seventh doses, AUC0-7 day after the first and eighth doses and t1/2, CL, Vss and MRT if possible. In addition, the accumulation rate during repeated dosing periods will be calculated. Pharmacokinetic parameters in plasma will be calculated using pharmacokinetic analysis software WinNonlin.

2) Phase Ib

(1) Safety

The type, frequency, and severity of adverse events will be observed. Adverse events will be judged according to CTCAE v4.0.

(2) Regulatory T-cell elimination effect

The ratio of CD25-positive and Foxp3-positive cells among CD4-positive T cells will be measured by FCM analysis. The CD4-positive rate among lymphocytes is also measured and converted to a ratio among lymphocytes to calculate the number of CD4-positive, CD25-strongly positive and Foxp3-positive cells.

The effect of regulatory T-cell removal is determined by comparing the number of CD4-positive, CD25-positive and Foxp3-positive cells to baseline.

**8-3-2. Definition of secondary evaluation items**

1) Phase Ia

(1) Efficacy in eliminating regulatory T cells

See "8-3-1. Definition of Primary Endpoints 2) Phase Ib”.

2) Tumor shrinkage effect

① Evaluation of RECIST

Tumor shrinkage effect is evaluated according to RECIST ver. 1.1.

② Variation of RECIST

In addition to the evaluation of RECIST, assuming that there is a time lag between the start of administration of the investigational drug and the onset of tumor shrinkage, an evaluation method that defines target lesions, non-target lesions, and new lesions as follows (hereinafter referred to as the "RECIST variant method") will be used (the different parts from RECIST (ver.1.1) are underlined). ).

i) Target lesion

The target lesion should be a measurable lesion of at least 10 mm in longest diameter (15 mm in shortest diameter for lymph nodes) at baseline. In addition, measurable lesions with a maximum diameter of 10 mm or greater (15 mm or greater for lymph nodes) and non-target lesions that have grown to a maximum diameter of 10 mm or greater (15 mm or greater for lymph nodes) that are newly observed after the start of treatment with the study drug will be evaluated in addition to target lesions. There is no limit to the number of target lesions. Target lesions are evaluated by comparing the sum of the longest diameter (shortest diameter for lymph nodes) to baseline and using the criteria for target lesions in RECIST (ver. 1.1).

ii) Non-target lesions

Non-target lesions are defined as lesions other than target lesions at baseline. After the start of treatment with the investigational drug, non-target lesions are defined as lesions excluding those non-target lesions observed at baseline that have increased to a maximum diameter of 10 mm or more (15 mm or more for lymph nodes in terms of short diameter) after the start of treatment with the investigational drug and are evaluated as target lesions. Non-target lesions will be evaluated using the criteria for non-target lesions in RECIST (ver. 1.1).

iii) New lesions

New lesions are defined as bone lesions, soft membrane lesions, ascites, pleural/pericardial effusions, cutaneous lymphangitis, pulmonary lymphangitis, abdominal masses not visible on imaging studies, cystic lesions, meningitis, and other unmeasurable lesions observed after the start of study drug administration. Lesions less than 10 mm in longest diameter (lymph nodes less than 15 mm in shortest diameter) are not considered as new lesions. Measurable lesions with a maximum diameter of 10 mm or more (15 mm or more for lymph nodes) that are newly observed after the start of treatment with the study drug will be evaluated as target lesions and will not be treated as new lesions.

Even if the criteria for PD are met by Week 12, if the trial is continued after Week 12, it will not be treated as PD at that time unless it is confirmed as PD by RECIST's variant method The date of confirmation of PD by RECIST's variant method shall be the date when PD is first confirmed beyond 12 weeks. However, if the first evaluation beyond 12 weeks was PD, the PD confirmation date shall be the date of the first PD backward in the evaluation period from that time, and if there is no PD in the backward evaluation period, the date of the first PD beyond 12 weeks shall be analyzed as the PD confirmation date.

③ Common to RECIST evaluation and RECIST variants

In cases where necessary examinations could not be performed due to rapid worsening of the disease, the date of PD confirmation shall be the date when the worsening of the disease is clinically evident; in cases where the patient died before PD is confirmed, regardless of the reason for death, the most recent hospital visit date specified in the study protocol shall be considered as the PD confirmation date.

For patients who are determined to have CR or PR, a central image evaluation should be performed.

<About the RECIST variant>

Tumor shrinkage will be evaluated using a variant of RECIST (ver. 1.1), which was developed based on the recommendations of the Cancer Vaccine Clinical Trial Working Group and referring to the Immune-related response criteria (irRC). This method is based on the idea that PD is acceptable and will be evaluated retrospectively, since there will be a time lag before the actual clinical effect is manifested in cancer immunotherapy. Based on this idea, in this clinical trial, even if the criteria for PD are met, the trial will continue, and if no clinical response is observed thereafter, the date will be retroactively determined to be PD. In addition, with regard to the determination of tumor shrinkage, even if a new measurable lesion is detected after the start of treatment with the investigational agent, if the tumor as a whole has shrunk, it is considered to indicate that the investigational agent has been effective. Therefore, in the variant of RECIST (ver. 1.1), measurable lesions with a minimum diameter of 10 mm (15 mm for lymph nodes) are defined as target lesions, and the sum of the longest diameter (shortest diameter for lymph nodes) is used to evaluate the target lesions. Measurable lesions with a maximum diameter of 10 mm or more (15 mm or more in short diameter for lymph nodes) that are newly observed after the start of treatment with the investigational drug will be evaluated as target lesions and will not be treated as new lesions. In RECIST (ver. 1.1), the number of target lesions is limited to a maximum of 2 lesions per organ, for a total of 5 lesions per organ. Therefore, it is assumed that the number of target lesions may exceed the number of target lesions specified in RECIST (ver. 1.1). Therefore, there is no limit on the number of target lesions.

(3) Progression-free survival

Progression-free survival (PFS) shall be defined as the period from the start date of the investigational drug administration to the date of PD confirmation during the study period. For patients who died before PD is confirmed, the date of PD confirmation shall be the date of the most recent hospital visit as specified in the study protocol, regardless of the reason for death.

(4) Overall Survival

Overall Survival (OS) is defined as the period from the start date of study drug administration to death from any cause. For patients who survived without confirmed death, the date of last confirmed survival shall be considered as the date of last confirmation of survival, and for patients who were lost to follow-up, the date of last confirmed survival shall be considered as the date of discontinuation.

2) Phase Ib

(1) Tumor reduction effect

See "8-3-2. Definition of Secondary Endpoints 1) Phase Ia".

(2) Progression-free survival

See "8-3-2. Definition of Secondary Endpoints 1) Phase Ia".

(3) Overall survival

See "8-3-2. Definition of Secondary Endpoints 1) Phase Ia".

(4) Determination of recommended doses for Phase II and beyond

Recommended doses for Phase II and beyond will be determined based on a comprehensive review of the safety and efficacy in eliminating regulatory T cells in Phase Ib.

**8-3-3. Other items defined**

1) Common to both Phase Ia and Phase Ib

(1) Antigen-specific antibody immunoreactivity test (NY-ESO-1 and XAGE-1b)

Antibody titers against NY-ESO-1 or XAGE antigens in serum are measured using recombinant NY-ESO-1 protein or synthetic XAGE protein by ELISA.

An IgG titer exceeding the mean of ELISA absorbance + 3 S.D. in healthy human serum is defined as positive, and an "increase" or "decrease" is defined as a change in the maximum dilution fold that shows positive results.

(2) Evaluation of regulatory T cells (optional)

Cell surface staining is performed using CD3, CD4, CD25, CD45RA, etc., which are markers of regulatory T cells. In addition, intracellular staining with transcription factor Foxp3, etc., and analysis using FCM. Among the regulatory T cells, the fraction with a strong inhibitory function that suppresses the anti-tumor immune response is accurately identified after careful removal of dead cells. Since this fraction strongly expresses CCR4, we will examine changes in this fraction upon administration of Mogamulizumab.

(3) Antigen-specific antibody immunoreactivity test (antigens other than NY-ESO-1 and XAGE-1b) (optional)

An ELISA using serum is performed to evaluate the enhancement effect of antigen-specific immune response. Measure the response to tumor antigens MAGE-A1, MAGE-A3, MAGE-A4, CT7/MAGEC1, CT10/MAGEC2, CT45, CT46/HORMAD1, SOX2, SSX2, p53 and other antigen proteins.

(4) Antigen-specific cellular immune response test (optional)

To evaluate the enhancement effect of antigen-specific immune response, antigen-specific CD4 and CD8 T cell responses are measured using peripheral blood. To observe the response to NY-ESO-1 and XAGE-1b, which are expressed 20-50% in the carcinoma of interest, CD4/CD8 T cells will be co-cultured with antigen-presenting cells to which NY-ESO-1 or XAGE-1b protein is added, and IFN-γ-producing cells will be detected by IFN-γ secretion assay.

(5) Evaluation of cytokine production (optional)

The effector cell side is affected by antigen stimulation due to changes in regulatory T cells induced by administration of Mogamulizumab, and the ability to produce multiple effector cytokines such as IL-2, TNF-α and IFN-γ is evaluated by intracellular staining. After antigen stimulation, cytokines are trapped intracellularly using transporter inhibitors, and the cell surface is stained with Live/Dead staining kit, anti-human CD3, CD4, CD8, CD45 antibodies, and fixed, and intracellularly stained with human IFN-γ antibody, human TNF-α antibody, human IL-2 antibody Intracellular staining with human IFN-γ, human TNF-α and human IL-2 antibodies, if necessary, clones of antigen-specific T cells will be established for HLA binding and epitope analysis. In addition, antigen-specific T cells will be monitored using tetramers.

6) Immunocompetent and regulatory cell analysis (optional)

Immunoregulatory cells such as regulatory T cells and MDSCs, helper T cell fractions (Th1, Th2, Th17), and the percentage of monocytes and dendritic cells are analyzed over time by FCM. In the analysis of immunosuppressor cells, regulatory T cells are stained with anti-human CD4, CD8, CD25, and CD45RA antibodies, and MDSCs are stained on the cell surface with anti-human CD3, CD11b, CD14, CD15, CD33, CD45 and HLA-DR antibodies.

The expression of immunoenhancing factors such as ICOS, OX-40, GITR, 4-1BB, and immunosuppressive factors such as PD-1, BTLA, TIM-3, 2B4, etc. are analyzed over time by FCM using the respective antibodies.

(7) Immunohistochemistry (optional)

Tissue biopsy specimens such as surgically removed tissues from the past or tissue biopsies obtained during endoscopic examinations as part of routine medical examinations will be used to determine the bio-distribution of immune-related cells. When possible, one or two additional biopsies should be obtained from the tumor site in addition to the biopsies normally required for diagnosis during endoscopic examination.

All specimens collected are used for pathological diagnosis at each institution. After diagnosis, unstained specimens are prepared from the stored blocks and analyzed for immune-related molecules by immunostaining.

(8) Gene analysis (optional)

In order to explore genomic biomarkers that may predict the clinical efficacy of mogamulizumab and the development of toxicity (autoimmune adverse events: skin rash, interstitial pneumonia, etc.), gene polymorphism analysis using DNA derived from peripheral blood mononuclear cells before the start of treatment and copy number analysis will be used for genome-wide association study (GWAS) using DNA derived from peripheral blood mononuclear cells before the start of treatment, and comprehensive expression analysis of miRNAs in serum.

**9. Observation items and methods**

**9-1. Clinical trial schedule**

The prescribed observations and examinations will be conducted according to the following schedule1. Patients will be hospitalized from before the first administration to the day after the first administration.

For subjects in Phase Ia, the investigators will obtain consent for the continuation of the clinical trial again prior to the 5th administration.

After 8 doses, if the subject has shown a regulatory T-cell elimination effect and antigen-specific antibody immunoreactivity or SD or higher on RECIST, the investigator may continue to administer the investigational drug if the subject wishes to receive maintenance dosing and the investigator determines that maintenance dosing is appropriate.

The first dose of investigational drug is defined as 1 week.


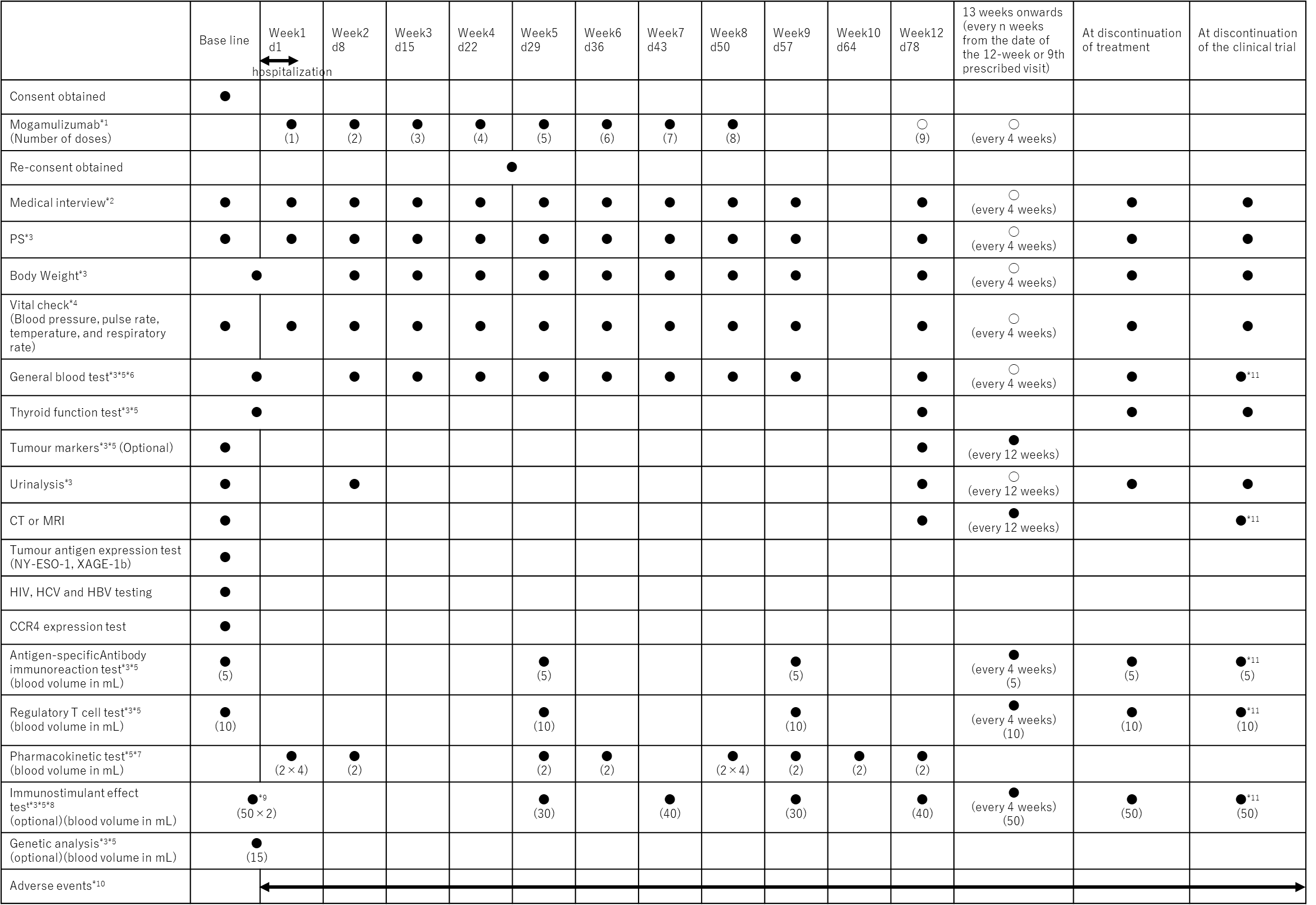
Table 1. Clinical trial schedule

○：Additional items to be performed when maintenance dosing is performed after 8 doses.

*1: The ninth and subsequent doses of the investigational drug should be administered only when SD or better is confirmed in the regulatory T-cell elimination effect and antigen-specific antibody immunoreactivity or RECIST, the subject wishes to receive a maintenance dose of the investigational drug, and the investigator and others consider that the maintenance dose of the investigational drug is appropriate.

*2: Continuous observation from before the start to the end of study drug administration.

*3: To be conducted before administration of the investigational medicinal product during the period of administration of the investigational medicinal product.

*4: Before administration of the investigational drug, at 30 minutes, 1 hour, 1.5 hours after the start of administration, at the end of administration, 30 minutes and 1 hour after the end of administration.

*5: If it is difficult to collect the prescribed volume of blood due to signs of anemia, etc., the following tests should be performed in consideration of subject safety: 1. General blood test (facility prescribed volume), 2. Thyroid function test (facility prescribed volume), 3. Regulatory T cell test (10 mL), 4. Pharmacokinetic test (Phase Ia part only, 2 mL), 5. Antigen-specific antibody immunological reaction test (5mL), 6. Genetic analysis (15mL), 7. Immunopotentiation effect test (30~50mL), 8. Tumor markers (facility's specified volume), as much blood as possible in the order of priority.

*6: Testing is also performed at week 12 after the last dose of the study drug.

*7: The volume of blood drawn per dose should be 2 mL. See Table 2 for the timing of the test.

*8: The test items should be those indicated in (2), (4) to (6) of 8-3-3 Definition of other items.

*9: Blood sampling on multiple days is acceptable.

*10: Adverse events should be observed until 12 weeks after the last dose of the study drug. However, for non-hematological toxicities of Grade 3 and above, the observation period for adverse events is up to 24 weeks.

*11: If no maintenance dose is given after 8 doses and the study is discontinued after 12 weeks of testing, only general blood tests, CT or MRI, antigen-specific antibody immunoreactivity tests, regulatory T-cell tests and immunoenhancing effect tests should be performed when the study is discontinued.


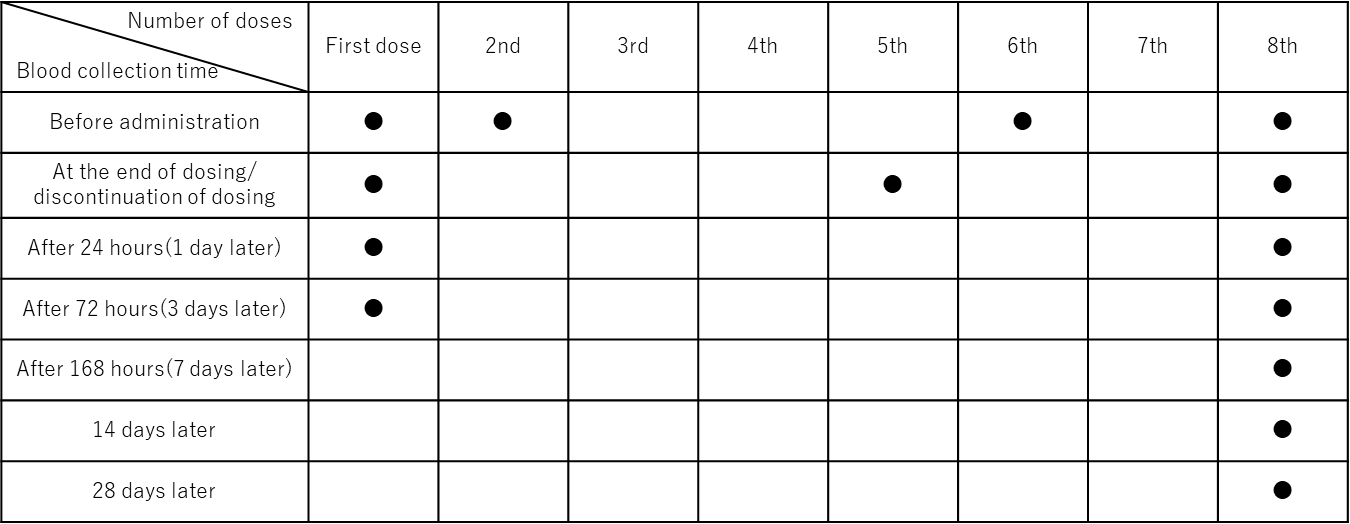
Table 2. Schedule of blood sampling for pharmacokinetic testing (only in phase Ia part)

**9-2. Patient background**

The following items should be verified and included in the case report form.

Date of obtaining consent, date of birth, gender, height, primary disease (name of primary disease, pathological or histological type, date of diagnosis, diagnostic method, TNM classification, staging), history of treatment for primary disease, medical history*, complications**, whether tumor antigen (NY-ESO-1, XAGE-1b) expression, HIV test***, HCV test***, HBs antigen test***, HBc antibody test***, HBs antibody test***, HBV-DNA test (performed if HBc or HBs antibody test result is positive)**, CCR4 expression test (IHC), arterial blood oxygen saturation, ECG, left heart ejection fraction, pregnancy test*** (hCG test: limited to premenopausal women)

*: Clinically problematic disease cured between 1 year prior to the start of the study drug and case enrollment, as well as any disease that was previously diagnosed more than 1 year prior to the start of the study but deemed important for the patient background.

**: Diseases that have not been cured at the time of case enrollment

***: To be conducted within 4 weeks prior to case enrollment.

**9-3. Status of investigational drug administration and concomitant treatment**

1) Administration of investigational drug

The date and time of administration of the investigational drug should be noted in the case report form. If administration of the investigational drug is discontinued, the reason should be stated in the case report.

2) Status of concomitant treatment

For all concomitant medications and concomitant therapies administered to the subject from Week 1 to the time of completion or discontinuation of the investigational drug (4 weeks after the last administration), the daily dose, route of administration, start and end dates, and purpose of administration should be noted in the case report.

**9-4. Efficacy parameters**

**9-4-1. Antigen-specific antibody immunoreaction tests (NY-ESO-1 and XAGE-1b)**

Blood samples will be drawn at baseline, every 4 weeks after 5, 9, and 12 weeks if possible, and every 4 weeks from the 9th dosing date for maintenance dosing, and at the time of discontinuation of the study, and measured by an external laboratory. However, on the day of administration of the investigational drug, blood should be drawn prior to administration of the investigational drug. If administration of the investigational drug is discontinued, blood should be drawn at the time of discontinuation of administration, and every 4 weeks after discontinuation if possible. Measurement results will be submitted directly to the data center from an external laboratory.

**9-4-2. Regulatory T cell tests (FOUR-COLOR FOXP3 test, CD4/CD25/CCR4 test and MLA CD45 gating)**

Peripheral blood will be used to evaluate the decrease in the number of activated regulatory T cells by immunological monitoring. For this purpose, blood samples will be drawn at baseline, every 4 weeks after 5, 9, and 12 weeks when possible, and every 4 weeks after the 9th dose date during maintenance dosing, and at the time of discontinuation of the study, and measured by an external laboratory. However, on the day of administration of the investigational drug, blood should be drawn prior to administration of the investigational drug. If administration of the investigational drug is discontinued, blood should be drawn at the time of discontinuation of administration, and every 4 weeks after discontinuation if possible. Measurement results will be submitted directly to the data center from an external laboratory.

**9-4-3. CT and MRI**

Measurements will be taken at baseline and at Week 12. Thereafter, it will be performed every 12 weeks. It will also be performed every 12 weeks after discontinuation of the investigational drug if possible, and measurements will be taken at the time of discontinuation of the study.

The CT or MRI scan should be performed using the same model at each site and under the same imaging conditions for each subject as far as possible.

The cases judged as CR or PR by the RECIST method are objectively and uniformly judged by the Central Evaluation Committee of Imaging, which is a third-party organization.

The investigator submits the image data of cases judged as CR or PR to the Central Radiological Evaluation Committee at an appropriate time in accordance with the "Procedures for Central Radiological Evaluation," which is separately stipulated. If the judgment of the investigator and that of the Central Radiological Evaluation Committee differ, the judgment of the Central Radiological Evaluation Committee shall be the final evaluation.

**9-4-4. Survival surveys and post-treatment**

Survival period of patients who did not receive maintenance dosing and those who received maintenance dosing but discontinued it will be investigated until the end of the clinical trial as described in "14-3. In case of death, the date of death and the reason shall be noted in the case report form”.

If post-treatment was given, the details of the treatment, its duration, efficacy, and duration of response shall be investigated.

**9-4-5. Tumor markers (optional)**

Measure at baseline and week 12. Thereafter, it should be measured every 12 weeks. Also, after discontinuation of the investigational drug, it should be conducted every 12 weeks as much as possible. The measurement results should be documented in the case report form.

However, the implementation of tumor marker measurement and the items of tumor markers to be measured are optional.

**9-4-6. Evaluation of regulatory T cells (optional)**

Mononuclear cells in peripheral blood and tumor tissue will be isolated and immunological monitoring will be used to assess the decrease in the number of activated regulatory T cells. For this purpose, blood samples will be drawn at baseline, every 4 weeks after 5, 9, and 12 weeks if possible, and every 4 weeks after the 9th dose date during maintenance dosing, and at the time of discontinuation of the study, and measured by an external laboratory. However, on the day of administration of the investigational drug, blood should be drawn prior to administration of the investigational drug. If administration of the investigational drug is discontinued, blood should be drawn at the time of discontinuation and every 4 weeks after discontinuation if possible. One or two additional pieces of tumor tissue will be collected at the time of endoscopy or other tissue biopsy as part of routine medical care. Results are submitted directly to the data center.

**9-4-7. Antigen-specific antibody immunoreactivity testing (for antigens other than NY-ESO-1 and XAGE-1b) (optional)**

Blood samples will be drawn at baseline, every 4 weeks after 5, 9, and 12 weeks if possible, and every 4 weeks from the 9th dose date for maintenance dosing, and at the time of discontinuation of the study, and measured by an external laboratory. However, on the day of administration of the investigational drug, blood should be drawn prior to administration of the investigational drug. If administration of the investigational drug is discontinued, blood should be drawn at the time of discontinuation of administration, and every 4 weeks after discontinuation if possible. Blood samples for this test should be collected in conjunction with the antigen-specific antibody immunoreactivity tests (NY-ESO-1 and XAGE-1b) described in 9-4-1. The results are submitted directly to the data center from an external laboratory.

**9-4-8. Antigen-specific cellular immuno-correspondence testing (arbitrary)**

Blood samples will be drawn at baseline, 7, 12 weeks, every 4 weeks after 12 weeks, if possible, every 4 weeks after the 9th dose at the maintenance dose, and at the time of discontinuation of the study, and the results will be measured at an external laboratory. However, on the day of administration of the investigational drug, blood should be drawn prior to administration of the investigational drug. If administration of the investigational drug is discontinued, blood should be drawn at the time of discontinuation of administration, and every 4 weeks after discontinuation if possible. Measurement results will be submitted directly to the data center from an external laboratory.

**9-4-9. Cytokine productivity evaluation (optional)**

Blood samples will be drawn at baseline, 7, 12 weeks, every 4 weeks after 12 weeks, if possible, every 4 weeks after the 9th dose at the maintenance dose, and at the time of discontinuation of the study, and measured by an external laboratory. However, on the day of administration of the investigational drug, blood should be drawn prior to administration of the investigational drug. If administration of the investigational drug is discontinued, blood should be drawn at the time of discontinuation of administration, and every 4 weeks after discontinuation if possible. Measurement results will be submitted directly to the data center from an external laboratory.

**9-4-10. Analysis of immune response cells and control cells (optional)**

Blood samples will be drawn at baseline, 7, 12 weeks, every 4 weeks after 12 weeks, if possible, every 4 weeks after the 9th dose at the time of maintenance administration, and at the time of discontinuation of the study, and measurements will be performed at an external laboratory. However, on the day of administration of the investigational drug, blood should be drawn prior to administration of the investigational drug. If administration of the investigational drug is discontinued, blood should be drawn at the time of discontinuation of administration, and every 4 weeks after discontinuation if possible. Measurement results will be submitted directly to the data center from an external laboratory.

**9-4-11. Immunohistochemistry (optional)**

Tissue biopsy specimens from past surgical extractions or tissue biopsies obtained during endoscopy as part of routine medical examinations are used for staining. When possible, one or two additional biopsies should be obtained from the tumor site in addition to the biopsies normally required for diagnosis during endoscopy.

Both specimens collected are used for pathological diagnosis at each facility. After diagnosis, unstained specimens are prepared from the stored blocks. Stained results are submitted directly to the data center from an external laboratory.

**9-4-12. Gene analysis (optional)**

Blood samples are collected at baseline (before the start of study drug administration) and assayed by an external laboratory. The results will be submitted directly to the data center from the external laboratory. 9-5.

**9-5. Safety-related items**

**9-5-1. PS (Performance status)**

Observe at baseline, at each dose of study drug, and at Weeks 9 and 12. It will also be observed at the time of discontinuation of dosing and at the time of discontinuation of the study during the period of study drug administration. However, for the date of administration of the investigational drug, observation will be conducted prior to administration of the investigational drug. The score shall be in accordance with the definition of Performance Status Scale/Scores ECOG.

**9-5-2. Vital signs (blood pressure, pulse rate, temperature, respiratory rate)**

Observed at baseline and at each dose of study drug, before administration, 30 minutes, 1 hour, 1.5 hours, at the end of administration, 30 minutes and 1 hour after the end of administration. Observations will also be made at Weeks 9, 12, at the time of discontinuation of dosing, and at the time of discontinuation of the study during the period of study drug administration. The observation results will be documented in the case report form.

**9-5-3. Body weight**

Body weight shall be measured at baseline, at each dose of study drug, and at Weeks 9 and 12. It will also be measured at the time of discontinuation of administration and at the time of discontinuation of the study during the study drug administration period. However, on the day of administration of the investigational drug, measurement shall be performed before administration of the investigational drug. The measurement results shall be documented in the case report form.

**9-5-4. General blood tests**

Blood tests shall be performed at baseline, each time the investigational drug is administered, and at Weeks 9 and 12. Also, at the time of discontinuation of dosing and at the time of discontinuation of the study. In addition, the test shall be performed at Week 12 after the last dose of the investigational drug. However, for the date of administration of the investigational drug, the test shall be conducted prior to administration of the investigational drug. The test results shall be documented in the case report form. The test items shall be as follows

1) Hematological tests

Red blood cell count, hemoglobin, hematocrit, white blood cell count, white blood cell fraction, platelet count

2) Blood biochemical tests

AST (GOT), ALT (GPT), ALP, LDH, total protein, albumin, total bilirubin, urea nitrogen, creatinine, uric acid, sodium, potassium, chlor, calcium, phosphorus

**9-5-5. Thyroid function test**

Tests shall be performed at baseline, Week 12, at the time of discontinuation of treatment, and at the time of discontinuation of the study during the period of study drug administration. However, for the date of administration of the investigational drug, the test shall be performed prior to the administration of the investigational drug. The test results will be documented in the case report form. The test items shall be as follows

FT3, FT4, TSH

**9-5-6. Urinalysis**

Perform at baseline, 2 and 12 weeks. At maintenance dosing, testing will be performed every 12 weeks from the 9th dosing date. However, for the date of administration of the investigational drug, the test shall be performed prior to the administration of the investigational drug. In addition, the test shall be performed at the time of discontinuation of administration and at the time of discontinuation of the clinical trial during the period of administration of the investigational drug. The test results shall be documented in the case report form. The test items shall be as follows

1) Urine qualitative

Protein, sugar, urobilinogen, occult blood

**9-6. Pharmacokinetics (Phase Ia part)**

Before the first dose, at the end of administration, 24 hours, 72 hours, and 168 hours after the end of administration (7 days later, before the second dose), at the end of the fifth dose, 168 hours after the end of administration (7 days later, before the sixth dose), before the eighth dose, at the end of administration, 24 hours, 72 hours, and 168 hours (7 days later), 14 days later, and 28 days later Blood samples are taken at 24 hours, 72 hours, 168 hours (after 7 days), 14 days, and 28 days after the end of administration, and the results are measured at an external laboratory. The results are submitted directly to the data center from the external laboratory. 10.

**10. Safety handling**

**10-1. Definitions**

**10-1-1. Definition of clinical examination value abnormalities and abnormal changes**

For blood and urine tests, normality and abnormality are determined for each measured value of each item, and then compared to the baseline to determine the presence or absence of abnormal fluctuations. If the normal range is not set, the investigator shall set it.

The investigators will determine whether or not there are any abnormal fluctuations based on the laboratory values before the first administration and the laboratory values performed after the administration. If an abnormal variation is detected, it should be treated as an adverse event in the laboratory values and entered in the case report form.

As with laboratory values, determine whether there are any abnormal changes in body weight, vital signs, etc. If abnormal changes are found, treat them as adverse events and enter them in the case report form.

**10-1-2 Definition of harmful events and side effects (harmful events with a negative causal relationship)**

An adverse event is any unfavorable medical event that occurs in a subject receiving an investigational drug. In other words, any unwanted or unintended signs (including abnormal changes in laboratory values), symptoms, or illnesses that occur when the investigational drug is administered, regardless of whether or not there is a causal relationship with the investigational drug. In addition, adverse drug reactions (adverse events for which a causal relationship cannot be denied) are all adverse events other than those judged to have a "no relationship" causal relationship to the investigational drug.

Signs, symptoms, and abnormal changes in laboratory values associated with a disease (diagnostic name) shall be treated as adverse events with the diagnostic name. However, if an atypical or extremely severe sign or symptom occurs in the disease, the individual sign or symptom as well as the diagnosis should be treated as an adverse event.

The observation period for adverse events will be from the date of the first dose of study drug to 12 weeks after the last dose. However, for Grade 3 or higher non-hematologic toxicity, the observation period for adverse events shall be up to 24 weeks.

**10-1-3. Definition of harmful signs of rehabilitation**

Serious adverse event" means any of the following

1) Death

2) An event that may lead to death

3) An event requiring hospitalization or prolonged hospitalization for treatment

4) Disability

5) An event that may lead to disability

6) Events as serious as those listed in 1) to 5)

7) Congenital disease or abnormality in later generations

The following events are not regarded as serious adverse events.

When "hospitalization or prolonged hospitalization" occurs due to the administration of the investigational drug.

**10-1-4. Harmful signs related to symptoms and observations**

Symptoms and findings that newly develop after the first administration of the investigational drug, or symptoms and findings that were observed before the first administration of the investigational drug, but which the investigator determines to have worsened after the first administration of the investigational drug, shall be considered as adverse events related to symptoms and findings and entered in the case report form.

However, fluctuation of PS and appearance of new lesions shall not be treated as adverse events.

Aggravation of the underlying disease and complications resulting from it should be reported in accordance with "10-3-1. Serious Adverse Event Reporting Procedures" if they meet the definition of "10-1-3. In case of death, determine whether the cause is aggravation of the underlying disease or other reasons, e.g., due to the investigational drug.

**10-2. Survey items**

**10-2-1. Names of harmful events**

Enter the name of the symptom or diagnosis in the case of an adverse event related to symptoms and findings, and enter the name of the test item or the details of fluctuation in the case of an adverse event related to laboratory test values in the case report form.

**10-2-2. Date of onset (manifestation) and date of worst deterioration**

Enter the date and grade of onset and worst case. (The worst-case date is the date when the worst grade is first confirmed.) Enter the time of onset and worst case for acute infusion reaction or cytokine release syndrome.

**10-2-3. Severity of illness**

Grade should be determined according to CTCAE v4.0 and recorded in the case report form.

**10-2-4. Severity**

Select from the following codes.

1) Serious

2) Non-serious

**10-2-5. Treatment**

1) Treatment of investigational drug

Select from the following codes.

(1) No change

(2) Postponed

(3) Discontinued

(4) Not applicable

2) Other treatment (Enter the treatment method.)

Select the procedure other than the investigational drug from the following codes.

(1) None

(2) Reduction of concomitant medication

(3) Increase dose of concomitant drug

(4) Prescription of other drugs

(5) Other

**10-2-6. Outcome**

Describe the outcome confirmed by 4 weeks after the last observation period of the adverse event (16 weeks (28 weeks for Grade 3 or higher non-hematologic toxicity) after the last administration date of the study drug). Follow-up of adverse drug reactions (adverse events for which a causal relationship cannot be ruled out) will be conducted until they are confirmed to have recovered or become mild.

1) Recovery (when the patient returns to the same level as before the first administration of the investigational drug)

2) Slightly improved (the worst is over, but not back to the same level as before the first dose of the investigational drug)

3) Unrecoverable (the worst has continued)

4) Severe sequelae (the adverse event has resolved but other related symptoms remain)

5) Death (if the adverse event resulted in death)

6) Unknown (when the patient is unknown)

**10-2-7. Outcome date**

Enter also the date of outcome and grade confirmed by 4 weeks after the last observation period of the adverse event (16 weeks (28 weeks for non-hematologic toxicity of Grade 3 or higher) after the last administration date of the investigational drug). For acute infusion reactions or cytokine-releasing syndromes, the time of day should also be recorded.

**10-2-8. Causal relationship with investigational drug**

The following criteria should be used when determining the relevance of an investigational drug.

If the investigator determines that there is no association, the reason should be stated in the case report.

＜Criteria for determining the relevance of an investigational drug

1) No association

The adverse event is judged not to be related to the investigational drug because there is no reasonable possibility (time course between administration of the investigational drug and occurrence of the adverse event, pharmacological effects, etc.) within the scope of available information, and it can be judged that the adverse event has other causes such as aggravation of the primary illness.

2) May be related

After careful medical evaluation, the relationship between the event and the study drug administration is not certain or strongly suspected, but the existence of a causal relationship cannot be ruled out at all.

3) Possibly related

After careful medical evaluation, there is a high degree of certainty of a relationship between the event and the study drug administration.

4) Relevant

There is no doubt that the event occurred as a result of the administration of the investigational drug.

**10-3. Adverse event reporting and response**

Adverse events that must be reported are those that occur during the observation period of adverse events defined in "10-1-2. Definition of adverse events and adverse reactions (adverse events for which a causal relationship cannot be ruled out)" and "Serious adverse events" defined in "10-1-3. When such an adverse event occurs”, it should be reported in accordance with "10-3-1. Serious Adverse Event Reporting Procedures”.

In addition, if an adverse event that is not a serious adverse event but meets the criteria for DLT (defined in 8-3-1. Primary Endpoints) occurs in the Phase Ia portion, regardless of whether or not there is a causal relationship, the investigator should take appropriate measures, prepare a "Report on Adverse Events Related to Dose Increases" and promptly submit it to the Trial The report shall be promptly submitted to the Clinical Trial Coordinating Committee.

**10-3-1. Serious adverse event reporting procedures**

1) Immediate report (within 24 hours from the time of knowledge of the occurrence)

In the event of a reportable adverse event, the investigator shall take appropriate measures and report it to the head of the site, regardless of whether there is a causal relationship with the investigational drug or not, and shall immediately (within 24 hours from the time when he/she becomes aware of the occurrence) submit a "Report on Serious Adverse Events (Medical Form 12-1)" to the site's coordinating committee, stating the information available at that time. The information available up to that point shall be reported to the Clinical Trial Coordinating Committee.

2) Detailed report (within 3 days from the time when the investigator becomes aware of the occurrence)

The investigator shall prepare the "(Medical) Form 12-1, 2 Report on Serious Adverse Events" within 3 days of learning of the occurrence of a serious adverse event, and report it to the head of the investigational site as well as to the Clinical Trial Coordinating Committee. At that time, the relationship between the investigational drug and the adverse event should be fully considered. However, if the adverse event is a fatal or life-threatening serious adverse event for which a causal relationship with the unknown investigational drug cannot be denied, a detailed report shall be submitted to the TIC immediately (the next day at the latest). The TIC shall also immediately notify the investigators and investigational drug providers at other sites.

3) Response

The investigator who conducts the clinical trial himself/herself shall consider how to respond and take necessary measures under the coordination of the Clinical Trial Coordinating Committee. In case of administration of the investigational drug or discontinuation of the clinical trial, the investigator must promptly report to the Clinical Trial Coordinating Committee.

The TIC shall also request the Efficacy and Safety Evaluation Committee to deliberate and decide whether the clinical trial should be continued or not.

**10-3-2. Evaluation and recommendations by the effectiveness and safety assessment committee**

The Efficacy and Safety Evaluation Committee, which is requested to make an evaluation, shall review the contents of the "Report on Serious Adverse Events" and, if necessary, give instructions for detailed investigation after consultation with the Trial Coordinating Committee.

The Effectiveness and Safety Evaluation Committee will make an evaluation based on all information submitted and make recommendations to the Clinical Trial Coordinating Committee regarding the necessity of countermeasures and their contents. Possible measures include discontinuation of the clinical trial, interruption/continuation of enrollment/resumption of enrollment, modification of the clinical trial protocol, modification of the consent document, lot inspection of the investigational drug, provision of adverse event information to all investigational sites, and review by the Clinical Trial Review Committee.

**10-3-3. Determination of measures**

The person who conducts the clinical trial himself/herself shall make a decision on the necessity of countermeasures and their contents based on the recommendation from the Effectiveness and Safety Assessment Committee. The investigator who conducts the clinical trial himself/herself will inform the Efficacy and Safety Evaluation Committee, the Clinical Trial Review Committee, and the investigator himself/herself of the decision as necessary. When contacted by other sites, the investigator who conducts the clinical trial himself/herself will inform the Clinical Trial Review Committee of the contents of the countermeasures and the reasons for them, as necessary.

**10-4. Expected side effects**

(1) Skin disorders

A domestic Phase I clinical trial in patients with CCR4-positive relapsed/relapsed ATL and PTCL (Study No. 0761-0501), a domestic Phase II clinical trial in patients with CCR4-positive relapsed/relapsed ATL (Study No. 0761-002), a domestic late Phase II clinical trial in untreated patients with CCR4-positive first-episode ATL A late phase II study in Japan in patients with relapsed/relapsed CCR4-positive peripheral T/NK cell lymphoma (Study No. 0761-003), a late phase II study in Japan in patients with CCR4-positive relapsed/relapsed peripheral T/NK cell lymphoma (Study No. 0761-004), and a European phase II study in patients with CCR4-positive relapsed/refractory PTCL (Study No. 0761-007). During the 0761-002 study, two dermatologists attended a case review of dermatological symptoms with the attendance of two dermatologists. The committee agreed that "Stevens-Johnson syndrome" may have been caused by cytomegalovirus, but that adverse skin reactions other than "Stevens-Johnson syndrome" were caused by the pharmacological action of mogamulizumab. However, the investigators were of the opinion that adverse skin symptoms other than "Stevens-Johnson syndrome" were caused by the pharmacologic effects of mogamulizumab. The study concluded that these skin symptoms were not considered normal drug eruptions because they were not severe enough to be controlled with oral steroids or recovered spontaneously even if treatment was continued after the onset of skin eruptions. The adverse events of skin symptoms with mogamulizumab do not seem to be so harmful when compared with the efficacy of mogamulizumab. However, sufficient caution and appropriate treatment should be taken when skin symptoms occur due to the administration of mogamulizumab, and depending on the condition of skin symptoms, the administration of the investigational drug may be postponed and attention should be paid to the development of bacterial infections. From the launch of POTELIGEO® 20 mg for intravenous infusion (May 29, 2012) to February 28, 2013, three cases of toxic epidermal necrolysis in three patients and three cases of Stevens-Johnson syndrome in three patients were reported. One of the three patients developed sepsis and died. Therefore, on December 4, 2012, the company revised the instructions for use in the package insert regarding severe skin disorders, including toxic epidermal necrolysis and Stevens-Johnson syndrome.

(2) Injection-Related Reactions/Cytokine Release Syndrome and Tumor Decay Syndrome

Results of Study 0761-0501 and Study 0761-002 showed that, similar to other antibody drugs, "injection reactions" such as "fever," "chills," and "tachycardia" were frequently observed with mogamulizumab (overall incidence 86% (37/43 patients), of which Grade 3 The overall incidence was 86% (37/43 patients), of which Grade 3 or higher was 2% (1/43 patients)]. In the 0761-0501 study, "cytokine release syndrome" was observed in one patient. All events resolved spontaneously or with appropriate medical treatment, except for the subject who was deemed to have no further adverse events due to worsening of the underlying disease. Appropriate measures (e.g., infusion therapy, oxygen inhalation, steroid prescription, etc.) should be taken if symptoms develop.

In Study 0761-002, "Tumor Decay Syndrome" occurred in one patient (Grade 3). In the post-marketing surveillance of POTELIGEO® 20 mg for intravenous infusion (May 29, 2012 - November 28, 2012), one death due to "tumor collapse syndrome" was reported. Based on the above, due attention should be paid to the development of TLS due to rapid tumor cell destruction.

(3) Hematologic toxicity (lymphocyte count decreased, neutrophil count decreased, white blood cell count decreased, platelet count decreased)

In Study 0761-0501 and Study 0761-002, the most frequently observed adverse events were lymphocyte count decreased (overall incidence 95% (41/43 patients), of which 70% (30/43 patients) were Grade 3 or higher), neutrophil count decreased (overall incidence 56% (24/43 patients), of which 19% (8/43 patients) were Grade 3 or higher), and platelet count decreased (overall incidence 95% (41/43 patients), of which 70% (30/43 patients) were Grade 3 or higher). (56% (24/43), of which 19% (8/43) were Grade 3 or higher), "decreased white blood cell count" (65% (28/43), of which 23% (10/43) were Grade 3 or higher), "decreased platelet count" (53% (23/43), of which 12% (5/43) were Grade 3 or higher), and "decreased blood cell count" (53% (23/43), of which 12% (5/43) were Grade 3 or higher). Aggravation of primary disease and transfer to other hospitals Except for subjects whose adverse events were deemed unnecessary to be followed up due to worsening of the underlying disease or transfer to another hospital, all events were confirmed to have recovered by natural history, G-CSF prescription, or appropriate treatment with platelet transfusion. Platelet function tests (β-thromboglobulin and platelet factor 4) were performed on some subjects in Study 0761-0501, but no abnormal levels of β-thromboglobulin or platelet factor 4 were found in the one subject with Grade 2 thrombocytopenia.

Based on the above, appropriate measures should be taken when hematologic toxicity develops.

(4) Hepatitis and fulminant hepatitis due to reactivation of hepatitis B virus

Hepatitis B" was observed in one patient who was re-administered Mogamulizumab. The patient was found to be positive for HBc antibodies prior to enrollment in the study, and the possibility that Mogamulizumab reactivated the hepatitis B virus and contributed to the development of hepatitis B could not be ruled out.

In view of the above, subjects who tested positive for HBs antigen or HBV-DNA at the time of study enrollment were excluded from the study, and those who tested positive for HBc or HBs antibodies were tested for HBV-DNA during the study period and at an appropriate time after the completion of the study, and if viral reactivation was confirmed, appropriate measures should be taken to prevent the reactivation of the hepatitis B virus. If viral reactivation was confirmed, appropriate measures (prescription of antiviral drugs) would be taken. In addition, one patient who developed fulminant hepatitis after reactivation of hepatitis B virus and died was reported from the launch of "POTELIGEO® 20 mg for intravenous infusion" (May 29, 2012) to February 28, 2013.

The patient was an ATL patient with chronic hepatitis who tested positive for HBs antigen and had elevated levels of HBV-DNA after chemotherapy. The patient was started on Clozapine in combination with entecavir, and no significant abnormality was observed in liver enzymes on the day of the third dose of Clozapine, but when the patient came to the hospital for the fourth dose of Clozapine, fatigue and a sharp increase in liver enzymes were observed.

Although hepatitis B virus was probably already activated by the chemotherapy administered prior to Clozapine administration, the possibility that Clozapine was involved in further activation of the hepatitis B virus cannot be ruled out. Therefore, liver function test values and hepatitis virus markers should be monitored as needed, and appropriate measures should be taken if abnormalities are observed.

(5) Infectious diseases

Among the serious adverse events of "Infectious and parasitic diseases" in Japan and overseas, the events for which a causal relationship to mogamulizumab could not be ruled out were pneumonia and cytomegalovirus chorioretinitis in 2 patients each, oral candidiasis, infection, cytomegalovirus infection, and one each of "cytomegalovirus pneumonia," "viral encephalitis," "herpes esophagitis," "herpes zoster," and "hepatitis B" were reported.

In addition, one death due to "cytomegalovirus infection" was reported in the post-marketing surveillance (May 29, 2012 to November 28, 2012) of POTELIGEO® 20 mg for intravenous infusion.

Based on the above, adequate precautions against infection should be taken, and further, appropriate measures for infection prevention (e.g., anti-tuberculosis drugs, ST drugs, antifungal agents, etc.) should be taken if necessary.

**10-5. Action to be taken when adverse effects occur**

**10-5-1. Reactions associated with injection**

If an injection reaction is observed, immediately consider discontinuing administration or slowing down the administration rate, and promptly take appropriate measures according to the symptoms. 10-5-2.

**10-5-2. Skin disorder**

From the start of administration of this product, appropriate measures should be taken if any skin disorder is observed in collaboration with a dermatologist.

Since symptoms may progress rapidly and become severe in a short period of time, appropriate measures such as the use of a sufficient number of corticosteroids should be taken from the early stage of the onset of skin disorders. Corticosteroids should be considered for oral administration from the Grade 2 stage. Especially in cases of mucocutaneous disorders or when symptoms are ongoing, a sufficient dose (1 mg/kg prednisolone equivalent) should be used from the early stages of administration, and steroid pulse therapy should be administered in some cases.

If complications are observed, the skin disorder should be addressed while taking measures to deal with the complications.

If the skin disorder is ongoing, defer administration of the drug until the condition resolves.

If the skin disorder is Grade 2 or less and is controlled with corticosteroids at moderate doses (0.5 mg/kg prednisolone equivalent) or lower, consider continuation of the drug without deferral.

If Grade 3 or higher skin disorders occur, discontinue administration of the drug and take appropriate measures. Skin lesions caused by this drug may appear not only on visible areas such as the extremities, but also on the back, chest, and other parts of the body hidden by clothing. Also, since toxic epidermal necrolysis and Stevens-Johnson syndrome generally have mucocutaneous symptoms and may be preceded by mucocutaneous symptoms, the eyes, oral cavity, anus, vulva, and other skin-mucosal transition areas should also be checked.

Since severe skin disorders have been reported to occur not only during treatment with the drug but also several weeks after the end of treatment, patients should continue to be monitored regularly for their general skin condition after the end of treatment.

The recommended measures for each Grade are shown in Appendix 1.

**10-5-3. Hepatitis B virus hepatitis**

If any abnormality is observed, administration of this drug should be discontinued, and appropriate measures such as administering antiviral drugs should be taken immediately.

**10-5-4. Tumor lysis syndrome**

If any abnormality is observed, administration of this drug should be discontinued immediately, and appropriate measures (e.g., administration of saline solution, hyperuricemia treatment, dialysis, etc.) should be taken and patients should be carefully monitored until symptoms recover.

**10-5-5. Hematotoxicity and liver dysfunction**

If any abnormality is observed, appropriate measures such as postponement or discontinuation of administration of the drug should be taken.

**10-6. Measures to be taken if pregnancy is confirmed**

If a subject becomes pregnant, the investigator shall immediately discontinue administration of the investigational drug and report to the Clinical Research Coordinating Committee in accordance with the "Procedures for Handling Safety Information" separately stipulated. The Clinical Research Coordinating Committee shall immediately report to the investigational drug provider. Pregnancy itself is not considered an adverse event unless it is suspected that the investigational drug interfered with the effectiveness of the contraceptive. However, all pregnancies (including cases in which the subject discontinues the clinical trial), the outcome of pregnancy (spontaneous abortion, artificial abortion, normal delivery or delivery with congenital abnormality) shall be followed up and recorded. Birth defects, birth defects, and spontaneous abortions must be reported as serious adverse events. Uncomplicated abortions are not treated as adverse events. All pregnancy outcomes shall be reported to the Clinical Research Coordinating Committee, which shall submit them to the investigational drug provider.

**10-7. Measures in case of overdose**

If an overdose is administered to a subject, it shall be reported to the Clinical Trial Coordinating Committee in accordance with the "Procedures for Handling Safety Information" separately stipulated. The TIC shall promptly report it to the investigational drug supplier.

**11. Statistical analysis**

**11-1. Analysis population**

1) Safety Analysis Population

For each of Phase Ia and Phase Ib, the population shall consist of subjects who satisfy all of the following conditions

(1) Subjects who do not fall into the category of serious GCP non-compliance cases such as failure to obtain appropriate informed consent.

(2) Subjects who have received at least one dose of the investigational drug

2) Efficacy analysis population (Full Analysis Set)

The Full Analysis Set shall be the population of subjects who satisfy all of the following conditions for each of Phase Ia and Phase Ib.

(1) Subjects who do not fall into the category of serious GCP non-compliance cases such as failure to obtain appropriate informed consent

(2) Subjects who have received at least one dose of the investigational drug

(3) Subjects who have evaluation data after receiving the investigational drug

3) Pharmacokinetic analysis population

Subjects in Phase Ia of the safety analysis population.

**11-2. Criteria for handling subjects**

1) Ineligible cases

Cases that violate the selection criteria or the exclusion criteria will be considered as ineligible cases.

2) Cases of GCP non-compliance

Subjects who are not in compliance with GCP, such as not obtaining proper informed consent, shall be considered as GCP noncompliance cases.

3) Discontinuation or dropout

Subjects who withdrew consent or discontinued the study due to the occurrence of adverse events or other reasons judged by the investigator, etc. are considered as discontinuation cases. Subjects who stop coming to the hospital for reasons not directly related to the clinical trial are also considered as dropout cases. Subjects who have received 8 or more doses of the investigational drug will not be treated as discontinuation or dropout cases.

4) Cases of treatment violations and incomplete evaluation data

Cases of violation of dosage and administration, concomitant drug use, etc. are considered as cases of violation of treatment, and cases of incomplete evaluation data are considered as cases of incomplete evaluation data.

5) Other problem cases

For unanticipated problem cases other than 1) to 4) above, the Clinical Trial Coordinating Committee will discuss them with the investigator who conducts the clinical trial and statistical analysis experts as necessary, and decide how to handle them in terms of statistical analysis. The impact of this handling on the results will be reported in the summary report.

**11-3. Data handling standards**

If there is a discrepancy between the observation and test dates specified in the protocol and the actual observation and test dates, each item will be used only when it is measured within the following criteria. In addition, the number of days shall be 0 days from the scheduled visit date as specified.

1) PS


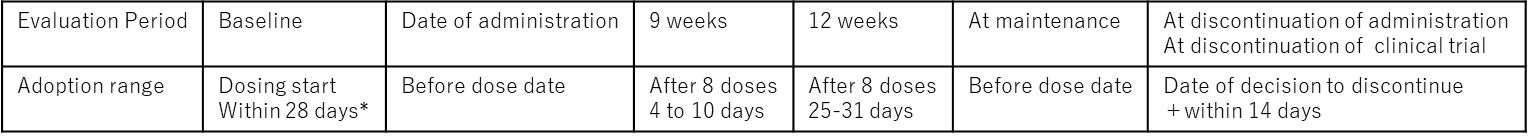
*: Same day of the week as the registration date is acceptable.


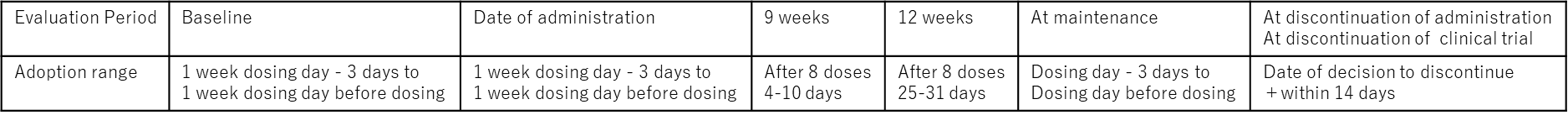
2) Body weight


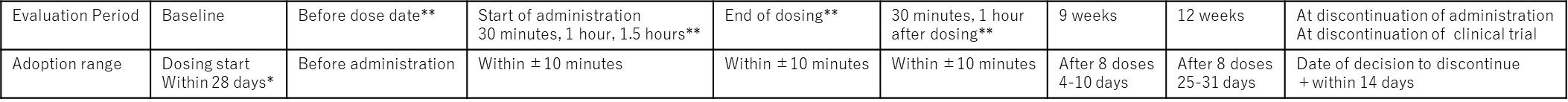
3) Vital sign

*: Same day of the week as the registration date is acceptable.

**: Including maintenance dose


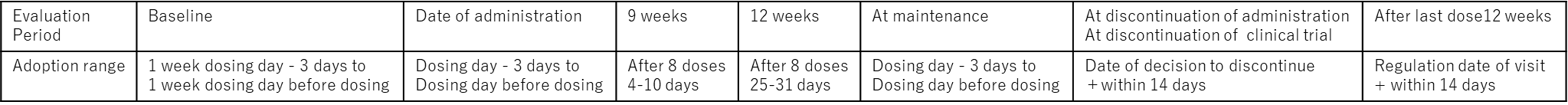
4) General blood tests (on the day of blood collection)


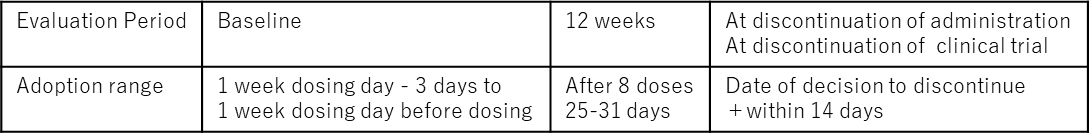
5) Thyroid function tests (on the day of blood collection)

6) Tumor marker (on the day of blood collection)


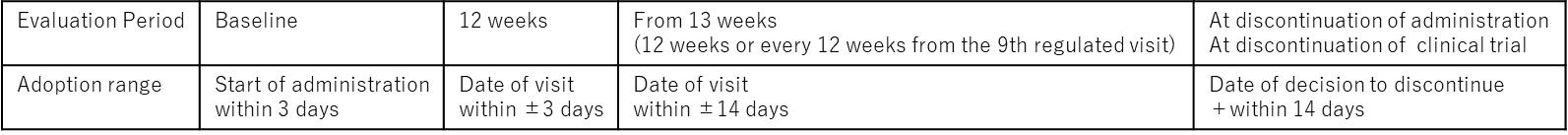


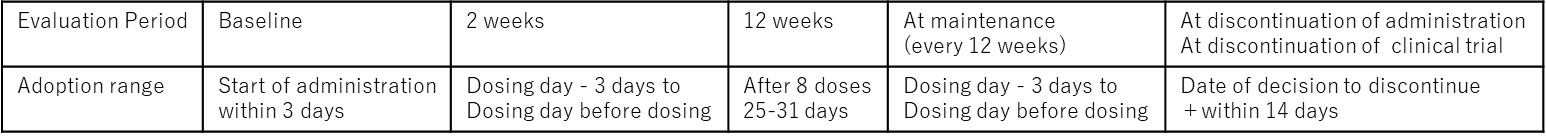
7) Urinalysis


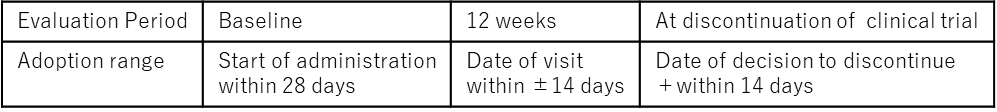
8) CT or MRI

9) Antigen-specific antibody immunoreaction test (date of blood collection)


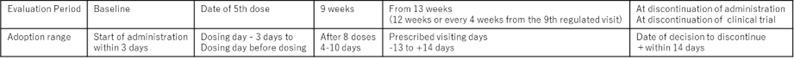


10) Regulatory T cells test (date of blood collection)


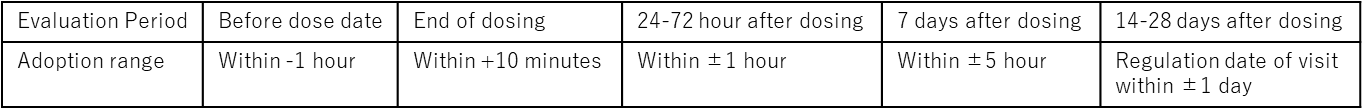

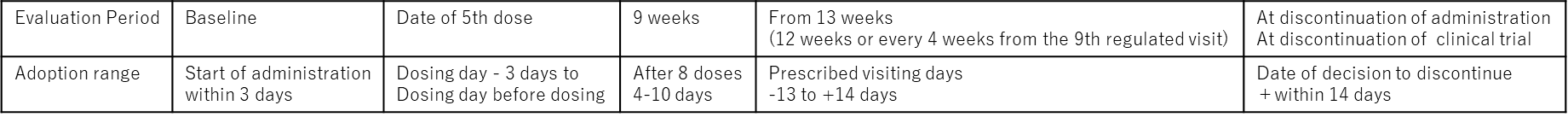


11) Pharmacokinetics


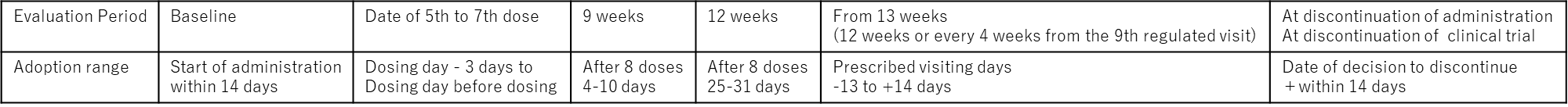
12) Immunopotentiation effectiveness test (date of blood collection)

13) Genetic analysis


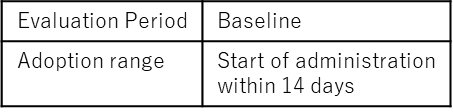


**11-4. Analysis items and methods**

Detailed analysis methods are described in the "Statistical Analysis Plan" and "Pharmacokinetic Analysis Plan" to be prepared separately.

1) Safety

(1) Number of DLT cases

The number of DLTs and the incidence rate of DLTs per treatment group in Phase Ia are shown.

(2) Occurrence of adverse events and adverse reactions

Adverse events that occurred in Phase Ia and Phase Ib are summarized in the table. All adverse events and adverse drug reactions are summarized by the MedDRA basic terms and by major organ category, and the number and percentage of adverse events for each treatment group are shown.

(3) Gene analysis

Exploratory studies will be conducted as necessary.

2) Efficacy

(1) Efficacy in eliminating regulatory T cells

Summary statistics are calculated for each treatment group.

(2) Antigen-specific antibody immune response

To be investigated in an exploratory manner for each treatment group.

(3) Anti-tumor effect

The number of responders and the percentage of responders (response rate) will be calculated for each treatment group.

(4) Progression-free survival (PFS)

Progression-free survival is calculated using the Kaplan-Meier method.

(5) Overall survival (OS)

Overall survival is calculated using the Kaplan-Meier method.

(6) Immunostimulatory effect

To be examined on an exploratory basis as necessary.

(7) Gene analysis

To be explored as necessary.

In addition, the relationship between regulatory T-cell elimination effect and anti-tumor effect, antigen-specific antibody immune response, etc. will be explored.

3) Pharmacokinetics

(1) Plasma Mogamulizumab concentration

Plasma concentrations of Mogamulizumab will be listed and descriptive statistics for each dose will be shown; plasma concentration trends of Mogamulizumab will be plotted graphically as individual subject data and mean (+ standard deviation) versus blood collection time on a real and log scale.

(2) Mogamulizumab pharmacokinetic parameters

Calculate the pharmacokinetic parameters for each subject as indicated in "8-3-1 Definition of primary endpoints 1) Phase Ia Part (2) Pharmacokinetics" and calculate descriptive statistics for each dose.

**12. Conducting genomic and genetic analyses**

**12-1. Objectives**

Genomic and genetic analyses as described in "8-3-3 Definitions of other items 1) Common to Phase Ia and Phase Ib (8) Gene analysis (optional)" will be performed using samples collected from subjects based on appropriate consent for the purpose of exploratory and basic investigations of the efficacy and safety of the investigational drug.

**12-2. Sample handling**

Samples for genomic/genetic analysis shall be blood samples collected in accordance with "9-1. Clinical Trial Schedule".

When samples collected from subjects are sent to the laboratory (see Appendix 1 "Clinical Trial Implementation System"), to ensure their anonymity, all identification of subjects will be done only by subject identification codes.

Samples should be stored under appropriate conditions in a designated laboratory under strict conditions until requested by the Clinical Trial Coordinating Committee. During the storage period, periodic checks should be made to ensure that the samples are being properly stored, and records should be kept until disposal. When disposing of the samples, they should be disposed of appropriately in accordance with the regulations of the laboratory, and a written report of the disposal of the samples should be submitted to the medical institution.

If the laboratory conducting the genomic/genetic analysis is different from the laboratory storing the samples, the procedures for sending the samples should be established and followed. Regarding the storage of samples, the samples should be stored strictly under appropriate conditions until requested by the Clinical Trial Coordinating Committee, and during the storage period, it should be periodically checked that the samples are properly stored, and records should be kept until disposal. When disposing of the samples, they should be appropriately disposed of in accordance with the regulations of the laboratory where the genomic/genetic analysis is performed, and the results should be reported in writing to the medical institution performing the genomic/genetic analysis.

**12-3. Analysis methods**

To explore genomic biomarkers that may predict the clinical response of mogamulizumab and the development of toxicity (autoimmune adverse events: skin rash, interstitial pneumonia, etc.), a genome-wide association study (GWAS) using gene polymorphism analysis and copy number analysis using DNA derived from peripheral blood mononuclear cells before the start of treatment was conducted. Genome-wide association study (GWAS) using DNA derived from peripheral blood mononuclear cells before the start of treatment and copy number analysis, and comprehensive expression analysis of miRNA in serum will be conducted.

**12-4. Consent for sample provision**

The use of samples for genome/gene analysis will be explained sufficiently in advance to the prospective subjects, and their written consent will be obtained. Points to be noted at that time are shown in "5-3. Even if a subject withdraws his/her consent to participate in a clinical trial, the samples collected up to that point may, in principle, be used for genomic/genetic analysis. The following is a brief description of the use of samples for the purpose of genome/gene analysis. Upon receiving a written request from a subject to withdraw consent for the use of a sample for genomic/genetic analysis, the sample and results pertaining to the subject shall, in principle, be disposed of after ensuring anonymity, and a written report to that effect shall be made to the subject via the implementing medical institution. However, if the test results have already been published in a paper or the like at the time of withdrawal of consent, they cannot be discarded.

**12-5. Disclosure of information to subjects**

Clinical trial information regarding the methods and results of genomic and genetic analyses conducted using samples collected from subjects (hereinafter referred to as "clinical trial information") shall be disclosed to subjects if they so request. Clinical trial information will be disclosed to subjects via the site only when the subject requests disclosure in writing.

**13. Compliance with and deviations, changes and amendments to the protocol**

The investigators shall not deviate from or change the clinical trial protocol without prior written approval from the investigational review committee.

The investigators may deviate from or change the protocol without prior approval of the Trial Review Committee for unavoidable medical reasons, such as to avoid immediate danger to the subject. In such cases, the investigator shall in such cases, the investigator shall submit the details and reasons of the deviation or change, as well as a proposal for revision of the protocol, if appropriate, to the head of the investigational institution and the investigational review committee as soon as possible for their approval.

The investigator who conducts the clinical trial shall revise the protocol as necessary when he/she becomes aware of matters related to the quality, efficacy and safety of the study drug or other information important for the proper conduct of the clinical trial.

**14. Suspension and termination of treatment**

**14-1. Suspension requirements for individual test subjects and basis for suspension**

**14-1-1. Test drug delivery and discontinuation criteria for individual test subjects**

When "7-7-3. Discontinuation Criteria" is applicable, administration of the investigational drug shall be discontinued immediately, and the investigator shall take appropriate measures, observe the clinical symptoms at the time of discontinuation, and perform general clinical examination. Furthermore, the date of discontinuation, reason for discontinuation, treatment and progress after discontinuation, etc. should be entered in the case report form. If the subject does not come to the hospital on the scheduled visit date and the investigational drug can no longer be administered, the investigator should follow up with the subject by telephone or in a sealed envelope to confirm the reason and the progress of the patient, and enter the details in the case report form.

**14-1-2. Basis for discontinuance of experimental drugs for individual subjects**

If any of the following criteria are met, not only the administration of the investigational drug will be discontinued, but also the evaluation related to the clinical trial will be discontinued.

1) When the subject withdraws consent

2) Death of the subject

3) The subject fails to show up for the scheduled visit and is unable to continue the clinical trial

4) Other cases in which the investigator determines that it is not appropriate to continue the clinical trial.

**14-1-3. Discontinuation or interruption of treatment in an actual medical institution**

In the event that the investigator finds that a violation of the GCP ordinance or the protocol by the site causes a problem with the proper conduct of the clinical trial (except for unavoidable medical reasons such as to avoid an immediate danger to the subject), the investigator shall notify the Trial Coordinating Committee in advance and discontinue the clinical trial at the site. The investigator who conducts the clinical trial himself/herself shall not conduct the clinical trial at the site. When the investigator suspends or discontinues a clinical trial, the investigator shall promptly report to that effect and the reasons in writing to the TICC through the head of the institution and the secretariat of the TICC. The Trial Coordinating Committee shall promptly notify to that effect and the reasons thereof to the person who conducts the clinical trial himself/herself at the other site through the Trial Coordinating Committee Secretariat. In the case of discontinuation of a clinical trial due to noncompliance, the site operator shall promptly report to the regulatory authority.

When the head of the site receives a report from the investigator to suspend or discontinue the clinical trial, the head of the site shall promptly notify the Investigational Review Committee in writing and explain the discontinuation or discontinuation in detail.

**14-2. Interruptions and suspensions of part and all tests**

1) Partial discontinuation of the clinical trial

If the investigator obtains information that may affect the safety of subjects, affect the implementation of the clinical trial, or change the approval of the Trial Review Committee regarding the continuation of the clinical trial, the investigator will consider suspending or discontinuing the clinical trial after discussing the matter with the Trial Coordinating Committee.

If the investigator decides to suspend or discontinue a part of the clinical trial, the investigator shall promptly report to that effect and the reasons in writing to the head of the institution and the Trial Coordinating Committee through the secretariat of the Trial Coordinating Committee. The regulatory authorities shall also be notified to that effect and the reasons thereof.

2) Discontinuation of the entire clinical trial

If the investigator receives information that may affect the safety of subjects, affect the conduct of the clinical trial, or change the approval of the Trial Review Committee for the continuation of the clinical trial, the investigator will consider suspending or terminating the entire clinical trial after consultation with the Trial Coordinating Committee.

**14-3. Completion of tests**

The clinical trial shall be terminated at the later of 1 year after the start of the last case of administration or 24 weeks after the last dose of the investigational drug.

After the clinical trial is terminated, the investigator shall notify the head of the institution in writing that the clinical trial has been terminated and report a summary of the results of the clinical trial in writing.

**15. Completion and submission of case report forms**

**15-1. Submission materials**

The investigator shall submit the following materials to the data center. The following documents shall be submitted by the investigator to the data center, and shall be applicable to all cases assigned to the registry.

1) List of reference values for laboratory tests

2) List of signatures and seals

A document indicating the signatures and seals of the investigator, sub investigator, and, if necessary, collaborator(s) who prepare, change, or revise the case report form.

3) Case report form

4) DCF (Data Clarification Form)

5) A record explaining any discrepancies with the source documents

A document in which the investigator explains the reason for any discrepancy between the data in the case report and the source documents.

6) Record of deviation from the study protocol to avoid immediate hazards (copy)

In case the investigator did not follow the protocol in order to avoid immediate danger to the subject or for other unavoidable medical reasons, a document stating this and the reasons. 15-2.

**15-2. Forms and submission deadlines**

A case report form (CRF) shall be written by the investigator at an appropriate time and submitted to the data center as soon as possible.

**15-3. How to fill in the form**

The entry should be made in pen, the date of correction should be noted, and the person making the correction should sign or affix his/her seal. In case of a major change, the reason for the change should be stated.

**15-4. Preparation and sending of case report forms**

The investigator shall fill in the necessary information on the case report form. The original copy shall be kept by the Trial Coordinating Committee. The investigator shall make and keep a copy of the original.

**15-5. Changing or amending case report forms**

Changes or amendments to the case report form shall be made in accordance with the "Procedures for Preparation, Changes or Amendments to Case Report Forms," which is separately prescribed, as follows

1) When making changes or amendments, cross out the relevant section with a double line so that the original description can be seen, write the correct information in the immediate vicinity of the section, and affix a seal or signature along with the date of the change or amendment.

2) For major changes or modifications, the reason for the change or modification should also be noted.

3) When imprinting the seal, use the same one as in the list of signature impressions.

**15-6. Issuing and submitting Data Clarification Forms (DCFs)**

When the data center inquires the investigator about questions concerning the case report form, a DCF shall be issued to the investigator. When a DCF is issued, the investigator shall promptly prepare a response to the DCF and submit it to the data center with the date of confirmation and his/her name and seal or signature. If any changes or modifications to the case report are necessary, they should be noted in the DCF so that the data after the changes or modifications are clear for each item. When the investigator prepares the response, the investigator shall inspect the entry, confirm that there are no problems, enter the date of confirmation, affix his/her name and seal or signature, and submit it to the data center. In addition, the investigator shall retain a copy of the submitted DCF.

**15-7. Discrepancies between the case report form and original documents such as medical records**

If there is any discrepancy between the case report form and the source documents such as medical records, the investigator shall prepare a "Record explaining the discrepancy with the source documents" as a record to explain the reason for the discrepancy. The investigator shall submit the "Record of Explanation of Discrepancies with Source Documents" to the data center and keep a copy.

**15-8. Handling of case report forms and data**

With regard to the handling of case report forms or laboratory report forms, or their copies, the implementing medical institution shall take the utmost care to protect personal information and ensure that information is not leaked, lost, transcribed, or copied in an unauthorized manner.

**16. Matters relating to access to source documents**

The investigator who conducts the clinical trial on his/her own and the head of the institution shall be open to inspection or direct inspection by monitors, auditors, clinical trial review committees and regulatory authorities to examine and confirm the records that must be kept. The following data and contents should be directly entered in the case report form and should be interpreted as source documents.

1) Eligibility for enrollment

2) Reason for concomitant therapy

3) Positive and negative results of general laboratory tests and the presence or absence of abnormal fluctuations

4) Grade of adverse events (including abnormal laboratory test results), causal relationship to the investigational drug, and determination of serious/non-serious

5) Evaluation of anti-tumor effect

6) Reasons for discontinuation of the investigational drug for individual subjects, treatment after discontinuation and subsequent progress, reasons for discontinuation of the investigational drug, treatment after discontinuation and subsequent progress

7) Comments

**17. Quality control and quality assurance of clinical trials**

Quality control and quality assurance of clinical trials shall be conducted in accordance with the "Standard Operating Procedures for Conducting Clinical Trials" to be separately stipulated.

**18. Ethical considerations**

**18-1. Rules and regulations to be observed**

This clinical trial will be conducted in accordance with the "Declaration of Helsinki," this protocol, the standards stipulated in Article 14, Paragraph 3 and Article 80-2 of the Pharmaceutical Affairs Law, and the "Ministerial Ordinance Concerning Standards for Conducting Clinical Studies on Pharmaceutical Products (GCP)" (Ministry of Health and Welfare Ordinance No. 28).

**18-2. Clinical trial review committees**

Prior to the implementation of this clinical trial, the investigational review committee at each site shall review the protocol, the methods used to obtain subject consent, the investigational new drug summary, and other necessary documents to determine whether this clinical trial is ethically and scientifically appropriate and whether it is otherwise appropriate for the site to conduct this clinical trial. The investigators will review whether the study is ethically and scientifically appropriate and whether the study is appropriate to be conducted at the site.

Continuation Review

1) The investigator shall submit a summary of the current status of the clinical trial in writing to the head of the site once a year, or more frequently if requested by the Investigational Review Committee, in order to receive continuing review by the Investigational Review Committee.

2) The head of the site shall submit a report on the current status of the clinical trial at the site, a notice of serious and unpredictable side effects from the site operator, a notice of serious adverse events from the investigator, and information that may affect the subject's decision to continue to participate in the clinical trial, to the site director. (2) When the investigator receives information that may affect the subject's decision to continue to participate in the clinical trial, the investigator shall request the opinion of the Clinical Trial Review Committee regarding the continuation of the said clinical trial.

**18-3. Informed consent**

The investigator or other relevant personnel shall fully explain the clinical trial to the subject and obtain his/her written consent. Provide the subject with an explanatory document that details the clinical trial for the subject to read carefully and retain. After giving the subject time to fully consider the information and to answer any questions, the consent document should be signed and dated.

**18-4. Provision of new information**

If the investigator obtains information that may adversely affect the safety of subjects, affect the conduct of the clinical trial, or change the approval of the Investigational Review Committee, or information on serious and unpredictable adverse drug reactions, the investigator will promptly report such information to the Investigational Review Committee in writing. If the investigator who conducts the clinical trial considers it necessary to revise the consent document and other explanatory documents, the investigator shall do so promptly.

**18-5. Ensuring confidentiality and privacy of subjects**

(1) Subjects shall be identified in the subject registration and case report forms by the person in charge of data and sample management, and shall be anonymized by means of a subject identification code or other linkable anonymization method. Careful consideration will be given to the protection of subjects' privacy when directly viewing and handling source documents. In the same way, the subject samples will be made linkable anonymized with a subject identification code, etc., and will be identified with this subject identification code, etc., when the samples are transferred to other facilities, etc.

**19. Preservation of essential documents**

**19-1. Self-initiated clinical trial provider**

In accordance with the "Procedures for Preservation of Records," which will be separately stipulated, the person conducting the clinical trial shall appropriately preserve the records related to this clinical trial until the later date of either 1) or 2) below. The investigator may request the head of the institution to which the investigator belongs to perform the preservation of the records. In the event that the person conducting the clinical trial no longer belongs to the institution to which he/she belongs, the head of the institution to which he/she belongs may be responsible for the preservation of said records.

If the Trial Coordinating Committee determines that these materials need to be stored for a longer period of time, the head of the site may discuss the storage period and storage method with the Trial Coordinating Committee.

When the need for storage is no longer necessary, the investigator who conducts the clinical trial himself/herself must notify the head of the site or the establisher of the investigational review committee via the head of the site to that effect.

1) The date of marketing authorization for the investigational drug concerned (in the case of notification of discontinuation of development or notification that the clinical trial results will not be attached to the application for approval pursuant to Article 26-10, Paragraphs 2 and 3 of the GCP Ordinance, the date on which three years have passed since the decision to discontinue development was made or notification was received that the results will not be attached to the application)

2) The date on which three years have elapsed since the discontinuation or termination of the clinical trial.

**19-2. The conducting medical institution**

Required documents to be kept by the head of the implementing medical institution or the establisher of the clinical trial review committee shall be appropriately kept by the person in charge of record keeping designated by the head of the implementing medical institution. The retention period shall be until the later of the following 1) or 2).

1) The date of marketing approval for the investigational drug (in the case of notification of discontinuation of development or that the clinical trial results will not be attached to the application for approval pursuant to Article 26-10, Paragraphs 2 and 3 of the GCP Ordinance, the date three years have passed since the decision to discontinue development was made or notification was received that the results will not be attached to the application).

2) The date on which three years have elapsed since the discontinuation or termination of this clinical trial.

**19-3. Principal investigator**

The investigator shall preserve documents related to the clinical trial in accordance with the instructions of the head of the implementing medical institution. 20.

**20. Monetary payments and insurance**

**20-1. Payment of money**

Each site will decide whether or not to pay the burden reduction expenses related to this clinical trial for the subject's visit to the site for the clinical trial after case enrollment, in accordance with the agreement of each site.

**20-2. Compensation**

If any health hazard is recognized in this clinical trial, appropriate diagnosis and treatment will be promptly provided within the scope of medical health insurance. However, no compensation will be paid for any health problems resulting from this clinical trial.

**21. Sources of funding and possible conflicts of interest**

Mogamulizumab (anti-CCR4 antibody) will be provided free of charge by Kyowa Hakko Kirin Co. Other costs will be paid by public research funds.

**22. Attribution of test results and publication of results**

**22-1. Publication of results**

After the completion of the study, the results will be summarized and published in appropriate domestic and international academic societies and English journals. 22-2.

**22-2. Preparation of a comprehensive report**

When the data up to one year from the start of administration of the final patient are fixed and after the completion of the trial, the investigator shall prepare a summary report in accordance with the "Procedures for Preparation of Summary Report" separately stipulated.

**22-3. Provision of data**

After the completion of the study, data of the study, excluding personal information, may be provided for a fee or free of charge in accordance with the instructions and guidance of the regulatory authorities or at the request of related companies, etc.

**22-4. Secondary use of data**

If the Clinical Trial Coordinating Committee determines that secondary use of the data obtained in this study would be beneficial, the data may be used for secondary purposes, paying close attention to the protection of personal information.

**23. References**

1) Cancer Research Foundation. Cancer Statistics 2005.

2）The Japan Society for Esophageal Disease．Comprehensive Registry of Esophageal Cancer in Japan（1998、1999）and long-term results of esophagectomy in Japan（1988-1997）3rd Edition

3）Dunn GP, Bruce AT, Ikeda H, Old LJ, Schreiber RD. Cancer immunoediting: from immunosurveillance to tumor escape. Nat Immunol. 2002;3:991-8.

4）Wada H, Sato E, Uenaka A, et al. Analysis of peripheral and local anti-tumor immune response in esophageal cancer patients after NY-ESO-1 protein vaccination. Int J Cancer. 2008;123:2362-9.

5）Hodi S, O’Day SJ, McDermott DF, et al. Improved survival with ipilimumab in patients with metastatic melanoma. N Engl J Med. 2010; 363: 711-723.

6）Yuan J, Adamow M, Ginsberg BA, et al. Integrated NY-ESO-1 antibody and CD8+ T-cell responses correlate with clinical benefit in advanced melanoma patients treated with ipilimumab. Proc Natl Acad Sci U S A. 2011;108:16723-8.

7）Onizuka S, Tawara I, Shimizu J, et al. Tumor rejection by in vivo administration of anti-CD25 (interleukin-2 receptor alpha) monoclonal antibody. Cancer Res. 1999;59:3128-33.

8）Miyara M, Sakaguchi S.Human. FoxP3(+)CD4(+) regulatory T cells: their knowns and unknowns. Immunol Cell Biol. 2011 ;8:346-51.

9）Ishida T, Ueda R. Antibody therapy for Adult T-cell leukemia-lymphoma. Int J Hematol. 2011;94:443-52.

10）Yamamoto K, Utsunomiya A, Tobinai K, et al. Phase I study of KW-0761, a defucosylated humanized anti-CCR4 antibody, in relapsed patients with adult T-cell leukemia-lymphoma and peripheral T-cell lymphoma. J Clin Oncol 2010; 28: 1591-1598.

11）Ishida T, Joh T, Uike N, et al. Defucosylated anti-CCR4 monoclonal antibpdy (KW-0761) for relapsed adult T-cell leukemia-lymphoma: a multicenter phase II study. J Clin Oncol 2012; 30: 837-842.

12）E.A. Eisenhauer, P. Therasse, J. Bogaerts, et al. New response evaluation criteria in solid tumours:Revised RECIST guideline (version 1.1). European Journal of Cancer. 2009;45:228-247.

13）Japan Clinical Oncology Group. Common Terminology Criteria for Adverse Events v4.0 Japanese translation JCOG version - December 17, 2011.
