# Supplementary material for: Integrated analysis of phase 1a and 1b randomized controlled trials; Treg-targeted cancer immunotherapy with the humanized anti-CCR4 antibody, KW-0761, for advanced solid tumors
Source: PLoS One. 2023 Sep 20;18(9):e0291772. doi: 10.1371/journal.pone.0291772 (PMC10511099; doi:10.1371/journal.pone.0291772)
Supplement: S1 File — (PDF) [file pone.0291772.s009.pdf]

進行又は再発固形がん患者に対する Mogamulizumab の第 Ia/Ib 相多施設共同医師主導治験

治験実施計画書

治験実施計画書番号：KW0761-IIT-01
版　　　　　　　数：第 6.0 版
作　成　年　月　日：2015 年 2 月 23 日作成

秘密の保全に関する記述

本治験実施計画書は、治験実施医療機関の長、治験分担医師、治験協力者、治験薬管理者及び治験審査委員会等の本治験の関係者にのみ提供されるものです。本治験実施計画書の記載内容については、第三者に漏洩することなく秘密情報としてお取り扱い頂きますようお願い致します。

治験実施計画書の要約

1. 治験の目的

進行再発がん患者に対して Mogamulizumab（抗 CCR4 抗体）を週 1 回反復投与した際の安全性及び薬物動態を検討すること（第Ⅰa 相部）、及び週 1 回反復投与した際の安全性及び制御性 T 細胞除去効果を検討すること（第Ⅰb 相部）を目的とする。

2. 評価項目

2-1. 主要評価項目

2-1-1. 第Ⅰa 相部

1) 安全性：最大耐用量（MTD）、用量制限毒性（DLT）、有害事象の種類・頻度・程度
2) 薬物動態の検討

2-1-2. 第Ⅰb 相部

1) 安全性：有害事象の種類・頻度・程度
2) 制御性 T 細胞除去効果

2-2. 副次的評価項目

2-2-1. 第Ⅰa 相部

1) 制御性 T 細胞除去効果
2) 有効性：腫瘍縮小効果、無増悪生存期間（PFS）、全生存期間（OS）

2-2-2. 第Ⅰb 相部

1) 有効性：腫瘍縮小効果、無増悪生存期間(PFS)、全生存期間(OS)
2) 第Ⅱ相試験以降の推奨投与量の決定

3. 対象

進行又は再発固形がん患者

3-1. 被験者の選択基準

進行又は再発固形がん患者で、登録時に以下の基準を全て満たす患者を適格とする。

1) 腫瘍細胞の CCR4 発現が陰性（診断は本邦で商品化されている方法を用いること）であり、且つ病理組織診断で肺がん、胃がん、食道がん、悪性黒色腫、卵巣がん等の悪性腫瘍の診断が確定している患者
2) 標準治療法に不応・不耐性の患者、適切な治療法がない患者、又は標準治療を拒否した患者
3) performance status（ECOG 基準）が 0、1、2 であること
4) 治験参加同意取得日の年齢が 20 歳以上であること
5) 主要臓器（骨髄、心、肺、肝、腎等）に高度な障害がなく臨床検査値（登録前 2 週間以内）

が以下の基準を満たすこと

好中球数：	1,500/μL 以上
ヘモグロビン値：	8.0g/dL 以上
血小板数：	75,000/μL 以上
血清総ビリルビン値：	2.0 mg/dL 以下
AST(GOT)、ALT(GPT)値：	施設基準値上限×2.5 以下（原疾患による肝浸潤に起因すると判断される場合においては、施設基準値上限×5.0 以下）
血清クレアチニン：	1.5 mg/dL 以下
動脈血酸素飽和度：	93%以上（室温）
心電図：	治療を要する異常所見を認めない
左心駆出率（心エコー検査による）：	50%以上

6) 同意取得時から治験薬投与終了後 24 週まで、コンドーム等の避妊方法による避妊に同意していること（閉経後（最終月経から 1 年以上経過）又は外科的不妊手術を施行した女性及び外科的避妊術を施行した男性は除く））

7) 本人が文書により同意していること

8) Mogamulizumab の初回投与前から初回投与翌日まで投与入院が可能な患者

9) 効果判定の対象になる RECIST ver.1.1（New response evaluation criteria in solid tumours:Revised RECIST guideline (version 1.1).）[12)] における測定可能病変を有すること

10) 3 カ月間以上の生存が期待できること

3-2. 被験者の除外基準

登録時に以下のいずれかの基準に該当する患者は除外する。

1) HIV 抗体陽性例

2) HCV 抗体陽性例

3) 自己免疫疾患例

4) HBs 抗原が陽性、もしくは HBV-DNA がリアルタイム PCR 法により「検出」*と報告された患者

* HBV-DNA 検査については HBc 抗体検査又は HBs 抗体検査結果が陽性の場合に実施する。

5) 抗体製剤の投与により重篤な過敏症の既往歴を有する患者

6) 重複がんを有する患者。ただし、重複がんとは、同時性重複がん及び無病期間が 5 年以内の異時性重複がんである。局所治療により治癒と判断される Carcinoma in situ （上皮内がん）もしくは粘膜内がん相当の病変は重複がんに含めないこととする。

7) 抗がん剤、副腎皮質ステロイド剤の持続全身投与、免疫抑制剤及び免疫強化剤の投与、サイトカイン療法、放射線療法及び原疾患に対する手術の施行から治験薬投与開始予定日まで 4 週以上経過していない場合

8) 妊娠中、授乳中、あるいは妊娠の可能性のある患者

9) 活動性の感染症を合併している患者

10) 精神障害又は認知症患者

11) 全身性のステロイド剤投与による治療の継続が必要な患者

12) 造血幹細胞移植等の移植療法が実施された患者

13) 中枢神経腫瘍浸潤を有する患者又はそれを疑わせる臨床所見を有する患者

14) 症例登録 4 週間以内に他の治験薬を投与された患者

15) 症例登録 12 週間以内に腫瘍に対する免疫療法(腫瘍ワクチン等)が実施された患者

16) その他治験遂行に不適当と考えられる患者

4. 治験デザイン

シェーマ

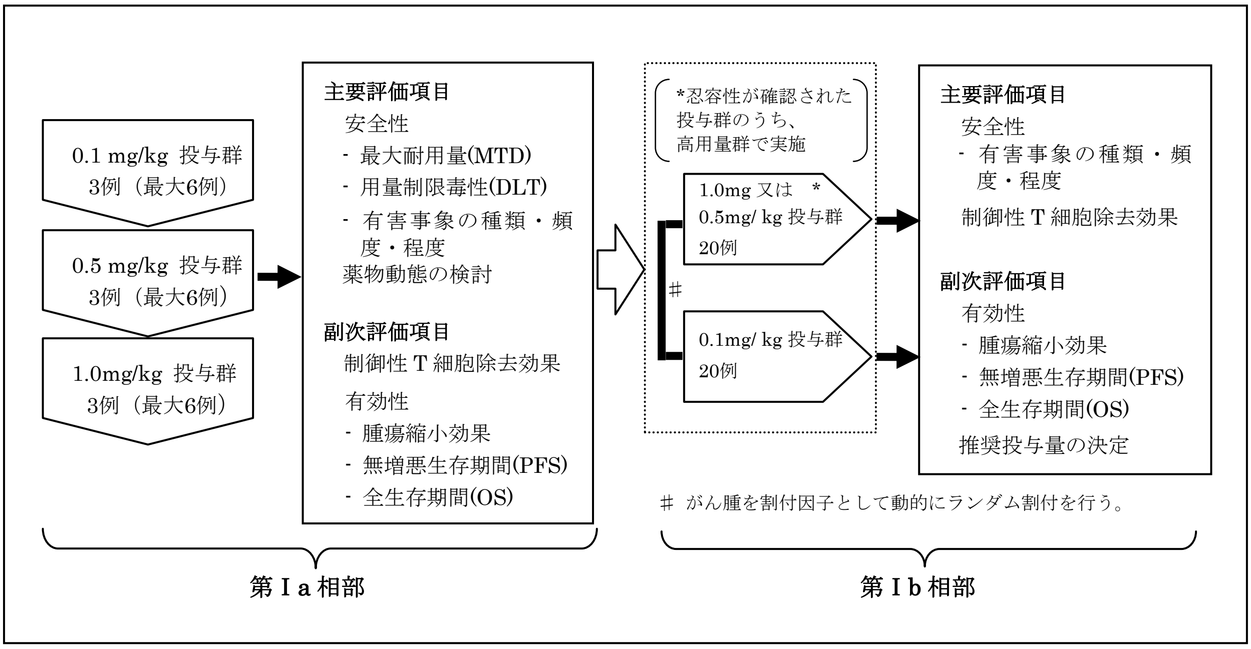

【第Ｉa 相部】0.1mg/ kg からスタートし、忍容性が確認されれば 0.5mg/ kg 及び 1.0mg/ kg へ dose escalation。

【第Ｉb 相部】忍容性が確認された投与群のうち高用量群及び 0.1mg/ kg の 2 群（第Ｉa 相部で 0.5mg/ kg が MTD の場合、0.1mg/ kg 1 群）で実施。各群 20 例となるように登録。

【忍容性の判定基準】MTD の一段階下の投与量を「忍容性が確認された投与量」とする。ただし、いずれの投与量でも MTD に達しなかった場合は 1.0mg/kg を忍容性が確認された投与量とする。MTD は、治験薬投与開始 28 日後までの期間における DLT 発現被験者数が 3 名以上に認められた投与量とする。

5. 投与方法

投与する際は、「7-7-1.投与基準」を満たしており、且つ「7-7-2.投与延期基準」及び「7-7-3.投与中止基準」に抵触していないことを確認する。

【第Ｉa 相部】0.1mg/kg、0.5mg/kg、1.0mg/kg のいずれかの投与量を 1 週間に 1 回 2 時間かけて静脈内投与し、計 8 回投与する。

【第Ｉb 相部】第Ｉa 相部で 1.0mg/kg までの忍容性が確認された場合は 0.1mg/kg 又は 1.0mg の

いずれか、0.5mg/kg までの忍容性が確認された場合は 0.1mg/kg 又は 0.5mg/kg のいずれか、0.1mg/kg までの忍容性が確認された場合は 0.1mg/kg を 1 週間に 1 回 2 時間かけて静脈内投与し、計 8 回投与する。

【第Ｉa 相部・第Ｉb 相部共通】

1) 維持投与

9 回目以降の投与は、「7-8.維持投与の実施基準」を満たした場合に実施可能とし、4 週毎に治験薬を維持投与する。8 回投与までと同様に、投与する際は、「7-7-1.投与基準」を満たしており、且つ「7-7-2.投与延期基準」及び「7-7-3.投与中止基準」に抵触していないことを確認する。

2) 投与間隔

8 回投与までは投与間隔の短縮は行わない。ただし、祝祭日又は休院日によって投与を 1 日延期した場合のみスケジュール調整のため次回投与を 1 日短縮することは可能とする。9 回投与以降は、最大 2 週間までの投与延期を可とし、次回投与を最大 2 週間短縮することは可能とする。

3) 投与時の前処置

注入に伴う反応はほぼ全例に見られることが想定されるため、治験薬の投与 30 分前に、ジフェンヒドラミン 30-50mg 及びアセトアミノフェン 300-500mg を内服する。また、ハイドロコーチゾン 100mg を静脈内に前投与する。2 回目の投与以降はジフェンヒドラミン 30-50mg 及びアセトアミノフェン 300-500mg の前投与を行う。投与薬剤あるいは投与量については年齢、症状等により適宜、変更、増減する。

6. 前治療及び併用療法

治験開始前 4 週間以内に対象疾患に対して前治療（化学療法、放射線療法等）が行われていないことを確認する。腫瘍に対する免疫療法（腫瘍ワクチン等）に関しては症例登録前 12 週間以内に行われていないことを確認する。除外基準に抵触しないことを確認する。

抗がん剤、免疫抑制剤（ステロイド剤を除く）、免疫強化剤、放射線療法、原疾患（転移した腫瘍を含む）に対する手術は治験薬投与期間中、併用禁止とする。副腎皮質ステロイド剤は、治験薬投与のための前処置、合併症及び有害事象の症状改善のための局所投与又は全身投与を許容する。

骨転移例に対するゾレドロン酸やパミドロネートの静脈内点滴、及びデノスマブの皮下注射については、本治験参加前から継続していた場合は、4 週に 1 回の投与であれば併用可とする。ただし、治験薬と同日の投与は避けることとする。

なお、以下のワクチンについては併用に注意する。

１．不活化ワクチン［ワクチンの効果を減弱させる恐れがある（ワクチン接種に対する応答が不明である）］。

２．生ワクチン又は弱毒生ワクチン［接種した生ワクチンの原病に基づく症状が発現した場合には適切な処置を行う（ワクチン接種に対する応答が不明であり、また、生ワクチンによる二次感染が否定できない）］。

その他合併症及び有害事象に対する治療は可能とする。

未承認医薬品や代替療法（民間療法等）は禁止するが、食品に準じたサプリメントの使用は禁止しない。

7. 治験スケジュール

次のスケジュール表 1 に従い、所定の観察・検査を実施する。なお、初回投与前から投与翌日まで入院する。

治験責任医師等は第 Ia 相部の被験者については 5 回目の投与に先立ち、治験の継続について再度同意を得ることとする。

8 回投与後に制御性 T 細胞除去効果及び抗原特異的抗体免疫反応又は RECIST において SD 以上が確認された被験者については、被験者に治験薬の維持投与の希望があり、且つ治験責任医師等が治験薬の維持投与が適当と判断した場合は、治験薬の維持投与を可能とする。

初回の治験薬投与日を 1 週と定義する。

表 1. 治験スケジュール

	ベースライン	1 週 d1 入院 ⟷	2 週 d8	3 週 d15	4 週 d22	5 週 d29	6 週 d36	7 週 d43	8 週 d50	9 週 d57	10 週 d64	12 週 d78	13 週以降 (12 週又は 9 回目規定来院日から n 週毎)	投与中止時	治験中止時
同意取得	●														
Mogamulizumab*1 (投与回数)		● (1)	● (2)	● (3)	● (4)	● (5)	● (6)	● (7)	● (8)			○ (9)	○ 4 週毎		
再同意取得						●									
問診*2	●	●	●	●	●	●	●	●	●	●		●	○ 4 週毎	●	●
PS*3	●	●	●	●	●	●	●	●	●	●		●	○ 4 週毎	●	●
体重*3	●		●	●	●	●	●	●	●	●		●	○ 4 週毎	●	●
Vital check*4 (血圧・脈拍数、体温、呼吸数)	●	●	●	●	●	●	●	●	●	●		●	○ 4 週毎	●	●
一般血液検査*3*5*6	●		●	●	●	●	●	●	●	●		●	○ 4 週毎	●	●*11
甲状腺機能検査*3*5*	●											●		●	●
腫瘍マーカー*3*5（任意）	●											●	● 12 週毎		
尿検査*3	●		●									●	○ 12 週毎	●	●
CT 又は MRI	●											●	● 12 週毎		●*11
腫瘍抗原発現検査 (NY-ESO-1、XAGE-1b)	●														
HIV、HCV、HBV 検査	●														
CCR4 発現検査	●														
抗原特異的抗体免疫反応検査*3*5 (採血量 mL)	● (5)					● (5)				● (5)			● 4 週毎 (5)	● (5)	●*11 (5)
制御性 T 細胞検査*3*5 (採血量 mL)	● (10)					● (10)				● (10)			● 4 週毎 (10)	● (10)	●*11 (10)
薬物動態検査*5*7 (採血量 mL)		● (2×4)	● (2)			● (2)	● (2)		● (2×4)	● (2)	● (2)	● (2)			
免疫増強効果検査*3*5*8 （任意）（採血量 mL）	●*9 (50×2)					● (30)		● (40)		● (30)		● (40)	● 4 週毎 (50)	● (50)	●*11 (50)
遺伝子解析*3*5（任意） (採血量 mL)	● (15)														
有害事象*10		◀	━	━	━	━	━	━	━	━	━	━	━	━	▶

○：8 回投与後に維持投与が行われた際に追加で実施する項目

*1：9 回目以降の治験薬の投与は、制御性 T 細胞除去効果及び抗原特異的抗体免疫反応又は RECIST において SD 以上が確認され、被験者に治験薬の維持投与の希望があり、且つ治験責任医師等が治験薬の維持投与が適当と判断した場合に限る。

*2：治験薬投与開始前～終了まで継続的に観察する。

*3：治験薬投与期間中は治験薬投与前に実施する。

*4：治験薬投与前、投与開始 30 分、1 時間、1.5 時間、投与終了時、投与終了後 30 分後、1 時間後に実施する。

*5：貧血の兆候が認められた場合等で規定量の採血が困難な場合は、被験者の安全性を考慮し、1.一般血液検査(施設の規定量)、2.甲状腺機能検査（施設の規定量）、3.制御性 T 細胞検査(10mL)、4.薬物動態検査(第 I a 相部のみ、2mL)、5.抗原特異的抗体免疫反応検査(5mL)、6.遺伝子解析(15mL)、7.免疫増強効果検査(30~50mL)、8.腫瘍マーカー(施設の規定量)の優先順位で可能な範囲の採血を行う。

*6：治験薬最終投与後 12 週目にも検査を実施する。

*7：1 回あたりの採血量は 2mL とする。実施時期については表 2 を参照のこと。

*8：「8-3-3 その他の項目の定義」（2）、（4）～（6）に示した検査項目とする。

*9：複数日に分けて採血を行うことを可とする。

*10：有害事象は治験薬の最終投与から 12 週後まで観察する。ただし、Grade3 以上の非血液毒性に関しては 24 週後までを有害事象の観察期間とする。

*11：8 回投与後に維持投与が行われず、12 週の検査実施後に治験中止した場合、治験中止時には一般血液検査、CT 又は MRI、抗原特異的抗体免疫反応検査、制御性 T 細胞検査及び免疫増強効果検査のみ実施する。

表 2. 薬物動態検査の採血スケジュール（第 Ia 相部のみ実施）

投与回数／採血時期	初回投与	2 回目	3 回目	4 回目	5 回目	6 回目	7 回目	8 回目
投与前	●	●				●		●
投与終了時／投与中止時	●				●			●
24 時間後（1 日後）	●							●
72 時間後（3 日後）	●							●
168 時間後（7 日後）								●
14 日後								●
28 日後								●

8. 投与中止基準

以下のいずれかの規定に該当する場合は、当該被験者の投与を中止する。

1) 投与延期基準により、規定の投与予定日より 2 週間を超えて投与延期となった場合
2) 24 週目以降において「RECIST の変法の評価において総合効果が PD」であった場合
3) 治験責任医師等により治験の継続が困難と判断される有害事象が発現した場合
4) 被験者が治験薬の投与中止を申し出た場合
5) 不適格例であることが判明した場合
6) その他、治験責任医師等が中止すべきと判断した場合

9. 予定登録症例数

9-1. 第 I a 相部

0.1mg/kg 群　3 例（最大 6 例）

0.5mg/kg 群　3 例（最大 6 例）

1.0mg/kg 群　3 例（最大 6 例）

計 9 例（最大 18 例）

9-2. 第 I b 相部

1 群で実施する場合は 20 例

2 群で実施する場合は各群 20 例、計 40 例

10. 治験実施予定期間

2013 年 2 月～2016 年 3 月

略号一覧表

略号	英名	和名
5-FU	5-Fluorouracil	5-フルオロウラシル
ADCC	Antibody Dependent Cellular Cytotoxicity	抗体依存性細胞傷害
ADP	Adenosine Diphosphate	アデノシンニリン酸
ALP	Alkaline Phosphatase	アルカリフォスファターゼ
ALT	Alanine Aminotransferase	アラニンアミノトランスフェラーゼ
AST	Aspartate Aminotransferase	アスパラギン酸アミノトランスフェラーゼ
ATL	Adult T-cell Leukemia	成人T細胞白血病リンパ腫
$AUC_{0\text{-}7d}$	Area Under the Curve 0-7day	0-7日目までの血漿中薬物濃度、時間曲線下面積
CBDCA	cis-Diammine(1,1-cyclobutanedicarboxylato)-Platinum(II), Carboplatin	カルボプラチン
CCR4	Chemokine (C-C motif) Receptor 4	CC ケモカイン受容体 4
CD	Cluster of Differentiation	白血球分化抗原
CDDP	cis-Diamminedichloro-platinum(II), Cisplatin	シスプラチン
CL	Clearance	クリアランス
C_{max}	Maximum Concentration	最高血漿中薬物濃度
CPT-11	Camptothecin 11, Irinotecan Hydrochloride	イリノテカン
CR	Complete Response	完全奏功
C_{trough}	Trough Concentration	トラフ濃度
CT	Cancer-testis	がん精巣
CT	Computer Tomography	コンピューター断層造影撮影
CTCAE	Common Terminology Criteria for Adverse Events	有害事象共通用語基準
CTL	Cytotoxic T Lymphocyte	細胞傷害性 T 細胞
CTLA4	Cytotoxic T Lymphocyte Antigen 4	細胞傷害性 T リンパ球抗原 4
DCF	Data Clalification Form	データクラリフィケーションフォーム
DLT	Dose Limiting Toxicity	用量制限毒性
DTIC	Dimethyl Triazeno Imidazole Carboxamide	ダカルバジン
ECOG	Eastern Cooperative Oncology Group	（米国）東部腫瘍共同研究グループ
ELISA	Enzyme-linked Immuno Sorbent Assay	固相酵素免疫検定法
FCM	Flow Cytometry	フローサイトメトリー
FT3	Free Triiodothyronine	遊離トリヨードサイロニン
FT4	Free Thyroxine	遊離サイロキシン
GCP	Good Clinical Practice	医薬品の臨床試験の実施に関する基準
G-CSF	Granulocyte Colony Stimulating Factor	顆粒球コロニー刺激因子
γ-GTP	Gamma-Glutamyl Transpeptidase	γ-グルタミルトランスペプチダーゼ

略号一覧表

略号	英名	和名
GMP	Good Manufacturing Practice	医薬品及び医薬部外品の製造管理及び品質管理規則
GOT	Glutamic Oxaloacetic Transaminase	グルタミン酸オキサロ酢酸トランスアミナーゼ
GPT	Glutamic Pyruvic Transaminase	グルタミン酸ピルビン酸トランスアミナーゼ
GVHD	Graft-Versus-Host Disease	移植片対宿主病
HBc	Hepatitis B Core	B 型肝炎コア
HBs	Hepatitis B Surface	B 型肝炎表面
HBV-DNA	Hepatitis B Virus Deoxyribonucleotide	B 型肝炎ウイルス-遺伝子（DNA）
hCG	Human Chorionic Gonadotrophin	ヒト絨毛性ゴナドトロピン
HCV	Hepatitis C Virus	C 型肝炎ウイルス
HIV	Human Immunodeficiency Virus	ヒト免疫不全ウイルス
HLA	Human Leukocyte Antigen	ヒト白血球型抗原
IFN-γ	Interferon-gamma	インターフェロン-γ
IgG	Immunogloburin G	免疫グロブリン G
IHC	Immunohistochemistry	免疫組織化学
LDH	Lactase Dehydrogenase	乳酸脱水素酵素
MAGE-A4	Melanoma-Associated Antigen 4	メラノーマ関連抗原 4
MDSC	Myeloid-derived Suppressor Cell	ミエロイド由来免疫抑制細胞
MedDRA	Medical Dictionary for Regulatory Activities	ICH 国際医薬用語集
MF	Mycosis Fungoides	菌状息肉症
miRNA	micro-RNA	マイクロ RNA
MRI	Magnetic Resonance Imaging	磁気共鳴映像法
MRT	Mean Residence Time	平均滞留時間
MTD	Maximum-tolerated Dose	最大耐用量
OS	Overall Survival	全生存期間
PCR	Polymerase Chain Reaction	ポリメラーゼ連鎖反応法
PD	Progressive Disease	病勢の進行
PFS	Progression Free Survival	無増悪生存期間
PR	Partial Response	部分奏効
PS	Performance Status	活動指標
PTCL	Peripheral T-cell Lymphoma	末梢性 T 細胞リンパ腫
PTX	Paclitaxel	パクリタキセル
RECIST	Response Evaluation Criteria in Solid Tumors	固形がんの治療効果判定のためのガイドライン
SD	Standard Deviation	標準偏差
SD	Stable Disease	安定した病状

略号一覧表

略号	英名	和名
$t_{1/2}$	Half Life	血中消失半減期
TARC	Thymus and Activation-regulated Chemokine	胸腺及び活性化制御ケモカイン
TLS	Tumor Lysis Syndrome	腫瘍融解症候群
TNF-α	Tumor Necrosis Factor-alpha	腫瘍壊死因子-α
TSH	Thyroid Stimulating Hormone	甲状腺刺激ホルモン
Vss	Volume of Distribution at Steady State	定常状態での分布容積

目次

1. 治験の目的 i
2. 評価項目 i
2-1. 主要評価項目 i
2-1-1. 第Ｉa 相部 i
2-1-2. 第Ｉb 相部 i
2-2. 副次的評価項目 i
2-2-1. 第Ｉa 相部 i
2-2-2. 第Ｉb 相部 i
3. 対象 i
3-1. 被験者の選択基準 i
3-2. 被験者の除外基準 ii
4. 治験デザイン iii
5. 投与方法 iii
6. 前治療及び併用療法 iv
7. 治験スケジュール v
8. 投与中止基準 vii
9. 予定登録症例数 vii
9-1. 第Ｉa 相部 vii
9-2. 第Ｉb 相部 vii
10. 治験実施予定期間 vii
1. 開発の背景 1
1-1. 進行又は再発、食道がん、胃がん、肺がん、悪性黒色腫、卵巣がんとその治療 1
1-2. 腫瘍免疫 1
1-2-1. 免疫の抗腫瘍効果 1
1-2-2. 腫瘍免疫の臨床応用 2
1-3. Mogamulizumab について 3
1-3-1. 非臨床試験データの概要 3
1-3-2. 臨床試験データの概要 5
1-4. 副作用 6
1-5. まとめ 7
2. 治験の実施体制と役割 7
3. 治験の目的 7
4. 対象 7
4-1. 被験者の選択基準 7
4-2. 被験者の除外基準 8
5. 被験者の同意取得 9
5-1. 同意文書及びその他の説明文書 9
5-2. 同意取得の時期、方法及びその内容 9

5-2-1. 治験開始時の同意取得 9
5-2-2. 第Ｉa 相部の 5 回目以降の治験薬投与に関する同意取得 11
5-3. 同意に関する留意事項 11
5-4. 同意文書及びその他の説明文書の改訂..12
6. 治験薬 12
6-1. 名称 12
6-2. 剤型及び成分..12
6-3. 分子式及び分子量 13
6-4. 貯法 13
6-5. 有効期間..13
6-6. 治験薬の調製..13
6-7. 治験薬の包装及び表示 13
6-8. 治験薬の提供..13
6-9. 治験薬の管理と未使用治験薬の返却 13
7. 治験方法 14
7-1. 治験デザイン..14
7-2. 第Ｉa 相部における群間移行及び手順 15
7-3. 第Ｉb 相部への移行..16
7-4. 治験実施予定期間 16
7-5. 登録方法と割り付け 16
7-5-1. 被験者の登録の手順..16
7-5-2. 割り付け 17
7-6. 予定登録症例数..17
7-6-1. 第Ｉa 相部 17
7-6-2. 第Ｉb 相部 17
7-7. 投与方法..17
7-7-1. 投与基準 18
7-7-2. 投与延期基準..18
7-7-3. 投与中止基準..19
7-8. 維持投与の実施基準..19
7-9. 前治療及び併用療法..19
7-10. 後治療..20
8. 評価項目 20
8-1. 主要評価項目..20
8-1-1. 第Ｉa 相部 20
8-1-2. 第Ｉb 相部 20
8-2. 副次的評価項目..20
8-2-1. 第Ｉa 相部 20
8-2-2. 第Ｉb 相部 20

8-3. 評価項目の定義 20
8-3-1. 主要評価項目の定義 20
8-3-2. 副次的評価項目の定義 21
8-3-3. その他の項目の定義 24
9. 観察項目・方法 25
9-1. 治験スケジュール 25
9-2. 患者背景 27
9-3. 治験薬の投与及び併用治療の状況 27
9-4. 有効性に関する項目 27
9-4-1. 抗原特異的抗体免疫反応検査（NY-ESO-1 及び XAGE-1b） 27
9-4-2. 制御性 T 細胞検査（FOUR-COLOR FOXP3 検査、CD4/CD25/CCR4 検査及び MLA CD45 ゲーティング） 28
9-4-3. CT 又は MRI 28
9-4-4. 生存期間調査及び後治療 28
9-4-5. 腫瘍マーカー（任意） 28
9-4-6. 制御性 T 細胞の評価（任意） 28
9-4-7. 抗原特異的抗体免疫反応検査（NY-ESO-1 及び XAGE-1b 以外の抗原）（任意） 29
9-4-8. 抗原特異的細胞免疫反応検査（任意） 29
9-4-9. サイトカイン産生能評価（任意） 29
9-4-10. 免疫担当細胞・制御細胞解析（任意） 29
9-4-11. 免疫組織染色（任意） 29
9-4-12. 遺伝子解析（任意） 30
9-5. 安全性に関する項目 30
9-5-1. PS（Performance Status） 30
9-5-2. バイタルサイン(血圧・脈拍数、体温、呼吸数) 30
9-5-3. 体重 30
9-5-4. 一般血液検査 30
9-5-5. 甲状腺機能検査 30
9-5-6. 尿検査 31
9-6. 薬物動態（第Ｉa 相部） 31
10. 安全性に関する取扱い 31
10-1. 定義 31
10-1-1. 臨床検査値の異常及び異常変動の定義 31
10-1-2. 有害事象及び副作用（因果関係が否定できない有害事象）の定義 31
10-1-3. 重篤な有害事象の定義 32
10-1-4. 症状及び所見に関する有害事象 32
10-2. 調査項目 32
10-2-1. 有害事象名 32

10-2-2. 発病（発現）日及び最悪化日 32
10-2-3. 重症度..32
10-2-4. 重篤度..32
10-2-5. 処置..33
10-2-6. 転帰..33
10-2-7. 転帰日..33
10-2-8. 治験薬との因果関係 33
10-3. 有害事象の報告と対応 34
10-3-1. 重篤な有害事象報告手順..34
10-3-2. 効果安全性評価委員会による評価・勧告 35
10-3-3. 対策の決定..35
10-4. 予想される副作用 35
10-5. 副作用発現時の対処..37
10-5-1. 注入に伴う反応 37
10-5-2. 皮膚障害 37
10-5-3. B 型肝炎ウイルスによる肝炎..38
10-5-4. 腫瘍崩壊症候群 38
10-5-5. 血液毒性・肝機能障害 38
10-6. 妊娠が確認された場合の措置 38
10-7. 過量投与を行った場合の措置 38
11. 統計解析 38
11-1. 解析対象集団..38
11-2. 被験者の取扱い基準..39
11-3. データの取扱い基準..39
11-4. 解析項目・方法 41
12. ゲノム・遺伝子解析の実施..42
12-1. 目的 42
12-2. 試料の取り扱い 42
12-3. 解析方法 43
12-4. 試料提供に関する同意について..43
12-5. 被験者への情報の開示 43
13. 治験実施計画書の遵守及び逸脱、変更ならびに改訂 43
14. 治験の中止と終了 44
14-1. 個々の被験者に対する中止規定又は中止基準..44
14-1-1. 個々の被験者に対する治験薬の投与中止基準..44
14-1-2. 個々の被験者に対する治験の中止基準..44
14-1-3. 実施医療機関での中止又は中断..44
14-2. 治験の一部及び全体の中断、又は中止規定 44
14-3. 治験の終了 45

15. 症例報告書等の記入と提出 45
15-1. 提出資料 45
15-2. 様式と提出期限 45
15-3. 記入方法 46
15-4. 症例報告書の作成及び送付 46
15-5. 症例報告書の変更又は修正 46
15-6. Data Clarification Form(DCF)の発行・提出等 46
15-7. 症例報告書と診療録等の原資料に矛盾がある場合 46
15-8. 症例報告書及びデータの取扱い 46
16. 原資料の閲覧に関する事項 46
17. 治験の品質管理及び品質保証 47
18. 倫理的配慮 47
18-1. 遵守すべき諸規則 47
18-2. 治験審査委員会 47
18-3. インフォームド・コンセント 48
18-4. 新たな情報の提供 48
18-5. 被験者の機密保持・プライバシー確保 48
19. 必須文書の保存 48
19-1. 自ら治験を実施する者 48
19-2. 実施医療機関 48
19-3. 治験責任医師 49
20. 金銭の支払い及び保険 49
20-1. 金銭の支払い 49
20-2. 補償 49
21. 資金源及び起こりうる利害の衝突 49
22. 試験成果の帰属と結果の公表 49
22-1. 結果の公表 49
22-2. 総括報告書の作成 49
22-3. データの提供 49
22-4. データの二次利用 49
23. 参考文献 51

1. 開発の背景

1-1. 進行又は再発、食道がん、胃がん、肺がん、悪性黒色腫、卵巣がんとその治療

食道がんは、診断時には進行していることが多く、予後不良ながんである。日本における悪性腫瘍による死亡の 3.5%を占め、罹患率は 10 万人比約 10 人である [1) 2)]。食道周囲には主要臓器が接していて、直接浸潤を起こしやすく、さらに血行性･リンパ行性転移も多い。手術が唯一の根治療法ではあるが、手術可能症例でも 5 年生存率は 36%と低く、IV 期では 10%である。近年、CDDP、5-FU、Docetaxel 等の化学療法や放射線療法等により、治療成績が改善したが、依然 3 年生存率は 30%前後である。

胃がんは、消化器がんの中で最も死亡者数が多く、国際比較でも非常に患者数が多い。死亡者数・罹患率ともに、近年、減少傾向にはあるが、依然として男性・女性を問わず 2～3 位を占め、毎年 10 万人以上が胃がんを発症し、5 万人近くが死亡している [1)]。手術が唯一の根治療法ではあるが、手術可能症例であっても 5 年生存率は約 60%であり、IV 期では 20%前後である。近年、CPT-11、CDDP、TS-1 等の多剤併用療法で奏効率は改善しているが、依然予後不良である。

肺がんは、その罹患率、死亡率は、ともに男性のほうが女性より高く、男性は女性の 3 倍から 4 倍にのぼり、悪性腫瘍による死亡数では肺がんは男性・女性ともに第 1 位である [1)]。罹患数と死亡数に大きな差はなく、これは、肺がん罹患者の生存率が低いことと関連している。肺がんは、小細胞がんと非小細胞がんの 2 つの型に大きく分類され、特に約 15～20%を占める小細胞がんは増殖が速く、脳・リンパ節・肝臓・副腎・骨等に転移しやすい悪性度の高いがんである。治療法としては、外科的切除以外では放射線療法やプラチナ製剤と併用した多剤投与が行われている。近年、タキサン系、ゲムシタビン、イリノテカン等の有効性が評価されているが、その予後は、非小細胞肺がんでの病期Ⅲ期であっても 5 年生存率は 20%以下と不良である。

悪性黒色腫は、その死亡率は戦後減少傾向が続き、現在では 10 万人当たり約 1 人と欧米に比較してまれである [1)]。しかし悪性度は非常に高い悪性腫瘍であり、切除不能進行例の場合、5 年生存率は約 10%と低い。化学療法としては、これまで様々な多剤併用化学療法が試みられてきた。近年では DTIC が進行期悪性黒色腫患者に対して最も頻用されている薬剤であるが、その奏効率は約 20%、完全奏効率は 5-10%、長期完全奏効率は 2%以下であり、満足できるものではない。

卵巣がんは、人口 10 万人あたり 11.4 人の罹患率であり、全女性器がんの約 32%を占めている [1)]。50 歳以降で増加を示し高齢者ほど罹患率が高い。大半の症例では、積極的な腫瘍減量手術が行われるが、手術のみでは治癒は望めず、術後に化学療法が実施される。初回薬物療法としては CBDCA と PTX の併用である TC 療法が標準療法として用いられているが、5 年生存率は 49.7%にすぎない。IV 期の 5 年生存率は 12%である。

これらのがん腫をはじめとする種々の高度進行･再発がん症例に対して、手術療法、化学療法、放射線療法に代わる、特異性、有効性、安全性に優れた新しい治療法の開発が求められている。

1-2. 腫瘍免疫

1-2-1. 免疫の抗腫瘍効果

免疫は、腫瘍の発生段階から、確立した腫瘍の排除にまで関与している。腫瘍の発生には、免疫学的監視機構が関与している。このことは、RAG 遺伝子欠失免疫不全マウスでは、ほとんどの

マウス個体に腫瘍形成が見られることから明らかとなった。また、近交系マウス間において同系腫瘍を移植した場合、生着腫瘍を切除したマウスでは再移植した同一腫瘍は拒絶される。一方で、別の腫瘍は生着・増殖することから、確立した腫瘍についても免疫の特異的な抗腫瘍効果が証明されている。同時に、腫瘍抗原及び腫瘍拒絶の効果細胞である細胞傷害性 T 細胞（CTL : cytotoxic T lymphocyte）の存在も明らかとなっている [3)] 。

1-2-2. 腫瘍免疫の臨床応用

腫瘍特異的に反応する CTL を患者体内で効率よく誘導あるいは増殖させることが、臨床的に抗腫瘍効果をもたらすと考えられている。抗腫瘍 CTL の効率的な誘導・増殖には、現在、大きく分けて 3 つの方法が用いられている。

第一に、がんワクチンのように腫瘍抗原特異的に働く CTL を体内で誘導、あるいは CTL 自体を輸注する養子免疫療法である。われわれは 2004 年より NY-ESO-1 タンパク、NY-ESO-1 長鎖ペプチド、NY-ESO-1 長鎖複合ペプチドさらには MAGE-A4 タンパクを用いたがんワクチン臨床試験を実施してきた。ワクチン投与後の患者体内では、抗原特異的に液性免疫及び細胞性免疫の誘導・増強が明らかとなっている [4)] 。しかし、臨床効果は限定的であった。

第二に、CTL の働きを抑制する分子を制御する方法である。近年アメリカで Ipilimumab を用いた腫瘍免疫増強効果が有望視されている。Ipilimumab は T 細胞の働きを抑制する CTLA4 分子に対する抗体であり、そのアンタゴニスト効果により抗腫瘍免疫、特に腫瘍特異的反応性 CTL がより有効に働くとされている。Ipilimumab 投与の臨床試験では参加 140 症例中、34%の悪性黒色腫患者に何らかの臨床効果をもたらしたことが報告された [5)] 。がん精巣抗原の一つで、強い免疫原性を持つ NY-ESO-1 は、この Ipilimumab 投与による臨床効果の際の抗腫瘍免疫に関与する非常に重要な因子として同時に報告されており、NY-ESO-1 抗体を持つ患者での臨床効果が有意に高かったと報告されている [6)] 。

第三の方法として近年有望視されているのは、CTL の働きを抑制する免疫細胞のコントロールである。この種類の細胞は制御性 T 細胞(regulatory T cell; Treg)と云われ、CD4 陽性 CD25 陽性の細胞群で転写因子 Foxp3 を特異的に発現している。制御性 T 細胞は、元来、自己に対する免疫応答を制御し、免疫寛容の維持に重要な役割を果たしているが、抗腫瘍免疫をも抑制しており、CD4 陽性 CD25 陽性細胞を除去することにより、マウス移植腫瘍が退縮することが明らかにされた [7)] 。さらに、最近、CCR4 ノックアウトマウスでは、制御性 T 細胞の抑制のため腫瘍増殖が抑制されることが明らかになった（下図参照）。Foxp3 を発現するヒト制御性 T 細胞の中でも、CTL の抑制に働く分画は活性化制御性 T 細胞であり、CCR4 を強く発現していることが明らかとなっている [7)、8)] 。上に述べたがんワクチン療法を受けた症例の体内では、抗原特異的免疫反応の誘導・増強は見られるものの、制御性 T 細胞の存在が臨床効果を抑制している可能性がある [9)] 。

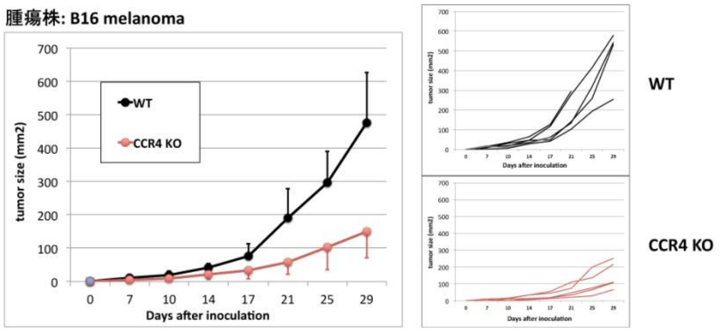

図　悪性黒色腫腫瘍はCCR4ノックアウトマウスで腫瘍増殖が阻害される
悪性黒色腫株(B16 melanoma)を野生型マウスとCCR4ノックアウトマウスに接種し、腫瘍増殖を検討した

1-3. Mogamulizumab について

協和発酵キリン株式会社が開発したヒト化抗 CCR4 モノクローナル抗体 KW-0761 は脱フコシル化技術により ADCC 活性を飛躍的に高めた抗体である [9]。CCR4 を発現する成人 T 細胞白血病リンパ腫（ATL）に対する治療薬剤として、平成 24 年 3 月に厚生労働省より製造承認を受けた [10] [11]。

CCR4 はATL 患者の約90%で発現がみられると報告されている [9]。本剤は世界初のCCR4 を標的とした抗体医薬品である。

本剤は、ADCC 活性を高めるポテリジェント技術を用いた世界初のポテリジェント抗体であり、ADCC 活性により抗腫瘍効果を示す。

前治療としての化学療法によって寛解に到達しなかった治療抵抗例を除く、CCR4 陽性の再発・再燃ATL 患者（急性型、リンパ腫型又は予後不良因子※を有する慢性型）に対して、本剤を単剤投与した際の奏効率は50.0%［13/26 例、完全寛解（CR）30.8%、部分寛解（PR）19.2%］であった [11]。［国内第Ⅱ相試験（ATL/単剤投与）］

※血漿尿素窒素(BUN)高値、乳酸脱水素酵素(LDH)高値又はアルブミン低値のいずれかを有する。

1-3-1. 非臨床試験データの概要

1-3-1-1. 薬理試験

Mogamulizumab の効力を裏付ける試験として実施した *in vitro* 試験の結果、Mogamulizumab の作用メカニズムは、ADCC 活性であることが示された。Mogamulizumab は、T 細胞由来の腫瘍細胞株のみならず ATL 患者由来の腫瘍細胞に対しても ADCC 活性を示したことから、ATL 患者への有効性が示唆された。カニクイザルに Mogamulizumab を静脈内投与した *in vivo* 試験では、0.01 mg/kg 以上を投与した群に末梢血リンパ球中の CD4 陽性/CCR4 陽性細胞数の明らかな減少が示された。更に、マウス皮下に CCR4 陽性ヒト T 細胞リンパ腫細胞を移植したモデルで 20 mg/kg の投与量で腫瘍増殖の抑制が認められ、*in vivo* での抗腫瘍作用が示された。以上の結果より、CCR4 陽性の ATL に対する Mogamulizumab の薬効が期待された。

副次的薬理評価では、CCR4 の発現が報告されている血小板に対する影響を評価した。Mogamulizumab は血小板へ結合せず、TARC、ADP 及びコラーゲンで誘発される血小板凝集作用に影響を及ぼさなかった。更に、Mogamulizumab を添加したヒト全血においても血小板数の

減少は認められなかった。以上の試験結果より、Mogamulizumab がヒト血小板への直接的な作用を示さないものと考えられた。Mogamulizumab によるリンパ球からの IFN-γ 分泌作用は 6 例中 5 例で認められなかった。IFN-γ の分泌が認められた 1 例も、その分泌作用は陽性対照抗体よりも弱かった。一方、TNF-α の分泌は 6 例全例で認められなかった。したがって、Mogamulizumab の TNF-α及び IFN-γ 分泌作用は 陽性対照抗体よりも低く、ヒトへの投与でも特に問題はないと考えられた。

安全性薬理は単回及び反復投与した毒性試験の中で評価した。一般状態観察のほか、電気生理学的検査、神経行動学的検査、心電図、心拍数、血圧、体温、血液生化学的検査及び病理組織学的検査を実施した。いずれの測定項目にも異常は認められず、Mogamulizumab は循環器系、呼吸器系、腎臓系及び中枢神経系への影響はないものと考えられた。

1-3-1-2. 毒性試験

毒性はヒトと同様の組織で抗原発現が認められたカニクイザルを使って評価した。急性毒性としては、単回 100 mg/kg までの投与量で死亡例や一般状態の変化は認められず、概略の致死量は 100 mg/kg 以上と考えられた。また、週 1 回 13 週までの反復投与では被験物質の影響は認められず、無毒性量は今回検討した最高投与量の 40 mg/kg/週であった。無毒性量での $AUC_{0\text{-}7d}$ は臨床試験において 1 mg/kg で 8 回投与したときの $AUC_{0\text{-}7d}$ の値の 33 倍以上であった。胚・胎児発生に関する試験では、妊娠カニクイザルへの 40 mg/kg/週の投与により、母動物の一般毒性及び生殖に関する影響は認められなかった。更に、胎児への Mogamulizumab の移行が認められたものの、胚・胎児発生への影響は認められなかった。また、各毒性試験内での投与部位の評価において、局所刺激性を示す所見は認められなかった。

カニクイザルに単回及び反復投与した試験で、抗 Mogamulizumab 抗体の産生が認められた。抗 Mogamulizumab 抗体の産生が認められた個体でも、毒性試験では特記すべき所見はなく、抗 Mogamulizumab 抗体が安全性上の問題となることはないと考えられた。

毒性試験で認められた CCR4 陽性 T 細胞の減少は、 Mogamulizumab の薬理作用に基づくものである。単回投与で 12 ヵ月の観察期間とした試験では、回復傾向は認められたものの、観察期間を通じて低値を示した。4 週間の反復投与試験でも、約 3 ヵ月の休薬期間で回復傾向が認められたものの、最終検査時点でも低値を示した。しかし、いずれの試験においても毒性兆候は認められず、CCR4 陽性 T 細胞の減少が持続したことで、毒性を発現する懸念はないものと考えられた。

1-3-1-3. 薬物動態及び薬物代謝

薬物動態は静脈内投与による血漿中濃度推移で評価した。単回及び反復投与試験の結果から、0.5 mg/kg 以上の投与量での線形性が確認された。また、血漿中濃度推移に雌雄差はなく、Mogamulizumab の体内動態に雌雄差はないものと考えられた。反復投与による薬物動態パラメータの変化はなく、反復投与によって定常状態になることも示された。

[^{125}I]KW-0761 を用いたカニクイザルの分布試験の結果より、血漿と血液を除く組織への分布量は最大で投与量の 4.86%であり、Mogamulizumab の組織分布は低いものと考えられた。妊娠カニクイザルへの反復投与の結果、血漿中濃度は非妊娠カニクイザルと同程度であり、妊娠によ

る薬物動態の変化はないと考えられた。また、胎児で Mogamulizumab の曝露が確認され、Mogamulizumab は胎盤を通過し胎児へ移行することが示された。

以上より、非臨床試験の結果から安全性に懸念のある事項は認められなかった。

1-3-2. 臨床試験データの概要

1-3-2-1. CCR4 陽性の再発・再燃 ATL 患者及び末梢血 T 細胞リンパ腫(PTCL)患者を対象とした国内第 I 相臨床試験

本邦において、第 I 相試験は、多施設共同非盲検用量設定試験として実施した。

対象は CCR4 陽性の化学療法奏効後に再発又は再燃した ATL 患者又は MF 等を含む PTCL 患者とし、Mogamulizumab 0.01、0.1、0.5 又は 1.0 mg/kg を 1 週間間隔で 4 回静脈内投与した際の安全性及び薬物動態を検討した。また、副次的に有効性も検討した。低い投与量から 3 名ずつ（最大 6 名）に静脈内投与し、安全性を確認した後、段階的に高い投与量へ移行した。3 名に用量制限毒性（DLT）が認められた投与量を最大耐用量（MTD）、MTD の 1 段階下の投与量を推奨用量（MTD に達しなかった場合は 1.0 mg/kg）とし、推奨用量に新たに 3 名を追加した。なお、第 I 相臨床試験で奏効が得られた後、再発又は再燃した被験者には Mogamulizumab の再投与を可とした。

0.01mg/kg から 1.0mg/kg まで順次増量していく中（計 13 名）で DLT は認められず、MTD に達しなかったため、推奨用量は 1.0mg/kg となり、さらに 3 名が追加された（合計 16 名）。推奨用量(1.0mg/kg)に追加された 3 名中 1 名に、DLT に該当する有害事象（好中球減少 Grade4、発熱性好中球減少 Grade3 並びに皮疹 Grade3）が認められたが、投与量 1.0mg/kg における DLT の発現は 6 名中 1 名であったことから、1.0mg/kg まで忍容可能と判断された。

Mogamulizumab は、他の抗体医薬等と同様に、発熱、悪寒等のサイトカイン放出症候群／急性輸注反応の発現や、リンパ球、好中球、白血球等の血球減少が高頻度に認められたが、いずれも自然経過あるいは薬剤等の適切な処置により回復が認められた。血小板減少は半数程度の被験者に認められたものの全て Grade2 以下の減少（Grade1：7 名、Grade2：1 名）でいずれも回復が認められ、Grade2 の血小板減少が認められた 1 名の血小板機能検査に異常値は認められなかった。本治験で発現した重篤な有害事象は、帯状疱疹、低酸素血症及び皮疹であった。

Mogamulizumab 4 回目投与時の最高血漿中薬物濃度(C_{max})及び 0-7 日目までの血漿中薬物濃度、時間曲線下面積（$AUC_{0\text{-}7\ days}$）は、0.01～1.0mg/kg の投与量範囲で投与量の増加に比例して増加していることが示された。また、血漿中抗 Mogamulizumab 抗体濃度ではすべての被験者において定量下限未満（<5.0mg/mL）であった。

奏効率は 31.3%(CR2 名+PR3 名／16 名)であった。ATL に対しては 30.8%（CR2 名＋PR2 名／13 名）の奏効率であった。

また、Mogamulizumab の再投与が実施された 1 例は、2 回投与した後に投与基準を満たさず（AST 上昇 Grade3）中止となった。再投与で発現した重篤な有害事象は B 型肝炎であった。なお、有効性は投与中止により評価不能であった。

1-3-2-2. CCR4 陽性の再発・再燃 ATL 患者を対象とした国内第Ⅱ相臨床試験

本治験は、多施設共同非盲検試験として実施した。

Mogamulizumab 投与直近の化学療法で奏効後に再発・再燃した CCR4 陽性 ATL 患者を対象として、Mogamulizumab　1.0mg/kg を 1 週間間隔で 8 回静脈内投与した。本治験には 28 名が登録され、そのうち 27 名に治験薬が投与された。26 名を有効性の解析対象、27 名を安全性及び薬物動態の解析対象とした。

主要評価項目である総合最良効果の奏効率は 50.0%（13/26 名）、95%信頼区間は 29.9～70.1%であった。95%信頼区間の下限値(29.9%)は閾値奏効率 5%を上回り、Mogamulizumab の有効性が確認された。なお、Kaplan-Meier 法により推定した PFS の 50%点は 158 日であり、最小値 12 日～最大値 401 日（打切り）であった。また、全生存期間(OS)の 50%点は死亡日調査時点で生存割合が 50%以上であったため算出不能であったが、最小値 101 日～最大値 401 日（打切り）であった。

本治験では死亡は認められず、重篤な又は重症度の高い有害事象の発現は認められたものの、いずれも適切な処置によりコントロール可能であった。本治験において発現した因果関係が否定できない重篤な有害事象は、「スティーブンス・ジョンソン症候群」及び「発疹」であった。また、発現した主な有害事象は、第 I 相臨床試験と同様、「発熱」、「悪寒」等を主訴とする「注入に伴う反応」及び「リンパ球数減少」、「白血球数減少」、「好中球数減少」、「血小板数減少」等の血球数減少並びに「発疹」であった。

コンパートメントオープンモデルで解析した Mogamulizumab8 回投与後の C_{max} 及び C_{trough} 推定値は実測値の平均値とおおむね等しく、反復投与により Mogamulizumab の薬物動態はほとんど変化しないと考えらえた。

血漿中抗 Mogamulizumab 抗体濃度は全ての被験者において定量下限未満（<5.0mg/mL）であった。

なお、本治験では Mogamulizumab の 8 回投与で最良総合効果が PR 以上と判定された後、再発又は再燃した被験者のうち、被験者本人が希望し、且つ医師が有用と判断した場合、再投与を可能とした。Mogamulizumab 再投与は 1 名の被験者に実施され、当該被験者の再投与時の最良総合効果は PR であった。再投与時に初めて発現した有害事象はなく、主な事象はこれまでの試験で確認された事象と同様に「注入に伴う反応」及び血球数減少（「リンパ球数減少」、「白血球数減少」）であった。また、再投与時の血漿中抗 Mogamulizumab 抗体濃度はすべての採血時点において定量下限未満（<5.0mg/mL）であった。

1-4. 副作用

国内臨床試験の安全性評価対象43 例における副作用発現率（臨床検査値異常を含む）は100%（43/43 例）であった。主な副作用（10.0%以上）は、リンパ球減少41 例（95.3%）、Infusion reaction 37 例（86.0%）、発熱34 例（79.1%）、白血球減少29 例（67.4%）、好中球減少24 例（55.8%）、悪寒24 例（55.8%）、血小板減少23 例（53.5%）、発疹18 例（41.9%）、ALT（GPT）上昇17 例（39.5%）、AST（GOT）上昇16 例（37.2%）、LDH 上昇13 例（30.2%）、頻脈12 例（27.9%）、ALP 上昇11 例（25.6%）、低酸素血症9 例（20.9%）、ヘモグロビン減少9 例（20.9%）、悪心8 例（18.6%）、血圧上昇8 例（18.6%）、低アルブミン血症7 例（16.3%）、そう痒症6 例（14.0%）、体重増加6 例（14.0%）、血中アルブミン減少5 例（11.6%）、γ-GTP 上昇5 例（11.6%）、頭痛5 例（11.6%）、高血圧5 例（11.6%）等であった。

［申請時］重大な副作用として、Infusion reaction（86.0%）、重度の皮膚障害、感染症（4.7%）、B型肝炎ウイルスによる劇症肝炎（頻度不明）、肝炎（2.3%）、腫瘍崩壊症候群（2.3%）、重度の血液毒性、肝機能障害が報告されている。

1-5. まとめ

がん患者では、制御性 T 細胞が抗腫瘍免疫反応を抑制していることが知られている。制御性 T 細胞のうち、特に活性化制御性 T 細胞は膜表面に CCR4 を発現することから、この医師主導治験では、Mogamulizumab 投与により活性化制御性 T 細胞分画を除去し臨床効果をもたらすことを期待して計画した。

2. 治験の実施体制と役割

別冊 1「治験実施体制」参照。

3. 治験の目的

進行再発がん患者に対して Mogamulizumab（抗 CCR4 抗体）を週 1 回反復投与した際の安全性及び薬物動態を検討すること（第 I a 相部）、及び週 1 回反復投与した際の安全性及び制御性 T 細胞除去効果を検討すること（第 I b 相部）を目的とする。

4. 対象

進行又は再発固形がん患者

4-1. 被験者の選択基準

進行又は再発固形がん患者で、登録時に以下の基準を全て満たす患者を適格とする。

1) 腫瘍細胞の CCR4 発現が陰性（診断は本邦で商品化されている方法を用いること）であり、且つ病理組織診断で肺がん、胃がん、食道がん、悪性黒色腫、卵巣がん等の悪性腫瘍の診断が確定している患者
2) 標準治療法に不応・不耐性の患者、適切な治療法がない患者、又は標準治療を拒否した患者
3) performance status（ECOG 基準）が 0、1、2 であること
4) 治験参加同意取得日の年齢が 20 歳以上であること
5) 主要臓器（骨髄、心、肺、肝、腎等）に高度な障害がなく臨床検査値（登録前 2 週間以内）が以下の基準を満たすこと

好中球数：	1,500/μL 以上
ヘモグロビン値：	8.0g/dL 以上
血小板数：	75,000/μL 以上
血清総ビリルビン値：	2.0 mg/dL 以下
AST(GOT)、ALT(GPT)値：	施設基準値上限×2.5 以下（原疾患による肝浸潤に起因すると判断される場合においては、施設基

準値上限×5.0 以下）

血清クレアチニン：　1.5 mg/dL 以下

動脈血酸素飽和度：　93%以上（室温）

心電図：　治療を要する異常所見を認めない

左心駆出率（心エコー検査による）：50%以上

6) 同意取得時から治験薬投与終了後 24 週まで、コンドーム等の避妊方法による避妊に同意していること（閉経後（最終月経から 1 年以上経過）又は外科的不妊手術を施行した女性及び外科的避妊術を施行した男性は除く）
7) 本人が文書により同意していること
8) Mogamulizumab の初回投与前から初回投与翌日まで投与入院が可能な患者
9) 効果判定の対象になる RECIST ver.1.1（New response evaluation criteria in solid tumours:Revised RECIST guideline (version 1.1).）[12] における測定可能病変を有すること
10) 3 カ月間以上の生存が期待できること

＜設定根拠＞

1) 制御性 T 細胞除去効果による臨床効果を確認するため、及び病理学的に対象の腫瘍を有していることを確診するため設定した。
2) 本治験薬を投与することに医療的、倫理的な問題がない患者を選択するため設定した。
3) 治験参加に支障がない一般状態の患者を選択するために設定した。
4) 患者本人より同意取得可能な年齢として設定した。
5) 安全性が確保できる患者を選択するため設定した。
6) 治験薬の胎児に対する安全性は確立されていないため設定した。
7) 適切に同意取得された患者を選択するために設定した。
8) 注入に伴う反応等が発生した際の被験者の安全性確保のため設定した。
9) 治験薬の有効性を評価できる患者を選択するため設定した。
10) 治験薬の反復投与の安全性及び有効性を評価するために、少なくとも 3 カ月間以上の生存が期待できる患者を選択するため設定した。

4-2. 被験者の除外基準

登録時に以下のいずれかの基準に該当する患者は除外する。

1) HIV 抗体陽性例
2) HCV 抗体陽性例
3) 自己免疫疾患例
4) HBs 抗原が陽性、もしくは HBV-DNA がリアルタイム PCR 法により「検出」と報告された患者
5) 抗体製剤の投与により重篤な過敏症の既往歴を有する患者
6) 重複がんを有する患者。ただし、重複がんとは、同時性重複がん及び無病期間が 5 年以内の異時性重複がんである。局所治療により治癒と判断される Carcinoma in situ （上皮内がん）もしくは粘膜内がん相当の病変は重複がんに含めないこととする。

7) 抗がん剤、副腎皮質ステロイド剤の持続全身投与、免疫抑制剤及び免疫強化剤の投与、サイトカイン療法、放射線療法及び原疾患に対する手術の施行から治験薬投与開始予定日まで 4 週以上経過していない場合
8) 妊娠中、授乳中、あるいは妊娠の可能性のある患者
9) 活動性の感染症を合併している患者
10) 精神障害又は認知症患者
11) 全身性のステロイド剤投与による治療の継続が必要な患者
12) 造血幹細胞移植等の移植療法が実施された患者
13) 中枢神経腫瘍浸潤を有する患者又はそれを疑わせる臨床所見を有する患者
14) 症例登録 4 週間以内に他の治験薬を投与された患者
15) 症例登録 12 週間以内に腫瘍に対する免疫療法(腫瘍ワクチン等)が実施された患者
16) その他治験遂行に不適当と考えられる患者

＜設定根拠＞
1)、2)、4) 安全性及び血液等の検体を取り扱う者の安全性を考慮して設定した。
3) 本治験薬の投与により、自己免疫疾患が増悪する可能性があることより設定した。
5)、8)、9) 安全性を確保できない可能性があるため設定した。
6) 安全性及び生命予後に影響する可能性があるため設定した。
7) 有効性の評価に影響を及ぼす可能性があるため設定した。
10) 適切な同意取得が困難である症例を除外するため設定した。
11) 本治験薬の評価に影響を与える可能性があるため設定した。
12) 本治験薬の投与により、移植片対宿主病（GVHD）が生じる可能性があることより設定した。
13) 安全性の評価に影響を及ぼす可能性があるため設定した。
14) 安全性の確保及び本治験薬の評価に影響を与える可能性があるため設定した。
15) 安全性の確保及び本治験薬の評価に影響を与える可能性があるため設定した。

5. 被験者の同意取得

5-1. 同意文書及びその他の説明文書

自ら治験を実施する者は、被験者から治験への参加の同意を得るために同意文書及びその他の説明文書を作成し、必要な場合にはこれを改訂するものとする。作成又は改訂された当該文書は、あらかじめ治験審査委員会の承認に基づく実施医療機関の長の決定が得られていなければならない。

5-2. 同意取得の時期、方法及びその内容

5-2-1. 治験開始時の同意取得

治験責任医師あるいは治験分担医師（以下、「治験責任医師等」という）は、症例登録に先立って対象となる被験者本人に以下に示す内容について記載された説明文書（あらかじめ各実施医療機関の治験審査委員会で承認を得たもの）を手渡し、十分に説明し、被験者が内容をよく理解

したことを確認した上で、本試験への参加について、被験者本人の自由意思による同意を文書にて得る。なお、同意書には説明を行った治験責任医師等及び被験者本人が記名・捺印又は署名し、各自日付を記入する。

また、治験協力者が補足的な説明を行った場合には、当該治験協力者も記名・捺印又は署名し、日付を記入する。なお、実施医療機関に特別の取り決めがある場合を除いて 3 連複写式の同意書の一枚を被験者へ手渡し、一枚を実施医療機関の治験事務局等、実施医療機関の定める部署へ提出し、残りの一枚を診療録と共に保存する。

1) 治験が研究を伴うものであること
2) 治験の目的
3) 治験責任医師等の氏名、職名及び連絡先
4) 治験の方法（治験の試験的側面、被験者の選択基準）
5) 予期される治験薬による被験者の心身の健康に対する臨床上の利益及び危険性又は不便
6) 他の治療方法の有無及びその治療方法に関して予測される重要な利益及び危険性
7) 被験者の治験への参加予定期間
8) 治験への参加は被験者の自由意思によるものであり、被験者は治験への参加を随時拒否又は撤回することができること。また、拒否・撤回によって被験者が受けるべき利益を失うことがないこと。
9) モニター、監査担当者、治験審査委員会等及び規制当局が原資料を閲覧できること。その際、被験者の秘密は保全されること。また、同意文書に被験者が記名・捺印又は署名することによって閲覧を認めたことになること。
10) 治験の結果が公表される場合であっても、被験者の秘密は保全されること
11) 被験者が治験及び被験者の権利に関してさらに情報が欲しい場合又は治験に関連する健康被害が生じた場合に照会すべき又は連絡を取るべき実施医療機関の相談窓口
12) 治験に関連する健康被害が発生した場合に被験者が受けることのできる治療
13) 治験への参加の継続について、被験者の意思に影響を与える可能性のある情報が得られた場合には速やかに被験者に伝えられること。
14) 治験への参加を中止させる場合の条件又は理由
15) 被験者が費用負担をする必要がある場合にはその内容
16) 被験者が守るべき事項
17) 当該治験の適否等について調査審議を行う治験審査委員会の種類、各治験審査委員会において調査審議を行う事項その他当該治験に係る治験審査委員会に関する事項
18) 治験審査委員会の手順書等が確認することができること及び治験審査委員会の手順書等を確認したい場合には申し出てほしいこと。また、治験審査委員会の手順書等をホームページで公表している場合は当該ホームページのアドレスを、公表していない場合は一般の閲覧に供していること。
19) 遺伝子検査に係る事項
20) 当該治験に係る必要な事項

5-2-2. 第Ｉa 相部の 5 回目以降の治験薬投与に関する同意取得

治験責任医師等は 5 回目の治験薬投与に先立ち、以下の内容について記載された説明文書を手渡し、十分に説明した上で、本治験への参加の継続について被験者本人の自由意思による追加的な同意を文書にて得ることとする。その他、同意取得の方法については「5-2-1. 治験開始時の同意取得」に準じる。

1) 最大耐用量を決定するための投与期間が終了又は終了予定であること。
2) 治験の目的
3) 治験責任医師等の氏名、職名及び連絡先
4) 被験者の治験への参加予定期間
5) 治験への参加は被験者の自由意思によるものであり、被験者は治験への参加を随時拒否又は撤回することができること。また、拒否・撤回によって被験者が不利な扱いを受けたり、治験に参加しない場合に受けるべき利益を失うことはないこと。
6) 被験者が治験及び被験者の権利に関してさらに情報が欲しい場合又は治験に関連する健康被害が生じた場合に照会すべき又は連絡を取るべき実施医療機関の相談窓口
7) 治験への参加の継続について被験者の意思に影響を与える可能性のある情報が得られた場合には速やかに被験者に伝えられること。
8) 治験への参加を中止させる場合の条件又は理由

5-3. 同意に関する留意事項

1) 治験責任医師等は、同意を得る前に、被験者となるべき者が質問する機会と治験に参加するか否かを判断するのに十分な時間を与える。その際、当該治験責任医師等又は補足説明者としての治験協力者は、全ての質問に対して被験者となるべき者が満足するように答える。
2) 治験責任医師等及び治験協力者は、治験への参加又は治験への参加の継続に関し、被験者となるべき者に強制したり又は不当な影響を及ぼしてはならない。
3) 原則として、同意書には被験者自ら記名・捺印又は署名するが、被験者が同意文書及びその他の説明文書を読むことができないが、口頭又は他の伝達方法ではその内容を理解することができる場合には、説明に際して公正な立会人を要する。被験者に対して、同意文書及びその他の説明文書が渡され、その内容が口頭又は他の伝達方法により説明され、被験者が治験への参加に口頭で同意し、さらに被験者が同意書に記名・捺印又は署名し、自ら日付を記入した後に、立会人も同意書に記名・捺印又は署名し、自ら日付を記入する。
4) 説明に際して、被験者となるべき者に権利を放棄させるかそれを疑わせる語句、又は治験責任医師、治験分担医師、治験協力者、実施医療機関、自ら治験を実施する者の法的責任を免除するかそれを疑わせる語句が含まれてはならない。また、被験者となるべき者（被験者となるべき者が説明文書を読むことができないが、口頭又は他の伝達方法ではその内容を理解することができる場合における公正な立会人を含む。）が理解可能で、可能な限り非専門的な言葉を用いる。
5) 本治験では、本治験薬の評価に係るゲノム・遺伝子解析を行うことを計画しており、提供を受けた試料のゲノム･遺伝子解析を目的とした使用に関して被験者となるべき者に予め十分な説明を行った上で、文書による同意を取得する。試料のゲノム・遺伝子解析への使用に関

しては、本内容について文書による同意を取得した被験者に限って実施することができる。治験責任医師又は治験分担医師は、試料の提供が、被験者となるべき者の自由意思に基づくものであり、また本治験への参加は試料の提供に左右されるものではないことを、被験者となるべき者に説明しなければならない。また、ゲノム・遺伝子解析を目的とした試料の使用についての同意取得後に同意を撤回する場合は、別途文書による同意撤回書の提出を必要とする。なお、試料の使用について、実施医療機関の治験審査委員会により承認されなかった場合、ゲノム・遺伝子解析を目的とした試料の使用は行わない。

5-4. 同意文書及びその他の説明文書の改訂

1) 治験への継続参加について被験者の意思に影響を与える可能性のある情報が得られた場合には、治験責任医師等は当該情報を速やかに被験者に伝え、被験者の治験への参加の継続についての意思を確認する。また、被験者に情報を伝えた日、伝えた情報の内容及びその確認結果を診療録等の原医療記録に記録する。
2) 治験責任医師は、同意文書及びその他の説明文書を改訂する必要があると認めたとき（被験者の同意に関連しうる新たな重要な情報が得られた場合）は、速やかに当該情報に基づき同意文書及びその他の説明文書を改訂し、あらかじめ治験審査委員会の承認を得る。また、治験責任医師等はすでに治験に参加している被験者に対しても速やかに、改訂された同意文書及びその他の説明文書を用いて説明し、治験への参加の継続について被験者から自由意思による同意を文書により得る。ただし、改訂時点で既に治験薬の投与や観察が終了した被験者についての同意は不要とする。また、治験に参加している被験者個人における一般臨床検査値の変動等の治験経過に関する情報についてはこの限りではない。

6. 治験薬

本治験に用いられるすべての治験薬は、治験薬 GMP（「治験薬の製造管理、品質管理等に関する基準（治験薬 GMP）について」平成 20 年 7 月 9 日付け薬食発第 0709002 号）を遵守して製造・取扱い・保管及び管理されている。

6-1. 名称

識別番号：KW-0761
一般名：モガムリズマブ（遺伝子組換え）

6-2. 剤型及び成分

本剤は、1 バイアルにモガムリズマブを 20mg、クエン酸水和物、グリシン、ポリソルベート 80、水酸化ナトリウム、塩酸、注射用蒸留水を含有する静脈注射用製剤である。pH は塩酸又は水酸化ナトリウムにより 5.0～6.0 に調整されている。モガムリズマブの濃度は 4.0mg/mL であり、1 バイアルに 5mL 分注されている。

6-3. 分子式及び分子量

分子式：$C_{6520}H_{10072}N_{1736}O_{2020}S_{42}$

分子量：約 146,443

化学構造式： アミノ酸 219 個の軽鎖 2 分子とアミノ酸 449 個の重鎖 2 分子からなる糖蛋白質

6-4. 貯法

2～8℃、遮光保存

6-5. 有効期間

製造日から 3 年間

6-6. 治験薬の調製

1) バイアルは振盪しないこと。また、激しく攪拌しないこと。

2) 本剤投与時には必要量を注射筒で抜き取り、日局生理食塩液 200mL 又は 250mL に添加する。

3) 調製時には、日局生理食塩液以外は使用しないこと。

4) 添加後は静かに混和し、急激な振盪は避けること。

5) 用時調製し、調製後は速やかに使用すること。また、残液は廃棄すること。

6) 他の薬剤との混注はしないこと。

6-7. 治験薬の包装及び表示

1）薬剤の包装形態

1 箱に 4 バイアルを入れて 1 単位とする。箱は治験薬提供者が封印する。

2）表示

ラベル表示及び包装については、別途定める「治験薬の管理に関する手順書」に記載する。

6-8. 治験薬の提供

治験薬は 1 箱を単位として各実施医療機関へ提供される。

自ら治験を実施する者は、厚生労働大臣に治験計画の届出が受理されるまで、治験薬の提供を受けてはならない。

6-9. 治験薬の管理と未使用治験薬の返却

1）治験薬の保管・管理

治験薬は治験薬提供者により治験計画届書提出後の所定の時期に各実施医療機関の自ら治験を実施する者に提供される。自ら治験を実施する者は所属する実施医療機関の治験薬管理者に治験内容を説明し、別途定める「治験薬の管理に関する手順書」を提出した上で、治験薬の保管・管理を依頼する。治験薬管理者は治験期間中、治験薬の使用の有無に関わらず、治験薬、使用済みバイアル及び外箱を適切に保管・管理するとともに、治験薬の使用状況を把握するために、治験薬管理表を作成する。自ら治験を実施する者は治験薬の管理記録、残薬及び症例報告書の記載内容の整合性について確認し、不整合が判明した場合には、直ちにその原因調査を

行い必要な訂正を行う。

2）未使用治験薬等の返却

治験終了後、治験薬管理者は未使用治験薬、空箱及び使用済みバイアルを自ら治験を実施する者に返却する。返却時には被験者氏名（イニシャル）、カルテ ID 等の被験者のプライバシーに係る事項が判読できないようにする。なお、未使用治験薬、空箱あるいは使用済みバイアルを紛失又は廃棄した場合は、その内容及び理由について記録を作成する。自ら治験を実施する者は、治験薬管理者より返却された全ての未使用治験薬、空箱及び使用済みバイアルを治験調整委員会事務局に提出する。治験調整委員会事務局は全ての自ら治験を実施する者から提出された未使用治験薬、空箱及び使用済みバイアルを適切に廃棄する。

7. 治験方法

7-1. 治験デザイン

【第Ⅰa 相部】Mogamulizumab 0.1 mg/kg 群 3 例（最大 6 例）、その後 0.1 mg/kg 群の忍容性が確認された場合は 0.5mg/kg 群を 3 例（最大 6 例）、0.5mg/kg 群の忍容性が確認された場合は 1.0mg/kg 群 3 例（最大 6 例）について所定の用量の治験薬を週に 1 回 8 週連続で静脈内投与する。

＜用量設定根拠＞

現在 Mogamulizumab の適応となっている ATL では、種々の程度の免疫能の低下が起こっているが、固形がん患者では、通常免疫能は保たれている。このため、固形がん患者では、Mogamulizumab 投与により制御性 T 細胞を除去した場合誘導される免疫反応は ATL より強くなり、腫瘍に対する免疫反応と同時に自己免疫反応も起こることが予想される。

さらに、ATL 患者への Mogamulizumab 投与では、Mogamulizumab は血液中の CCR4 発現腫瘍との反応に主に消費されると考えられるが、固形がん患者では Mogamulizumab は主に少数の制御性 T 細胞除去に費やされると考えられ、ATL 患者への投与時に比して血中濃度が高くなる可能性がある。

また、ATL を対象として実施された第Ⅰ相試験では Mogamulizumab 投与量 0.01mg/kg 群において制御性 T 細胞除去効果が確認されており、その 10 倍量である 0.1mg/kg 群で本対象においても制御性 T 細胞除去効果が得られると考えられる。

これらの理由により、固形がん患者では制御性 T 細胞除去効果による自己免疫疾患の発現リスクが高くなる可能性があり、固形がん患者に対しては、ATL 患者に使用されている投与量である 1.0mg/kg の 1/10 量の 0.1mg/kg を初回投与量として設定し、忍容性を確認しながら dose escalation を行う設定とした。

【第Ⅰb 相部】第Ⅰa 相部において忍容性が確認された投与量（MTD の一段階下の投与量又は MTD に達していない場合は 1.0mg/kg）のなかで高用量の 1 用量及び 0.1mg/kg にて各群 20 例となるように投与する。投与は、週に 1 回 8 週連続で静脈内投与する。ただし、第Ⅰa 相部において、忍容性が確認された投与量が 0.1mg/kg のみであった場合は、第Ⅰb 相部では、0.1mg/kg の

1 用量を用いて 20 例となるように投与する。

＜用量設定根拠＞

本治験により固形がん患者に対する Mogamulizumab 投与の安全性及び制御性 T 細胞効果が確認された場合、今後、Mogamulizumab とは異なる機序により免疫能の抑制を解除する薬剤を併用することも想定される。その場合、Mogamulizumab 単剤投与に比して自己免疫反応が起こる可能性がより高くなると考えられるため、制御性 T 細胞除去効果が確認できる広い用量幅においてデータを取得するために、低用量群を第 Ia 相部で設定されている最低用量である 0.1mg/kg に設定した。

以上より、第 Ib 相部では忍容性が確認された投与群のうち高用量の投与群及び 0.1mg/kg 群で第 Ib 相部を実施する設定とした。

なお、8 回投与後の制御性 T 細胞検査及び抗原特異的抗体免疫反応検査あるいは画像診断において、制御性 T 細胞除去効果及び抗原特異的抗体免疫反応又は RECIST において SD 以上が確認された被験者については、被験者に治験薬の維持投与の希望があり、且つ治験責任医師等が治験薬の維持投与が適当と判断した場合は、12 週以降 4 週毎に、投与中止基準に該当するまで治験薬の維持投与を可とする。

シェーマ

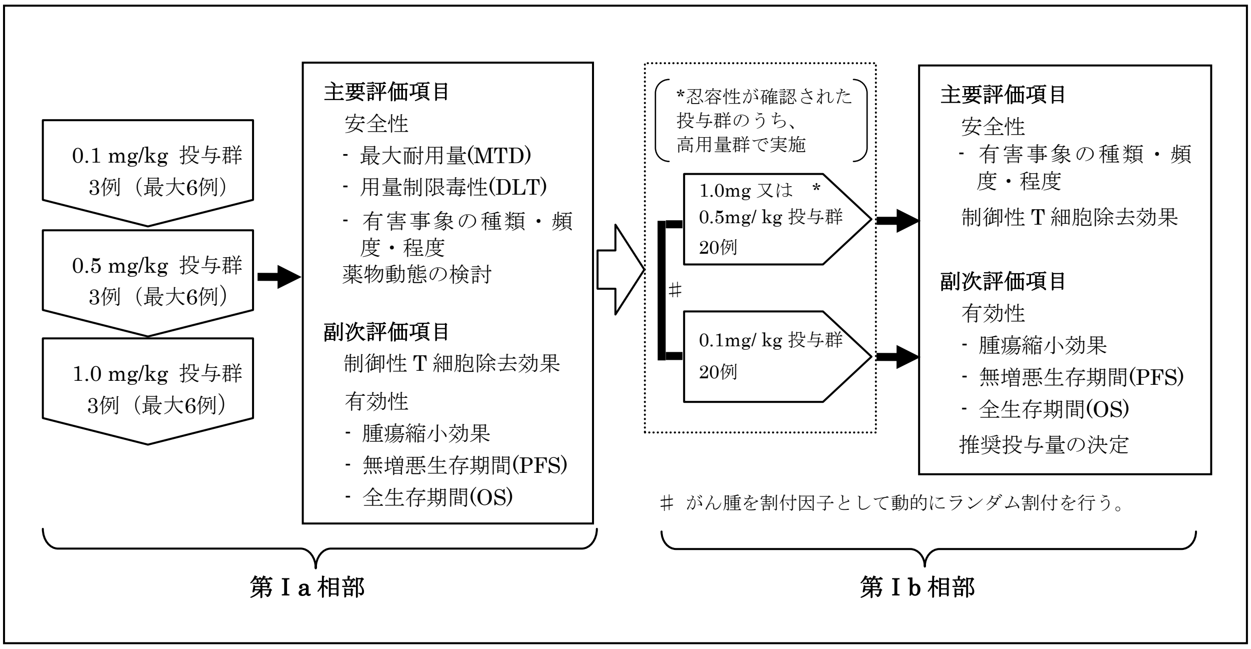

【第 I a 相部】0.1mg/ kg からスタートし、忍容性が確認されれば 0.5mg/ kg 及び 1.0mg/ kg へ dose escalation。

【第 I b 相部】忍容性が確認された投与群のうち高用量群及び 0.1mg/ kg の 2 群（第 I a 相部で 0.5mg/ kg が MTD の場合、0.1mg/ kg 1 群）で実施。各群 20 例となるように登録。

7-2. 第 I a 相部における群間移行及び手順

以下の手順に従って、0.1mg/kg 投与群から開始し、0.5mg/kg 投与群及び 1.0mg/kg 投与群に移

行する。投与群の移行は治験調整委員会において決定する。ただし、必要に応じて効果安全性評価委員会においても評価する。

1）0.1mg/kg 投与群 3 例のうち 1 例も DLT が発現しなかった場合、0.5mg/kg 群に移行する。
0.1mg/kg 投与群 3 例のうち 1 例に DLT が発現した場合、さらに 3 例を 0.1mg/kg 投与群に登録する。0.1mg/kg 投与群のうち 3 例以上に DLT が発現した場合、MTD は 0.1 mg/kg となり、本治験は終了する。DLT の発現が 2 例以下の場合は 0.5mg/kg 投与群に移行する。

2）0.5mg/kg 投与群 3 例のうち 1 例も DLT が発現しなかった場合、1.0mg/kg 群に移行する。
0.5mg/kg 投与群 3 例のうち 1 例に DLT が発現した場合、さらに 3 例を 0.5mg/kg 投与群に登録する。0.5mg/kg 投与群のうち 3 例以上に DLT が発現した場合、MTD は 0.5mg/kg となり、第 Ia 相部は終了する。DLT の発現が 2 例以下の場合は 1.0mg/kg 群に移行する。

3）1.0mg/kg 投与群 3 例のうち 1 例も DLT が発現しなかった場合、MTD は 1.0mg/kg を超える用量となり、第Ｉa 相部は終了する。
1.0mg/kg 投与群 3 例のうち 1 例に DLT が発現した場合、さらに 3 例を 1.0mg/kg 投与群に登録する。1.0mg/kg 投与群のうち 3 例以上に DLT が発現した場合、MTD は 1.0 mg/kg となり、第Ｉa 相部は終了する。DLT の発現が 2 例以下の場合は MTD は 1.0mg/kg を超える用量となり、第Ｉa 相部は終了する。

MTD が決定した場合、MTD に該当する投与群の治験薬投与は中止する。

次の群への移行のための安全性評価（DLT 観察期間）は治験薬投与開始 28 日後までの情報・検査結果にて判断可能とするが、最終的な安全性評価対象期間は各症例、最終投与 24 週間後までの期間とする。

なお、DLT による投与延期を除き DLT 観察期間（治験薬投与開始 28 日後まで）に 2 回以上の投与（投与後 1 週間の評価を含む）が出来なかった場合は、同じ投与群に症例を追加する。

7-3. 第Ｉb 相部への移行

第Ｉa 相部の DLT 観察期間終了後、忍容性が確認された用量のうちで高用量の投与群及び 0.1mg/kg 投与群で第Ｉb 相部を実施する。MTD が 0.5mg/kg 投与群の場合は、0.1 mg/kg 投与群のみで第Ｉb 相部を実施する。第Ｉb 相部への移行は、治験調整委員会において決定する。ただし、必要に応じて効果安全性評価委員会においても評価する。

7-4. 治験実施予定期間

2013 年 2 月～2016 年 3 月

7-5. 登録方法と割り付け

7-5-1. 被験者の登録の手順

各実施医療機関の治験責任医師等は治験審査委員会での承認が得られ、治験計画届を提出した後、候補症例への十分な説明の後、文書にて同意を取得し、適格性を判断するために必要な検査等を実施し、症例登録センターに症例登録を依頼する。症例登録センターは症例の適格性を判定し、適格と判定された被験者にのみ所定の登録番号を割り当て、治験責任医師等に FAX にて報告

を行う。

**KW-0761 症例登録センター**<br>名称：株式会社ファイブリングス<br>TEL ：06-6358-7110<br>FAX：06-6226-7122<br><br>受付時間：月曜日～金曜日　9：00～18：00<br>（土・日曜日、祝日及び年末年始の所定日を除く）

7-5-2. 割り付け

症例登録センターは、第Ⅰa 相部は「7-2. 第Ⅰa 相部における群間移行及び手順」に従い、所定の投与量を被験者に割付ける。また症例登録センターは、第Ⅰb 相部を 2 用量の投与群で実施する場合がん腫内で 2 群の症例数に大きな差が生じないように、がん腫を割付因子とする最小化法を用いて動的にランダム割付を行う。

7-6. 予定登録症例数

7-6-1. 第Ⅰa 相部

0.1mg/kg 群　3 例（最大 6 例）

0.5mg/kg 群　3 例（最大 6 例）

1.0mg/kg 群　3 例（最大 6 例）

計 9 例（最大 18 例）

＜設定根拠＞

用量制限毒性（dose limiting toxicity: DLT）が 2/6(33.3%)を超えない投与量であることを確認しながら ATL 患者に対する投与量である 1.0 mg/kg までの安全性を確認することを目的に、通常の Fibonacci の変法を参考に設定した。

7-6-2. 第Ⅰb 相部

1 群で実施する場合は 20 例

2 群で実施する場合は各群 20 例、計 40 例

＜設定根拠＞

第Ⅱ相試験以降の推奨用量を決定するために、制御性 T 細胞除去効果を検討できる症例数及び症例登録の実現可能性を踏まえて 1 群 20 例として設定した。

7-7. 投与方法

投与する際は、「7-7-1.投与基準」を満たしており、且つ「7-7-2.投与延期基準」及び「7-7-3.投与中止基準」に抵触していないことを確認する。

なお、症例登録後 2 週間以内に治験薬の投与を開始しなければならない。

【第Ⅰa 相部】0.1mg/kg、0.5mg/kg、1.0mg/kg のいずれかの投与量を 1 週間に 1 回 2 時間かけて静脈内投与し、計 8 回投与する。

【第Ⅰb 相部】第Ⅰa 相部で 1.0mg/kg までの忍容性が確認された場合は 0.1mg/kg 又は 1.0mg/kg

のいずれか、0.5mg/kg までの忍容性が確認された場合は 0.1mg/kg 又は 0.5mg/kg のいずれか、0.1mg/kg までの忍容性が確認された場合は 0.1mg/kg を 1 週間に 1 回 2 時間かけて静脈内投与し、計 8 回投与する。

【第Ｉa 相部・第Ｉb 相部共通】

1) 維持投与

9 回目以降の投与は、「7-8.維持投与の実施基準」を満たした場合に実施可能とし、4 週毎に治験薬を維持投与する。8 回投与までと同様に、投与する際は、「7-7-1.投与基準」を満たしており、且つ「7-7-2.投与延期基準」及び「7-7-3.投与中止基準」に抵触していないことを確認する。

2) 投与間隔

8 回投与までは投与間隔の短縮は行わない。ただし、投与を 1 日延期した場合のみスケジュール調整のため次回投与を 1 日短縮することは可能とする。9 回投与以降は、最大 2 週間までの投与延期を可とし、次回投与を最大 2 週間短縮することは可能とする。

3) 投与時の前処置

注入に伴う反応はほぼ全例に見られることが想定されるため、治験薬の投与 30 分前に、ジフェンヒドラミン 30-50mg 及びアセトアミノフェン 300-500mg を内服する。また、ハイドロコーチゾン 100mg を静脈内に前投与する。2 回目の投与以降はジフェンヒドラミン 30-50mg 及びアセトアミノフェン 300-500mg の前投与を行う。投与量については年齢、症状により適宜増減する。

7-7-1. 投与基準

投与当日の投与前*1における血液領域の有害事象が Grade3 以下*2且つ非血液領域の有害事象が Grade2 以下*3であること。

*1 投与当日の投与前に臨床検査が実施できない場合は、投与前 3 日以内の臨床検査でも代用は可能（この期間内に複数回実施した場合は直近の臨床検査）とする。なお、症状及び所見は投与当日の投与前に必ず確認する。

*2 「発熱性好中球減少症」については発現していないこと。また、「リンパ球数減少」及び「CD4 リンパ球減少」は除く。

*3 臨床検査に関しては、AST（GOT）、ALT（GPT）、ALP、総ビリルビン、クレアチニン、カリウムに関するもののみとする。

7-7-2. 投与延期基準

治験薬投与予定日*1 に以下のいずれかの状況であった場合、治験薬の投与を延期する。延期期間は規定の投与予定日より最大 2 週間とする。

1) Grade4 以上の血液領域の有害事象。ただし、「発熱性好中球減少症」は Grade3 以上。
2) Grade3 以上の非血液領域の有害事象が発現した場合（皮膚障害を除く）。ただし、臨床検査に関しては、AST（GOT）、ALT（GPT）、ALP、総ビリルビン、クレアチニン、カリウムに関するもののみとする。
3) 皮膚障害が進行中の場合

4)　被験者が投与延期を申し出た場合
5)　その他、治験責任医師等が投与延期すべきと判断した場合

*1　規定の投与予定日 3 日以内を含む。

7-7-3. 投与中止基準

以下のいずれかの規定に該当する場合は、当該被験者の投与を中止する。

1)　投与延期基準により、規定の投与予定日より 2 週間を超えて投与延期となった場合
2)　24 週目以降において「RECIST の変法の評価において総合効果が PD」であった場合
3)　Grade 3 以上の皮膚障害が発現した場合
4)　治験責任医師等により治験の継続が困難と判断される有害事象が発現した場合
5)　被験者が治験薬の投与中止を申し出た場合
6)　不適格例であることが判明した場合
7)　その他、治験責任医師等が中止すべきと判断した場合

7-8. 維持投与の実施基準

以下のすべての基準を満たす場合は、「7-7-3. 投与中止基準」に該当するまで当該被験者への治験薬の維持投与を許可する。

1)　8 回投与から 6 週間以内に維持投与を開始出来る場合
2)　治験薬投与後、制御性 T 細胞除去効果及び抗原特異的抗体免疫反応又は RECIST において SD 以上が確認された場合
3)　被験者が治験薬の維持投与を希望する場合
4)　治験責任医師等が治験薬の維持投与が適当と判断した場合

7-9. 前治療及び併用療法

治験開始前 4 週間以内に対象疾患に対して前治療（化学療法、放射線療法等）が行われていないことを確認する。腫瘍に対する免疫療法（腫瘍ワクチン等）に関しては症例登録前 12 週間以内に行われていないことを確認する。除外基準に抵触しないことを確認する。

抗がん剤、免疫抑制剤（ステロイド剤を除く）、免疫強化剤、放射線療法、原疾患（転移した腫瘍を含む）に対する手術は治験薬投与期間中、併用禁止とする。副腎皮質ステロイド剤は、治験薬投与のための前処置、合併症及び有害事象の症状改善のための局所投与又は全身投与を許容する。

骨転移例に対するゾレドロン酸やパミドロネートの静脈内点滴、及びデノスマブの皮下注射については、本治験参加前から継続していた場合は、4 週に 1 回の投与であれば併用可とする。ただし、治験薬と同日の投与は避けることとする。

なお、以下のワクチンについては併用に注意する。

１．不活化ワクチン［ワクチンの効果を減弱させる恐れがある（ワクチン接種に対する応答が不明である）］。

２．生ワクチン又は弱毒生ワクチン［接種した生ワクチンの原病に基づく症状が発現した場合には適切な処置を行う（ワクチン接種に対する応答が不明であり、また、生ワクチンによる二次感

染が否定できない）］。

その他合併症及び有害事象に対する治療は可能とする。

未承認医薬品や代替療法（民間療法等）は禁止するが、食品に準じたサプリメントの使用は禁止しない。

7-10. 後治療

投与中止後の治療は制限を設けない。

8. 評価項目

8-1. 主要評価項目

8-1-1. 第Ｉa 相部

1） 安全性：最大耐用量（MTD）、用量制限毒性（DLT）、有害事象の種類・頻度・程度

2） 薬物動態の検討

8-1-2. 第Ｉb 相部

1） 安全性：有害事象の種類・頻度・程度

2） 制御性 T 細胞除去効果

8-2. 副次的評価項目

8-2-1. 第Ｉa 相部

1） 制御性 T 細胞除去効果

2） 有効性：腫瘍縮小効果、無増悪生存期間（PFS）、全生存期間（OS）

8-2-2. 第Ｉb 相部

1） 有効性：腫瘍縮小効果、無増悪生存期間(PFS)、全生存期間(OS)

2） 第Ⅱ相試験以降の推奨投与量の決定

8-3. 評価項目の定義

8-3-1. 主要評価項目の定義

1） 第Ｉa 相部

(1) 安全性

最大耐用量(MTD）、用量制限毒性（DLT）を決定するとともに有害事象の種類・頻度・程度を観察する。有害事象の grading は、Common Terminology Criteria for Adverse Events（CTCAE） v4.0 日本語訳 JCOG 版[13)] を用いる。有害事象は CTCAE v4.0 に従い、判定を行う。

MTD は、治験薬投与開始 28 日後までの期間における DLT 発現被験者数が 3 名以上に認められた投与量とする。MTD の一段階下の投与量を「忍容性が確認された投与量」とする。ただし、いずれの投与量でも MTD に達しなかった場合は 1.0mg/kg を忍容性が確認された投与量とする。

DLT の観察期間は、治験薬投与開始 28 日後までとする。治験薬との因果関係が否定できない有害事象のうち、以下に挙げた事象を DLT とする。

Grade は、CTCAE v4.0 に従い判定する。

① 7 日間以上継続する Grade4 以上の血液毒性*1

*1 「発熱性好中球減少症」は継続期間に関係なく Grade3 以上のもの、「好中球数減少」は Grade3 以下でも G-CSF による治療が実施されたもの。また、「リンパ球数減少」及び「CD4 リンパ球減少」は除外する。

② Grade3 以上の非血液毒性

「注入に伴う反応」と判断された症状（「発熱」、「頭痛」、「疼痛」、「そう痒」及び「アレルギー反応／過敏症」等）は Grade4 以上を DLT とする。また、臨床検査に関しては、AST（GOT）、ALT（GPT）、ALP、総ビリルビン、クレアチニン、カリウムに関するもののみとする。

③ その他

i) ②の「注入に伴う反応」と判断された症状のうちいずれかに Grade3 が認められたことにより、予定された治験薬の投与が規定の投与予定日より 2 週間を超える原因となった毒性

ii) 上記以外に、DLT の観察期間中に同一の事象により治験薬の投与が 2 回以上できなかった毒性

(2) 薬物動態

治験薬を反復投与時の血漿中 Mogamulizumab 濃度から薬物動態パラメータを算出する。

Mogamulizumab の反復投与時の血漿中動態を評価する。血漿中 Mogamulizumab 薬物動態パラメータとして、1、5、8 回目投与後の C_{max}、1、5、7 回目投与後の C_{trough}、1、8 回目投与後の $AUC_{0\text{-}7\,day}$ 及び可能であれば $t_{1/2}$、CL、Vss 及び MRT を算出する。また、反復投与期間中の蓄積率を算出する。血漿中薬物動態パラメータの算出は薬物動態解析ソフト WinNonlin を用いる。

2) 第 I b 相部

(1) 安全性

有害事象の種類・頻度・程度を観察する。有害事象は CTCAE v4.0 に従い、判定を行う。

(2) 制御性 T 細胞除去効果

FCM 解析で CD4 陽性 T 細胞中の CD25 強陽性且つ Foxp3 陽性の細胞集団の比率を測定する。また、リンパ球中の CD4 陽性率を測定し、リンパ球中の比率に換算し、CD4 陽性 CD25 強陽性且つ Foxp3 陽性細胞数を算出する。

制御性 T 細胞除去効果は、CD4 陽性 CD25 強陽性且つ Foxp3 陽性細胞数についてベースラインと比較することにより判定する。

8-3-2. 副次的評価項目の定義

1) 第 I a 相部

(1) 制御性 T 細胞除去効果

「8-3-1. 主要評価項目の定義 2）第 I b 相部」 参照。

(2) 腫瘍縮小効果

① RECIST の評価

腫瘍縮小効果は、RECIST ver.1.1 に従って効果判定する。

② RECIST の変法

RECIST の評価に加え、治験薬投与開始から腫瘍縮小効果を発揮するまでに時間差があることを想定し、標的病変、非標的病変及び新病変を以下のように定義した評価方法（以下、「RECIST の変法」という）でも行う（RECIST（ver.1.1）と異なる箇所に下線を示す）。

i) 標的病変

標的病変は、ベースラインにおいて最長径10mm以上（リンパ節については短径で15mm以上）の測定可能な病変とする。また、治験薬投与開始後に新たに認められた最長径10mm以上（リンパ節については短径で15mm以上）の測定可能病変及び非標的病変が増大し最長径10mm以上（リンパ節については短径で15mm以上）の測定可能病変となったものは標的病変に加えて評価する。標的病変数については制限を設けない。標的病変の評価は、最長径（リンパ節については短径）の和をベースラインと比較し、RECIST（ver.1.1）の標的病変の判定基準にて評価する。

ii) 非標的病変

非標的病変は、ベースラインにおいては、標的病変以外の病変とし、治験薬投与開始後においては、ベースラインで認められた非標的病変のうち、治験薬投与開始後に最長径10mm以上（リンパ節は短径で15mm以上）に増大し、標的病変として評価されるものを除く病変とする。非標的病変の評価はRECIST（ver.1.1）の非標的病変の判定基準にて評価する。

iii) 新病変

新病変は、治験薬投与開始後に認められた骨病変、軟膜病変、腹水、胸水/心膜液、皮膚リンパ管炎、肺リンパ管炎、画像検査で確認できない腹部腫瘤、嚢胞性病変、髄膜炎等の測定不能病変とする。最長径10mm未満（リンパ節は短径で15mm未満）の病変に関しては新病変として取り扱わない。また、治験薬投与開始後に新たに認められた最長径10mm以上（リンパ節については短径で15mm以上）の測定可能病変は、標的病変として評価することから新病変として取り扱わない。

また、12週目までにPDの判定基準を満たした場合でも、12週以降も治験を継続している場合は、RECISTの変法でPDと判定されない限りは、その時点ではPDとして取り扱わない。RECISTの変法のPD確認日は、12週を超えて最初にPDが確認された日とする。ただし、12週を超えた最初の評価がPDであった場合、その時点から評価時期を遡って初めにPDとなった日付をPD確認日とし、遡った評価時期にPDがない場合には12週を超えて最初にPDとなった日をPD確認日として解析する。

③ RECIST の評価、RECIST の変法共通

病状の急激な増悪等の理由により、必要な検査が出来なかった場合は、臨床上増悪が明らかと判断された日をもって PD 確認日とする。PD が確認される前に死亡した

症例については、死亡理由の如何を問わず、治験実施計画書に定められた直近の来院日を PD 確認日とみなす。

なお、CR 又は PR と判定された症例については、画像中央判定を実施する。

＜RECIST の変法について＞

腫瘍縮小効果は、Cancer Vaccine Clinical Trial Working Group の提言に基づき、Immune-related response criteria（irRC） を参考にして作成した RECIST（ver.1.1）の変法を用いて評価する。これは、がんの免疫療法においては実際の臨床効果が発現するまで時間的ずれが生じるので、PD が認められてもこれを容認し、レトロスペクティブに評価する、という方法である。この考えに基づき、本治験では PD の判定基準を満たした場合でも治験を継続し、それ以降臨床効果が認められない場合は日付を遡って PD と判定する。また、腫瘍縮小判定については、治験薬投与開始後に新たな測定可能病変が認められても、腫瘍全体として縮小している場合は、治験薬の効果が示されていると考えられることから、RECIST（ver.1.1）の変法では、ベースライン及び治験薬投与開始後に新たに認められた最長径 10mm 以上（リンパ節については短径で 15mm 以上）の測定可能病変を標的病変として定義し、最長径（リンパ節については短径）の和で評価する。なお、治験薬投与開始後に新たに認められた最長径 10mm 以上（リンパ節については短径で 15mm 以上）の測定可能病変は、標的病変として評価することから新病変として取り扱わない。また、標的病変数については、RECIST（ver.1.1）では、1 臓器につき最大 2 ヶ所、合計 5 病変までとしているが、RECIST（ver.1.1）の変法では、治験薬投与開始後に新たに認められた最長径 10mm 以上の病変を標的病変と捉えベースラインに加え評価することとしているため、RECIST（ver.1.1）で規定された標的病変数を超えることが想定される。従って、標的病変数については制限を設けない。

(3) 無増悪生存期間

無増悪生存期間（PFS）は、治験薬投与開始日から治験期間中の PD 確認日までの期間とする。病状の急激な増悪等の理由により、必要な検査が出来なかった場合は、臨床上増悪が明らかと判断された最初の日をもって PD 確認日とする。PD が確認される前に死亡した症例については、死亡理由の如何を問わず、治験実施計画書に定められた直近の来院日を PD 確認日とみなす。

(4) 全生存期間

全生存期間（OS）は、治験薬投与開始日からあらゆる原因による死亡までの期間とする。死亡が確認されずに生存した症例では最終生存確認日、追跡不能となった症例では、最後に生存が確認された日をもって打ち切りとする。

2) 第Ｉb 相部

(1) 腫瘍縮小効果

「8-3-2. 副次的評価項目の定義　1）第Ｉa 相部」参照。

(2) 無増悪生存期間

「8-3-2. 副次的評価項目の定義　1）第Ｉa 相部」参照。

(3) 全生存期間

「8-3-2. 副次的評価項目の定義　1）第Ⅰa 相部」参照。

(4) 第Ⅱ相試験以降の推奨用量の決定

第Ⅱ相試験以降の推奨用量は、第Ⅰb 相部の安全性及び制御性 T 細胞除去効果等を総合的に検討して決定する。

8-3-3. その他の項目の定義

1）　第Ⅰa 相部及び第Ⅰb 相部共通

(1) 抗原特異的抗体免疫反応検査（NY-ESO-1 及び XAGE-1b）

血清中の NY-ESO-1 あるいは XAGE 抗原に対する抗体価を、ELISA 法により組換え NY-ESO-1 タンパクあるいは合成 XAGE タンパクを用いて測定する。

IgG 価が健常人血清での ELISA 吸光度の平均値 ＋3 S.D.を超えるものを陽性とし、陽性を示す最大希釈倍数が変動した場合に「上昇」または「低下」と定義する。

(2) 制御性 T 細胞の評価（任意）

制御性 T 細胞のマーカーである CD3、CD4、CD25、CD45RA 等を用いて細胞表面染色する。加えて転写因子 Foxp3 等で細胞内染色し、FCM を用いて解析する。制御性 T 細胞のなかでも抑制機能が強く抗腫瘍免疫応答を抑制している分画を、死細胞等を慎重に除去したのち、正確に同定する。またこの分画は、CCR4 を強発現しているため、Mogamulizumab 投与によるこの分画の変化を検討する。

(3) 抗原特異的抗体免疫反応検査（NY-ESO-1 及び XAGE-1b 以外の抗原）（任意）

抗原特異的な免疫反応の増強効果を評価するため、血清を用いた ELISA を行う。腫瘍抗原 MAGE-A1、MAGE-A3、MAGE-A4、CT7/MAGEC1、CT10/MAGEC2、CT45、CT46/HORMAD1、SOX2、SSX2、p53 等の抗原タンパクに対する反応を測定する。

(4) 抗原特異的細胞免疫反応検査（任意）

抗原特異的な免疫反応の増強効果を評価するため、末梢血を用いた抗原特異的 CD4・CD8T 細胞反応を計測する。対象とするがん腫に 20－50%発現している NY-ESO-1、XAGE-1b に対する反応を観察するため、CD4・CD8T 細胞と、NY-ESO-1 あるいは XAGE-1b タンパクを付加した抗原提示細胞を共培養し、IFN-γ産生細胞を IFN-γ分泌アッセイにて検出する。

(5) サイトカイン産生能評価（任意）

Mogamulizumab 投与による制御性 T 細胞の変化によって、エフェクター細胞側が抗原刺激により受ける影響について、IL-2、TNF-α、IFN-γ等のエフェクターサイトカイン多重産生能を細胞内染色により評価する。抗原刺激後、トランスポーター阻害剤を用いて、細胞内にサイトカインを捕縛し、Live/Dead staining kit、抗ヒト CD3、CD4、CD8、CD45 抗体で細胞表面を染色したのち固定し、ヒト IFN-γ抗体、ヒト TNF-α抗体、ヒト IL-2 抗体で細胞内染色する。また、必要に応じて、抗原特異的 T 細胞のクローンを樹立し、HLA 拘束性、エピトープ解析を行う。さらにテトラマーを用い抗原特異的 T 細胞のモニタリングを行う。

(6) 免疫担当細胞・制御細胞解析（任意）

制御性T細胞、MDSC等の免疫制御細胞、ヘルパーT細胞分画（Th1、Th2、Th17）及び、単球や樹状細胞等の割合をFCMにより経時的に解析する。免疫抑制細胞の解析において、制御性T細胞は抗ヒトCD4、CD8、CD25、CD45RA抗体で染色し、MDSCは抗ヒトCD3、CD11b、CD14、CD15、CD33、CD45抗体、HLA-DR抗体で細胞表面を染色する。

また、免疫増強因子であるICOS、OX-40、GITR、4-1BB、及び免疫抑制因子であるPD-1、BTLA、TIM-3、2B4等の発現を各々の抗体を用いてFCMにより経時的に解析する。

(7) 免疫組織染色（任意）

免疫関連細胞の生体内分布等を検索するため、過去の手術摘出組織等、または通常診療の一環としての内視鏡検査時等組織生検検体を用いる。なお内視鏡検査等にあたり、可能な場合は通常の診断に必要な生検に加えて、腫瘍部から 1-2 箇所を追加的に採取することが望ましい。

採取された検体はいずれも各施設での病理診断に用いられる。診断後に保管されているブロックから未染色標本を作製し、免疫関連分子を免疫染色法にて解析する。

(8) 遺伝子解析（任意）

Mogamulizumab の臨床効果や毒性（自己免疫性有害事象：皮疹、間質性肺炎等）の発症を予測しうるゲノムバイオマーカーを探索的に検討するため、治療開始前の末梢血単核球由来の DNA を用いた遺伝子多型解析とコピー数多型解析を用いた Genome-wide association study(GWAS)、及び血清中miRNAの網羅的発現解析を行う。

9. 観察項目・方法

9-1. 治験スケジュール

次のスケジュール表 1 に従い、所定の観察・検査を実施する。なお、初回投与前から投与翌日まで入院する。

治験責任医師等は第Ia相部の被験者については5回目の投与に先立ち、治験の継続について再度同意を得ることとする。

8回投与後に制御性T細胞除去効果及び抗原特異的抗体免疫反応又はRECISTにおいてSD以上が確認された被験者については、被験者に治験薬の維持投与の希望があり、且つ治験責任医師等が治験薬の維持投与が適当と判断した場合は、治験薬の維持投与を可能とする。

初回の治験薬投与日を 1 週と定義する。

表 1. 治験スケジュール

	ベースライン	1 週 d1 入院 ←→	2 週 d8	3 週 d15	4 週 d22	5 週 d29	6 週 d36	7 週 d43	8 週 d50	9 週 d57	10 週 d64	12 週 d78	13 週以降 (12 週又は 9 回目規定来院日から n 週毎)	投与中止時	治験中止時
同意取得	●														
Mogamulizumab*1 (投与回数)		● (1)	● (2)	● (3)	● (4)	● (5)	● (6)	● (7)	● (8)			○ (9)	○ 4 週毎		
再同意取得						●									
問診*2	●	●	●	●	●	●	●	●	●	●		●	○ 4 週毎	●	●
PS*3	●	●	●	●	●	●	●	●	●	●		●	○ 4 週毎	●	●
体重*3	●		●	●	●	●	●	●	●	●		●	○ 4 週毎	●	●
Vital check*4 (血圧・脈拍数, 体温, 呼吸数)	●	●	●	●	●	●	●	●	●	●		●	○ 4 週毎	●	●
一般血液検査*3*5*6	●		●	●	●	●	●	●	●	●		●	○ 4 週毎	●	●*11
甲状腺機能検査*3*5	●											●		●	●
腫瘍マーカー*3*5（任意）	●											●	● 12 週毎		
尿検査*3	●		●									●	○ 12 週毎	●	●
CT 又は MRI	●											●	● 12 週毎		●*11
腫瘍抗原発現検査 (NY-ESO-1、XAGE-1b)	●														
HIV、HCV、HBV 検査	●														
CCR4 発現検査	●														
抗原特異的抗体免疫反応検査*3*5 (採血量 mL)	● (5)					● (5)				● (5)			● 4 週毎 (5)	● (5)	●*11 (5)
制御性 T 細胞検査*3*5 (採血量 mL)	● (10)					● (10)				● (10)			● 4 週毎 (10)	● (10)	●*11 (10)
薬物動態検査*5*7 (採血量 mL)		● (2×4)	● (2)			● (2)	● (2)		● (2×4)	● (2)	● (2)	● (2)			
免疫増強効果検査*3*5*8 (任意) (採血量 mL)	●*9 (50×2)					● (30)		● (40)		● (30)		● (40)	● 4 週毎 (50)	● (50)	●*11 (50)
遺伝子解析*3*5（任意） (採血量 mL)	● (15)														
有害事象*10		←→													→

○：8 回投与後に維持投与が行われた際に追加で実施する項目

*1：9 回目以降の治験薬の投与は、制御性 T 細胞除去効果及び抗原特異的抗体免疫反応又は RECIST において SD 以上が確認され、被験者に治験薬の維持投与の希望があり、且つ治験責任医師等が治験薬の維持投与が適当と判断した場合に限る。

*2：治験薬投与開始前～終了まで継続的に観察する。

*3：治験薬投与期間中は治験薬投与前に実施する。

*4：治験薬投与前、投与開始 30 分、1 時間、1.5 時間、投与終了時、投与終了後 30 分後、1 時間後に実施する。

*5：貧血の兆候が認められた場合等で規定量の採血が困難な場合は、被験者の安全性を考慮し、1.一般血液検査(施設の規定量)、2.甲状腺機能検査(施設の規定量)、3.制御性 T 細胞検査(10mL)、4.薬物動態検査(第 I a 相部のみ、2mL)、5.抗原特異的抗体免疫反応検査(5mL)、6.遺伝子解析(15mL)、7.免疫増強効果検査(30~50mL)、8.腫瘍マーカー(施設の規定量)の優先順位で可能な範囲の採血を行う。

*6：治験薬最終投与後 12 週目にも検査を実施する。

*7：1 回あたりの採血量は 2mL とする。実施時期については表 2 を参照のこと。

*8：「8-3-3 その他の項目の定義」（2）、（4）～（6）に示した検査項目とする。

*9：複数日に分けて採血を行うことを可とする。

*10：有害事象は治験薬の最終投与から 12 週後まで観察する。ただし、Grade3 以上の非血液毒性に関しては 24 週後までを有害事象の観察期間とする。

*11：8 回投与後に維持投与が行われず、12 週の検査実施後に治験中止した場合、治験中止時には一般血液検査、CT 又は MRI、抗原特異的抗体免疫反応検査、制御性 T 細胞検査及び免疫増強効果検査のみ実施する。

表 2. 薬物動態検査の採血スケジュール（第 Ia 相部のみ実施）

採血時期＼投与回数	初回投与	2 回目	3 回目	4 回目	5 回目	6 回目	7 回目	8 回目
投与前	●	●				●		●
投与終了時／投与中止時	●				●			●
24 時間後（1 日後）	●							●
72 時間後（3 日後）	●							●
168 時間後（7 日後）								●
14 日後								●
28 日後								●

9-2. 患者背景

以下の項目について確認し、症例報告書に記載する。

同意取得日、生年月日、性別、身長、原疾患（原疾患名、病型又は組織型、診断年月日、診断方法、TNM 分類、病期分類）、原疾患に対する治療歴、既往歴*、合併症**、腫瘍抗原(NY-ESO-1、XAGE-1b)発現の有無、HIV 検査***、HCV 検査***、HBs 抗原検査***、HBc 抗体検査***、HBs 抗体検査***、HBV-DNA 検査（HBc 抗体検査又は HBs 抗体検査結果が陽性の場合に実施）***、CCR4 発現検査（IHC）、動脈血酸素飽和度、心電図、左心駆出率、妊娠検査***（hCG 検査：閉経前女性に限る）

＊：治験薬投与開始 1 年前から症例登録までに治癒した臨床的に問題となる疾患、及び治験開始 1 年以上前の既往でも患者背景として重要と判断された疾患
＊＊：症例登録の時点で治癒していない疾患
＊＊＊：症例登録前 4 週間以内に実施する。

9-3. 治験薬の投与及び併用治療の状況

1) 治験薬の投与状況

治験薬の投与日時を症例報告書に記載する。なお、投与を中止した場合はその理由を症例報告書に記載する。

2) 併用治療の状況

1 週から治験薬投与終了時又は投与中止時（最終投与 4 週後）まで被験者に投与された全ての併用薬及び併用療法について、1 日投与量、投与経路、開始日及び終了日、投与目的を症例報告書に記載する。

9-4. 有効性に関する項目

9-4-1. 抗原特異的抗体免疫反応検査（NY-ESO-1 及び XAGE-1b）

ベースライン、5、9 週、12 週以降は可能な限り 4 週毎、維持投与時は 9 回目投与日より 4 週毎に、また、治験中止時に採血を行い、外部検査機関で測定する。ただし、治験薬投与日につい

ては、治験薬の投与前に採血を実施する。治験薬の投与を中止した場合は投与中止時に採血を行い、中止後も可能な限り 4 週毎に採血を実施する。測定結果は外部検査機関よりデータセンターに直接提出される。

9-4-2. 制御性 T 細胞検査（FOUR-COLOR FOXP3 検査、CD4/CD25/CCR4 検査及び MLA CD45 ゲーティング）

末梢血を用いて、免疫学的モニタリングにより活性化制御性 T 細胞数の減少を評価する。このため、ベースライン、5、9 週、12 週以降は可能な限り 4 週毎、維持投与時は 9 回目投与日より 4 週毎に、また、治験中止時に採血を行い、外部検査機関で測定する。ただし、治験薬投与日については、治験薬の投与前に採血を実施する。治験薬の投与を中止した場合は投与中止時に採血を行い、中止後も可能な限り 4 週毎に採血を実施する。測定結果は外部検査機関よりデータセンターに直接提出される。

9-4-3. CT 又は MRI

ベースライン及び 12 週目に測定する。その後は 12 週毎に実施する。また、治験薬の投与中止後も可能な限り 12 週毎に実施し、治験中止時に測定する。

撮影部位は、標的病変を含む箇所とする。CT はヘリカル CT 機を使用し、スライス厚は 5mm で撮影する。CT 又は MRI 検査は可能な限り実施医療機関毎に同一機種、各被験者の撮像条件は毎回同一条件で行うこととする。

また、RECIST の変法で CR 又は PR と判定された症例については、第三者機関である画像中央判定委員会により客観的且つ統一的に判定される。

治験責任医師等は、CR 又は PR と判定した症例の画像データを別途定める「画像中央判定に係る手順書」に従い適切な時期に画像中央判定委員会に提出する。なお、治験責任医師等と画像中央判定委員会の判定が異なる場合は、画像中央判定委員会の判定を最終評価とする。

9-4-4. 生存期間調査及び後治療

維持投与を実施しなかった症例、及び維持投与を実施したが中止した症例について「14-3. 治験の終了」に示す治験終了までの期間、生存期間を調査する。死亡した場合、死亡日及びその理由を症例報告書に記載する。

なお、後治療が行われた場合、その治療内容、実施期間、効果及び奏効期間を調査する。

9-4-5. 腫瘍マーカー（任意）

ベースライン及び 12 週目に測定する。その後は 12 週毎に測定する。また、治験薬の投与中止後も可能な限り 12 週毎に実施する。測定結果を症例報告書に記載する。

ただし、腫瘍マーカーの測定の実施及び測定する場合の腫瘍マーカーの項目は任意とする。

9-4-6. 制御性 T 細胞の評価（任意）

末梢血及び腫瘍組織中の単核球を分離し、免疫学的モニタリングにより活性化制御性 T 細胞数の減少を評価する。このため、ベースライン、5、9 週、12 週以降は可能な限り 4 週毎、維持投

与時は 9 回目投与日より 4 週毎に、また、治験中止時に採血を行い、外部検査機関で測定する。ただし、治験薬投与日については、治験薬の投与前に採血を実施する。治験薬の投与を中止した場合は投与中止時に採血を行い、中止後も可能な限り 4 週毎に採血を実施する。腫瘍組織については通常診療の一環としての内視鏡検査時等組織生検時に、1-2 個の組織片を追加採取する。測定結果はデータセンターに直接提出される。

9-4-7. 抗原特異的抗体免疫反応検査（NY-ESO-1 及び XAGE-1b 以外の抗原）（任意）

ベースライン、5、9 週、12 週以降は可能な限り 4 週毎、維持投与時は 9 回目投与日より 4 週毎に、また、治験中止時に採血を行い、外部検査機関で測定する。ただし、治験薬投与日については、治験薬の投与前に採血を実施する。治験薬の投与を中止した場合は投与中止時に採血を行い、中止後も可能な限り 4 週毎に採血を実施する。なお、本検査の採血に際しては、9-4-1.抗原特異的抗体免疫反応検査（NY-ESO-1 及び XAGE-1b）と併せて実施する。測定結果は外部検査機関よりデータセンターに直接提出される。

9-4-8. 抗原特異的細胞免疫反応検査（任意）

ベースライン、7、12 週、12 週以降は可能な限り 4 週毎、維持投与時は 9 回目投与日より 4 週毎に、また、治験中止時に採血を行い、外部検査機関で測定する。ただし、治験薬投与日については、治験薬の投与前に採血を実施する。治験薬の投与を中止した場合は投与中止時に採血を行い、中止後も可能な限り 4 週毎に採血を実施する。測定結果は外部検査機関よりデータセンターに直接提出される。

9-4-9. サイトカイン産生能評価（任意）

ベースライン、7、12 週、12 週以降は可能な限り 4 週毎、維持投与時は 9 回目投与日より 4 週毎に、また、治験中止時に採血を行い、外部検査機関で測定する。ただし、治験薬投与日については、治験薬の投与前に採血を実施する。治験薬の投与を中止した場合は投与中止時に採血を行い、中止後も可能な限り 4 週毎に採血を実施する。測定結果は外部検査機関よりデータセンターに直接提出される。

9-4-10. 免疫担当細胞・制御細胞解析（任意）

ベースライン、7、12 週、12 週以降は可能な限り 4 週毎、維持投与時は 9 回目投与日より 4 週毎に、また、治験中止時に採血を行い、外部検査機関で測定する。ただし、治験薬投与日については、治験薬の投与前に採血を実施する。治験薬の投与を中止した場合は投与中止時に採血を行い、中止後も可能な限り 4 週毎に採血を実施する。測定結果は外部検査機関よりデータセンターに直接提出される。

9-4-11. 免疫組織染色（任意）

過去の手術摘出組織等、または通常診療の一環としての内視鏡検査時等組織生検検体を染色に用いる。なお内視鏡検査等にあたり、可能な場合は通常の診断に必要な生検に加えて、腫瘍部から 1-2 箇所を追加的に採取することが望ましい。

採取された検体はいずれも各施設での病理診断に用いられる。診断後に保管されているブロックから未染色標本を作製する。染色結果は外部検査機関よりデータセンターに直接提出される。

9-4-12. 遺伝子解析（任意）

ベースライン(治験薬の投与開始前まで)に採血を行い、外部検査機関で測定する。測定結果は外部検査機関よりデータセンターに直接提出される。

9-5. 安全性に関する項目

9-5-1. PS（Performance Status）

ベースライン、治験薬投与毎、第 9 週及び 12 週に観察する。また、投与中止時及び治験薬投与期間中の治験中止時に観察する。ただし、治験薬投与日については、治験薬の投与前に観察を実施する。なお、スコアは Performance Status Scale/Scores ECOG の定義に従うものとする。

9-5-2. バイタルサイン(血圧・脈拍数、体温、呼吸数)

ベースライン及び治験薬投与毎に、投与前、投与 30 分後、1 時間後、1.5 時間後、投与終了時、投与終了 30 分後、1 時間後に観察する。また、第 9 週、12 週、投与中止時及び治験薬投与期間中の治験中止時に観察する。観察結果を症例報告書に記載する。

9-5-3. 体重

ベースライン、治験薬投与毎、第 9 週及び 12 週に測定する。また、投与中止時及び治験薬投与期間中の治験中止時に測定する。ただし、治験薬投与日については、治験薬の投与前に測定を実施する。測定結果を症例報告書に記載する。

9-5-4. 一般血液検査

ベースライン、治験薬投与毎、第 9 週及び 12 週に検査する。また、投与中止時及び治験中止時に検査する。また、最終投与後 12 週目に検査を実施する。ただし、治験薬投与日については、治験薬の投与前に実施する。検査結果を症例報告書に記載する。検査項目は以下のものとする。

1) 血液学的検査

赤血球数、ヘモグロビン、ヘマトクリット、白血球数、白血球分画、血小板数

2) 血液生化学的検査

AST（GOT）、ALT（GPT）、ALP、LDH、総蛋白、アルブミン、総ビリルビン、尿素窒素、クレアチニン、尿酸、ナトリウム、カリウム、クロール、カルシウム、リン

9-5-5. 甲状腺機能検査

ベースライン、第 12 週、投与中止時及び治験薬投与期間中の治験中止時に検査する。ただし、治験薬投与日については、治験薬の投与前に実施する。検査結果を症例報告書に記載する。検査項目は以下のものとする。

FT3、FT4、TSH

9-5-6. 尿検査

ベースライン、2、12 週に実施する。維持投与時には 9 回目投与日より 12 週毎に検査を実施する。ただし、治験薬投与日については、治験薬の投与前に実施する。また、投与中止時及び治験薬投与期間中の治験中止時に検査する。検査結果を症例報告書に記載する。検査項目は以下のものとする。

1)　尿定性

蛋白、糖、ウロビリノゲン、潜血

9-6. 薬物動態（第Ｉa 相部）

初回投与前、投与終了時、投与終了後 24 時間、72 時間、168 時間（7 日間後、2 回目投与前）、5 回目投与終了時、投与終了後 168 時間（7 日間後、6 回目投与前）、8 回目投与前、投与終了時、投与終了後 24 時間、72 時間、168 時間（7 日間後）、14 日間後、28 日間後に採血を行い、外部検査機関で測定する。測定結果は外部検査機関よりデータセンターに直接提出される。

10. 安全性に関する取扱い

10-1. 定義

10-1-1. 臨床検査値の異常及び異常変動の定義

血液検査及び尿検査は、各項目の測定値毎に正常・異常の判定を行った上、ベースラインと比較し、異常変動の有無を判定する。正常範囲が設定されていない場合は、治験責任医師等が設定する。

治験責任医師等は、初回投与前の臨床検査値と投与後に実施された臨床検査値から、異常変動の有無を判断する。異常変動ありと判断した場合は、臨床検査値の有害事象として扱い、症例報告書に記入する。

臨床検査値と同様に、体重、バイタル等 についても異常変動の有無を判断し、異常変動ありと判断した場合は有害事象として扱い、症例報告書に記入する。

10-1-2. 有害事象及び副作用（因果関係が否定できない有害事象）の定義

有害事象とは、治験薬を投与された被験者に生じた、あらゆる好ましくない医療上のできごとをいう。すなわち、治験薬が投与された際に起こる、あらゆる好ましくない又は意図しない徴候（臨床検査値の異常変動を含む）、症状又は病気のことであり、当該治験薬との因果関係の有無は問わない。また、副作用（因果関係が否定できない有害事象）とは、治験薬との因果関係が「関係なし」と判定された事象以外のすべての有害事象とする。

病気（診断名）に随伴する徴候、症状及び臨床検査値の異常変動は、診断名を有害事象として扱う。ただし、その病気において非典型的な徴候、症状や極端に重い徴候、症状が発現した場合は診断名と同様に個々の徴候、症状もあわせて有害事象として扱う。

有害事象の観察期間は、治験薬初回投与日から最終投与 12 週後までとする。ただし、Grade3 以上の非血液毒性に関しては 24 週後までを有害事象の観察期間とする。

10-1-3. 重篤な有害事象の定義

「重篤な有害事象」とは次の場合をいう。

1) 死亡

2) 死亡につながるおそれのある事象

3) 治療のために入院又は入院期間の延長が必要とされる事象

4) 障害

5) 障害につながるおそれのある事象

6) 1)～5)に掲げる事象に準じて重篤である事象

7) 後世代における先天性の疾病又は異常

なお、以下の場合は、重篤な有害事象と扱わない。

・治験薬投与のために"入院又は入院期間の延長"が生じた場合

10-1-4. 症状及び所見に関する有害事象

治験薬初回投与後に新たに発現した症状及び所見、又は治験薬初回投与前に認められていた症状及び所見の中で、治験責任医師等が治験薬初回投与後に悪化したと判断したものを症状及び所見の有害事象とし、症例報告書に記入する。

ただし、PS の変動及び新病変の出現は有害事象として扱わない。

原疾患の悪化及びそれに起因する併発症は上記の「10-1-3. 重篤な有害事象の定義」に該当する場合は「10-3-1. 重篤な有害事象報告手順」に従い、報告対象とする。死亡の場合はその原因が原疾患の悪化によるものなのか、その他の理由、例えば治験薬によるものなのかを判定する。

10-2. 調査項目

10-2-1. 有害事象名

症状及び所見に関する有害事象の場合は症状又は診断名を、臨床検査値に関する有害事象の場合は検査項目名又は変動の詳細を症例報告書に記入する。

10-2-2. 発病（発現）日及び最悪化日

発現時及び最悪化時の日付、Grade を記入する。（最悪化日は最悪 Grade を最初に確認した日付）。急性輸注反応又はサイトカイン放出症候群の発現時及び最悪化時に対しては時刻についても記入する。

10-2-3. 重症度

CTCAE v4.0 に準じて Grade を判定し、症例報告書に記載する。

10-2-4. 重篤度

下記のコードから選択する。

1) 重篤

2） 非重篤

10-2-5. 処置

1） 治験薬の処置

下記のコードから選択する。

(1) 変更せず

(2) 延期

(3) 中止

(4) 該当せず

2） その他の処置（処置方法を記入する）

治験薬以外の処置を下記のコードから選択する。

(1) なし

(2) 併用薬減量

(3) 併用薬増量

(4) 他剤処方

(5) その他

10-2-6. 転帰

有害事象の最終観察期間から 4 週後（治験薬の最終投与日から 16 週後（Grade3 以上の非血液毒性は 28 週後））までに確認した転帰を記載する。なお、副作用（因果関係が否定できない有害事象）に関しては回復又は軽快と確認されるまで追跡調査を実施する。

1） 回復（治験薬初回投与前と同じ程度に戻った場合）

2） 軽快（最悪は脱したが、治験薬初回投与前と同じ程度に戻っていない場合）

3） 未回復（最悪の程度が継続した場合）

4） 後遺症あり（当該有害事象は回復したが、それに関連した別の症状が残った場合）

5） 死亡（有害事象により死亡した場合）

6） 不明（不明な場合）

10-2-7. 転帰日

有害事象の最終観察期間から 4 週後（治験薬の最終投与日から 16 週後（Grade3 以上の非血液毒性は 28 週後））までに確認した転帰日及び Grade も記入する。急性輸注反応又はサイトカイン放出症候群に対しては時刻についても記入する。

10-2-8. 治験薬との因果関係

治験薬投与との関連性を判定する場合は、以下の基準に従う。

治験責任医師等が関連性を「関連なし」と判断した場合は、その理由を症例報告書に記載する。

＜治験薬との関連性判定基準＞

1) 関連なし

入手した情報の範囲内で、合理的な可能性（治験薬投与と有害事象発現との時間的経過、薬理作用等）がなく、原病の悪化等他に原因があると判断でき、当該治験薬に起因するものでないと判断できた場合。

2) 関連あるかもしれない

慎重な医学的評価の結果､当該事象と治験薬投与との関係に確実性を有するか又は強く疑うほどではないが、因果関係の存在を全く除外することが出来ない場合。

3) おそらく関連あり

慎重な医学的評価の結果、当該事象と治験薬投与との関係に高い確実性がある場合。

4) 関連あり

当該事象の発生が治験薬投与の結果であることに疑いの余地がない場合。

10-3. 有害事象の報告と対応

報告義務のある有害事象は、「10-1-2. 有害事象及び副作用（因果関係が否定できない有害事象）の定義」に規定された有害事象の観察期間中に発現したもののうち、「10-1-3. 重篤な有害事象の定義」に定義された「重篤な有害事象」とする。当該有害事象発生時には、「10-3-1. 重篤な有害事象報告手順」に従い報告する。

また、重篤な有害事象ではないが、第Ｉa 相部においては、因果関係の有無に関わらず DLT の基準に合致する有害事象（8-3-1.主要評価項目の定義）が発生した場合は、治験責任医師等は適切な処置を行い、「用量増加に関わる有害事象に関する報告書」を作成し、速やかに治験調整委員会に提出する。

10-3-1. 重篤な有害事象報告手順

1) 速報（発生を知った時点から 24 時間以内）

報告義務のある有害事象が発生した場合、治験責任医師等は治験薬との因果関係の有無に関わらず、適切な処置を行い、実施医療機関の長に報告するとともに、直ち（発生を知った時点から 24 時間以内）に「（医）書式 12-1 重篤な有害事象に関する報告書」にその時点までに把握できている情報を記載して治験調整委員会に報告する。

2) 詳細報（発生を知った時点から 3 日以内）

治験責任医師等は、重篤な有害事象の発生を知った時点から 3 日以内に「（医）書式 12-1、2 重篤な有害事象に関する報告書」を作成し、実施医療機関の長に報告するとともに、治験調整委員会に報告する。その際、治験薬と有害事象との関連性を十分考察する。ただし、該当する有害事象が未知の治験薬との因果関係が否定できない致死的及び生命を脅かす重篤な有害事象であった場合は、詳細な報告書を直ちに（遅くとも翌日）に治験調整委員会に提出するものとする。なお、治験調整委員会は直ちに他の実施医療機関の治験責任医師及び治験薬提供者に通知する。

3) 対応

自ら治験を実施する者は、治験調整委員会の調整の下、対応を検討し必要な措置を講ずる。

なお、治験薬の投与又は治験を中止した場合は、治験調整委員会に速やかに報告しなければならない。

また、治験調整委員会は効果安全性評価委員会に審議を依頼し、治験継続の可否等について決定する。

10-3-2. 効果安全性評価委員会による評価・勧告

評価を依頼された効果安全性評価委員会は、「重篤な有害事象に関する報告書」の内容を検討し、必要な場合には、治験調整委員会と協議の上、詳細調査の指示を行う。

効果安全性評価委員会は、提出されたすべての情報に基づき評価を行い、治験調整委員会に対して対策の必要性及びその内容に関する勧告を行う。考えられる対策としては、治験の中止、登録の中断・中断継続・再開、治験実施計画書変更、同意説明文書の変更、治験薬のロット検査、全実施医療機関への有害事象情報の提供、治験審査委員会による審査等がある。

10-3-3. 対策の決定

自ら治験を実施する者は、効果安全性評価委員会からの勧告を踏まえて、対策の必要性及びその内容について決定を行う。自ら治験を実施する者は、必要に応じて決定事項を効果安全性評価委員会、治験審査委員会、に連絡する。他施設から連絡を受けた場合、自ら治験を実施する者は、必要に応じて治験審査委員会に対策の内容とその理由を連絡する。

10-4. 予想される副作用

1)　皮膚障害

CCR4 陽性の再発・再燃 ATL 患者及び PTCL 患者を対象とした国内第 I 相臨床試験（試験番号 : 0761-0501 試験）、CCR4 陽性の再発・再燃 ATL 患者を対象とした国内第Ⅱ相臨床試験（試験番号：0761-002 試験）、CCR4 陽性の ATL 初発未治療患者を対象とした国内後期第 II 相臨床試験（試験番号：0761-003 試験）、CCR4 陽性の再発・再燃末梢性 T/NK 細胞リンパ腫患者を対象とした国内後期第 II 相臨床試験（試験番号：0761-004）及び CCR4 陽性の再発・難治性 PTCL 患者を対象とした欧州第 II 相臨床試験（試験番号：0761-007）において、重篤な副作用として「発疹」が 7 名、「乾癬」、「全身性皮疹」、「斑状皮疹」、「スティーブンス・ジョンソン症候群」、「全身紅斑」、「剥脱性発疹」、「中毒性皮疹」が各 1 名に発現した。0761-002 試験実施中に、皮膚科専門医 2 名の出席による皮膚症状症例検討会を開催し助言を求めたところ、「スティーブンス・ジョンソン症候群」はサイトメガロウイルスにより引き起こされた可能性があるが、「スティーブンス・ジョンソン症候群」以外の皮膚症状の有害事象は Mogamulizumab の薬理作用が引き起こしているとの見解であった。これらの皮膚症状は、皮疹発現後に投与を継続してもそれほど重篤な症状とはならず、ステロイド内服でコントロールが可能あるいは自然回復するものもあるため、通常の薬疹ではないと考えられるとの結論を得た。ATL という予後不良な疾患に対して、Mogamulizumab 投与により奏効を認めている点は患者にとって大きなメリットであり、Mogamulizumab による皮膚症状の有害事象はその効果と比較較量すればそれほど有害なものとは思われないとの見解が得られた。しかしながら、Mogamulizumab 投与による皮膚症状発現時には十分な注意と適切な処置を行うとともに、皮

膚症状の状態によっては治験薬投与の延期を考慮し、細菌性感染症の発現にも注意することとする。また、「ポテリジオ®点滴静注 20mg」販売開始（2012 年 5 月 29 日）から 2013 年 2 月 28 日までに、「中毒性表皮壊死融解症」3 名 3 件、「スティーブンス・ジョンソン症候群」3 名 3 件が報告された。内 1 名は敗血症を併発し死亡に至ったことから、「中毒性表皮壊死融解症」及び「スティーブンス・ジョンソン症候群」を含む重度の皮膚障害について 2012 年 12 月 4 日に添付文書の使用上の注意を改訂した。

2) 注入に伴う反応/サイトカイン放出症候群及び腫瘍崩壊症候群

0761-0501 試験及び 0761-002 試験の結果、Mogamulizumab は他の抗体医薬と同様、投与時（特に 1 回目）に「発熱」、「悪寒」、「頻脈」等を主徴とする「注入に伴う反応」が高頻度に認められた〔全体の発現率 86%（37/43 名）、うち Grade 3 以上は 2%（1/43 名）〕。また、0761-0501 試験では「サイトカイン放出症候群」が 1 名に認められた。原疾患の悪化により有害事象の追跡不要と判断された被験者以外、いずれの事象も自然経過又は薬剤等の適切な処置により回復が確認されたが、これらの症状を軽減させるため、Mogamulizumab 投与前に抗ヒスタミン剤及び解熱鎮痛剤による予防投与を行うほか、症状が発現した際には適切な処置（輸液療法、酸素吸入、ステロイド処方等）を行うこととする。

また、0761-002 試験において、「腫瘍崩壊症候群」が 1 名（Grade 3）に発現した。「ポテリジオ®点滴静注 20mg」の市販直後調査（2012 年 5 月 29 日～2012 年 11 月 28 日）では、「腫瘍崩壊症候群」による死亡例が 1 名報告された。以上より、急激な腫瘍細胞破壊による TLS 発症への十分な注意が必要である。

3) 血液毒性（リンパ球数減少、好中球数減少、白血球数減少、血小板数減少）

0761-0501 試験及び 0761-002 試験において高頻度に認められた有害事象として、「リンパ球数減少」〔全体の発現率 95%（41/43 名）、うち Grade 3 以上は 70%（30/43 名）〕、「好中球数減少」〔全体の発現率 56%（24/43 名）、うち Grade 3 以上は 19%（8/43 名）〕、「白血球数減少」〔全体の発現率 65%（28/43 名）、うち Grade 3 以上は 23%（10/43 名）〕又は「血小板数減少」〔全体の発現率 53%（23/43 名）、うち Grade 3 以上は 12%（5/43 名）〕等の血球数減少の発現が挙げられる。原疾患の悪化及び他院転院等により有害事象の追跡不要と判断された被験者以外、いずれの事象も自然経過、G-CSF 処方又は血小板輸血の適切な処置により回復が確認された。なお、0761-0501 試験では一部の被験者に血小板機能検査（β-トロンボグロブリン及び血小板第 4 因子）が実施されたが、Grade 2 の血小板減少が認められた 1 名のβ-トロンボグロブリン及び血小板第 4 因子においても異常値は認められなかった。

以上より、血液毒性発現の際には必要に応じ適切な処置を行うこととする。

4) B 型肝炎ウイルス再活性化による肝炎及び劇症肝炎

Mogamulizumab 再投与を実施した 1 名において、「B 型肝炎」の発現が認められた。当該患者は治験参加前から HBc 抗体が陽性であったことが判明し、Mogamulizumab は B 型肝炎ウイルスを再活性化させ、B 型肝炎の発現に関与した可能性が否定できないと考えられた。

以上より、治験登録時の検査において HBs 抗原陽性若しくは HBV-DNA が検出された被験者は対象から除外し、HBc 抗体又は HBs 抗体が陽性と判定された被験者に対しては、治験期間中及び治験終了後の適切な時期に HBV-DNA の検査を実施するとともに、ウイルスの再活性化が確認された場合には、適切な処置（抗ウイルス薬処方）を行うこととした。

また、「ポテリジオ®点滴静注 20mg」販売開始（2012 年 5 月 29 日）から 2013 年 2 月 28 日までに、B 型肝炎ウイルス再活性化後に劇症肝炎を発現し、死亡に至った患者が 1 名報告された。

当該患者は慢性肝炎を合併する ATL 患者であり、化学療法後に HBs 抗原陽性となり、HBV-DNA 高値が認められた。エンテカビル併用にて本剤の投与を開始し、本剤 3 回目投与日の検査では肝酵素に顕著な異常は認められなかったが、本剤 4 回目投与のため来院した際に倦怠感と肝酵素の急激な上昇が認められた。

B 型肝炎ウイルスは本剤投与前に実施された化学療法により既に活性化されていたと考えられるが、本剤が B 型肝炎ウイルスの更なる活性化に関与した可能性は否定できないと考えられる。このため、肝機能検査値や肝炎ウイルスマーカーのモニタリングを随時行い、異常が認められた場合には適切な処置を行うこととする。

5） 感染症

国内外における「感染症および寄生虫症」の重篤な有害事象のうち、Mogamulizumab との因果関係が否定されなかった事象として、「肺炎」及び「サイトメガロウイルス性脈絡網膜炎」各 2 名、「口腔カンジダ症」、「感染」、「サイトメガロウイルス感染」、「サイトメガロウイルス性肺炎」、「ウイルス性脳炎」、「ヘルペス性食道炎」、「帯状疱疹」及び「B 型肝炎」各 1 名が報告されている。

また、「ポテリジオ®点滴静注 20mg」の市販直後調査（2012 年 5 月 29 日～2012 年 11 月 28 日）では、「サイトメガロウイルス感染」による死亡例が 1 名報告された。

以上より、感染症への十分な注意が必要であり、更に、必要に応じ、感染予防（抗結核薬、ST 合剤、抗真菌剤等）の適切な処置を行うこととする。

10-5. 副作用発現時の対処

10-5-1. 注入に伴う反応

注入に伴う反応が認められた場合は、直ちに投与の中断や投与速度の減速を考慮し、速やかに症状に応じた適切な処置を行う。

10-5-2. 皮膚障害

本剤投与開始時より皮膚科医と連携の上、皮膚障害があらわれた場合は適切な処置を行う。

短期間で症状が急速に進行し重症化する恐れがあるため、皮膚障害発現早期から副腎皮質ステロイド剤を十分量使用する等、適切な処置を行う。副腎皮質ステロイド剤の投与については、Grade 2 の段階から内服を考慮する。特に、粘膜障害を伴う場合や症状が進行中の場合は、投与初期から十分量（プレドニゾロン換算で 1mg/kg）を用いることが望ましく、場合によってステロイドパルス療法を実施する。

合併症が認められる場合は、合併症の対策をとりながら皮膚障害の対処を実施する。

皮膚障害が進行中の場合は、状態が落ち着くまで本剤の投与を延期する。

Grade2 以下で、中等量（プレドニゾロン換算で 0.5mg/kg）以下の副腎皮質ステロイド剤で皮膚障害をコントロールできている場合に限り、本剤の投与は延期せず継続を考慮する。

Grade 3 以上の皮膚障害が発現した場合は本剤の投与を中止し、適切な処置を行う。

本剤による皮膚障害は、四肢等の見える部分だけでなく、背部や胸部、衣類に隠れて見えない部分にあらわれることがあるため、全身の皮膚の状態を観察する。また、中毒性表皮壊死融解症及びスティーブンス・ジョンソン症候群では一般的に粘膜症状を有し、また粘膜症状が先行する場合もあるため、眼、口腔内、肛門、外陰部等の皮膚粘膜移行部も確認する。

重度の皮膚障害が本剤投与中だけでなく投与終了後数週間以降もあらわれることが報告されているため、投与終了後も継続して患者の全身の皮膚の状態を定期的に観察する。

なお、Grade 毎に推奨する対処法を付録 1 に示す。

10-5-3. B 型肝炎ウイルスによる肝炎

異常が認められた場合は本剤の投与を中止し、直ちに抗ウイルス剤を投与する等、適切な処置を行う。

10-5-4. 腫瘍崩壊症候群

異常が認められた場合は直ちに本剤の投与を中断し、適切な処置（生理食塩液、高尿酸血治療剤等の投与、透析等）を行うとともに、症状が回復するまで患者の状態を十分に観察する。

10-5-5. 血液毒性・肝機能障害

異常が認められた場合は、本剤の投与延期又は中止等の適切な処置を行う。

10-6. 妊娠が確認された場合の措置

被験者が妊娠した場合には、治験責任医師等は直ちに治験薬の投与を中止し、別途定める「安全性情報の取扱いに関する手順書」に従い治験調整委員会に報告する。治験調整委員会は、速やかに治験薬提供者に報告する。治験薬が避妊薬の効果を妨げたと疑われる場合以外は、妊娠そのものを有害事象とはみなさない。ただし、全ての妊娠について（被験者が治験を中止した場合を含む）、妊娠の転帰（自然流産、人工中絶、正常分娩又は先天性異常を伴う分娩）を追跡調査し、記録を残す。先天性異常、先天性欠損、及び自然流産は、重篤な有害事象として報告しなければならない。合併症を伴わない人工中絶は、有害事象として扱わない。妊娠の転帰は全て治験調整委員会へ報告し、治験調整委員会は、治験薬提供者に提出する。

10-7. 過量投与を行った場合の措置

被験者に対して過量投与を行った場合、別途定める「安全性情報の取扱いに関する手順書」に従い治験調整委員会に報告する。治験調整委員会は、速やかに治験薬提供者に報告する。

11. 統計解析

11-1. 解析対象集団

1) 安全性解析対象集団

第Ｉa 相部及び第Ｉb 相部それぞれについて、以下の条件を全て満たす被験者の集団とする。

(1) 適切にインフォームド・コンセントが得られていない等の重大な GCP 不遵守例に該当しな

い被験者

(2) 治験薬の投与を1回でも受けた被験者

2) 有効性解析対象集団（Full Analysis Set）

第Ｉa 相部及び第Ｉb 相部それぞれについて、以下の条件を全て満たす被験者の集団とする。

(1) 適切にインフォームド・コンセントが得られていない等の重大な GCP 不遵守例に該当しない被験者

(2) 治験薬の投与を1回でも受けた被験者

(3) 治験薬投与後に評価データを有する被験者

3) 薬物動態解析対象集団

安全性解析対象集団のうち、第Ｉa 相部の被験者とする。

11-2. 被験者の取扱い基準

1) 不適格例

選択基準に違反する症例、除外基準に抵触する症例を不適格例とする。

2) GCP 不遵守例

適切にインフォームド・コンセントが得られていない等の GCP 不遵守例に該当する被験者を GCP 不遵守例とする。

3) 中止・脱落例

同意撤回や有害事象発現及びその他治験責任医師等の判断で治験を中止した被験者を中止例とする。また、治験と直接関係のない理由により、来院しなくなった被験者等を脱落例とする。なお、治験薬を8回以上投与した被験者については、中止・脱落例として取り扱わない。

4) 処置違反・評価データ不完備例

用法・用量違反、併用薬違反等を処置違反例、評価データ不完備の被験者等を評価データ不完備例とする。

5) その他の問題症例

1）～4）以外の予測されなかった問題症例については治験調整委員会が必要に応じて自ら治験を実施する者及び統計解析専門家と検討し、統計解析上の取扱いを決定する。なお、この取り扱いの結果に及ぼす影響については総括報告書で報告する。

11-3. データの取扱い基準

治験実施計画書に規定した観察、検査日と実際の観察、検査日との間にズレがある場合、各項目は以下の基準内に測定された場合のみ採用とする。なお、日数は規定の予定来院日を0日とする。

1) PS

評価時期	ベースライン	投与日	9週	12週	維持投与時	投与中止時<br>治験中止時
採用幅	投与開始<br>28日以内*	投与日投与前	8回投与後<br>4～10日	8回投与後<br>25～31日	投与日投与前	中止決定日<br>＋14日以内

＊：登録日と同一曜日は可

2) 体重

評価時期	ベースライン	投与日	9 週	12 週	維持投与時	投与中止時<br>治験中止時
採用幅	1 週投与日－3 日<br>～<br>1 週投与日投与前	投与日－3 日<br>～<br>投与日投与前	8 回投与後<br>4～10 日	8 回投与後<br>25～31 日	投与日－3 日～<br>投与日投与前	中止決定日<br>＋14 日以内

3) バイタル

評価時期	ベース<br>ライン	投与日<br>投与前<br>**	投与開始<br>30 分､1 時間､<br>1.5 時間**	投与<br>終了時<br>**	投 与 終 了 後<br>30 分､1 時間<br>**	9 週	12 週	投与中止時<br>治験中止時
採用幅	投与開始<br>28 日以内*	投与前	±10 分以内	＋10 分<br>以内	±10 分以内	8 回投与後<br>4～10 日	8 回投与後<br>25～31 日	中止決定日<br>＋14 日以内

＊：登録日と同一曜日は可

＊＊：維持投与時を含む

4) 一般血液検査（採血日）

評価時期	ベースライン	投与日	9 週	12 週	維持投与時	投与中止時<br>治験中止時	最終投与後<br>12 週
採用幅	1 週投与日<br>－3 日～<br>1 週投与日<br>投与前	投与日－3 日<br>～<br>投与日投与前	8 回投与後<br>4～10 日	8 回投与後<br>25～31 日	投与日－3 日<br>～<br>投与日投与前	中止決定日<br>＋14 日以内	規定来院日<br>＋14 日以内

5) 甲状腺機能検査（採血日）

評価時期	ベースライン	12 週	投与中止時<br>治験中止時
採用幅	1 週投与日－3 日<br>～<br>1 週投与日投与前	8 回投与後<br>25～31 日	中止決定日<br>＋14 日以内

6) 腫瘍マーカー（採血日）

評価時期	ベースライン	12 週	13 週～<br>（12 週又は 9 回目規定来院日<br>より 12 週毎に実施）	投与中止時<br>治験中止時
採用幅	投与開始<br>3 日以内	規定来院日<br>±3 日以内	規定来院日<br>±14 日以内	中止決定日<br>±14 日以内

7) 尿検査

評価時期	ベースライン	2 週	12 週	維持投与時<br>（12 週毎に実施）	投与中止時<br>治験中止時
採用幅	投与開始<br>3 日以内	投与日－3 日～<br>投与日投与前	8 回投与後<br>25～31 日	投与日－3 日～<br>投与日投与前	中止決定日<br>＋14 日以内

8) CT 又は MRI

評価時期	ベースライン	12 週～	治験中止時
採用幅	投与開始<br>28 日以内	規定来院日<br>±14 日以内	中止決定日±14 日以内

9)　抗原特異的抗体免疫反応検査（採血日）

評価時期	ベースライン	5 回目投与日	9 週	13 週～<br>（12 週又は 9 回目規定来院日より 4 週毎に実施）	投与中止時<br>治験中止時
採用幅	投与開始<br>3 日以内	投与日－3 日～<br>投与日投与前	8 回投与後<br>4～10 日	規定来院日<br>－13 日～＋14 日	中止決定日<br>±14 日以内

10) 制御性 T 細胞検査（採血日）

評価時期	ベースライン	5 回目投与日	9 週	13 週～<br>（12 週又は 9 回目規定来院日より 4 週毎に実施）	投与中止時<br>治験中止時
採用幅	投与開始<br>3 日以内	投与日－3 日～<br>投与日投与前	8 回投与後<br>4～10 日	規定来院日<br>－13 日～＋14 日	中止決定日<br>±14 日以内

11) 薬物動態（採血日時）

評価時期	投与日<br>投与前	投与終了時	投与終了後<br>24～72 時間	投与終了後<br>7 日	投与終了後<br>14～28 日
採用幅	－1 時間以内	＋10 分以内	±1 時間以内	±5 時間以内	規定来院日<br>±1 日以内

12) 免疫増強効果検査（採血日）

評価時期	ベースライン	5、7 回目投与日	9 週	12 週	13 週～<br>（12 週又は 9 回目規定来院日より 4 週毎に実施）	投与中止時<br>治験中止時
採用幅	投与開始<br>14 日以内	投与日－3 日～<br>投与日投与前	8 回投与後<br>4～10 日	8 回投与後<br>25～31 日	規定来院日<br>－13 日～＋14 日	中止決定日<br>±14 日以内

13) 遺伝子解析（採血日）

評価時期	ベースライン
採用幅	投与開始<br>14 日以内

11-4. 解析項目・方法

詳細な解析方法については別途作成する「統計解析計画書」及び「薬物動態解析計画書」に記載し、以下に解析の概要を示す。

1)　安全性

(1)　DLT 発現例数

第 I a 相部の投与群毎の DLT 発現例数と発現割合を示す。

(2)　有害事象及び副作用の発現状況

第 I a 相部及び第 I b 相部において発現した有害事象を一覧表にまとめる。全ての有害事象及び副作用は MedDRA の基本語と器官別大分類別に集計した各投与群毎の発現例数と発現割合を示す。

(3)　遺伝子解析

必要に応じて探索的に検討する。

2) 有効性

(1) 制御性 T 細胞除去効果

投与群毎に要約統計量を算出する。

(2) 抗原特異的抗体免疫反応

投与群毎に探索的に検討する。

(3) 抗腫瘍効果

投与群毎に奏効症例数と奏効例の割合（奏効率）を示す。

(4) 無増悪生存期間（PFS）

無増悪生存期間は、Kaplan-Meier 法を用いて計算する。

(5) 全生存期間（OS）

全生存期間は、Kaplan-Meier 法を用いて計算する。

(6) 免疫増強効果

必要に応じて探索的に検討する。

(7) 遺伝子解析

必要に応じて探索的に検討する。

また、制御性 T 細胞除去効果と抗腫瘍効果、抗原特異的抗体免疫反応等の関連について探索的に検討する。

3) 薬物動態

(1) 血漿中 Mogamulizumab 濃度

Mogamulizumab の血漿中濃度は一覧とし、投与量毎の記述的統計量を示す。Mogamulizumab の血漿中濃度推移は、実数及び対数スケールによって、採血時間に対する被験者個々のデータ及び平均値（＋標準偏差）をグラフ化する。

(2) Mogamulizumab 薬物動態パラメータ

「8-3-1 主要評価項目の定義　1)　第 I a 相部　(2) 薬物動態」に示した各被験者の薬物動態パラメータを算出し、投与量毎の記述的統計量を算出する。

12. ゲノム・遺伝子解析の実施

12-1. 目的

適切な同意に基づき被験者から採取した試料を用いて、治験薬の有効性、安全性等を探索的且つ基礎的に検討することを目的とし、「8-3-3. その他の項目の定義　1)　第 I a 相部及び第 I b 相部共通　(8) 遺伝子解析（任意）」に示すゲノム・遺伝子解析を実施する。

12-2. 試料の取り扱い

ゲノム・遺伝子解析に用いる試料については、「9-1. 治験スケジュール」に従って採取する血液とする。

被験者から採取した試料を検査機関（別冊 1「治験実施体制」参照）に送付する際は、その匿名性を確保するために、被験者の特定は全て被験者識別コードのみで行う。

試料については、所定の検査機関にて、治験調整委員会からの要請があるまで適切な条件で厳

重に保存する。保存期間中、適切に保存できていることを定期的に確認し、廃棄するまで記録を残すこととする。廃棄する場合には、検査機関の規定に従い適切に廃棄し、実施医療機関へ試料を廃棄した旨を文書で報告する。

なお、ゲノム・遺伝子解析を実施する検査機関が試料を保存している検査機関と異なる場合は、試料の送付について手順を定め、その手順に従って行うこととする。試料の保管に関しては、治験調整委員会からの要請があるまで適切な条件で厳重に保存し、保存期間中、適切に保存できていることを定期的に確認し、廃棄するまで記録を残すこととする。廃棄する場合には、ゲノム・遺伝子解析を実施する検査機関の規定に従い適切に廃棄し、実施医療機関へ文書で報告する。

12-3. 解析方法

Mogamulizumab の臨床効果や毒性（自己免疫性有害事象：皮疹、間質性肺炎等）の発症を予測しうるゲノムバイオマーカーを探索的に検討するため、治療開始前の末梢血単核球由来の DNA を用いた遺伝子多型解析とコピー数多型解析を用いた Genome-wide association study(GWAS)、及び血清中 miRNA の網羅的発現解析を行う。

12-4. 試料提供に関する同意について

ゲノム・遺伝子解析に用いる試料の使用に関して、被験者となるべき者に予め十分な説明を行った上で、文書による同意を取得する。その際の留意事項について「5-3. 同意に関する留意事項」に示す。治験の参加に関して同意撤回の申し出があった場合においても、その時点までに採取された試料については、原則としてゲノム・遺伝子解析に利用できるものとし、ゲノム・遺伝子解析を目的とした試料の使用について同意を撤回する場合は、別途文書による同意撤回書の提出を必要とする。ゲノム・遺伝子解析を目的とした試料の使用について被験者より文書での同意撤回の申し出を受けた際は、原則として当該被験者に係る試料及び結果については、匿名性を確保した上で廃棄し、その旨を実施医療機関を介して当該被験者に文書にて報告することとする。ただし、同意撤回の時点で、既に検査結果が論文等で公表されていた場合等は、廃棄できないものとする。

12-5. 被験者への情報の開示

被験者から採取した試料を用いて実施されるゲノム・遺伝子解析の方法及び結果（以下、治験情報という）に関して、被験者が希望した場合は治験情報を開示する。なお、治験情報の開示については、被験者より文書にて開示請求があった場合に限り、実施医療機関を介して被験者へ治験情報を開示する。

13. 治験実施計画書の遵守及び逸脱、変更ならびに改訂

治験責任医師等は、治験審査委員会の事前の審査に基づく文書による承認を得ることなく、治験実施計画書からの逸脱又は変更を行わない。

治験責任医師等は、被験者の緊急の危険を回避する等、医療上やむを得ない事情のために、治験審査委員会の事前の承認なしに治験実施計画書からの逸脱又は変更を行うことができる。その

際には、治験責任医師は、逸脱又は変更の内容及び理由ならびに治験実施計画書の改訂が適切である場合にはその案を可能な限り早急に実施医療機関の長、治験審査委員会に提出してその承認を得る。

自ら治験を実施する者は、被験薬の品質、有効性及び安全性に関する事項その他の治験を適正に行うために重要な情報を知ったときは、必要に応じ、治験実施計画書を改訂しなければならない。

14. 治験の中止と終了

14-1. 個々の被験者に対する中止規定又は中止基準

14-1-1. 個々の被験者に対する治験薬の投与中止基準

「7-7-3. 投与中止基準」に該当した場合は直ちに治験薬の投与を中止し、治験責任医師等は適切な処置を実施し、中止時の臨床症状を観察し、一般臨床検査を行う。さらに、中止日、中止理由、中止後の処置及び経過等を症例報告書に記入する。来院予定日に来院せず、治験薬の投与が出来なくなった場合には、治験責任医師等はその理由とその後の経過について、電話又は封書等で追跡調査を行い、患者の状況を確認し、その内容を症例報告書に記入する。

14-1-2. 個々の被験者に対する治験の中止基準

以下の基準に該当した場合は治験薬の投与を中止するだけでなく、治験に係る評価も中止する。

1) 同意を撤回した場合
2) 被験者が死亡した場合
3) 来院予定日に来院せず、治験の継続ができなくなった場合
4) その他、治験の継続が適当でないと治験責任医師等が判断した場合

14-1-3. 実施医療機関での中止又は中断

自ら治験を実施する者は、実施医療機関が GCP 省令及び治験実施計画書に違反することにより適正な治験に支障を及ぼしたと認める場合（被験者の緊急の危険を回避するためその他医療上やむを得ない理由による場合を除く）には、治験調整委員会にその旨を事前に通知した上で、当該実施医療機関における治験を中止する。自ら治験を実施する者は、治験を中断し、又は中止する場合には、速やかにその旨及びその理由を実施医療機関の長及び治験調整委員会事務局を通じて治験調整委員会に文書で詳細に報告する。治験調整委員会は、治験調整委員会事務局を通じて、他の実施医療機関の自ら治験を実施する者に速やかにその旨及びその理由を通知する。また、不遵守のため治験を中止した場合には、自ら治験を実施する者は規制当局に速やかに報告する。

実施医療機関の長は、自ら治験を実施する者から、治験を中断し、若しくは中止する旨を報告してきた場合は、治験審査委員会に対し速やかに文書で通知し、詳細に説明する。

14-2. 治験の一部及び全体の中断、又は中止規定

1) 治験の一部中止

自ら治験を実施する者は被験者の安全性に影響を及ぼし、治験の実施に影響を与え、又は治験

継続に関する治験審査委員会の承認を変更する可能性のある情報を得た場合は、治験調整委員会と協議のうえ、治験の一部の中断、又は中止を検討する。

自ら治験を実施する者は治験の一部の中断、又は中止を決定した場合は、速やかにその旨及びその理由を実施医療機関の長、及び治験調整委員会事務局を通じて治験調整委員会に文書で詳細に報告する。また、規制当局に対してその旨及びその理由を通知するものとする。

2) 治験全体の中止

自ら治験を実施する者は被験者の安全性に影響を及ぼし、治験の実施に影響を与え、又は治験継続に関する治験審査委員会の承認を変更する可能性のある情報を得た場合は、治験調整委員会と協議のうえ、治験全体の中断、又は中止を検討する。

14-3. 治験の終了

最終症例の投与開始から1年後もしくは治験薬の最終投与24週後のいずれか遅い時点をもって治験終了とする。

治験責任医師等は治験終了後、実施医療機関の長に治験が終了した旨を文書で通知し、治験結果の概要を文書で報告する。

15. 症例報告書等の記入と提出

15-1. 提出資料

治験責任医師は、以下の資料をデータセンターに提出する。なお、登録割付された全症例を対象とする。

1) 臨床検査の基準値一覧
2) 署名印影一覧表

   症例報告書の作成、変更、修正を行う治験責任医師、治験分担医師及び必要に応じ治験協力者の署名と印影を示した文書。

3) 症例報告書
4) DCF（Data Clalification Form）
5) 原資料との矛盾を説明した記録

   症例報告書中のデータが原資料と何らかの矛盾がある場合、治験責任医師がその理由を説明した文書。

6) 緊急の危険を回避するための治験実施計画書からの逸脱記録（写し）

   被験者の緊急の危険を回避するためその他医療上やむを得ない理由により治験実施計画書に従わなかった場合、その旨及びその理由を記載した文書。

15-2. 様式と提出期限

症例報告書（CRF）は、治験責任医師等が適切な時期に記載し、速やかにデータセンターに提出する。

15-3. 記入方法

記載はペンにより行い、訂正時は、訂正日を記し、訂正者が署名、又は捺印する。重大な変更の場合はその理由を記載する。

15-4. 症例報告書の作成及び送付

治験責任医師等は、症例報告書に必要な内容を記入する。原本は、治験調整委員会が保管する。

治験責任医師は、原本のコピーを作成し保管する。

15-5. 症例報告書の変更又は修正

症例報告書の変更又は修正は、別途定める「症例報告書の作成、変更又は修正に関する手順書」に従い、下記の通り行う。

1) 変更又は修正の際には、当初の記載がわかるように修正液等は使用せず、該当部分を二重線で消し、該当部分のすぐ近くに正しい事項を記入し、変更又は修正を行った日付を併記の上、捺印又は署名する。
2) 重大な変更又は修正については、その理由を併記する。
3) 捺印する際には、署名印影一覧表と同一のものを用いる。

15-6. Data Clarification Form(DCF)の発行・提出等

データセンターが症例報告書に関する疑義事項について治験責任医師に問い合せを行う場合には、DCF を治験責任医師に発行する。治験責任医師は、DCF が発行された場合、速やかに DCF の回答を作成し、確認日、記名捺印又は署名した上で、データセンターに提出する。また、症例報告書の変更又は修正が必要な場合には、その項目毎に変更又は修正後のデータが明確になるように DCF に記載する。なお、治験分担医師が回答を作成した場合には、治験責任医師は記入内容を点検し、問題がないことを確認した上で、確認日を記入し、記名捺印又は署名し、データセンターに提出する。また、治験責任医師は提出した DCF の写しを保存する。

15-7. 症例報告書と診療録等の原資料に矛盾がある場合

治験責任医師は症例報告書の記載内容の中で、診療録等の原資料との何らかの矛盾がある場合には、その理由を説明する記録として「原資料との矛盾を説明した記録」を作成する。治験責任医師は作成した「原資料との矛盾を説明した記録」をデータセンターに提出し、その写しを保存する。

15-8. 症例報告書及びデータの取扱い

実施医療機関は、症例報告書又は検査報告書、あるいはその写しの取り扱いに関して、個人情報の保護に細心の注意を払い、情報の漏洩、紛失、転記、不正な複写等がないように行う。

16. 原資料の閲覧に関する事項

自ら治験を実施する者及び実施医療機関の長は、保管義務のある記録等の調査・確認のためモ

ニター、監査担当者、治験審査委員会及び規制当局による査察又は直接閲覧に応じるものとする。

また、症例報告書に直接記入され、且つ原資料と解すべきデータとその内容を以下に示す。

1) 登録症例としての適格性
2) 併用療法の併用理由
3) 一般臨床検査の正異判定及び、異常変動の有無
4) 有害事象（臨床検査値異常変動を含む）のグレード、治験薬との因果関係及び重篤／非重篤の判定
5) 抗腫瘍効果の評価
6) 個々の被験者の治験薬の投与中止の理由、投与中止後の処置及びその後の経過、治験中止の理由、治験中止後の処置及びその後の経過
7) コメント

17. 治験の品質管理及び品質保証

治験の品質管理及び品質保証は、別途定める「治験実施に係る標準業務手順書」に従い行う。

18. 倫理的配慮

18-1. 遵守すべき諸規則

本治験は、「ヘルシンキ宣言」ならびに、且つ本治験実施計画書、薬事法第 14 条第 3 項及び第 80 条の 2 に規定する基準及び「医薬品の臨床試験実施の基準に関する省令（GCP）」（厚生省令第 28 号）に則り実施する。

18-2. 治験審査委員会

本治験は、実施に先立ち、各実施医療機関の治験審査委員会において、治験実施計画書、被験者の同意を得るのに使用される方法、治験薬概要書及びその他の必要な文書を審議し、本治験が倫理的及び科学的に妥当であるかどうか、その他、本治験が実施医療機関において行うのに適当であるかどうかを審査する。

継続審査

1) 治験責任医師は、治験審査委員会の継続審査を受けるために、治験の現状の概要を年に 1 回又は治験審査委員会の求めに応じてそれ以上の頻度で、実施医療機関の長に文書をもって提出する。
2) 実施医療機関の長は当該実施医療機関における治験の現況の報告、重篤で予測できない副作用等について自ら治験を実施する者から通知を受けた場合、重篤な有害事象について治験責任医師から通知を受けた場合、及び治験について継続して参加するかどうかについて被験者の意思に影響を与える情報を得た場合、当該治験の継続について治験審査委員会に意見を求めるものとする。

18-3. インフォームド・コンセント

治験責任医師等は、被験者に治験について十分に説明し、文書による同意を得る。被験者が熟読し保管できるように、治験の詳細を示した説明文書を被験者に渡す。被験者に情報を十分に考慮する時間を与え、疑問点を確認した後で、同意文書に署名及び日付を記入する。

18-4. 新たな情報の提供

自ら治験を実施する者は、被験者の安全性に悪影響を及ぼし、治験の実施に影響を与え、又は治験審査委員会の承認を変更する可能性のある情報、あるいは重篤で予測できない副作用等の情報を入手した場合は、治験審査委員会に速やかに文書で報告する。自ら治験を実施する者は同意文書及びその他の説明文書を改訂する必要があると認めた場合は、速やかに改訂する。

18-5. 被験者の機密保持・プライバシー確保

被験者の登録及び症例報告書における被験者の特定はデータ・試料管理担当者によって、被験者識別コード等で行う等連結可能匿名化を行なう。原資料の直接閲覧・取り扱い等においては被験者のプライバシー保護に十分配慮する。被験者試料等も、同様に被験者識別コード等で連結可能匿名化を行うとともに、他施設への試料の移送等に際しては、この被験者識別コード等にて識別する。

19. 必須文書の保存

19-1. 自ら治験を実施する者

自ら治験を実施する者は別途定める「記録の保存に関する手順書」に従い、本治験に関する記録を以下の 1）又は 2）のうち、いずれか遅い日までの期間適切に保存する。なお、当該記録の保存について、自ら治験を実施する者が所属する実施医療機関の長にその業務を依頼することができる。また、当該自ら治験を実施する者がその所属する実施医療機関に所属しなくなった場合については、その所属する実施医療機関の長が当該記録の保存業務を担うことができる。

また、これらの資料について治験調整委員会がこれよりも長期間の保存を必要と判断する場合には、保存期間及び保存方法について治験調整委員会とその対応について協議する。

自ら治験を実施する者は、保管の必要がなくなった場合には、その旨を実施医療機関の長又は実施医療機関の長を経由して、治験審査委員会の設置者に通知しなければならない。

1) 当該被験薬に係る製造販売承認日（GCP 省令第 26 条の 10 第 2 項及び第 3 項の規定により開発の中止若しくは治験の成績が承認申請書に添付されない旨の通知を受けた場合には開発中止が決定された若しくは申請書に添付されない旨の通知を受けた日から 3 年が経過した日）
2) 本治験の中止もしくは終了の後 3 年が経過した日

19-2. 実施医療機関

実施医療機関の長又は治験審査委員会の設置者が保存すべき必須文書は、実施医療機関の長が定めた記録保存責任者が適切に保管する。保存期間は以下の 1）又は 2）のうちいずれか遅い日ま

でとする。

1) 当該被験薬に係る製造販売承認日（GCP 省令第 26 条の 10 第 2 項及び第 3 項の規定により開発の中止若しくは治験の成績が承認申請書に添付されない旨の通知を受けた場合には開発中止が決定された若しくは申請書に添付されない旨の通知を受けた日から 3 年が経過した日）
2) 本治験の中止もしくは終了後 3 年が経過した日

19-3. 治験責任医師

治験責任医師は、治験に係る文書を実施医療機関の長の指示に従って保存する。

20. 金銭の支払い及び保険

20-1. 金銭の支払い

被験者の本治験に係る負担軽減費については、症例登録後の治験のための来院について、各実施医療機関の取り決めに従い、被験者へ支払うか否かを各実施医療機関で決定する。

20-2. 補償

本治験において何らかの健康被害を認めた場合には、速やかに適切な診断と処置を医療健康保険の範囲内で行う。しかし、本治験に起因した健康被害に対する補償金は支払われない。

21. 資金源及び起こりうる利害の衝突

Mogamulizumab（抗 CCR4 抗体）は、協和発酵キリン株式会社により無償提供される。その他の費用は公的研究費により支払われる。

22. 試験成果の帰属と結果の公表

22-1. 結果の公表

試験終了後、その成果をまとめ、しかるべき国内外の学会及び英文誌に発表する。

22-2. 総括報告書の作成

最終症例の投与開始から 1 年までのデータが固定された時点及び治験終了後に、自ら治験を実施する者は別途定める「総括報告書の作成に関する手順書」に従い、総括報告書を作成する。

22-3. データの提供

試験終了後、規制当局の指示・指導もしくは関係企業等の希望により、個人情報を除いた本試験データを有償又は無償で提供することがある。

22-4. データの二次利用

本試験で得られたデータを二次利用することが有益であると治験調整委員会が判断した場合は、

個人情報の保護に細心の注意を払い、データの二次利用をできる。

23. 参考文献

1）がん研究振興財団．がんの統計 2005 年版

2）The Japan Society for Esophageal Disease. Comprehensive Registry of Esophageal Cancer in Japan（1998、1999）and long-term results of esophagectomy in Japan（1988-1997）3rd Edition

3）Dunn GP, Bruce AT, Ikeda H, Old LJ, Schreiber RD. Cancer immunoediting: from immunosurveillance to tumor escape. Nat Immunol. 2002;3:991-8.

4）Wada H, Sato E, Uenaka A, et al. Analysis of peripheral and local anti-tumor immune response in esophageal cancer patients after NY-ESO-1 protein vaccination. Int J Cancer. 2008;123:2362-9.

5）Hodi S, O'Day SJ, McDermott DF, et al. Improved survival with ipilimumab in patients with metastatic melanoma. N Engl J Med. 2010; 363: 711-723.

6）Yuan J, Adamow M, Ginsberg BA, et al. Integrated NY-ESO-1 antibody and CD8+ T-cell responses correlate with clinical benefit in advanced melanoma patients treated with ipilimumab. Proc Natl Acad Sci U S A. 2011;108:16723-8.

7）Onizuka S, Tawara I, Shimizu J, et al. Tumor rejection by in vivo administration of anti-CD25 (interleukin-2 receptor alpha) monoclonal antibody. Cancer Res. 1999;59:3128-33.

8）Miyara M, Sakaguchi S.Human. FoxP3(+)CD4(+) regulatory T cells: their knowns and unknowns. Immunol Cell Biol. 2011 ;8:346-51.

9）Ishida T, Ueda R. Antibody therapy for Adult T-cell leukemia-lymphoma. Int J Hematol. 2011;94:443-52.

10）Yamamoto K, Utsunomiya A, Tobinai K, et al. Phase I study of KW-0761, a defucosylated humanized anti-CCR4 antibody, in relapsed patients with adult T-cell leukemia-lymphoma and peripheral T-cell lymphoma. J Clin Oncol 2010; 28: 1591-1598.

11）Ishida T, Joh T, Uike N, et al. Defucosylated anti-CCR4 monoclonal antibpdy (KW-0761) for relapsed adult T-cell leukemia-lymphoma: a multicenter phase II study. J Clin Oncol 2012; 30: 837-842.

12）E.A. Eisenhauer, P. Therasse, J. Bogaerts, et al. New response evaluation criteria in solid tumours:Revised RECIST guideline (version 1.1). European Journal of Cancer. 2009;45:228-247.

13）Japan Clinical Oncology Group. 有害事象共通用語規準 v4.0 日本語訳 JCOG 版 – 2011 年 12 月 17 日.

付録 1. 皮膚障害が認められた場合に推奨する対処法

（協和発酵キリン株式会社提供、「ポテリジオの皮膚障害対策　臨床医が推奨する対処法」より）

<table>
<tr><th colspan="2">薬効分類</th><th>Grade 1</th><th>Grade 2</th><th>Grade 3</th><th>Grade 4</th></tr>
<tr><td rowspan="4">副腎皮質ステロイド剤（外用）</td><td rowspan="2">very strong（かなり強力）</td><td colspan="4">【体幹四肢】・0.05%ベタメタゾン酪酸エステルプロピオン酸エステル、
・0.1%モメタゾンフランカルボン酸エステル等　1日2回塗布</td></tr>
<tr><td colspan="4">副腎皮質ステロイド剤の内服を開始後は適宜中止可能</td></tr>
<tr><td rowspan="2">strong（強力）</td><td colspan="4">【顔面】※1・0.12%ベタメタゾン吉草酸エステル、
・0.025%ベクロメタゾンプロピオン酸エステル等　1日2回塗布</td></tr>
<tr><td colspan="4">副腎皮質ステロイド剤の内服を開始後は適宜中止可能</td></tr>
<tr><td colspan="2" rowspan="2">副腎皮質ステロイド剤（内服）※2</td><td>−</td><td>プレドニゾロン
0〜60mg/日※3
（0〜1mg/kg）</td><td colspan="2">プレドニゾロン
60mg/日以上
（1mg/kg以上）</td></tr>
<tr><td colspan="4">皮膚症状を観察しながら適宜漸減</td></tr>
<tr><td colspan="2" rowspan="2">抗ヒスタミン剤</td><td colspan="4">オロパタジン塩酸塩10mg/日内服等</td></tr>
<tr><td colspan="4">d-クロルフェニラミンマレイン酸塩 8mg/日内服等</td></tr>
<tr><td colspan="2">副腎皮質ステロイド剤（静注）【ステロイドパルス療法】※4</td><td>−</td><td colspan="3">メチルプレドニゾロン1,000mg/body、3日間
（・体格や全身状態を考慮して、500mg/日で投与することもある
・症状が回復しない場合はもう1クール追加することもある）</td></tr>
<tr><td colspan="2">その他の治療法</td><td>−</td><td>−</td><td>−</td><td>・血漿交換療法
・ヒト免疫グロブリン製剤静注(IVIG)療法</td></tr>
</table>

Grade　は有害事象共通用語基準（CTCAE）v3.0　日本語訳 JCOG/JSCO　版に準ずる

副腎皮質ステロイド剤の全身投与が長期化する場合は、日和見感染のリスクが高まることに十分に注意する

※1：顔面への副腎皮質ステロイド剤（外用）の長期間の使用は避ける
※2：副腎ステロイド剤の内服は、漫然とした長期投与は避け、皮膚障害の状況に応じて減量を検討すること
※3：粘膜障害を伴う場合や症状が進行中の場合は 1mg/kg を投与
※4：Grade 2　では、中毒性表皮壊死融解症/Stevens-Johnson 症候群（TEN/SJS）が疑われるような粘膜障害を伴う場合に実施。ステロイドパルス療法終了後は副腎皮質ステロイド剤の内服を継続する
